# Transcriptional override: a regulatory network model of indirect responses to modulations in microRNA expression

**DOI:** 10.1186/1752-0509-8-36

**Published:** 2014-03-25

**Authors:** Christopher G Hill, Lilya V Matyunina, DeEtte Walker, Benedict B Benigno, John F McDonald

**Affiliations:** 1 Integrated Cancer Research Center, School of Biology, and Parker H. Petit Institute of Bioengineering and Biosciences, Georgia Institute of Technology, 315 Ferst Dr, Atlanta, GA 30332, USA; 2 Ovarian Cancer Institute, 960 Johnson Ferry Road Suite 130, Atlanta, GA 30342, USA

**Keywords:** Cancer systems biology, Feed-forward loops, Gene regulation, miRNAs, Ovarian cancer

## Abstract

**Background:**

Documented changes in levels of microRNAs (miRNA) in a variety of diseases including cancer are leading to their development as early indicators of disease, and as a potential new class of therapeutic agents. A significant hurdle to the rational application of miRNAs as therapeutics is our current inability to reliably predict the range of molecular and cellular consequences of perturbations in the levels of specific miRNAs on targeted cells. While the direct gene (mRNA) targets of individual miRNAs can be computationally predicted with reasonable degrees of accuracy, reliable predictions of the indirect molecular effects of perturbations in miRNA levels remain a major challenge in molecular systems biology.

**Results:**

Changes in gene (mRNA) and miRNA expression levels between normal precursor and ovarian cancer cells isolated from patient tissue samples were measured by microarray. Expression of 31 miRNAs was significantly elevated in the cancer samples. Consistent with previous reports, the expected decrease in expression of the mRNA targets of upregulated miRNAs was observed in only 20-30% of the cancer samples. We present and provide experimental support for a network model (The Transcriptional Override Model; TOM) to account for the unexpected regulatory consequences of modulations in the expression of miRNAs on expression levels of their target mRNAs in ovarian cancer.

**Conclusions:**

The direct and indirect regulatory effects of changes in miRNA expression levels in vivo are interactive and complex but amenable to systems level modeling. Although TOM has been developed and validated within the context of ovarian cancer, it may be applicable in other biological contexts as well, including of potential future use in the rational design of miRNA-based strategies for the treatment of cancers and other diseases.

## Background

Human miRNAs regulate gene expression post-transcriptionally by degrading target mRNAs and/or blocking their translation [1]. As a consequence, mRNA expression changes are expected to be inversely correlated (IC) with changes in levels of their targeting miRNAs. Although this expectation has been validated in studies of individual miRNAs and specific mRNA targets, the expected inverse relationship is often not observed in global transcriptome level studies [2-4]. While these unexpected findings may, in some instances, be attributed to inaccuracies in miRNA target prediction algorithms [5], recent evidence suggests that many of the unexpected regulatory effects may be the result of feed-back or feed-forward loops and/or other system level complexities [3,6].

## Results

To systematically address the relationship between miRNAs and their regulated mRNA targets in the same cellular context, we employed microarray gene expression profiling to compare differences in expression levels of mRNAs and miRNAs in ovarian surface epithelial cells (OSE) vs. serous papillary ovarian cancer epithelial cells (CEPI) isolated from patient tissues (Additional file 1) by laser capture microdissection. Gene expression profiling identified 5910 significantly differentially expressed genes (mRNAs) between OSE and CEPI (Additional file 2). Of these, 2232 (38%) were upregulated and 3678 (62%) downregulated in CEPI. MiRNA expression profiling identified 31 significantly differentially expressed miRNAs between OSE and CEPI. All of these miRNAs were upregulated in CEPI (Table 1).

**Table 1:** MiRNA expression profiling identified 31 significantly differentially expressed miRNAs between OSE and CEPI

**MicroRNA**	**P-value**	**Fold-change**	**MicroRNA**	**P-value**	**Fold-change**
MIR7	0.00004	25.81	MIR106b	0.00204	32.90
MIR10a	0.00305	28.25	MIR128	0.00542	15.78
MIR17	0.01120	14.12	MIR130b	0.03129	11.71
MIR18a	0.00001	38.05	MIR141	0.00000	118.60
MIR18b	0.00000	46.21	MIR143	0.00792	5.98
MIR19a	0.00001	41.64	MIR148a	0.00406	10.70
MIR19b	0.00103	61.39	MIR148b	0.01725	7.94
MIR20a	0.00707	14.42	MIR155	0.00179	35.75
MIR20b	0.03363	19.16	MIR181d	0.02989	8.40
MIR21	0.01296	14.12	MIR182	0.00046	58.49
MIR25	0.01993	5.58	MIR200a	0.00264	39.12
MIR29b	0.00874	23.10	MIR200b	0.01871	14.52
MIR30b	0.01763	11.55	MIR200c	0.00232	13.36
MIR30c	0.01292	26.54	MIR205	0.00018	67.18
MIR93	0.02678	9.58	MIR429	0.00181	21.11
MIR106a	0.00505	20.53			

Expression profiles for miRNAs in OSE and CEPI samples were determined using the miRChip system (Asuragen Inc.). Human probe sets with >65% present calls in either of the two groups (OSE and CEPI) were selected for analysis. All reported microarray data are described in accordance with MIAME guidelines. The processed and raw data files for the samples used in this study have been deposited in the Gene Expression Omnibus (GEO) as SuperSeries GSE42460.

Employing three commonly used miRNA target prediction algorithms (miRanda-mirSVR [7], TargetScan [8], SVMicrO [9]), we identified putative mRNA targets of these 31 miRNAs to determine if differences in their levels of expression between the OSE and CEPI samples were IC, positively correlated (PC) or unchanged (NC). Based on the established molecular mechanism of miRNA regulation, levels of miRNAs are expected to be IC with levels of their target mRNAs. Contrary to this expectation and consistent with previous findings [3], we observed a consistently low percentage (23-31%) of target mRNAs displaying expression level changes IC with their regulating miRNAs (Table 2). Since predictions from the most commonly employed mirSVR algorithm were representative of the results from all tested algorithms, mirSVR was employed in subsequent analyses.

**Table 2:** Percentage of miRNA target genes displaying changes in expression between OSE and CEPI

**Algorithm**	**Inversely correlated**	**Positively correlated**	**No change detected**	**Total**
miRanda-mirSVR	24% (3110)	13% (1719)	62% (8015)	12844
TargetScan	31% (1519)	16% (779)	54% (2681)	4979
SVMicrO	23% (2027)	13% (1164)	64% (5787)	8978

Predicted gene targets of miRNAs were computed independently using three commonly used prediction algorithms: miRanda-mirSVR [7], TargetScan [8] and SVMicrO [9]. For each prediction algorithm, the frequency of target genes displaying changes in gene expression IC, PC or NC with changes in expression of their regulating miRNAs are shown. The number of predicted target genes falling into each class is presented in parentheses.

**Figure 1:**
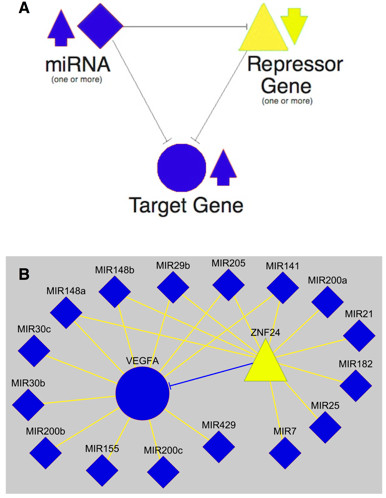
**The transcriptional override model (TOM).** The network motif **(A)** typifies how the expected downregulation of target genes by elevated levels of regulating miRNAs may be masked or overridden by target gene derepression mediated by miRNA-induced downregulation of repressor genes. **(B)** MiRNA-mediated derepression of repressor gene *ZNF24* (yellow triangle) overrides the expected downregulatory effects of miRNAs (blue diamonds) on VEGFA expression (blue circle).

The **t**ranscriptional **o**verride **m**odel (TOM) postulates that downregulation of target genes induced by elevated levels of regulating miRNAs may be masked (NC) or overridden (PC) by increases in expression mediated by the downregulation of repressor genes that are themselves targets of upregulated miRNAs (Figure 1A). The possibility of such feed-forward loops was prompted by the fact that several of the predicted mRNA targets of the 31 overexpressed miRNAs encode documented repressors of gene expression (e.g. *ZNF24*[10], *YY1*[11], *SPEN*[12], *BACH1*[13]). MiRNA-mediated downregulation of these repressor genes would be expected to result in the derepression of their respective target genes and a consequent increase in levels of expression. If these derepressed gene targets were also the targets of upregulated miRNAs, the expected downregulation of these genes by the miRNAs (IC) could be masked (NC) or overridden (PC). For example (Figure 1B), one of the predicted targets of ten of the 31 miRNAs upregulated in cancer is the well-documented repressor gene *ZNF24*[10]. Consistent with the fact that *ZNF24* is targeted by upregulated miRNAs, its expression in CEPI is significantly reduced. An experimentally validated target of *ZNF24* is *VEGFA*[10]. Despite the fact that *VEGFA* is itself directly targeted by 11 upregulated miRNAs (including five of those targeting *ZNF24*), its level of expression is significantly increased (PC) in CEPI. These results are consistent with the hypothesis that *ZNF24*-mediated derepression is overriding the expected downregulatory effects of the upregulated miRNAs on VEGFA expression.

Although many of the genes falling within the NC category could be the result of partial transcriptional override, they might also simply be the result of no or slight miRNA regulatory effects. Since we cannot experimentally distinguish between these two possibilities, we will operationally only consider PC differences in expression as being inconsistent with the expected IC differences.

**Figure 2:**
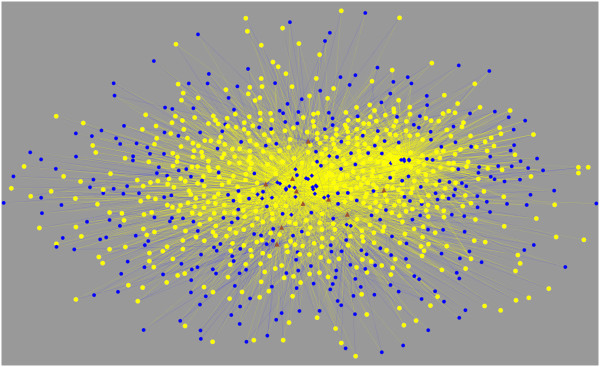
**Highly interconnected network of 31 microRNAs, repressors and their mutual targets.** Relationships among 31 miRNAs upregulated in cancer (blue diamonds), ten downregulated repressors that are targets of one or more of the upregulated miRNAs (brown triangles) and 843 genes that are the gene targets (blue and yellow circles) of both the upregulated miRNAs and the downregulated repressor genes. Yellow lines depict the regulatory connection between miRNAs and their target genes; blue lines depict the regulatory connection between repressors and target genes. From the perspective of the upregulated miRNAs, all target genes should be downregulated (all yellow circles); from the perspective of the downregulated repressors, all target genes should be derepressed/upregulated (all blue circles). According to TOM, the response of any particular target gene will be determined by the relative strengths of these opposing regulatory controls.

To further evaluate TOM, we identified 105 genes that are 1) targets of one-or more of the 31 upregulated miRNAs, 2) significantly downregulated in our cancer samples and 3) previously characterized as transcriptional repressors [14] (Additional file 3). The targets of ten (Table 3) of these 105 genes have been previously identified in the Transcription Factor Binding Site database (TRANSFAC) [15]. This resulted in 843 genes (Additional file 4) predicted to be directly targeted by both the ten downregulated repressors and one-or-more of the 31 upregulated miRNAs. From the perspective of miRNA regulation, all 843 of these target genes are expected to be downregulated while from the perspective of the downregulated repressor genes all the targets are expected to be upregulated. The observed reality lies somewhere between these two expectations (Figure 2). TOM predicts that the response of any particular target gene will be determined by the relative strengths of these two opposing regulatory controls.

**Table 3:** Ten genes characterized as validated repressors and predicted targets of one or more of the 31 miRNAs upregulated in CEPI

**Probeset_ID**	**Gene-symbol**	**P-value**	**Fold-change**
204194_at	BACH1	0.000564	-3.65
236796_at	BACH2	0.003707	-3.07
207186_s_at	BPTF	0.001731	-6.64
204314_s_at	CREB1	0.026886	-2.09
224891_at	FOXO3	0.001945	-2.23
210002_at	GATA6	0.000000	-56.60
212535_at	MEF2A	0.000016	-4.99
209239_at	NFKB1	0.001162	-1.66
209706_at	NKX3-1	0.000000	-20.57
224718_at	YY1	0.000758	-2.75

**Figure 3:**
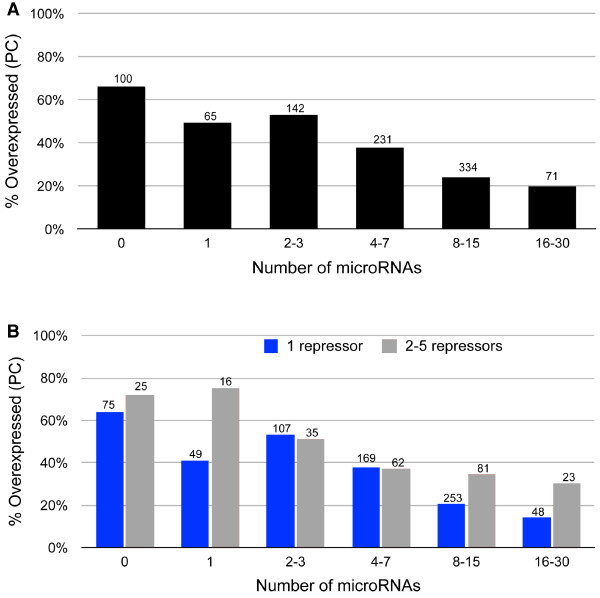
**Analysis of changes in expression of miRNA regulated repressor genes and their predicted target genes is consistent with TOM.** The 843 predicted target genes of 31 upregulated miRNAs and 10 downregulated repressors were divided into 6 groups based upon the number of upregulated miRNAs targeting each gene. (The number of genes included in each group is shown over the bars). **(A)** The strength of miRNA regulatory control on target genes increases with the number of miRNAs targeting individual genes. Bars represent the percentage of upregulated genes. The fact that ~20% of genes targeted by even large numbers (16–31) of upregulated miRNAs continue to display the unexpected PC is consistent with TOM. The chi-square test for trend is *X*^2^ = 67.34; p < 0.0001. **(B)** Genes targeted by 2–5 downregulated repressors (gray bars) override miRNA regulatory effects on coregulated target genes relative to genes targeted by one downregulated repressor (blue bars). The chi-square test for trend is *X*^2^ = 5.25, p < 0.0219.

The number of miRNAs targeting individual human genes (mRNAs) is known to vary from zero to over 100 with an estimated average of 7.1 miRNA targets per gene [16]. Thus, the relative strength of the regulatory effect of miRNAs on target genes might be expected to be a function of the number of miRNAs targeting individual genes. To explore this possibility, we grouped the 843 predicted gene targets of the 31 upregulated miRNAs and the 10 downregulated repressors into 5 groups based upon the number of upregulated miRNAs predicted to target each gene (Figure 3A). A sixth group was comprised of targets of the ten repressor genes (TRANSFAC) but not predicted targets of any of the 31 miRNAs. For each group, we computed the percentage of target genes that were upregulated (PC). In addition, for each group we divided the target genes into those predicted to be regulated by a single repressor vs. those predicted to be regulated by multiple repressors (Figure 3B).

The results demonstrate that as the number of upregulated miRNAs targeting individual genes increases, the percentage of target genes displaying the unexpected PC change in expression decreases. These results are consistent with TOM and indicate that as the relative strength of the miRNA regulatory effect increases, the impact of the opposing derepression effect mediated by the downregulated repressor genes is diminished. However, the fact that ~20% of genes targeted by even large numbers (>15) of upregulated miRNAs continue to display the unexpected PC indicates that, in some cases, the magnitude of derepression is sufficient to completely override miRNA regulation. The results presented in Figure 3B suggest that genes targeted by multiple repressors tend to be associated with a higher percentage of PC genes than those targeted by a single repressor. The effect, however, is not as consistent as observed with increasing numbers of regulating miRNA likely due to the relatively low number of repressor genes in this dataset and the fact that not all repressor genes can be expected to exert the same magnitude of regulatory control.

**Figure 4:**
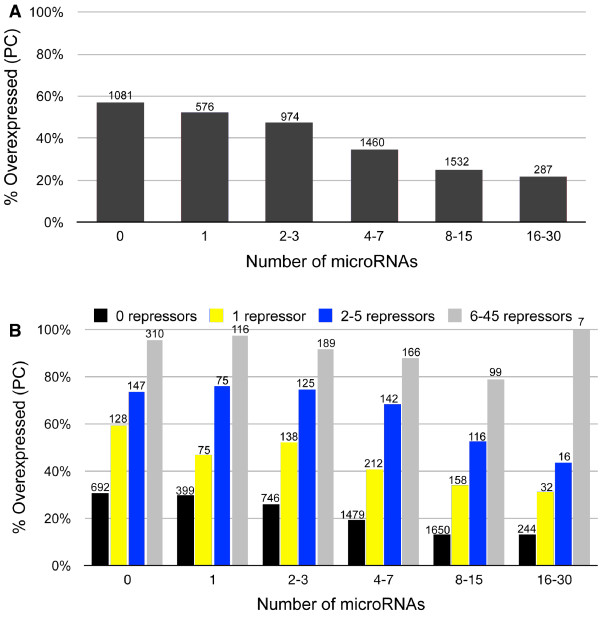
**Analysis of global changes in gene expression is consistent with TOM.** The 5910 differentially expressed genes targeted by upregulated miRNAs were divided into 6 groups based on the number of miRNAs targeting each gene (The number of genes included in each group are presented on top of the bars). **(A)** An inverse relationship exists between the number of upregulated miRNAs targeting genes and the percentage of these genes displaying the unexpected PC change in expression. The chi-square test for trend is *X*^2^ = 311.5, p < < 0.0001, indicating a significant increase in downregulated targets as the number of targeting microRNAs increases. **B)** The strength of miRNA regulatory control is diminished as the number of deregulated repressor genes increase. A chi-square test for trend for the miRNAs in this figure (4B) is *X*^2^ = 444.6, p < < 0.0001, indicating a significant increase in downregulated miRNA targets as the number of miRNAs increases. The chi-square test for trend for the transcriptional repressors in this figure (4B) is *X*^2^ = 1904.6, p < < 0.0001, indicating a significant increase in upregulated targets as the number of repressors increases.

We were next interested to see if the model’s ability to account for trends observed using the limited dataset described above might also extend more globally. We divided all differentially expressed genes including those that are predicted gene targets of the 31 upregulated miRNAs (4829) and those that are not (1081), into 6 groups based on the number of miRNAs targeting each gene. The results (Figure 4A) demonstrate a clear inverse relationship between the number of miRNAs targeting genes and the percentage of these genes displaying the unexpected PC. Again, however, we found that ~20% of genes targeted by even large numbers (>15) of upregulated miRNAs continue to display the unexpected PC consistent with the hypothesis that the magnitude of repressor gene mediated derepression is, in some instances, sufficient to completely override miRNA regulation.

Testing the model’s ability to globally predict the relative influence of miRNA and repressor gene regulatory controls on target gene expression is problematic for two reasons: first, a compendium of all human repressor genes and their regulatory targets is currently unavailable; second, many regulatory proteins can function as repressors or activators depending on cellular context and protein complex association [17]. One approach taken by systems biologists to model regulatory relationships in complex cellular contexts is to use highly correlated changes in expression patterns among genes as evidence of direct and/or indirect interactions [18,19]. In our case, we examined variation in gene expression patterns across our OSE samples to identify genes displaying consistent inverse correlations (Pearson correlation coefficient < -0.8) in expression with changes in expression of the 105 repressor genes previously characterized as significantly downregulated in CEPI and regulatory targets of one-or-more of the 31 miRNAs (see above). Genes displaying an inversely correlated pattern of co-expression (1205) were operationally classified as targets of these repressor genes. Genes not displaying this pattern of expression (3624) were classified as non-targets of the designated repressor genes. Having established these classes, it became possible to distinguish between regulatory interactions fulfilling the triangular relationship of TOM from those that do not.

**Figure 5:**
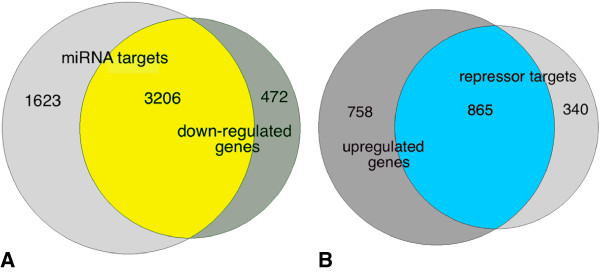
**Upregulated repressor targets and downregulated miRNA targets show significant enrichment consistent with TOM.** Venn diagrams display the enrichment of **A)** downregulated targets of miRNAs. The results are consistent with TOM (hypergeometric distribution, p < 1E-12: population = 5910, downregulated genes = 3678, number of predicted miRNA targets = 4829, downregulated miRNA regulated targets = 3206; **B)** upregulated targets of repressors that are also predicted targets of 1 or more of the 31 upregulated miRNAs. The results are consistent with TOM (hypergeometric distribution, p <1E-12: population = 4829, total upregulated genes = 1623, number of repressor targets = 1205, upregulated repressor targets = 865).

The results presented in Figure 4B again indicate that as the number of upregulated miRNAs targeting genes increases, the percentage of the unexpected PC decreases significantly (chi-square test for trend *X*^2^ = 444.6, p < <0.0001). Consistent with the results presented in Figure 4, the overlap of miRNA targets and downregulated genes is highly significant. (Figure 5A; hypergeometric p < 1E-12). Likewise, the overlap of repressor targets and upregulated genes is also highly significant (Figure 5B; hypergeometric p < 1E-12). Collectively, the results indicate that the derepression of target genes mediated by high (6–45) numbers of downregulated repressors is sufficient to nearly or completely override the regulatory controls of even large numbers (>15) of upregulated miRNAs.

## Discussion

Recent studies have clearly established miRNAs as early indicators of disease [20,21] and as a potential new class of therapeutic agents [22,23]. Full appreciation of the biological significance of modulations in levels of miRNAs, as well as, the future rational employment of miRNAs as therapeutic agents will require an understanding of both the direct and indirect molecular consequences of changes in the levels of miRNAs on cell function. While the direct gene (mRNA) targets of individual miRNAs can be computationally predicted and experimentally validated with varying degrees of accuracy [24], reliable predictions of the indirect molecular effects of changes in miRNA levels has remained a major challenge in molecular systems biology [23,25].

In this paper, we present a regulatory network model (TOM) that explains a significant component of the unexpected low frequency of IC changes in expression levels between mRNAs and their regulating miRNAs. The model postulates that the expected downregulation of target genes induced by elevated levels of regulating miRNAs may be masked or “overridden” by increases in transcriptional initiation mediated by the downregulation of repressor genes that are themselves targets of the same regulating miRNAs (Figure 1A). Depending upon the strength of the transcriptional override (i.e., the relative strengths of miRNA and repressor gene mediated de-repression), TOM predicts that increases in miRNA levels may display no effect (NC) or be positively correlated (PC) with changes in levels of their targeted mRNAs.

It is widely recognized that the operation of regulatory effects mediated by miRNAs in vivo is a complex and interactive process and a number of explanatory models have been previously offered [26-29]. In this paper, we propose an additional model (TOM) that focuses on the feedback interactions that exist between miRNAs, regulatory (repressor) genes and their mutual gene targets. While we have evaluated TOM within the context of its ability to account for global patterns of changes in gene expression, the model also provides a framework for predicting interactions between specific miRNAs and target genes (e.g., Figure 1). Further testing in other cancers (and other biological contexts) will be needed to evaluate the robustness of TOM. Nevertheless, our initial findings in ovarian cancer indicate that interactions between miRNAs and repressor genes may well play a significant role in effecting the unexpected regulatory responses of targeted genes to modulations in levels of their regulatory miRNAs.

## Conclusions

It is now widely acknowledged that the complexity of molecular interactions taking place on the cellular level can significantly obscure the expected consequences of molecular processes characterized in vitro [30,31]. Our findings indicate that the direct and indirect regulatory effects of changes in miRNA expression levels in vivo are interactive and complex but amenable to systems level modeling. We have shown that TOM can account for a major component of the unexpected consequences of changes in miRNA expression levels on their target mRNAs. Although the model has been developed and evaluated within the context of ovarian cancer, we believe it may be applicable in other biological contexts as well including of potential future use in the rational design of miRNA-based strategies for the treatment of cancer and other diseases.

## Methods

All tissues were collected according to previously published procedures [32] following approved Institutional Review Board protocols from Northside Hospital (Atlanta) and Georgia Institute of Technology. Informed consent was obtained from all subjects. The histopathology for all cancer patients was serous papillary adenocarcinoma of the ovary and for the control patients the ovaries were considered within normal limits.

### mRNA microarray data analysis

Ten OSE (normal) and ten CEPI (cancer) samples were analyzed for mRNA expression using the Affymetrix Gene Chip Operating System (GCOS HG-U133 Plus 2.0). CEL files generated by GCOS were converted to expression values using GCRMA normalization on the arrayanalysis.org [33] website, which output also included quality control metrics, principal components analysis (PCA) and cluster dendrograms. Present/absent calls were generated from the MAS 5.0 statistical algorithm as implemented in Affymetrix Expression Console. Probe sets with >60% present calls in either of the two groups (OSE and CEPI) were selected for further analysis. After log2 transformation, signal values of those probe sets were submitted to Statistical Analysis of Microarrays (SAM) for multiple testing correction where a 5.5% FDR was applied resulting in 7462 probe sets representing 5910 differentially expressed genes (DEGs). Annotations for probe sets were obtained from Affymetrix [34]. The processed and raw data files for the samples used in this study have been deposited in the Gene Expression Omnibus (GSE52037 with SuperSeries GSE52460).

### microRNA microarray data analysis

Expression profiles for microRNAs from three OSE and three CEPI samples were generated by Asuragen (Austin, TX) using Ambion miRChip technology (Life Technologies). Two sets of CEL files, created from 6 biological replicates and two sets of technical replicates were normalized using MAS 5.0 to expression signals, giving 6 values per probe/gene. Probe sets labeled as human (those having an “hsa-” prefix), known to be conserved to mouse, and with at least 65% present calls (calculated by Asuragen) in either of the two groups (OSE and CEPI) were selected for further filtering. Thirty-one differentially expressed microRNAs (fc > 6, p-value < .03) were selected. The repressive potential of all 31 microRNAs was validated by noting that > 65% of the predicted DEG targets of each upregulated microRNA were actually downregulated, while only 44% of DEGs *not* predicted to be targets of any upregulated microRNA were downregulated. Mean repression over all 31 microRNAs was 71%. The processed and raw data files for the samples used in this study have been deposited in the Gene Expression Omnibus (GSE52459 with SuperSeries GSE52460).

### microRNA target prediction

The miRNA target prediction file based on mirSVR was downloaded from microRNA.org (August 2010 release). The mirSVR score refers to targets of microRNAs with scores obtained from their support vector regression algorithm. To reduce the occurrence of false positives, only predicted targets with a mirSVR score less than -.2 were considered. The microRNA target predictions based on TargetScan and SVMicrO were downloaded from http://www.targetscan.org (retrieved 8/2010) and http://www.compgenomics.utsa.edu/Result/Human/hsa_human (retrieved 9/2010), respectively.

### Transcriptional repressor selection

Members of the Gene Ontology categories GO:0045892, GO:0000122, GO:0010944, GO:0032088 and GO:0008156, relating to the negative-regulation-of-transcription or its child terms, were downloaded from the European Bioinformatics Institute (EBI) and parsed using UNIX scripts. In that download, we found 439 potential repressor genes. Of those, 109 genes were significantly downregulated according to our microarray analysis and 105 of these genes were also predicted targets of one or more of the 31 upregulated microRNAs. These 105 transcriptional repressor genes formed the basis for microRNA target derepression in our model.

### Transcriptional repressor target prediction and experimental validation

To obtain predicted and/or experimentally validated transcription factor binding site data, we downloaded the TRANSFAC data file **c3.tft.v3.1.symbols.gmt** from GSEA (Gene Set Enrichment Analysis website - http://www.broadinstitute.org/gsea/downloads.jsp). Data files were parsed with UNIX scripts, which extracted pairs of genes consisting of one repressor and one or more binding partners. All repressor-partner pairs under consideration had to be DEGs and predicted targets of at least one of the 31 upregulated microRNAs, and all transcriptional repressors were downregulated in cancer. Further, all repressor-partner pairs were required to show a correlation coefficient of r < -.8 across all normal samples.

### Correlation coefficient calculation

For the global analysis of relationships among all 105 transcriptional repressors and their binding partners, Pearson’s correlation coefficient (PCC) was calculated across all ten OSE (normal) samples between all transcriptional repressors and predicted microRNA targets. Specifically, we used the Mathematica [35] correlation function (n = 10; r < -.8) for a directional significance of (p < .0027). Fold-change from normal to cancer in these genes ranged from -625 to 121.

### Availability of supporting data

The processed and raw data files for the samples used in the mRNA and miRNA expression studies have been deposited in the Gene Expression Omnibus (GSE52037 and GSE52459 with SuperSeries GSE52460).

## Abbreviations

CEPI: Cancer epithelial cells; IC: Inversely correlated; mRNA: Messenger RNA; miRNA: MicroRNA; NC: No change; OSE: Ovarian surface epithelial cells; PC: Positively correlated.

## Competing interests

The authors declare that they have no competing interests.

## Authors’ contributions

CGH & JFM conceived the project and wrote the paper; LVM conducted microarray analyses; CGH performed the computational data analyses; LDW coordinated collection of patient history data, conducted laser capture microdissections and edited the manuscript; BBB collected patient samples. All authors read and approved the final manuscript.

## Supplementary Material

Additional file 1 **Patient samples analyzed in this study.** All tissues were collected according to previously published procedures [32], following approved Institutional Review Board protocols from Northside Hospital (Atlanta) and Georgia Institute of Technology. Click here for file

Additional file 2 **Gene expression profiling identified 5910 significantly differentially expressed genes (mRNAs) between OSE and CEPI.** Of these, 2232 (38%) were significantly upregulated and 3678 (62%) significantly downregulated in CEPI. Click here for file

Additional file 3 **List of genes that are 1) targets of one-or more of the 31 upregulated miRNAs, 2) downregulated in our cancer samples and 3) previously characterized as transcriptional repressors [**[14]**].** Genes belonging to gene ontology categories GO:0045892, GO:0000122, GO:0010944, GO:0032088 and GO:0008156, relating to the negative-regulation-of-transcription, were downloaded from the European Bioinformatics Institute. Downregulated repressor genes that were also predicted microRNA targets formed the 105 transcriptional repressor genes presented in this list. Click here for file

Additional file 4 **List of TRANSFAC [**[15]**]-identified regulatory targets of 10 repressor genes (see Table **3**) that are also the targets of one-or-more of 31 miRNAs upregulated in CEPI (843 genes).** Click here for file
