# Supplementary material for: Transcriptional override: a regulatory network model of indirect responses to modulations in microRNA expression
Source: BMC Syst Biol. 2014 Mar 25;8:36. doi: 10.1186/1752-0509-8-36 (PMC3987680; doi:10.1186/1752-0509-8-36)
Supplement: Additional file 1 — Patient samples analyzed in this study. All tissues were collected according to previously published procedures [32], following approved Institutional Review Board protocols from Northside Hospital (Atlanta) and Georgia Institute of Technology. [file 1752-0509-8-36-S1.pdf]

ID #	Age at time of surgery	Stage	Grade
183	66	III	2
336	63	Ic	3
369	52	IIIc	2
489	48	IV	3
528	66	IIIc	3
537	64	IIIa	2/3
542	61	IV	3
551	59	IIIc/IV	3
588	71	IIIc	2/3
606	54	IIIa	3
620	62	III/IV	3
434	41	N/A	N/A
437	54	N/A	N/A
440	50	N/A	N/A
452	51	N/A	N/A
470	44	N/A	N/A
475	63	N/A	N/A
482	44	N/A	N/A
567	77	N/A	N/A
665	84	N/A	N/A
541	41	N/A	N/A
552	41	N/A	N/A
563	66	N/A	N/A
783	52	N/A	N/A
