# Supplementary material for: Transcriptional override: a regulatory network model of indirect responses to modulations in microRNA expression
Source: BMC Syst Biol. 2014 Mar 25;8:36. doi: 10.1186/1752-0509-8-36 (PMC3987680; doi:10.1186/1752-0509-8-36)
Supplement: Additional file 2 — Gene expression profiling identified 5910 significantly differentially expressed genes (mRNAs) between OSE and CEPI. Of these, 2232 (38%) were significantly upregulated and 3678 (62%) significantly downregulated in CEPI. [file 1752-0509-8-36-S2.pdf]

Additional file 2

Probeset_ID	Gene-Symbol	P-value	Fold-change
1007_s_at	DDR1	0.0549881	1.22887
121_at	PAX8	0	15.0578
1405_i_at	CCL5	0.0530068	-3.29232
1552283_s_at	ZDHHC11	0.0350079	1.29007
1552291_at	PIGX	0.0000248	-2.30082
1552310_at	C15orf40	0.0009282	-4.12538
1552330_at	CENPBD1	0.0011106	-2.81287
1552347_at	CRYZL1	0.0009221	-2.10401
1552360_a_at	TIRAP	0.0000151	-1.78123
1552365_at	SCIN	0.0017148	-2.23779
1552368_at	CTCFL	0.0230758	3.51595
1552390_a_at	C8orf47	0.0000224	-4.93011
1552400_a_at	C15orf27	0.0029674	-1.76271
1552410_at	CLEC4F	0.0188336	1.19002
1552426_a_at	TM2D3	0.0102293	-2.14789
1552502_s_at	RHBDL2	0.0002123	-2.34471
1552579_a_at	ADAM21	0.0094439	1.23792
1552611_a_at	JAK1	0.0313456	-2.19323
1552628_a_at	HERPUD2	0.0031113	-1.79576
1552633_at	ZNF101	0.0616072	1.21299
1552644_a_at	PHC3	0.0598531	1.25595
1552670_a_at	PPP1R3B	0.0012892	1.20766
1552701_a_at	CARD16	0.0002669	-10.6179
1552703_s_at	CARD16///CAS	0.0000002	-11.7943
1552727_s_at	ADAMTS17	0.0006301	-2.18071
1552729_at	SNHG7	0.0086515	1.29152
1552733_at	KLHDC1	0.0000075	-4.62514
1552797_s_at	PROM2	0.0000369	12.7273
1552842_at	HS6ST3	0.039747	1.5041
1552845_at	CLDN15	0.0000394	-5.62293
1552889_a_at	EXOC3L2	0.0150072	1.29696
1552895_a_at	C21orf99	0.0208122	1.15625
1552910_at	SIGLEC11	0.0018767	-4.05868
1552939_at	ANGPT1	0.0071604	1.143
1552946_at	ZNF114	0.0589597	1.3533
1553099_at	TIGD1	0.0593985	2.08452
1553101_a_at	ALKBH5	0.0444306	1.26771

1553103_at	NFX1	0.0092265	-2.46141
1553153_at	ATP6V0D2	0.0114094	1.318
1553169_at	LRRN4	0	-17.2772
1553172_at	ZNF777	0.0130754	2.02375
1553217_s_at	ZNF41	0.06359	1.14512
1553228_at	CCDC89	0.003274	-2.32769
1553234_at	ADAMTS18	0.0084896	1.19
1553244_at	FANCB	0.0879093	1.2817
1553292_s_at	SGK494	0.0145363	1.45563
1553322_s_at	TEAD1	0.0114548	1.66964
1553329_at	C7orf45	0.0088679	1.18971
1553363_at	C6orf195	0.0440268	1.21789
1553386_at	MFSD2A	0.0470187	1.4502
1553508_at	MDS2	0.0252933	1.5319
1553514_a_at	VNN3	0.0840234	1.2465
1553549_at	VN1R2	0.0292821	1.15269
1553562_at	CD8B///CD8BP	0.0576917	1.37275
1553582_a_at	SPAG11A///SPA	0.0303636	2.63183
1553613_s_at	FOXC1	0.0312648	-2.51337
1553644_at	C14orf49	0.0110777	1.15191
1553677_a_at	TIPRL	0.038681	1.43424
1553690_at	SGOL1	0.0067566	1.4385
1553703_at	ZNF791	0.0000732	-3.93011
1553709_a_at	PRPF38A	0.0158537	1.58125
1553780_at	MGC23270	0.0070432	1.17639
1553878_at	GOT1L1	0.0107202	1.33231
1553906_s_at	FGD2	0.0418794	2.84087
1553954_at	ALG14	0.0138544	-2.9036
1553961_s_at	SNX21	0.0027176	-1.69151
1553972_a_at	CBS	0.0000063	4.75127
1553984_s_at	DTYMK	0.0000689	2.18958
1553986_at	RASEF	0.0074188	-3.2283
1553998_at	DMRTC1///DMI	0.0007578	-4.09968
1554008_at	OSMR	0.0043015	-2.52762
1554018_at	GPNMB	0.0037958	-4.56492
1554020_at	BICD1	0.0015644	2.87156
1554045_at	ZNF24	0.0127662	1.28158
1554050_at	SMPDL3B	0.0006271	1.35017
1554063_at	C8orf76	0.0336054	2.17886
1554101_a_at	TMTC4	0.0025788	1.49198

1554114_s_at	SSH2	0.037096	1.24452
1554119_at	C16orf57	0.0455046	1.53875
1554175_at	CD300LB	0.0881861	1.38483
1554182_at	TRIM73///TRIM	0.0242634	-1.9642
1554248_at	ZNF638	0.0000758	-2.05323
1554274_a_at	SSH1	0.0177698	1.22231
1554341_a_at	HELQ	0.000031	-2.02783
1554345_a_at	GIN1	0.0003305	-2.25476
1554352_s_at	DENND4A	0.0002966	-1.74455
1554356_at	GINS4	0.0069573	1.41197
1554429_a_at	DMWD	0.0172472	1.26091
1554462_a_at	DNAJB9	0.0109707	-2.06923
1554485_s_at	TMEM37	0.0000059	-4.35293
1554486_a_at	C6orf114	0.0750429	2.31368
1554488_at	CEP70	0.0257763	1.24315
1554557_at	ATP11B	0.0378003	1.24858
1554608_at	TGOLN2	0.0723755	1.4107
1554707_at	C9orf68	0.0540154	1.68519
1554741_s_at	FGF7///KGFLP1	0.0000114	-6.9427
1554789_a_at	PDE8B	0.0005297	-2.55016
1554800_at	RAB39	0.0456125	1.22396
1554804_a_at	CLDN19	0.0012872	1.81567
1554860_at	PTPN7	0.0248582	1.32164
1554869_at	WDR37	0.0002478	1.44302
1554890_a_at	TIA1	0.0033883	1.64662
1554894_a_at	PCBD2	0.0013905	1.97798
1554986_a_at	SNX19	0.0156042	1.23141
1554987_at	GOLGA3	0.0169631	1.17922
1554989_at	KIAA0317	0.0433536	1.47987
1555004_a_at	RBL1	0.0017818	1.23907
1555007_s_at	WDR66	0.0853608	1.18497
1555009_a_at	SYNJ2	0.0516107	1.30122
1555015_a_at	ZNF398	0.0671194	1.18309
1555021_a_at	SCARF1	0.0001099	1.52054
1555099_at	MPP4	0.081453	1.19238
1555100_at	APLF	0.0031489	1.48711
1555168_a_at	CALN1	0.0138002	1.58281
1555225_at	C1orf43	0.0278456	1.69531
1555248_a_at	WNK3	0.0089463	1.29321
1555274_a_at	EPT1	0.0011946	3.13561

1555279_at	ARMC8	0.0361462	1.58872
1555288_s_at	FBF1	0.0106723	1.45451
1555348_at	TFAP2E	0.0384635	1.26296
1555399_a_at	DUSP16	0.0439195	1.54037
1555495_a_at	CWC27	0.0195236	-2.63685
1555529_at	RNH1	0.0458033	1.2041
1555542_at	AFAP1L1	0.0633519	1.39526
1555564_a_at	CFI	0.0005317	-1.97975
1555579_s_at	PTPRM	0.0000484	4.22742
1555612_s_at	G6PC	0.0203328	1.69012
1555634_a_at	LILRA5	0.0173521	1.52799
1555746_at	CD79B	0.086855	1.8416
1555760_a_at	RBM15	0.0060541	2.4239
1555779_a_at	CD79A	0.0038814	1.47479
1555780_a_at	RHEB	0.0108533	-1.94198
1555790_a_at	TMEM192///ZN	0.006202	-3.90074
1555809_at	CRISPLD2	0.0213794	1.69715
1555820_a_at	MKS1	0.0034072	1.25593
1555824_a_at	PACS2	0.0000928	2.26054
1555841_at	C9orf30	0.0001963	-2.85457
1555842_at	CYTH2	0.0015041	1.86708
1555843_at	HNRNPM	0.0630714	1.14182
1555862_s_at	MICALL2	0.000212	2.29274
1555870_at	RNF207	0.0267846	1.56608
1555878_at	RPS24	0.0256804	3.01994
1555883_s_at	SPIN3	0.0167246	1.33723
1555886_at	PDSS2	0.0001344	-3.08477
1555897_at	KDM1A	0.0042686	1.23245
1555908_at	FAM120A	0.0819759	1.22342
1555910_at	PTCD2	0.0016293	-2.39595
1555923_a_at	C10orf114	0.0105925	1.496
1555933_at	KIAA2013	0.0805401	1.30925
1556033_at	FLJ39739	0.001612	1.62904
1556039_s_at	GPR173	0.0025879	1.52806
1556047_s_at	MAGEE1	0.0342796	-2.45666
1556121_at	NAP1L1	0.00142	-1.87927
1556167_at	HEATR7B1	0.0034224	1.33825
1556183_at	FLJ40330	0.0036056	1.42636
1556201_at	RNASET2	0.0052415	1.22107
1556203_a_at	SRGAP2	0.0343167	-2.06372

1556213_a_at	BTG3	0.0019552	1.33698
1556269_at	MYT1	0.0020707	1.53256
1556277_a_at	PAPD4	0.0332494	-2.42213
1556426_at	HEXA	0.045453	1.2718
1556427_s_at	LRRN4CL	0.0003127	-3.61509
1556499_s_at	COL1A1	0.0000582	2.58066
1556533_at	C17orf52	0.0042109	1.47388
1556876_s_at	TPTE2P2	0	-1.875
1556950_s_at	SERPINB6	0.0114493	-2.0287
1557014_a_at	C9orf122	0.0506897	1.32383
1557122_s_at	GABRB2	0.0056141	-2.12803
1557132_at	WDR17	0.0002629	-2.86134
1557143_at	CSMD2	0.03674	1.9871
1557322_at	ZNF230	0.0003526	-2.40817
1557411_s_at	SLC25A43	0.0220516	-2.24094
1557458_s_at	SHB	0.0064625	-2.18516
1557553_at	PPP1R12B	0.0037687	-1.86779
1557613_at	FLJ39534	0.0749055	1.47493
1557674_s_at	EFCAB2	0.0435443	1.29691
1557866_at	C9orf117	0.0817303	2.92046
1557953_at	ZKSCAN1	0.0006868	-1.70742
1558034_s_at	CP	0.0028481	12.0512
1558117_s_at	USP31	0.0087214	-1.89381
1558143_a_at	BCL2L11	0.0618087	1.75077
1558199_at	FN1	0.0744504	1.22828
1558290_a_at	PVT1	0.0004242	1.63314
1558292_s_at	PIGW	0.0002164	2.22426
1558323_at	TMEM72	0.0954277	1.31408
1558375_at	LRRC38	0.0913853	1.29802
1558407_at	PLEKHG2	0.0014291	1.41682
1558467_a_at	UGGT2	0.0093441	-1.98099
1558483_at	LRRC27	0.0300573	1.25156
1558487_a_at	TMED4	0.0019168	-2.52957
1558511_s_at	ESYT2	0.0636778	1.9156
1558515_at	NCRNA00182	0.0768249	1.16096
1558586_at	ZNF33B	0.0020247	-2.4338
1558641_at	SPATA24	0.0010102	1.40663
1558706_a_at	ATOH8	0.0845831	1.45722
1558747_at	SMCHD1	0.0418149	-2.10585
1559064_at	NUP153	0.0000463	1.52788

1559093_at	FLJ30403	0.0413123	1.24594
1559140_at	FAM87A	0.0237261	1.28776
1559419_at	CACNB2	0.0002579	-3.62947
1559471_s_at	D21S2088E	0.0925011	1.16656
1559490_at	LRCH3	0.0050552	-2.21088
1559561_at	FBXO18	0.0018182	1.46328
1559624_at	STK32A	0.0155404	1.4964
1559645_at	NCRNA00184	0.0730322	2.04937
1559901_s_at	C21orf34	0.0181337	-1.93481
1559946_s_at	RUVBL2	0.0005248	2.34941
1560225_at	CNR1	0.0006636	-1.73116
1560275_at	TMEM44	0.0942988	1.59449
1560359_at	PELO	0.0095505	-1.8988
1560507_at	AWAT1	0.0138994	1.54955
1560814_a_at	C15orf57	0.0011024	-2.44296
1560821_at	ARHGAP22	0.0209824	1.16674
1560854_s_at	ZNF107	0.0012809	1.4608
1560874_at	EFCAB4B	0.0989604	1.48192
1560879_a_at	SYT15	0.0690501	1.24725
1560897_a_at	KRTAP10-11	0.0102675	1.41023
1561042_at	ITGB1	0.0188618	1.48422
1561578_s_at	MCART6	0.0090765	1.48545
1561651_s_at	TAL1	0.0006895	1.32286
1561882_at	SYTL3	0.0060412	1.25479
1561976_at	C1orf167	0.0053151	1.44018
1562736_at	LHX9	0	-152.096
1562815_at	EFCAB6	0.0006416	-1.69646
1563090_at	CCDC33	0.0167189	1.34605
1563315_s_at	ERICH1	0.0044932	-1.85042
1563533_at	GADL1	0.0000001	-29.9076
1563638_at	FAM18A	0.0000051	-4.70357
1563646_a_at	TMEM67	0.0214911	2.03955
1563687_a_at	FRYL	0.0050157	1.63456
1563728_at	NCRNA00032	0.0240591	1.27766
1563845_at	FAM153B	0.0000331	-4.87538
1563969_at	FLJ33360	0.0269926	1.20055
1564207_at	FLJ35390	0.0000316	-3.14188
1564211_at	C14orf64	0.047952	1.52778
1564276_at	C5orf56	0.0173922	1.28237
1564308_a_at	MPP7	0.0004207	1.62118

1565027_at	OFCC1	0.001972	1.2382
1565454_at	XAGE-4	0.0452814	1.74525
1565641_at	C16orf45	0.0114524	1.66104
1565681_s_at	DIP2C	0.0038405	1.2608
1565817_at	IKZF1	0.0956601	1.56393
1565898_at	METT5D1	0.0246889	-2.19881
1565905_at	FLJ46026	0.0430714	1.39339
1566101_at	TTLL5	0.0867802	1.3724
1566603_s_at	RPUSD3	0.0709515	1.47053
1566766_a_at	MACC1	0.0030949	2.50696
1566851_at	TRIM42	0.0009662	1.35825
1568592_at	TRIM69	0.0030841	-2.37621
1568613_at	RSPH3	0.0800966	1.22531
1568634_a_at	LRRC66	0.0254341	1.23225
1568663_a_at	PWRN2	0.0704895	1.30356
1568678_s_at	FGFR1OP	0.000591	2.173
1568834_s_at	CCDC90B	0.0012531	-3.27028
1568857_a_at	NBR1	0.0025654	-2.43904
1568868_at	CYP27C1	0.0021996	1.52195
1568951_at	ZNF280D	0.0033572	-1.90937
1569030_s_at	NUB1	0.0055588	1.9174
1569396_at	RAB40C	0.0113175	1.76747
1569448_at	PGM2L1	0.0002668	1.55191
1569557_at	ZNF248	0.014711	1.18306
1569594_a_at	SDCCAG1	0.0000773	-3.86659
1569607_s_at	ANKRD20A1///	0.0049436	-5.35476
1569637_at	ZNF736	0.0401933	1.27926
1569652_at	MLLT3	0.0189736	-2.02319
1569679_at	CDH22	0.0337644	1.22727
1569683_at	XYLB	0.0905668	1.18804
1569690_at	CCDC36	0.0031691	1.27961
1569827_at	ATG7	0.0807073	1.27616
1569990_at	NUDT3	0.0166389	1.45375
1569998_at	MMD2	0.000684	1.21864
1570035_at	TBC1D10A	0.0271124	1.96446
1570078_a_at	DOCK5	0.0004011	-1.95835
1570315_at	HTA	0.0000586	1.42369
1570447_at	FLJ44054	0.0033067	1.18796
1570470_at	CATSPERB	0.0322505	1.12725
200004_at	EIF4G2	0.0102019	-1.84101

200005_at	EIF3D	0.0000001	-5.625
200007_at	SRP14	0.0000933	-2.20058
200010_at	RPL11	0.0009532	-2.00922
200014_s_at	HNRNPC	0.0064343	-1.8435
200021_at	CFL1	0.000274	2.45894
200022_at	RPL18	0.0004157	-2.38952
200023_s_at	EIF3F	0.0000138	-2.93254
200026_at	RPL34	0.0006709	-1.76867
200029_at	RPL19	0.0004752	-2.33245
200030_s_at	SLC25A3	0.0023776	-3.31366
200032_s_at	RPL9	0.0005998	-1.63469
200033_at	DDX5	0.0093636	-3.69463
200034_s_at	RPL6	0.0002128	-2.76048
200038_s_at	RPL17	0.0002982	-2.2948
200042_at	C22orf28	0.0000007	-2.80888
200046_at	DAD1	0.0163689	-2.04161
200051_at	SART1	0.0582898	1.44559
200054_at	ZNF259	0.0165244	1.3838
200055_at	TAF10	0.0447907	1.56865
200056_s_at	C1D	0.0249868	-2.29784
200059_s_at	RHOA	0.0075581	-3.37855
200066_at	IK	0.0000021	-3.00938
200068_s_at	CANX	0.0925141	1.27341
200070_at	C2orf24	0.0018607	1.67633
200074_s_at	RPL14	0.0036009	-2.81914
200079_s_at	KARS	0.0000039	-3.08576
200085_s_at	TCEB2	0.0756917	1.80054
200086_s_at	COX4I1	0.00036	-1.79987
200089_s_at	RPL4	0.0000003	-2.00842
200097_s_at	HNRNPK	0.0051514	-2.5261
200597_at	EIF3A	0.0038789	-2.19148
200598_s_at	HSP90B1	0.0103317	-5.15586
200602_at	APP	0.0313875	1.62479
200608_s_at	RAD21	0.0233044	-2.11138
200611_s_at	WDR1	0.0197017	1.24891
200614_at	CLTC	0.0000945	-2.92696
200617_at	MLEC	0.0254954	1.6607
200620_at	TMEM59	0.0001205	-3.12724
200626_s_at	MATR3	0.0017625	-3.36991
200627_at	PTGES3	0.013806	-2.06943

200631_s_at	SET	0.0038832	-3.40964
200633_at	UBB	0.0023001	-2.96639
200636_s_at	PTPRF	0.0024099	-2.59812
200645_at	GABARAP	0.0090731	-1.89145
200651_at	GNB2L1	0.0027311	-2.81201
200656_s_at	P4HB	0.0502847	1.24179
200660_at	S100A11	0.0127223	1.97341
200662_s_at	TOMM20	0.0087716	-2.23213
200663_at	CD63	0.0035683	-2.1512
200673_at	LAPTM4A	0.0035576	-1.86584
200677_at	PTTG1IP	0.0001943	-2.43137
200681_at	GLO1	0.0018518	-2.17451
200685_at	SFRS11	0.0006437	-5.38411
200697_at	HK1	0.0608819	1.30817
200701_at	NPC2	0.0039914	-2.31244
200704_at	LITAF	0.0111894	-2.64605
200705_s_at	EEF1B2	0.0000917	-2.74332
200710_at	ACADVL	0.0521686	1.48681
200719_at	SKP1	0.0010796	-1.84112
200721_s_at	ACTR1A	0.045596	1.31222
200723_s_at	CAPRIN1	0.0093146	-2.1856
200726_at	PPP1CC	0.0037945	-2.11529
200728_at	ACTR2	0.0052776	-3.12788
200732_s_at	PTP4A1	0.0000036	-6.84592
200734_s_at	ARF3	0.0008939	3.38332
200743_s_at	TPP1	0.0000001	-3.84836
200745_s_at	GNB1	0.055637	-2.60903
200747_s_at	NUMA1	0.0229836	1.67196
200750_s_at	RAN	0.0623156	-2.52925
200755_s_at	CALU	0.0110825	1.68295
200758_s_at	NFE2L1	0.0001762	-2.07053
200761_s_at	ARL6IP5	0.0028228	-2.32917
200762_at	DPYSL2	0.0000177	-3.4058
200768_s_at	MAT2A	0.0002679	1.56855
200770_s_at	LAMC1	0.0009695	2.721
200777_s_at	BZW1	0.0001008	-3.31701
200778_s_at	Sep 2, 2013	0.0006643	2.23783
200782_at	ANXA5	0.0000037	-6.13175
200783_s_at	STMN1	0.0533403	1.63939
200786_at	PSMB7	0.006038	-3.27129

200788_s_at	PEA15	0.0006107	2.23247
200790_at	ODC1	0.0021476	-4.04763
200792_at	XRCC6	0.0014636	-2.23923
200815_s_at	PAFAH1B1	0.0019771	2.02504
200818_at	ATP5O	0.0025051	-2.50897
200824_at	GSTP1	0.0000009	2.87514
200827_at	PLOD1	0.0053661	1.58148
200830_at	PSMD2	0.0162141	1.66072
200832_s_at	SCD	0.0126909	3.57735
200833_s_at	RAP1B	0.0012906	-3.26231
200843_s_at	EPRS	0.0010506	-2.50778
200847_s_at	TMEM66	0.000276	-3.33556
200850_s_at	AHCYL1	0.0022797	-3.38272
200851_s_at	KIAA0174	0.0000231	-2.38624
200853_at	H2AFZ	0.0406684	-2.21174
200854_at	NCOR1	0.0023295	-1.77261
200860_s_at	CNOT1	0.0000323	-3.42982
200862_at	DHCR24	0.0407004	2.00109
200864_s_at	RAB11A	0.0161591	-2.10279
200867_at	RNF114	0.0045811	-2.788
200872_at	S100A10	0.0000005	-5.46549
200876_s_at	PSMB1	0.0003315	-4.37884
200877_at	CCT4	0.0003455	-4.62336
200878_at	EPAS1	0.0025148	-2.33243
200880_at	DNAJA1	0.02334	-2.29976
200882_s_at	PSMD4	0.026104	1.38807
200884_at	CKB	0.0001452	2.08788
200895_s_at	FKBP4	0.0000176	2.75253
200899_s_at	MGEA5	0.0000463	-2.3745
200902_at	Sep 15, 2013	0.0027127	-4.07728
200904_at	HLA-E	0.0002714	-3.46689
200912_s_at	EIF4A2	0.0039001	-1.81591
200916_at	TAGLN2	0.0000874	2.50486
200919_at	PHC2	0.0209175	1.43167
200920_s_at	BTG1	0.0001036	-6.56378
200925_at	COX6A1	0.0003783	2.47511
200927_s_at	RAB14	0.0055434	-2.09159
200940_s_at	RERE	0.0329634	-2.29127
200941_at	HSBP1	0.0000006	-2.51917
200943_at	HMGN1	0.0147192	-2.20079

200948_at	MLF2	0.0032288	1.90884
200950_at	ARPC1A	0.0058286	-2.22336
200952_s_at	CCND2	0.046266	1.14161
200958_s_at	SDCBP	0.0052795	-4.1571
200962_at	RPL31	0.0241529	-2.64667
200965_s_at	ABLIM1	0.0005983	-2.18964
200970_s_at	SERP1	0.0018371	-2.21337
200973_s_at	TSPAN3	0.0039487	-1.89592
200986_at	SERPING1	0.0000205	-3.66442
200993_at	IPO7	0.0055227	-2.95901
201001_s_at	TMEM189///TM	0.0398648	2.06621
201004_at	SSR4	0.0453342	1.79265
201005_at	CD9	0.0696987	-2.54922
201008_s_at	TXNIP	0.0000022	-12.49
201011_at	RPN1	0.0026248	1.61112
201017_at	EIF1AX	0.0145039	-2.37501
201019_s_at	EIF1AX///EIF1A	0.0086784	-2.37786
201023_at	TAF7	0.000194	-2.56501
201025_at	EIF5B	0.0393654	1.69774
201034_at	ADD3	0.0008041	-2.54076
201037_at	PFKP	0.0000023	3.11135
201055_s_at	HNRNPA0	0.0154005	1.33388
201067_at	PSMC2	0.0000003	-4.1887
201069_at	MMP2	0.0747353	1.7376
201074_at	SMARCC1	0.0005347	2.84091
201079_at	SYNGR2	0.0004314	1.58104
201088_at	KPNA2	0.0000357	2.995
201089_at	ATP6V1B2	0.0041206	-2.50236
201091_s_at	CBX3	0.0502767	1.81601
201097_s_at	ARF4	0.0162011	-1.97367
201099_at	USP9X	0.0254997	-2.30672
201110_s_at	THBS1	0.0176966	2.40732
201111_at	CSE1L	0.0013847	2.14939
201113_at	TUFM	0.0202732	1.83015
201116_s_at	CPE	0.0000005	-3.72361
201119_s_at	COX8A	0.0214226	1.4538
201125_s_at	ITGB5	0.0002719	-2.10252
201127_s_at	ACLY	0.0059956	1.74491
201129_at	SFRS7	0.0000222	-2.3673
201132_at	HNRNPH2	0.0375065	-2.80015

201133_s_at	PJA2	0.0002817	-5.5711
201136_at	PLP2	0.0061477	-1.92495
201140_s_at	RAB5C	0.0538403	1.3742
201143_s_at	EIF2S1	0.004792	-1.75866
201146_at	NFE2L2	0.000139	-4.87097
201150_s_at	TIMP3	0.0000004	-3.77966
201153_s_at	MBNL1	0.0077724	-2.03988
201162_at	IGFBP7	0.0001573	-3.62894
201166_s_at	PUM1	0.0107867	-1.9441
201174_s_at	TERF2IP	0.0007577	-4.3143
201176_s_at	ARCN1	0.0039351	-1.87153
201177_s_at	UBA2	0.0029762	-2.42906
201178_at	FBXO7	0.0000835	-2.01284
201184_s_at	CHD4	0.0004561	1.5006
201185_at	HTRA1	0.0000007	-7.35681
201197_at	AMD1	0.0036478	-2.10162
201200_at	CREG1	0.0164701	-1.89461
201201_at	CSTB	0.0258366	-1.99887
201204_s_at	RRBP1	0.0000088	2.05763
201207_at	TNFAIP1	0.0027508	-1.69659
201215_at	PLS3	0.023158	-2.2567
201223_s_at	RAD23B	0.0230201	-2.04333
201226_at	NDUFB8///SEC	0.0081729	-1.90746
201230_s_at	ARIH2	0.0012084	-2.08119
201238_s_at	CAPZA2	0.0181304	-2.56663
201241_at	DDX1	0.0000137	-4.12041
201250_s_at	SLC2A1	0.0000011	26.7524
201258_at	RPS16	0.0182997	-2.11585
201260_s_at	SYPL1	0.0000216	-6.74794
201266_at	TXNRD1	0.0000203	-4.40944
201271_s_at	RALY	0.0422732	1.58857
201272_at	AKR1B1	0.0047153	-2.10667
201273_s_at	SRP9	0.0295575	-2.30249
201274_at	PSMA5	0.0020398	-3.35366
201275_at	FDPS	0.0001203	2.12504
201279_s_at	DAB2	0.0000001	-2.64422
201286_at	SDC1	0.0003653	2.51732
201288_at	ARHGDIB	0.0400839	1.55078
201292_at	TOP2A	0.0004659	5.53899
201301_s_at	ANXA4	0	-7.11817

201312_s_at	SH3BGRL	0.0000331	-3.30776
201313_at	ENO2	0.0033328	2.51329
201314_at	STK25	0.0133481	1.37239
201317_s_at	PSMA2	0.0123856	-2.42965
201318_s_at	MYL12A///MYL	0.004374	-2.32587
201322_at	ATP5B	0.0466804	-2.23564
201325_s_at	EMP1	0.0032441	-1.71982
201328_at	ETS2	0.0000001	-5.30448
201330_at	RARS	0.0023977	-2.00029
201335_s_at	ARHGEF12	0.0002981	-1.91107
201336_at	VAMP3	0.0000002	-3.36923
201339_s_at	SCP2	0.0000226	-11.1846
201349_at	SLC9A3R1	0.0003595	-2.65934
201352_at	YME1L1	0.0003788	-1.75235
201359_at	COPB1	0.000575	-5.2725
201368_at	ZFP36L2	0.0006934	-2.99203
201371_s_at	CUL3	0.0050787	-2.45434
201375_s_at	PPP2CB	0.0007792	-3.98956
201377_at	UBAP2L	0.0137665	1.83614
201379_s_at	TPD52L2	0.0362931	1.35474
201380_at	CRTAP	0.0007949	-2.1548
201386_s_at	DHX15	0.0003401	-4.7147
201392_s_at	IGF2R	0.0282388	-2.04734
201394_s_at	RBM5	0.002588	-1.78039
201405_s_at	COPS6	0.0003764	-2.93196
201407_s_at	PPP1CB	0.0427217	1.75426
201413_at	HSD17B4	0.000114	-2.53755
201414_s_at	NAP1L4	0.0085064	1.26374
201415_at	GSS	0.005533	1.64971
201425_at	ALDH2	0.0005603	-3.02888
201426_s_at	VIM	0.0000045	-16.9576
201427_s_at	SEPP1	0.0002654	-2.64217
201431_s_at	DPYSL3	0.0007917	-3.16734
201432_at	CAT	0.0020572	-2.91951
201434_at	TTC1	0.0043549	-2.43875
201438_at	COL6A3	0.0000832	8.67397
201439_at	GBF1	0.0071887	1.34709
201441_at	COX6B1	0.004196	2.23774
201455_s_at	NPEPPS	0.0061995	-1.95545
201460_at	MAPKAPK2	0.0521261	1.19916

201462_at	SCRN1	0.0014456	-3.35725
201466_s_at	JUN	0.0358071	2.3226
201470_at	GSTO1	0.0000006	-3.55504
201479_at	DKC1	0.0827256	1.45208
201484_at	SUPT4H1	0.003513	-2.16828
201487_at	CTSC	0.0197123	-2.25619
201489_at	PPIF	0.0264436	2.11207
201515_s_at	TSN	0.0029992	-2.52303
201518_at	CBX1	0.0018238	-2.68366
201520_s_at	GRSF1	0.0022254	-5.40154
201521_s_at	NCBP2	0.0044208	2.35
201527_at	ATP6V1F	0.0001592	2.00364
201528_at	RPA1	0.0338997	-2.14704
201532_at	PSMA3	0.0004799	-6.82052
201534_s_at	UBL3	0.0000011	-4.75036
201536_at	DUSP3	0.0016652	-2.03179
201542_at	SAR1A	0.0040565	-2.14313
201545_s_at	PABPN1	0.0004989	1.73723
201546_at	TRIP12	0.0016232	-2.74892
201547_at	KDM5B	0.0331753	1.42303
201556_s_at	VAMP2	0.044219	1.31143
201560_at	CLIC4	0.0009895	-3.52624
201563_at	SORD	0.0000008	6.30911
201565_s_at	ID2	0.0125169	-2.01764
201567_s_at	GOLGA4	0.010738	-1.9162
201568_at	UQCRQ	0.0141363	1.59318
201570_at	SAMM50	0.0228712	-2.0499
201574_at	ETF1	0.0030488	-2.9773
201578_at	PODXL	0	-7.65645
201580_s_at	TMX4	0.0000727	-2.95607
201583_s_at	SEC23B	0.0019266	-2.22329
201587_s_at	IRAK1	0.0869083	1.38751
201588_at	TXNL1	0.0003793	-1.93656
201592_at	EIF3H	0.0003843	-2.81268
201599_at	OAT	0.0000078	-5.10446
201602_s_at	PPP1R12A	0.0037887	-3.12687
201608_s_at	PWP1	0.0002927	-1.69899
201612_at	ALDH9A1	0.0000575	-2.39782
201619_at	PRDX3	0.0009262	-3.06348
201626_at	INSIG1	0.0063635	-2.47026

201628_s_at	RRAGA	0.0002861	-2.37188
201641_at	BST2	0.0341133	1.95955
201646_at	SCARB2	0.0003011	-5.00728
201650_at	KRT19	0.0016479	-7.45774
201651_s_at	PACSIN2	0.0017594	-1.75925
201652_at	COPS5	0.0044033	-3.10924
201653_at	CNIH	0.0108797	-2.63457
201658_at	ARL1	0.0013857	-1.82946
201661_s_at	ACSL3	0.0000118	-3.49602
201664_at	SMC4	0.0022448	2.37143
201666_at	TIMP1	0.0000031	-6.1157
201667_at	GJA1	0.0002614	-4.62831
201669_s_at	MARCKS	0.0079956	-3.4254
201682_at	PMPCB	0.0041438	-1.89258
201684_s_at	TOX4	0.0629016	1.24981
201687_s_at	API5	0.001166	-2.3502
201693_s_at	EGR1	0.0139932	2.47628
201696_at	SFRS4	0.0314765	-2.02913
201697_s_at	DNMT1	0.001328	1.91488
201701_s_at	PGRMC2	0.0015729	-2.80251
201707_at	PEX19	0.0000525	-2.49957
201709_s_at	NIPSNAP1	0.0001662	1.61602
201715_s_at	ACIN1	0.0132938	1.60537
201719_s_at	EPB41L2	0.0117565	-2.0277
201723_s_at	GALNT1	0.028099	-2.14354
201733_at	CLCN3	0.0252303	-2.19512
201737_s_at	Mar 6, 2013	0.0000266	-4.02432
201738_at	EIF1B	0.0021199	-2.49647
201739_at	SGK1	0.0148015	-2.66955
201746_at	TP53	0.0424727	1.57596
201748_s_at	SAFB	0.0621797	1.33346
201751_at	JOSD1	0.0000039	-2.91524
201756_at	RPA2	0.0006995	-1.97721
201758_at	TSG101	0.0097217	-2.00248
201761_at	MTHFD2	0.0000591	11.8393
201762_s_at	PSME2	0.014665	1.86536
201768_s_at	CLINT1	0.0002287	1.26284
201773_at	ADNP	0.0028255	-2.87924
201776_s_at	KIAA0494	0.0875557	1.77762
201780_s_at	RNF13	0.0000288	-5.97305

201784_s_at	C11orf58	0.0017663	-2.92897
201788_at	DDX42	0.0009438	-2.1544
201794_s_at	SMG7	0.0019682	2.22672
201795_at	LBR	0.0678483	1.41064
201800_s_at	OSBP	0.0013978	-1.82808
201803_at	POLR2B	0.0006522	-2.54964
201807_at	VPS26A	0.0026892	-2.82246
201810_s_at	SH3BP5	0.0004726	-5.54704
201814_at	TBC1D5	0.0000709	-2.19049
201816_s_at	GBAS	0.0015372	-2.23933
201817_at	UBE3C	0.0918206	1.31216
201822_at	TIMM17A	0.0057227	1.21933
201823_s_at	RNF14	0.0070793	-1.92569
201825_s_at	SCCPDH	0.0013566	-2.52147
201827_at	SMARCD2	0.0035665	1.79247
201832_s_at	USO1	0.0000717	-6.6464
201838_s_at	SUPT7L	0.006396	-2.45327
201839_s_at	EPCAM	0.0000867	11.4416
201842_s_at	EFEMP1	0	-84.3868
201844_s_at	RYBP	0.0218756	-2.60489
201847_at	LIPA	0	-16.5657
201854_s_at	ATMIN	0.0000197	-3.44948
201857_at	ZFR	0.0003968	-2.5209
201860_s_at	PLAT	0.0980426	1.61089
201861_s_at	LRRFIP1	0.0034486	-3.12607
201870_at	TOMM34	0.0064674	1.29452
201874_at	MPZL1	0.0010703	-1.75629
201876_at	PON2	0.0069452	-1.83625
201877_s_at	PPP2R5C	0.0001572	-2.28858
201879_at	ARIH1	0.0279271	-2.00909
201886_at	DCAF11	0.0166602	1.44671
201887_at	IL13RA1	0.0027102	-1.71924
201890_at	RRM2	0.0000008	12.6971
201892_s_at	IMPDH2	0.0003468	-4.21562
201894_s_at	SSR1	0.0267373	-2.2852
201896_s_at	PSRC1	0.0003373	4.41597
201897_s_at	CKS1B	0.0069677	3.25871
201898_s_at	UBE2A	0.0053993	1.83552
201901_s_at	YY1	0.0121641	-2.69782
201913_s_at	COASY	0.0500392	1.41434

201915_at	SEC63	0.0031386	-1.80167
201919_at	SLC25A36	0.0156116	-2.30948
201921_at	GNG10	0.001488	-2.00748
201922_at	NSA2	0.0001017	-5.24061
201924_at	AFF1	0.001769	-2.26714
201925_s_at	CD55	0.0475505	-2.2325
201930_at	MCM6	0.0034499	1.64735
201933_at	CHMP1A	0.0047188	2.46527
201939_at	PLK2	0.0021179	-2.16363
201944_at	HEXB	0.0000641	-5.2449
201947_s_at	CCT2	0.0255585	-2.08996
201951_at	ALCAM	0.0068887	-2.32826
201955_at	CCNC	0.0066925	-2.60012
201956_s_at	GNPAT	0.0000735	-2.7387
201959_s_at	MYCBP2	0.011023	-3.24611
201962_s_at	RNF41	0.000847	1.63969
201963_at	ACSL1	0.000584	-4.1502
201964_at	SETX	0.0006097	-3.59757
201968_s_at	PGM1	0.0003035	-2.58927
201973_s_at	C7orf28A///C7c	0.0002882	-1.97815
201975_at	CLIP1	0.0156922	-2.09866
201976_s_at	MYO10	0.0009865	4.29627
201987_at	MED13	0.0007879	-2.62373
201988_s_at	CREBL2	0.0000779	-1.62676
201995_at	EXT1	0.020278	1.50065
201997_s_at	SPEN	0.0931099	1.37601
201998_at	ST6GAL1	0.0694487	1.73174
201999_s_at	DYNLT1	0.0013849	-2.44366
202001_s_at	NDUFA6	0.0003469	-1.74055
202003_s_at	ACAA2	0.0000018	-8.09529
202006_at	PTPN12	0.0114294	-2.3724
202012_s_at	EXT2	0.0393629	1.33343
202020_s_at	LANCL1	0.0000001	-5.95511
202026_at	SDHD	0.0136317	-3.49358
202027_at	TMEM184B	0.0000839	2.40299
202028_s_at	RPL38	0.0074112	1.70849
202032_s_at	MAN2A2	0.0021176	1.48552
202036_s_at	SFRP1	0.0000931	-9.76693
202038_at	UBE4A	0.01029	-2.39421
202039_at	MYO18A///TIAF	0.0230747	1.44646

202053_s_at	ALDH3A2	0.0000518	-2.40456
202059_s_at	KPNA1	0.0000089	4.36711
202060_at	CTR9	0.0000799	-5.49519
202061_s_at	SEL1L	0.0000435	-2.78414
202074_s_at	OPTN	0.0050217	-1.80604
202078_at	COPS3	0.0001357	-2.63997
202081_at	IER2	0.014949	1.63995
202082_s_at	SEC14L1	0.0291367	-2.59554
202087_s_at	CTSL1	0.0007129	-2.68233
202088_at	SLC39A6	0.0040427	-1.90125
202092_s_at	ARL2BP	0.0075071	-1.81593
202095_s_at	BIRC5	0.0000035	5.20432
202100_at	RALB	0.0000715	-2.12661
202105_at	IGBP1	0.0001606	-2.37211
202107_s_at	MCM2	0.0005007	2.58741
202113_s_at	SNX2	0.0002581	-3.37709
202119_s_at	CPNE3	0.0032294	-3.45134
202121_s_at	CHMP2A	0.0025423	-2.62343
202124_s_at	TRAK2	0.0054626	-2.20903
202130_at	RIOK3	0.002789	-2.23029
202135_s_at	ACTR1B	0.0054658	1.70052
202136_at	ZMYND11	0.0000059	-3.29572
202139_at	AKR7A2	0.0045772	-1.75419
202141_s_at	COPS8	0.000887	-1.93118
202144_s_at	ADSL	0.0002216	-2.77393
202148_s_at	PYCR1	0.0000431	1.79598
202149_at	NEDD9	0.0002114	4.06346
202162_s_at	CNOT8	0.0387523	1.51296
202166_s_at	PPP1R2	0.0051335	-3.5194
202168_at	TAF9	0.0015981	-3.29399
202169_s_at	AASDHPPT	0.0111241	-4.60032
202172_at	VEZF1	0.0198751	-1.91319
202182_at	KAT2A	0.0128833	1.3659
202185_at	PLOD3	0.0715434	1.28038
202191_s_at	GAS7	0.0239649	-2.41946
202197_at	MTMR3	0.0006289	-2.23923
202200_s_at	SRPK1	0.0002095	2.16665
202202_s_at	LAMA4	0.0000003	-14.2472
202208_s_at	ARL4C	0.0253392	1.79872
202211_at	ARFGAP3	0.0114388	-2.87032

202214_s_at	CUL4B	0.0005312	-4.68642
202217_at	C21orf33	0.0039145	-1.76498
202219_at	SLC6A8	0.0048428	2.50036
202220_at	KIAA0907	0.0589088	1.82144
202228_s_at	NPTN	0.0008502	-2.1973
202230_s_at	CHERP	0.0050535	1.7012
202231_at	EIF3M	0.000482	-2.09365
202236_s_at	SLC16A1	0.0006989	-2.41691
202239_at	PARP4	0.0001234	-4.88395
202241_at	TRIB1	0.0879485	1.73267
202249_s_at	DCAF8	0.0308609	1.4482
202251_at	PRPF3	0.0093539	1.65733
202254_at	SIPA1L1	0.000018	-2.38022
202256_at	CD2BP2	0.0360874	1.54567
202258_s_at	N4BP2L2	0.0000179	-2.50414
202260_s_at	STXBP1	0.00399	-2.30593
202264_s_at	TOMM40	0.0594334	1.58042
202266_at	TDP2	0.0001529	-8.5113
202267_at	LAMC2	0.0419863	2.08306
202268_s_at	NAE1	0.0013671	-2.7856
202271_at	FBXO28	0.000025	-3.39579
202277_at	SPTLC1	0.0125375	-2.37785
202279_at	C14orf2	0.0025121	2.28537
202283_at	SERPINF1	0.0559686	1.41761
202286_s_at	TACSTD2	0.0103862	5.05918
202290_at	PDAP1	0.0768885	1.20139
202291_s_at	MGP	0.0010639	-6.04416
202294_at	STAG1	0.000007	-2.50662
202297_s_at	RER1	0.0866726	1.29783
202300_at	HBXIP	0.0089951	-3.30821
202302_s_at	RSRC2	0.0243406	-2.05165
202304_at	FNDC3A	0.0000002	-9.49667
202309_at	MTHFD1	0.0641634	1.34016
202315_s_at	BCR	0.0837379	1.2274
202318_s_at	SENP6	0.0102915	-2.23283
202329_at	CSK	0.0072756	2.10245
202330_s_at	UNG	0.0003828	1.51225
202334_s_at	UBE2B	0.0003663	-1.75398
202337_at	PMF1	0.0386831	1.45108
202338_at	TK1	0.0000667	2.40048

202342_s_at	TRIM2	0.0278856	-2.74666
202345_s_at	FABP5	0.0033354	-4.02889
202348_s_at	TOR1A	0.0639235	1.19575
202350_s_at	MATN2	0.0078553	-2.59909
202351_at	ITGAV	0.0000008	-5.1363
202352_s_at	PSMD12	0.0481784	-2.38317
202361_at	SEC24C	0.0029516	1.63103
202363_at	SPOCK1	0	-75.1249
202364_at	MXI1	0.0003033	-3.04035
202370_s_at	CBFB	0.0030802	-1.81297
202371_at	TCEAL4	0.0016354	-2.07651
202373_s_at	RAB3GAP2	0.0001001	-2.80824
202376_at	SERPINA3	0.0310648	1.46596
202379_s_at	NKTR	0.0209023	-3.56996
202381_at	ADAM9	0.0055029	-2.71845
202382_s_at	GNPDA1	0.0007911	-2.17254
202383_at	KDM5C	0.0547376	1.42625
202385_s_at	TCOF1	0.0049019	1.52477
202386_s_at	KIAA0430	0.0000024	-4.81336
202387_at	BAG1	0.0165323	-1.91342
202388_at	RGS2	0.0016648	-3.71194
202390_s_at	HTT	0.0830659	1.20246
202391_at	BASP1	0.034469	-2.44086
202393_s_at	KLF10	0.0000051	-4.67751
202397_at	NUTF2	0.0011814	1.57133
202404_s_at	COL1A2	0.0029133	2.81437
202409_at	IGF2///INS-IGF2	0.0001761	2.89478
202411_at	IFI27	0.0032913	4.54603
202413_s_at	USP1	0.0163557	-2.46462
202414_at	ERCC5	0.0001689	-2.43133
202417_at	KEAP1	0.0255384	1.75341
202419_at	KDSR	0.0004239	-1.83408
202422_s_at	ACSL4	0.0014308	-1.9967
202426_s_at	RXRA	0.0272371	1.30623
202429_s_at	PPP3CA	0.0057081	-3.8197
202432_at	PPP3CB	0.0003445	-2.82422
202433_at	SLC35B1	0.0073864	-1.879
202440_s_at	ST5	0.0037046	1.67952
202441_at	ERLIN1	0.0019361	-2.22338
202442_at	AP3S1	0.0004886	-1.8849

202446_s_at	PLSCR1	0.0063003	-2.92648
202447_at	DECR1	0.0000093	-4.71581
202450_s_at	CTSK	0.0124135	-2.86355
202451_at	GTF2H1	0.0022332	-2.05688
202461_at	EIF2B2	0.0047461	-1.75882
202464_s_at	PFKFB3	0.0041059	2.08416
202467_s_at	COPS2	0.0001413	-2.47401
202468_s_at	CTNNAL1	0.0000016	-11.4984
202478_at	TRIB2	0.0022577	1.89683
202484_s_at	MBD2	0.0002337	-3.87026
202488_s_at	FXYD3	0.0088234	1.86474
202491_s_at	IKBKAP	0.0007381	-2.25014
202502_at	ACADM	0.0003868	-2.68523
202503_s_at	KIAA0101	0.000009	12.7374
202506_at	SSFA2	0.0004684	-3.70832
202510_s_at	TNFAIP2	0.0061628	1.80831
202512_s_at	ATG5	0.0000061	-6.14966
202513_s_at	PPP2R5D	0.031222	1.28053
202518_at	BCL7B	0.0259095	1.40007
202519_at	MLXIP	0.0122508	-2.13464
202520_s_at	MLH1	0.0000408	-2.97356
202524_s_at	SPOCK2	0.0035605	-1.98936
202527_s_at	SMAD4	0.0050297	-3.03979
202531_at	IRF1	0.0622341	1.43207
202538_s_at	CHMP2B	0.0170945	-2.35108
202542_s_at	AIMP1	0.0231321	-1.98461
202544_at	GMFB	0.0000006	-3.97587
202546_at	VAMP8	0.0742267	1.73291
202547_s_at	ARHGEF7	0.0120892	1.46522
202550_s_at	VAPB	0.0307512	1.59436
202552_s_at	CRIM1	0.0000003	-6.76798
202553_s_at	SYF2	0.0002109	-2.07029
202555_s_at	MYLK	0.0103137	-4.05732
202561_at	TNKS	0.0000695	-2.86729
202563_at	C14orf1	0.0795274	1.43479
202565_s_at	SVIL	0.0074858	3.47443
202568_s_at	MARK3	0.0001886	-3.4069
202572_s_at	DLGAP4	0.0007599	1.35002
202581_at	HSPA1A///HSP/	0.0092503	2.87831
202589_at	TYMS	0.0001664	3.81767

202591_s_at	SSBP1	0.037486	-2.88789
202592_at	BLOC1S1	0.0739191	1.32605
202595_s_at	LEPROTL1	0.007219	-2.06196
202598_at	S100A13	0.000001	5.04959
202602_s_at	HTATSF1	0.0006391	-2.47449
202607_at	NDST1	0.0010263	1.49686
202609_at	EPS8	0.0000011	-7.91947
202611_s_at	MED14	0.0033845	-2.57321
202613_at	CTPS	0.0247606	1.57701
202619_s_at	PLOD2	0.0465666	-2.35976
202623_at	EAPP	0.0028848	-3.15235
202631_s_at	APPBP2	0.004057	-1.83939
202633_at	TOPBP1	0.0678567	1.75234
202636_at	RNF103	0.0000432	-2.66646
202641_at	ARL3	0.0034263	-1.79129
202644_s_at	TNFAIP3	0.038553	1.77848
202661_at	ITPR2	0.0000553	-2.58367
202664_at	WIPF1	0.0021857	-2.90935
202675_at	SDHB	0.0048562	-2.79043
202679_at	NPC1	0.0060784	-2.38726
202680_at	GTF2E2	0.0005831	-3.51875
202682_s_at	USP4	0.0060692	-1.98933
202688_at	TNFSF10	0.0528153	-3.04559
202693_s_at	STK17A	0.019416	-2.35318
202705_at	CCNB2	0.0000233	5.39432
202710_at	BET1	0.001677	-3.95968
202715_at	CAD	0.0081698	1.47711
202720_at	TES	0.0021567	2.63993
202723_s_at	FOXO1	0.0000316	-2.34966
202729_s_at	LTBP1	0.0109984	3.35567
202731_at	PDCD4	0.0000709	-2.36535
202732_at	PKIG	0.0002668	-2.19846
202733_at	P4HA2	0.0000365	-3.85871
202735_at	EBP	0.0006593	2.34108
202737_s_at	LSM4	0.0000594	3.77994
202738_s_at	PHKB	0.0005122	-2.79439
202741_at	PRKACB	0	-6.41123
202746_at	ITM2A	0.0040876	-3.09543
202748_at	GBP2	0.0050108	-2.86101
202749_at	WRB	0.0003374	-6.65466

202753_at	PSMD6	0.0077563	-3.35048
202754_at	R3HDM1	0.000306	1.85159
202755_s_at	GPC1	0.0605319	1.69518
202757_at	COBRA1	0.013534	1.51005
202761_s_at	SYNE2	0.002177	-3.53291
202762_at	ROCK2	0.0033604	-2.82751
202770_s_at	CCNG2	0.0060031	-2.41208
202776_at	DNTTIP2	0.0251485	-2.05995
202780_at	OXCT1	0.0010723	-3.51185
202783_at	NNT	0.0195579	-1.92388
202786_at	STK39	0.0053661	-3.72371
202789_at	PLCG1	0.0073895	1.97319
202790_at	CLDN7	0.0019604	1.80511
202794_at	INPP1	0.0000649	-2.6914
202798_at	SEC24B	0.0000327	-7.86384
202800_at	SLC1A3	0.0015476	2.52952
202806_at	DBN1	0.0000076	3.52096
202810_at	DRG1	0.001359	-1.81831
202813_at	TARBP1	0.0734371	1.83505
202820_at	AHR	0.0020042	-2.28095
202825_at	SLC25A4	0.000106	-2.93704
202829_s_at	VAMP7	0.0004332	-4.81883
202832_at	GCC2	0.0016176	-2.16982
202834_at	AGT	0.0050791	1.33815
202836_s_at	TXNL4A	0.0323255	1.63952
202838_at	FUCA1	0	-3.90712
202840_at	TAF15	0.0007252	-1.89004
202852_s_at	AAGAB	0.0143595	1.36762
202858_at	U2AF1	0.0134324	-2.08982
202860_at	DENND4B	0.0002858	2.22565
202863_at	SP100	0.0025041	-3.39691
202870_s_at	CDC20	0.0087089	1.95737
202876_s_at	PBX2	0.0027812	1.88083
202878_s_at	CD93	0.0026919	3.49108
202880_s_at	CYTH1	0.0109167	1.40738
202890_at	MAP7	0.046496	1.5283
202891_at	NIT1	0.0008552	1.43066
202894_at	EPHB4	0.0008239	1.84459
202897_at	SIRPA	0.0009757	-1.72838
202899_s_at	SFRS3	0.0211863	-2.51075

202900_s_at	NUP88	0.0190778	-2.26389
202909_at	EPM2AIP1	0.0069476	-2.26165
202916_s_at	FAM20B	0.0000838	-1.88894
202920_at	ANK2	0.0013474	-2.38279
202923_s_at	GCLC	0.0287468	1.69489
202927_at	PIN1	0.0279336	1.54386
202930_s_at	SUCLA2	0.0003674	-5.988
202932_at	YES1	0.0175291	-4.13679
202934_at	HK2	0.0032052	3.21407
202936_s_at	SOX9	0.0002313	2.92137
202941_at	NDUFV2	0.0080806	-2.16061
202948_at	IL1R1	0.0022086	-2.59282
202949_s_at	FHL2	0.0017804	-2.83251
202950_at	CRYZ	0.0008954	-5.79562
202952_s_at	ADAM12	0.0029368	1.49465
202954_at	UBE2C	0.0002852	3.15164
202960_s_at	MUT	0.0039413	-1.74254
202961_s_at	ATP5J2	0.0000087	3.59592
202964_s_at	RFX5	0.0097579	1.88317
202967_at	GSTA4	0.006591	-2.09836
202968_s_at	DYRK2	0.0001653	2.81598
202985_s_at	BAG5	0.01212	1.44401
202986_at	ARNT2	0.0000527	-2.1368
202990_at	PYGL	0.0006984	-1.76156
202998_s_at	LOXL2	0.0000141	2.05525
203006_at	INPP5A	0.0083007	-1.85877
203011_at	IMPA1	0.0022181	-2.7582
203018_s_at	SSX2IP	0.0000058	-3.8211
203022_at	RNASEH2A	0.0009497	2.30811
203024_s_at	C5orf15	0.0081674	-2.3398
203025_at	NAA10	0.017888	2.05158
203029_s_at	PTPRN2	0.0006392	-3.85355
203035_s_at	PIAS3	0.0364938	1.63722
203037_s_at	MTSS1	0.0005017	-3.56216
203040_s_at	HMBS	0.0020613	1.63413
203044_at	CHSY1	0.0022338	1.94001
203046_s_at	TIMELESS	0.0000374	2.99835
203048_s_at	TTC37	0.0027154	-2.63087
203058_s_at	PAPSS2	0.0000003	-7.63121
203062_s_at	MDC1	0.0515511	1.61975

203064_s_at	FOXK2	0.000044	2.14442
203069_at	SV2A	0.0079764	1.39062
203074_at	ANXA8///ANXA	0	-87.2669
203083_at	THBS2	0.0188116	3.48827
203090_at	SDF2	0.0005836	-2.92612
203094_at	MAD2L1BP	0.0022391	-1.96179
203097_s_at	RAPGEF2	0.0029618	-2.17177
203102_s_at	MGAT2	0.0305347	-2.48381
203112_s_at	WHSC2	0.002471	1.34218
203115_at	FECH	0.0035935	-1.87022
203117_s_at	PAN2	0.0174286	1.24836
203120_at	TP53BP2	0.0012142	-1.7263
203131_at	PDGFRA	0.0088368	-4.76495
203138_at	HAT1	0.0543262	-2.37184
203139_at	DAPK1	0	-18.0507
203144_s_at	KIAA0040	0.0068888	1.68665
203145_at	SPAG5	0.0002927	2.42722
203152_at	MRPL40	0.004569	-1.92895
203153_at	IFIT1	0.020266	-4.64968
203155_at	SETDB1	0.0004193	1.5043
203156_at	AKAP11	0.0000001	-11.7326
203158_s_at	GLS	0.0007408	-1.87895
203162_s_at	KATNB1	0.0671007	1.76528
203166_at	CFDP1	0.0002802	-4.18737
203168_at	ATF6B	0.0001086	1.984
203178_at	GATM	0.0016347	-4.82512
203180_at	ALDH1A3	0	-28.0162
203186_s_at	S100A4	0.0005693	5.44429
203188_at	B3GNT1	0.0015913	-2.21863
203190_at	NDUFS8	0.0557157	1.76121
203194_s_at	NUP98	0.0054003	1.17918
203203_s_at	KRR1	0.0209236	-2.38294
203213_at	CDK1	0.0000985	9.61919
203217_s_at	ST3GAL5	0.0000011	-3.23444
203227_s_at	TSPAN31	0.0016767	-2.3102
203232_s_at	ATXN1	0.0000482	-2.44986
203245_s_at	NCRNA00094	0.0001982	-4.29801
203249_at	EZH1	0.0005361	-1.68184
203253_s_at	PPIP5K2	0.0021823	-4.36155
203260_at	HDDC2	0.0009688	-2.3831

203261_at	DCTN6	0.0002508	-2.66516
203266_s_at	MAP2K4	0.0001104	-1.76927
203272_s_at	TUSC2	0.0004836	1.92392
203276_at	LMNB1	0.0038669	3.66323
203283_s_at	HS2ST1	0.0025205	-2.05337
203286_at	RNF44	0.0173856	1.63853
203288_at	KIAA0355	0.0003913	-2.95704
203289_s_at	NPRL3	0.0045562	1.6296
203297_s_at	JARID2	0.0251135	1.58489
203301_s_at	DMTF1	0.0092661	-2.17334
203303_at	DYNLT3	0.0043723	-2.73542
203304_at	BAMBI	0	-12.7407
203306_s_at	SLC35A1	0.0007122	-3.01678
203307_at	GNL1	0.0148026	1.97676
203310_at	STXBP3	0.0000678	-3.19362
203317_at	PSD4	0.002716	1.62112
203324_s_at	CAV2	0	-9.10276
203325_s_at	COL5A1	0.0000116	4.14849
203333_at	KIFAP3	0.0004421	-3.05295
203336_s_at	ITGB1BP1	0.0008208	-3.35502
203341_at	CEBPZ	0.005265	-2.88896
203347_s_at	MTF2	0.0129656	2.28698
203354_s_at	PSD3	0.0323505	-2.27339
203356_at	CAPN7	0.00445	-2.27909
203358_s_at	EZH2	0.0000307	6.07678
203362_s_at	MAD2L1	0.0298255	2.77158
203366_at	POLG	0.0325696	1.31019
203367_at	DUSP14	0.0543891	1.36195
203373_at	SOCS2	0.0001313	-2.67648
203376_at	CDC40	0.0000641	-3.4828
203378_at	PCF11	0.0065301	-2.10953
203384_s_at	GOLGA1	0.0000901	-1.66981
203386_at	TBC1D4	0.0005253	-2.71757
203389_at	KIF3C	0.0658634	1.32871
203394_s_at	HES1	0.0198093	2.11009
203396_at	PSMA4	0.0029229	-2.34378
203403_s_at	RNF6	0.0058398	-4.05795
203404_at	ARMCX2	0.0012012	-2.72626
203406_at	MFAP1	0.0000239	-3.02526
203408_s_at	SATB1	0.000027	-6.71333

203412_at	LZTR1	0.0927653	1.2868
203413_at	NELL2	0	-127.49
203414_at	MMD	0.0916268	1.52485
203416_at	CD53	0.0165672	-3.43712
203420_at	FAM8A1	0.000162	-3.29594
203427_at	ASF1A	0.0030119	-2.98003
203429_s_at	C1orf9	0.0001859	-2.17537
203431_s_at	ARHGAP32	0.0002153	-2.94907
203436_at	RPP30	0.0002475	-2.51803
203438_at	STC2	0.0000862	3.22055
203448_s_at	TERF1	0.001639	-2.26426
203453_at	SCNN1A	0.0000024	6.12201
203454_s_at	ATOX1	0.0136104	1.69409
203456_at	PRAF2	0.0011345	1.3254
203464_s_at	EPN2	0.037933	1.33661
203468_at	CDK10	0.0732783	1.89192
203475_at	CYP19A1	0.0001714	-3.93881
203476_at	TPBG	0.0000269	-7.89882
203477_at	COL15A1	0.0038836	2.63815
203478_at	NDUFC1	0.0000716	-2.05468
203482_at	FAM178A	0.0053382	-1.74357
203491_s_at	CEP57	0.0290776	-2.79029
203495_at	LRRC14	0.0177868	1.59474
203497_at	MED1	0.002568	-3.23107
203501_at	PGCP	0.0000014	-6.03249
203512_at	TRAPPC3	0.0105018	1.45479
203513_at	SPG11	0.000207	-2.62823
203515_s_at	PMVK	0.0192215	1.51821
203518_at	LYST	0.0118791	-2.37417
203522_at	CCS	0.0156335	1.60638
203525_s_at	APC	0.0012043	-3.17834
203529_at	PPP6C	0.0016672	1.59505
203531_at	CUL5	0.0091322	-2.10316
203536_s_at	CIAO1	0.0052568	1.47299
203537_at	PRPSAP2	0.0085962	-1.89111
203542_s_at	KLF9	0.0000016	-5.95362
203544_s_at	STAM	0.0047135	-2.39942
203546_at	IPO13	0.0003902	1.64957
203549_s_at	LPL	0.0434883	1.59309
203550_s_at	FAM189B	0.01602	1.6781

203551_s_at	COX11	0.0041603	-2.20863
203556_at	ZHX2	0.0433269	1.62579
203574_at	NFIL3	0.0058165	-2.27044
203578_s_at	SLC7A6	0.0067645	1.48423
203581_at	RAB4A	0.0400436	1.51114
203583_at	UNC50	0.0009705	-3.37794
203584_at	TTC35	0.0074543	-3.91164
203585_at	ZNF185	0.0031966	-2.33172
203588_s_at	TFDP2	0.0213788	1.90663
203593_at	CD2AP	0.0314606	-2.70398
203597_s_at	WBP4	0.0000059	-2.51956
203600_s_at	FAM193A	0.0195216	1.26626
203603_s_at	ZEB2	0.0062582	-2.68652
203604_at	ZNF516	0.0078656	-1.90098
203606_at	NDUFS6	0.0179992	1.72435
203607_at	INPP5F	0.0003311	-2.52566
203614_at	UTP14C	0.0023287	-3.55916
203620_s_at	FCHSD2	0.0664264	1.71595
203632_s_at	GPRC5B	0.0006172	4.54011
203635_at	DSCR3	0.0016145	-2.81945
203640_at	MBNL2	0.0011979	-2.44591
203642_s_at	COBLL1	0	-12.6253
203645_s_at	CD163	0.0061935	-4.02061
203650_at	PROCR	0	-21.8477
203651_at	ZFYVE16	0.00095	-2.36518
203656_at	FIG4	0.000717	-3.01266
203661_s_at	TMOD1	0.0002437	-7.40622
203675_at	NUCB2	0.0002646	-2.52765
203678_at	MTMR15	0.0051589	-1.783
203680_at	PRKAR2B	0	-35.8168
203685_at	BCL2	0.0000008	-8.60957
203688_at	PKD2	0.0000001	-9.74057
203690_at	TUBGCP3	0.0013211	-3.4154
203695_s_at	DFNA5	0	-40.0908
203700_s_at	DIO2	0.0127142	1.473
203703_s_at	TTLL4	0.0011434	1.3244
203705_s_at	FZD7	0.0003386	-7.39321
203708_at	PDE4B	0.0045368	-2.90656
203710_at	ITPR1	0.0000626	-3.21401
203713_s_at	LLGL2	0.0013877	2.34804

203717_at	DPP4	0.0002201	-2.78344
203718_at	PNPLA6	0.0022875	1.39371
203721_s_at	UTP18	0.00109	-4.78242
203725_at	GADD45A	0.0388019	-2.07847
203731_s_at	ZKSCAN5	0.0029579	1.36634
203732_at	TRIP4	0.0000195	-2.51188
203734_at	FOXJ2	0.0036956	-1.81848
203737_s_at	PPRC1	0.0293187	1.33106
203740_at	MPHOSPH6	0.0000003	-5.29298
203741_s_at	ADCY7	0.0886173	1.62979
203744_at	HMGB3	0.0012474	3.67643
203755_at	BUB1B	0.0000006	7.44104
203758_at	CTSO	0.0001332	-5.23239
203763_at	DYNC2LI1	0.0003819	-2.47996
203764_at	DLGAP5	0.0001363	8.0389
203765_at	GCA	0.0000123	-4.98589
203778_at	MANBA	0.000664	-1.9298
203781_at	MRPL33	0.0283052	1.48953
203788_s_at	SEMA3C	0.0000984	-5.99415
203791_at	DMXL1	0.0108393	-2.29332
203797_at	VSNL1	0.0111679	-1.87445
203799_at	CD302	0	-9.19558
203801_at	MRPS14	0.0000201	-2.86051
203810_at	DNAJB4	0.0001022	-5.24837
203814_s_at	NQO2	0.0088377	-1.88883
203817_at	GUCY1B3	0.0086797	3.15511
203822_s_at	ELF2	0.0018782	-3.76956
203824_at	TSPAN8	0.0050418	-5.00566
203829_at	ELP4	0.0008361	-3.59092
203831_at	R3HDM2	0.0269844	1.48581
203845_at	KAT2B	0.0000093	-8.13169
203855_at	WDR47	0.0025767	-2.01184
203856_at	VRK1	0.0136189	1.86757
203857_s_at	PDIA5	0.0335605	2.0376
203860_at	PCCA	0.0000003	-5.16415
203874_s_at	SMARCA1	0.0129792	-2.14511
203881_s_at	DMD	0.0000022	-10.7612
203883_s_at	RAB11FIP2	0.0001124	-4.66559
203885_at	RAB21	0.0030279	-2.06772
203889_at	SCG5	0.0000018	-18.617

203892_at	WFDC2	0	14.5697
203895_at	PLCB4	0.0659047	1.54852
203897_at	LYRM1	0.0058072	-2.88892
203898_at	CRCP	0.0384688	1.20195
203909_at	SLC9A6	0.0014921	-2.34245
203911_at	RAP1GAP	0.0000178	1.80474
203913_s_at	HPGD	0.0000921	-3.69629
203915_at	CXCL9	0.0099599	-4.47266
203916_at	NDST2	0.0040712	1.70364
203921_at	CHST2	0.0017836	1.38529
203934_at	KDR	0.0000001	-7.15353
203935_at	ACVR1	0.0030934	-3.69538
203938_s_at	TAF1C	0.0035568	1.52566
203940_s_at	VASH1	0.0020509	2.31883
203956_at	MORC2	0.00291	2.18723
203962_s_at	NEBL	0.0001286	-2.88163
203968_s_at	CDC6	0.0000097	1.66843
203970_s_at	PEX3	0.0026525	-1.78187
203974_at	HDHD1A	0.0032059	-1.75749
203978_at	NUBP1	0.0057922	-1.88617
203987_at	FZD6	0.0284093	-3.40298
203988_s_at	FUT8	0.0005089	1.92114
203992_s_at	KDM6A	0.0046746	-2.01977
203999_at	SYT1	0	-21.4012
204007_at	FCGR3B	0.0280431	-2.87954
204021_s_at	PURA	0.0001071	-1.67426
204024_at	OSGIN2	0.0020868	-1.75346
204026_s_at	ZWINT	0.0000007	19.2697
204027_s_at	METTL1	0.0144741	1.68477
204028_s_at	RABGAP1	0.0037547	-2.4402
204031_s_at	PCBP2	0.0287964	-2.46243
204032_at	BCAR3	0.0000001	-7.5657
204033_at	TRIP13	0.001024	6.57352
204036_at	LPAR1	0.034939	-2.57891
204040_at	RNF144A	0.0285165	1.46815
204041_at	MAOB	0.0000048	-8.74603
204042_at	WASF3	0.0000039	-6.75835
204044_at	QPRT	0.0027526	2.63755
204045_at	TCEAL1	0.0000641	-3.46921
204049_s_at	PHACTR2	0.0000081	-10.724

204055_s_at	CTAGE5	0.0157426	-2.0575
204060_s_at	PRKX///PRKY	0.0000157	1.99973
204061_at	PRKX	0.0116141	1.50397
204063_s_at	ULK2	0.0003535	-2.25533
204068_at	STK3	0.0013323	-2.51163
204069_at	MEIS1	0.0001023	2.26817
204071_s_at	TOPORS	0.0021057	-2.12662
204073_s_at	C11orf9	0.0005168	-2.22115
204078_at	SC65	0.0090569	1.52883
204082_at	PBX3	0.0000035	-5.85023
204084_s_at	CLN5	0.0010298	-3.61097
204093_at	CCNH	0.0000032	-2.64395
204105_s_at	NRCAM	0.0043862	-2.6848
204112_s_at	HNMT	0.0000246	-3.5379
204113_at	CELF1	0.0018227	1.81466
204114_at	NID2	0.0038256	-3.70847
204115_at	GNG11	0.000003	-4.26014
204124_at	SLC34A2	0.0020918	3.37585
204129_at	BCL9	0.0003592	1.37115
204136_at	COL7A1	0.0000207	3.49516
204137_at	GPR137B	0.000002	-9.845
204143_s_at	ENOSF1	0.0091708	-2.30169
204145_at	FRG1	0.0006484	-2.40964
204146_at	RAD51AP1	0.0002732	8.11416
204154_at	CDO1	0.0095071	-2.17168
204161_s_at	ENPP4	0.0004311	-3.22228
204165_at	WASF1	0.0107314	-2.711
204167_at	BTD	0.000009	-2.15034
204169_at	IMPDH1	0.0334247	1.45489
204170_s_at	CKS2	0.0000037	9.80784
204172_at	CPOX	0.0041139	-2.07672
204173_at	MYL6B	0.0013037	1.78096
204176_at	KLHL20	0.0071964	-2.65183
204190_at	USPL1	0.0000129	-2.61646
204194_at	BACH1	0.0005642	-3.64938
204197_s_at	RUNX3	0.000988	2.05552
204201_s_at	PTPN13	0.0000033	-5.81565
204204_at	SLC31A2	0.000004	-4.35792
204206_at	MNT	0.0005586	1.78356
204214_s_at	RAB32	0.0002043	-2.11077

204215_at	C7orf23	0.0005765	-4.08463
204219_s_at	PSMC1	0.0001318	-2.69232
204226_at	STAU2	0.032898	-2.31802
204230_s_at	SLC17A7	0.043587	1.1971
204233_s_at	CHKA	0.0014381	1.68685
204237_at	GULP1	0.000037	-6.32057
204240_s_at	SMC2	0.0279619	1.71217
204251_s_at	CEP164	0.0768031	1.22802
204252_at	CDK2	0.0007191	1.45217
204254_s_at	VDR	0.0028522	3.07018
204256_at	ELOVL6	0.0013155	4.35043
204257_at	FADS3	0.0426922	1.85338
204259_at	MMP7	0.0014337	5.66876
204260_at	CHGB	0	-12.8047
204268_at	S100A2	0.0003075	26.4036
204271_s_at	EDNRB	0.0056479	-4.96258
204278_s_at	EBAG9	0.0283044	-2.09647
204279_at	PSMB9	0.0039915	-3.43733
204284_at	PPP1R3C	0.0001751	-1.83782
204294_at	AMT	0.0002981	1.67815
204297_at	PIK3C3	0.00025	-2.16647
204301_at	KBTBD11	0.0000011	-4.65589
204324_s_at	GOLIM4	0.0074706	-4.36557
204328_at	TMC6	0.0046109	1.87998
204332_s_at	AGA	0.0001081	-2.18403
204334_at	KLF7	0.0011293	-2.15385
204336_s_at	RGS19	0.0050396	2.24195
204337_at	RGS4	0	-52.8012
204350_s_at	MED7	0.0007819	-1.89075
204352_at	TRAF5	0.0006089	-2.77161
204358_s_at	FLRT2	0.0003484	-2.11683
204360_s_at	NAGLU	0.0389704	1.52097
204364_s_at	REEP1	0	-44.8302
204368_at	SLCO2A1	0.0012291	1.81429
204372_s_at	KHSRP	0.0383385	1.4523
204373_s_at	CEP350	0.0221933	-2.08355
204377_s_at	VPRBP	0.0016844	1.55987
204379_s_at	FGFR3	0.0325813	2.89326
204388_s_at	MAOA	0.0003939	-4.43976
204393_s_at	ACPP	0.0086241	-4.33631

204407_at	TTF2	0.0479596	2.27208
204412_s_at	NEFH	0.0002256	-2.18666
204415_at	IFI6	0.0475948	2.27089
204417_at	GALC	0.0000012	-11.8011
204420_at	FOSL1	0.0009165	1.33046
204422_s_at	FGF2	0.0000297	-2.43908
204424_s_at	LMO3	0.0039247	2.56071
204425_at	ARHGAP4	0.0007901	2.19212
204429_s_at	SLC2A5	0.0268389	1.30572
204432_at	SOX12	0.0005868	2.24054
204437_s_at	FOLR1	0.000262	8.49936
204438_at	MRC1///MRC1L	0.0000339	-3.62813
204440_at	CD83	0.0199012	-2.88863
204444_at	KIF11	0.0029814	2.62786
204457_s_at	GAS1	0.0000003	-33.9575
204464_s_at	EDNRA	0.0266863	-3.24109
204467_s_at	SNCA	0.0000043	-4.71145
204469_at	PTPRZ1	0.0000085	-7.38958
204474_at	ZNF142	0.0011	1.54533
204485_s_at	TOM1L1	0.0002023	-5.14798
204491_at	PDE4D	0.0226521	1.31128
204495_s_at	C15orf39	0.0003887	1.62156
204499_at	AGTPBP1	0.0056095	-1.75089
204510_at	CDC7	0.0006238	1.93506
204516_at	ATXN7	0.0073417	-2.66012
204517_at	PPIC	0.0028437	2.17797
204519_s_at	PLLP	0.0102158	-2.01224
204523_at	ZNF140	0.0020919	-2.19001
204542_at	ST6GALNAC2	0.00066	3.12974
204544_at	HPS5	0.0006628	-2.15774
204547_at	RAB40B	0.0133659	-2.97213
204558_at	RAD54L	0.0002882	2.08198
204566_at	PPM1D	0.0002174	-4.47438
204568_at	KIAA0831	0.0000007	-2.5137
204573_at	CROT	0.0000231	-3.98935
204576_s_at	CLUAP1	0.0028326	-2.45553
204578_at	PPIP5K1	0.0102734	1.35234
204584_at	L1CAM	0.0005461	3.86104
204588_s_at	SLC7A7	0.0059691	-1.86671
204589_at	NUAK1	0.0003767	3.46148

204600_at	EPHB3	0.0000754	1.82228
204602_at	DKK1	0.0086067	-2.08381
204604_at	CDK14	0.0007342	-1.811
204605_at	CGRRF1	0.000033	-3.28591
204613_at	PLCG2	0.0052168	-2.23858
204616_at	UCHL3	0.0017147	-3.08315
204619_s_at	VCAN	0.0000408	10.6445
204624_at	ATP7B	0.0000682	-4.6991
204627_s_at	ITGB3	0.0005031	1.79714
204633_s_at	RPS6KA5	0.0035307	-1.98231
204639_at	ADA	0.0001143	2.52632
204641_at	NEK2	0.0000009	9.4489
204643_s_at	ENOX2	0.0004566	-2.11593
204646_at	DPYD	0	-57.4465
204647_at	HOMER3	0.0667035	1.37616
204650_s_at	APBB3	0.0728063	1.26813
204652_s_at	NRF1	0.0334702	1.47606
204653_at	TFAP2A	0.0012348	10.7722
204659_s_at	GFER	0.0158969	1.26016
204665_at	SIKE1	0.0907856	1.23062
204675_at	SRD5A1	0.0002726	5.47294
204679_at	KCNK1	0.0004995	-2.74604
204684_at	NPTX1	0.0000631	-2.01535
204688_at	SGCE	0.000002	-14.4082
204690_at	STX8	0.0000378	-2.223
204703_at	IFT88	0.0000082	-3.3616
204709_s_at	KIF23	0.0048149	2.31882
204712_at	WIF1	0.0234816	1.43804
204717_s_at	SLC29A2	0.0044291	1.72611
204719_at	ABCA8	0	-128.699
204720_s_at	DNAJC6	0.0018367	-3.37055
204722_at	SCN3B	0.0022371	-2.52649
204725_s_at	NCK1	0.0162229	-2.06042
204728_s_at	WDHD1	0.0136174	1.62009
204732_s_at	TRIM23	0.0013936	-2.77799
204733_at	KLK6	0.0003399	2.99356
204739_at	CENPC1	0.0002731	-2.94863
204740_at	CNKSR1	0.0000309	2.22967
204749_at	NAP1L3	0	-21.6601
204754_at	HLF	0.0000117	-3.13601

204757_s_at	C2CD2L	0.045485	1.36905
204759_at	RCBTB2	0.0000263	-2.96617
204766_s_at	NUDT1	0.0174036	1.71081
204767_s_at	FEN1	0.000018	2.38571
204771_s_at	TTF1	0.0134161	-1.89955
204773_at	IL11RA	0.0023684	-1.978
204777_s_at	MAL	0.0000032	30.5076
204779_s_at	HOXB7	0.0017513	2.26807
204781_s_at	FAS	0.000007	-2.19457
204783_at	MLF1	0.0034949	-2.46261
204787_at	VSIG4	0.0071855	-2.47374
204788_s_at	PPOX	0.0046127	1.50597
204793_at	GPRASP1	0	-8.47312
204797_s_at	EML1	0.0000374	-3.46328
204798_at	MYB	0.0001793	2.50426
204807_at	TMEM5	0.0281805	-2.27655
204818_at	HSD17B2	0.0000005	-15.994
204820_s_at	BTN3A2///BTN3	0.005253	-3.28601
204821_at	BTN3A3	0.0000326	-2.29329
204822_at	TTK	0.0039595	4.84
204823_at	NAV3	0	-14.6352
204825_at	MELK	0.0000907	9.73313
204826_at	CCNF	0.0078936	1.45093
204831_at	CDK8	0.006364	-1.96268
204834_at	FGL2	0	-3.56571
204836_at	GLDC	0.0001348	8.29832
204847_at	ZBTB11	0.0032681	-2.42145
204853_at	ORC2L	0.0894586	1.34049
204866_at	PHF16	0.0250931	1.74924
204872_at	TLE4	0.0000115	-7.95862
204886_at	PLK4	0.0776164	1.7286
204897_at	PTGER4	0	-26.8101
204906_at	RPS6KA2	0.0236188	1.86985
204913_s_at	SOX11	0.0471025	2.03205
204916_at	RAMP1	0.0617876	1.48786
204924_at	TLR2	0.0269336	-2.24017
204931_at	TCF21	0.0004251	-3.92889
204937_s_at	ZNF274	0.0015991	-3.15498
204942_s_at	ALDH3B2	0.0027669	1.94584
204944_at	PTPRG	0.0016838	3.38387

204949_at	ICAM3	0.0149454	1.85488
204959_at	MNDA	0	-28.0466
204962_s_at	CENPA	0.0002237	6.93457
204967_at	SHROOM2	0.0007392	-1.80958
204977_at	DDX10	0.0133062	-2.59491
204978_at	SFRS16	0.0590477	1.41786
204980_at	CLOCK	0.0000404	-2.3258
204984_at	GPC4	0.0000183	-2.14386
204994_at	MX2	0.0069539	3.90392
204998_s_at	ATF5	0.0001652	1.76601
205003_at	DOCK4	0.0000058	-6.69965
205006_s_at	NMT2	0.0009028	-2.54239
205016_at	TGFA	0.0273172	1.85913
205020_s_at	ARL4A	0.0000037	-8.85089
205024_s_at	RAD51	0.0003177	1.66398
205026_at	STAT5B	0.0959995	1.14584
205031_at	EFNB3	0.0000021	-16.3191
205034_at	CCNE2	0.0303488	1.7737
205036_at	LSM6	0.0195639	-2.02998
205042_at	GNE	0.0002276	-2.68579
205044_at	GABRP	0.006147	1.52448
205052_at	AUH	0.0018673	-5.32876
205054_at	NEB	0.0008537	1.59339
205060_at	PARG	0.0381892	1.24566
205061_s_at	EXOSC9	0.0132856	-1.83291
205070_at	ING3	0.0051991	-5.31344
205072_s_at	XRCC4	0.004699	-2.12112
205078_at	PIGF	0.002045	-3.04217
205079_s_at	MPDZ	0.0010957	-1.68623
205080_at	RARB	0.003962	-1.84443
205082_s_at	AOX1	0.0000281	-2.08046
205087_at	RWDD3	0.0263307	-2.11751
205094_at	PEX12	0.0004247	-4.18358
205097_at	SLC26A2	0.0023173	-4.07538
205100_at	GFPT2	0	-14.4014
205104_at	SNPH	0.0808474	1.30888
205110_s_at	FGF13	0	-32.9208
205112_at	PLCE1	0.0012644	-3.16675
205117_at	FGF1	0.0001005	-3.79451
205129_at	NPM3	0.0003055	2.04646

205130_at	RAGE	0.0213732	1.30082
205140_at	FPGT	0.0000647	-4.06056
205141_at	ANG	0.0000368	-1.82241
205151_s_at	TRIL	0.0023974	-2.06798
205158_at	RNASE4	0.0005689	-2.35492
205159_at	CSF2RB	0.0699947	-3.37505
205160_at	PEX11A	0.0001079	-1.73552
205167_s_at	CDC25C	0.0001615	2.25284
205176_s_at	ITGB3BP	0.0358793	-2.11557
205189_s_at	FANCC	0.0204434	1.43171
205198_s_at	ATP7A	0.0000022	-2.86264
205206_at	KAL1	0	-16.6305
205215_at	RNF2	0.006574	1.44812
205226_at	PDGFRL	0.0002257	-2.44111
205234_at	SLC16A4	0.0048251	-2.58112
205235_s_at	KIF20B	0.0012996	1.87424
205242_at	CXCL13	0.0293612	2.57778
205247_at	NOTCH4	0.0057028	1.56857
205251_at	PER2	0.0004779	-2.80969
205256_at	ZBTB39	0.0341873	1.28818
205259_at	NR3C2	0.0000001	-11.0937
205264_at	CD3EAP	0.0125994	1.60885
205267_at	POU2AF1	0.0893755	2.22836
205282_at	LRP8	0.0000005	1.84135
205286_at	TFAP2C	0.0327814	1.79827
205288_at	CDC14A	0.0000038	-5.16606
205290_s_at	BMP2	0.0000954	-5.63685
205292_s_at	HNRNPA2B1	0.0110437	-2.31517
205301_s_at	OGG1	0.0413989	1.24862
205304_s_at	KCNJ8	0	-6.27797
205305_at	FGL1	0.0661321	1.11242
205310_at	FBXO46	0.0002752	2.07356
205313_at	HNF1B	0.0468818	1.60786
205321_at	EIF2S3	0.0056018	-2.21304
205328_at	CLDN10	0.0965565	3.01912
205339_at	STIL	0.0000934	4.60454
205347_s_at	TMSB15A	0.0393809	2.88464
205352_at	SERPINI1	0.000899	-2.45465
205357_s_at	AGTR1	0.0001346	-6.4403
205361_s_at	PFDN4	0.0215539	1.9624

205364_at	ACOX2	0.000002	-2.86532
205366_s_at	HOXB6	0.0086302	1.66116
205372_at	PLAG1	0.000074	5.82819
205376_at	INPP4B	0.0050383	-1.91289
205379_at	CBR3	0.0000042	-2.41879
205394_at	CHEK1	0.0043388	4.02484
205403_at	IL1R2	0.0100071	3.09927
205405_at	SEMA5A	0.0000911	-4.15451
205407_at	RECK	0.0000043	-4.45065
205412_at	ACAT1	0.0000137	-5.32821
205414_s_at	RICH2	0	-7.48817
205428_s_at	CALB2	0	-17.3419
205429_s_at	MPP6	0.0000522	-2.46296
205433_at	BCHE	0	-139.611
205437_at	ZNF211	0.0002467	-2.81442
205440_s_at	NPY1R	0	-14.8846
205441_at	OCEL1	0.0980682	1.36735
205442_at	MFAP3L	0.0022405	-3.25279
205449_at	SAC3D1	0.0008624	3.71799
205452_at	PIGB	0.0000287	-2.35436
205453_at	HOXB2	0.0070041	2.02493
205463_s_at	PDGFA	0.0764304	1.49807
205466_s_at	HS3ST1	0.0001097	-3.06781
205467_at	CASP10	0.0002072	1.37252
205470_s_at	KLK11	0.0000049	-3.83183
205488_at	GZMA	0.0186985	-2.81287
205497_at	ZNF175	0.002584	-1.76639
205498_at	GHR	0	-22.1284
205499_at	SRPX2	0.0328694	1.44462
205501_at	PDE10A	0.000023	-3.19485
205508_at	SCN1B	0.0036112	-2.45596
205509_at	CPB1	0.0000773	-2.48478
205514_at	ZNF415	0.0041738	-3.23328
205515_at	PRSS12	0.002064	1.37983
205522_at	HOXD4///MIR1(	0.0000137	-7.18292
205526_s_at	KATNA1	0.0000057	-3.13107
205528_s_at	RUNX1T1	0.0031019	-1.89061
205530_at	ETFDH	0.0028476	-1.82733
205532_s_at	CDH6	0.0000019	28.981
205541_s_at	GSPT2	0.0000174	-8.83549

205546_s_at	TYK2	0.0037547	1.78435
205547_s_at	TAGLN	0.0017307	1.91393
205560_at	PCSK5	0.0066222	-1.75578
205568_at	AQP9	0	-79.2607
205571_at	LIPT1	0.0053474	-3.21826
205573_s_at	SNX7	0.0000098	-12.5895
205601_s_at	HOXB5	0.0009743	1.74207
205632_s_at	PIP5K1B	0.0000021	-6.37258
205636_at	SH3GL3	0.0262448	1.70901
205638_at	BAI3	0.0002625	-2.13268
205647_at	RAD52	0.0073582	1.83571
205654_at	C4BPA	0.0039441	-7.07495
205656_at	PCDH17	0.000218	-9.7733
205661_s_at	FLAD1	0.0041667	1.29398
205662_at	B9D1	0.0432206	1.40927
205672_at	XPA	0.0001006	-4.13483
205673_s_at	ASB9	0.0038152	1.54941
205684_s_at	DENND4C	0.0000166	-5.04358
205694_at	TYRP1	0.0288929	2.73816
205698_s_at	MAP2K6	0.0105412	1.73938
205700_at	HSD17B6	0.0034599	-2.75231
205710_at	LRP2	0.0000066	-1.98617
205715_at	BST1	0.000001	-2.53989
205726_at	DIAPH2	0.0000196	-5.13366
205728_at	ODZ1	0.0513236	-2.31732
205733_at	BLM	0.0001091	3.36084
205741_s_at	DTNA	0.0000764	-3.43823
205750_at	BPHL	0.0081478	1.34483
205751_at	SH3GL2	0.0002268	-2.96409
205752_s_at	GSTM5	0.0145782	-2.19513
205768_s_at	SLC27A2	0.0791598	1.30156
205771_s_at	AKAP7	0.000115	-4.69111
205780_at	BIK	0.0000469	4.2796
205786_s_at	ITGAM	0.0069152	-2.23562
205795_at	NRXN3	0.0002605	-4.82016
205802_at	TRPC1	0.0001693	-4.79541
205816_at	ITGB8	0.036766	2.11062
205819_at	MARCO	0.0017774	-2.31637
205836_s_at	YTHDC2	0.0520748	1.35208
205849_s_at	UQCRB	0.0012909	-1.97318

205862_at	GREB1	0.0078259	-7.10311
205880_at	PRKD1	0.0107612	-2.20353
205898_at	CX3CR1	0.0043908	-2.21438
205907_s_at	OMD	0.0000004	-16.817
205932_s_at	MSX1	0.033488	5.25432
205934_at	PLCL1	0.0008713	-3.51784
205961_s_at	PSIP1	0.0019925	-4.70968
205964_at	ZNF426	0.0003226	-1.75007
205969_at	AADAC	0.0037851	-2.48252
205979_at	SCGB2A1	0.0004886	15.8021
205981_s_at	ING2	0.0071921	-1.76304
205992_s_at	IL15	0.0005858	-4.20004
206001_at	NPY	0.0129737	-1.88597
206007_at	PRG4	0	-48.7353
206011_at	CASP1	0.0165892	-2.17282
206033_s_at	DSC3	0.0001257	-2.88812
206038_s_at	NR2C2	0.0632445	1.25888
206039_at	RAB33A	0.0000046	-5.97697
206044_s_at	BRAF	0.0091978	1.37021
206052_s_at	SLBP	0.0095158	-3.41839
206055_s_at	SNRPA1	0.0162664	1.92164
206059_at	ZNF91	0.0003972	-4.5597
206067_s_at	WT1	0.0014587	-3.43204
206074_s_at	HMGA1	0	10.8859
206082_at	HCP5	0.000036	-3.77548
206090_s_at	DISC1///TSNAX	0.0012676	-1.70481
206101_at	ECM2	0.0000004	-19.4071
206102_at	GINS1	0.0000084	6.20101
206110_at	HIST1H3H	0.0261178	1.84729
206117_at	TPM1	0.0180227	2.60644
206123_at	LLGL1	0.0145898	1.28861
206125_s_at	KLK8	0.0000045	2.41285
206128_at	ADRA2C	0.0003346	2.89654
206140_at	LHX2	0	-259.058
206157_at	PTX3	0.0008862	7.45417
206167_s_at	ARHGAP6	0.0001366	-3.01513
206177_s_at	ARG1	0.0002089	-2.07273
206201_s_at	MEOX2	0.0031485	-5.41798
206228_at	PAX2	0.0697991	2.43823
206230_at	LHX1	0.002448	1.84335

206236_at	GPR4	0.0063379	1.6729
206245_s_at	IVNS1ABP	0.0276745	-2.01292
206247_at	MICB	0.0418437	2.36739
206262_at	ADH1C	0.0000029	-8.9449
206263_at	FMO4	0.0014915	-2.1468
206271_at	TLR3	0.0231543	-2.33386
206272_at	RAB4A///SPHA	0.0049537	-3.10013
206286_s_at	TDGF1///TDGF:	0.0370705	-3.31721
206295_at	IL18	0.0000022	-11.8457
206297_at	CTRC	0.0291999	1.56872
206299_at	FAM155B	0.0067891	1.47942
206331_at	CALCRL	0.0001828	-2.49479
206336_at	CXCL6	0.0036218	-3.62493
206363_at	MAF	0	-9.35847
206371_at	FOLR3	0.0037419	2.46426
206373_at	ZIC1	0.0008602	23.6207
206375_s_at	HSPB3	0.000003	-5.50594
206381_at	SCN2A	0.0591722	1.23789
206391_at	RARRES1	0.0000024	-7.51034
206414_s_at	ASAP2	0.0854778	1.54217
206415_at	TLL1	0.000048	-2.48583
206435_at	B4GALNT1	0.0003447	1.42619
206451_at	TBCCD1	0.0138509	1.88793
206458_s_at	WNT2B	0	-51.758
206499_s_at	RCC1///SNHG3	0.0053937	1.52013
206504_at	CYP24A1	0.0368247	1.27474
206508_at	CD70	0.0719021	1.21769
206542_s_at	SMARCA2	0.0000466	-8.79189
206545_at	CD28	0.0004559	-2.06625
206567_s_at	PHF20	0.00466	-1.76523
206581_at	BNC1	0.0000004	-30.4402
206584_at	LY96	0.0239092	-1.93544
206593_s_at	MED22	0.0370658	1.20627
206600_s_at	SLC16A5	0.0000335	-2.90203
206601_s_at	HOXD3	0.0000348	-6.52396
206613_s_at	TAF1A	0.0023483	-2.01766
206621_s_at	EIF4H	0.0054434	-2.15906
206645_s_at	NR0B1	0.0000004	-6.21725
206648_at	ZNF571	0.0004649	-3.16109
206654_s_at	POLR3G	0.0807889	1.43579

206658_at	UPK3B	0.0033341	-4.43872
206686_at	PDK1	0.0027963	1.71471
206687_s_at	PTPN6	0.0280762	1.76867
206688_s_at	CPSF4	0.038983	1.44886
206698_at	XK	0.0272642	2.6268
206702_at	TEK	0.0011786	-2.05303
206723_s_at	LPAR2	0.0023807	1.9096
206734_at	JRKL	0.0118007	1.57906
206737_at	WNT11	0.0070432	3.25437
206754_s_at	CYP2B6///CYP:	0.0110967	-2.39595
206765_at	KCNJ2	0.0000022	-5.11032
206772_at	PTH2R	0.0098485	5.50532
206799_at	SCGB1D2	0.0046274	5.99047
206809_s_at	HNRNPA3///HN	0.0005175	-2.87269
206822_s_at	L3MBTL	0.0003075	1.64266
206825_at	OXTR	0.0024926	1.92075
206826_at	PMP2	0.003933	1.41767
206838_at	TBX19	0.0313941	1.45092
206854_s_at	MAP3K7	0.0566626	-2.34443
206858_s_at	HOXC6	0.0000007	-5.72071
206864_s_at	HRK	0.0038809	1.50719
206875_s_at	SLK	0.0000631	-4.53059
206884_s_at	SCEL	0.0506494	1.46524
206898_at	CDH19	0.0003866	-2.19303
206904_at	MATN1	0.0345894	1.55018
206918_s_at	CPNE1	0.0025127	1.87853
206949_s_at	RUSC1	0.000017	3.30801
206953_s_at	LPHN2	0.0055541	-3.21434
206954_at	WIT1	0.0001051	-4.17232
206958_s_at	UPF3A	0.0015082	-3.74527
206991_s_at	CCR5	0.0368221	-2.17654
206993_at	ATP5S	0.0063629	2.54719
207002_s_at	PLAGL1	0.0157646	-1.94008
207010_at	GABRB1	0.0002279	-2.14845
207013_s_at	MMP16	0.0272662	1.67435
207016_s_at	ALDH1A2	0	-360.546
207030_s_at	CSRP2	0.0020547	2.86959
207039_at	CDKN2A	0.0005543	17.2937
207057_at	SLC16A7	0.0028603	-4.2834
207064_s_at	AOC2	0.0595849	1.16711

207069_s_at	SMAD6	0.00001	2.72071
207071_s_at	ACO1	0.0011213	-2.90635
207076_s_at	ASS1	0.0001666	5.62379
207078_at	MED6	0.0222475	-2.2342
207109_at	POU2F3	0.0048345	3.1377
207124_s_at	GNB5	0.0000875	-2.41309
207142_at	KCNJ3	0.0502878	1.16754
207156_at	HIST1H2AG	0.047859	1.5546
207165_at	HMMR	0.0000509	12.8049
207183_at	GPR19	0.0155365	1.65937
207191_s_at	ISLR	0.0246627	1.34353
207228_at	PRKACG	0.0968455	1.23855
207245_at	UGT2B17	0.0717102	1.92686
207251_at	MEP1B	0.0097099	1.3274
207264_at	KDELR3	0.0055509	1.55576
207267_s_at	DSCR6	0.0156326	1.34433
207283_at	RPL23AP32	0.0000003	-4.53918
207302_at	SGCG	0	-37.401
207305_s_at	KIAA1012	0.0022142	-4.98847
207309_at	NOS1	0.0005797	1.38258
207332_s_at	TFRC	0.0001828	-7.44281
207338_s_at	ZNF200	0.0134849	-1.90123
207339_s_at	LTB	0.0177286	1.83115
207363_at	RS1	0.0468675	1.63692
207386_at	CYP7B1	0.0341802	1.16511
207389_at	GP1BA	0.030165	1.26163
207391_s_at	PIP5K1A	0.005613	1.29842
207405_s_at	RAD17	0.0002023	-1.95366
207431_s_at	DEGS1	0.0161179	-1.9323
207435_s_at	SRRM2	0.0047064	1.73184
207438_s_at	SNUPN	0.0000978	-2.31955
207480_s_at	MEIS2	0	-22.8197
207483_s_at	CAND1	0.0108553	-2.65844
207498_s_at	CYP2D6	0.0476487	1.48221
207513_s_at	ZNF189	0.0003135	-2.31796
207525_s_at	GIPC1	0.0010529	1.73277
207543_s_at	P4HA1	0.0762084	1.62477
207545_s_at	NUMB	0.0015929	-2.09491
207563_s_at	OGT	0.0093697	1.28764
207582_at	PIN1L	0.0019683	1.46616

207585_s_at	RPL36AL	0.000045	-4.3402
207593_at	ABCG4	0.0446348	1.2833
207606_s_at	ARHGAP12	0.0174871	-1.94216
207614_s_at	CUL1	0.0040359	-2.48326
207625_s_at	CBFA2T2	0.0003498	1.62928
207642_at	HCRT	0.0076181	1.32777
207687_at	INHBC	0.0177741	1.13597
207689_at	TBX10	0.0725377	1.29735
207699_at	ZFHX2	0.0109106	1.62771
207717_s_at	PKP2	0.0046789	-3.62601
207734_at	LAX1	0.0198871	1.28889
207761_s_at	METTL7A	0	-14.6267
207785_s_at	RBPJ	0.0000735	-3.49573
207788_s_at	SORBS3	0.0792509	1.56175
207804_s_at	FCN2	0.026415	1.54865
207808_s_at	PROS1	0	-13.1937
207811_at	KRT12	0.0054631	1.30749
207830_s_at	PPP1R8	0.0000618	-2.84557
207839_s_at	TMEM8B	0.0000031	-4.17867
207845_s_at	ANAPC10	0.0055946	-2.73704
207850_at	CXCL3	0.0009768	2.02273
207872_s_at	LILRA1	0.0865748	1.44016
207876_s_at	FLNC	0.0073124	-3.47816
207919_at	ART1	0.0378357	1.18901
207922_s_at	MAEA	0.0007307	-1.67086
207971_s_at	CEP68	0.0263588	1.35228
207983_s_at	STAG2	0.0012539	-1.68791
208002_s_at	ACOT7	0.084992	1.49956
208017_s_at	MCF2	0.006633	-1.8823
208021_s_at	RFC1	0.0000738	-2.84072
208025_s_at	HMGA2	0.0004439	5.93385
208035_at	GRM6	0.041164	1.17513
208051_s_at	PAIP1	0.0331013	1.54802
208064_s_at	ST8SIA3	0.0835218	1.22999
208066_s_at	GTF2B	0.0007373	-3.09741
208070_s_at	REV3L	0.0231012	-2.78244
208072_s_at	DGKD	0.0005526	1.60063
208079_s_at	AURKA	0	16.9319
208089_s_at	TDRD3	0.0000418	-2.36662
208096_s_at	COL21A1	0.0000482	-2.69041

208103_s_at	ANP32E	0.0111804	2.89927
208109_s_at	C15orf5	0.0024836	-2.89835
208119_s_at	ZNF93	0.0399958	1.46672
208131_s_at	PTGIS	0.0000076	-19.8771
208138_at	GAST	0.0111497	1.42438
208140_s_at	LRRC48	0.0006895	-1.68573
208146_s_at	CPVL	0.0000003	-7.85708
208161_s_at	ABCC3	0.0770605	1.82593
208165_s_at	PRSS16	0.047267	-2.2968
208217_at	GABRR2	0.0003157	1.29707
208228_s_at	FGFR2	0.0048067	3.642
208249_s_at	TGDS	0.0008538	-2.01323
208264_s_at	EIF3J	0.0002849	-2.51187
208290_s_at	EIF5	0.0004697	-3.4893
208305_at	PGR	0.0033854	-2.14051
208319_s_at	RBM3	0.0032881	-4.06376
208328_s_at	MEF2A	0.0226651	-2.16493
208331_at	BPY2	0.0426397	1.18009
208369_s_at	GCDH	0.0034067	1.43617
208383_s_at	PCK1	0.0000887	-4.19448
208393_s_at	RAD50	0.0015258	-3.06937
208422_at	MSR1	0.0325138	1.18185
208446_s_at	ZFYVE9	0.0479002	1.24187
208450_at	LGALS2	0	-24.4034
208451_s_at	C4A///C4B///LC	0.007643	-2.7954
208470_s_at	HP///HPR	0.0646227	-2.43838
208511_at	PTTG3P	0.0000995	2.12847
208569_at	HIST1H2AB	0.0081099	1.57235
208614_s_at	FLNB	0.0003161	-2.09081
208615_s_at	PTP4A2	0.010528	-2.48325
208620_at	PCBP1	0.0118175	-2.61882
208625_s_at	EIF4G1	0.0359401	1.61016
208632_at	RNF10	0.0001911	-2.47622
208634_s_at	MACF1	0.0000782	-3.09785
208636_at	ACTN1	0.000011	2.99117
208641_s_at	RAC1	0.0000036	-2.75566
208643_s_at	XRCC5	0.0008184	-2.36797
208644_at	PARP1	0.0352095	1.41484
208650_s_at	CD24	0.0000054	71.9447
208653_s_at	CD164	0.0997485	1.89867

208656_s_at	CCNI	0.0000009	-9.04367
208658_at	PDIA4	0.0001512	3.00915
208663_s_at	TTC3	0.0220338	-2.49767
208666_s_at	ST13	0.0123473	-3.26967
208671_at	SERINC1	0.0000068	-12.1434
208678_at	ATP6V1E1	0.0001465	-2.59927
208679_s_at	ARPC2	0.000228	-7.97365
208680_at	PRDX1	0.0021825	-4.44455
208683_at	CAPN2	0.0000286	-5.25276
208693_s_at	GARS	0.0129368	-2.6723
208694_at	PRKDC	0.0977546	1.49684
208697_s_at	EIF3E	0.0002993	-3.18403
208698_s_at	NONO	0.0003281	2.70446
208712_at	CCND1	0.0000322	7.55021
208714_at	NDUFV1	0.0035473	1.6652
208717_at	OXA1L	0.0028403	-1.93612
208726_s_at	EIF2S2	0.0110408	-2.81175
208732_at	RAB2A	0.0033941	-2.33329
208737_at	ATP6V1G1	0.0017323	-2.73599
208742_s_at	SAP18	0.0006468	-2.63817
208745_at	ATP5L	0.0001384	-4.0694
208747_s_at	C1S	0.0003154	-4.37286
208756_at	EIF3I	0.0003862	-3.74488
208758_at	ATIC	0.0048703	-1.7559
208760_at	UBE2I	0.0000167	-5.03031
208771_s_at	LTA4H	0.0001077	-3.51734
208773_s_at	ANKHD1///ANK	0.0009126	-3.10867
208776_at	PSMD11	0.000012	-1.87509
208778_s_at	TCP1	0.0006067	-3.98691
208783_s_at	CD46	0.0007743	-4.55231
208785_s_at	MAP1LC3B	0.003951	-2.29504
208787_at	MRPL3	0.0957365	1.52325
208792_s_at	CLU	0.0128181	1.71694
208795_s_at	MCM7	0.0007412	2.8672
208796_s_at	CCNG1	0.0002336	-4.3171
208797_s_at	GOLGA8A	0.0270591	1.3944
208801_at	SRP72	0.0074775	-1.84257
208804_s_at	SFRS6	0.000127	-2.01739
208810_at	DNAJB6///TME	0.001073	-4.71693
208813_at	GOT1	0.0884942	1.39672

208814_at	HSPA4	0.0048298	-3.1579
208821_at	SNRPB	0.0571031	1.61773
208832_at	ATXN10	0.000011	-1.82979
208835_s_at	LUC7L3	0.0047352	-4.37129
208841_s_at	G3BP2	0.0093038	-3.40348
208847_s_at	ADH5	0.0005998	-1.97006
208850_s_at	THY1	0.0390776	1.68024
208860_s_at	ATRX	0.0042682	-2.02152
208862_s_at	CTNND1	0.0441802	1.29675
208863_s_at	SFRS1	0.0068932	-2.24124
208869_s_at	GABARAPL1	0.0000003	-3.43023
208873_s_at	REEP5	0.0000265	-3.84011
208875_s_at	PAK2	0.0037476	2.49
208886_at	H1F0	0.0439952	2.55207
208894_at	HLA-DRA	0.0389423	-2.28295
208896_at	DDX18	0.0185307	-2.15573
208898_at	ATP6V1D	0.0000165	-3.67182
208910_s_at	C1QBP	0.0081398	-3.52701
208911_s_at	PDHB	0.0007491	-3.07153
208912_s_at	CNP	0.0013555	1.8275
208913_at	GGA2	0.0770733	1.36208
208918_s_at	NADK	0.0794241	2.06717
208921_s_at	SRI	0.002913	-3.15402
208923_at	CYFIP1	0.0025537	-1.82917
208924_at	RNF11	0.0006718	-6.3336
208925_at	CLDND1	0.001786	-2.04946
208934_s_at	LGALS8	0.0000016	-5.19525
208944_at	TGFBR2	0.0000062	-7.47707
208945_s_at	BECN1	0.0164298	-1.89788
208949_s_at	LGALS3	0.0000003	-4.70558
208952_s_at	LARP4B	0.0037434	1.91846
208955_at	DUT	0.0135844	-2.52956
208959_s_at	ERP44	0.0070831	-2.93351
208965_s_at	IFI16	0.0017713	-4.86444
208980_s_at	UBC	0.0000434	-2.80352
208982_at	PECAM1	0.0009009	4.02645
208989_s_at	KDM2A	0.0010964	1.49725
208990_s_at	HNRNPH3	0.0016246	-3.84117
208991_at	STAT3	0.0032482	-3.49167
208993_s_at	PPIG	0.0064909	-3.86721

208998_at	UCP2	0.0000011	7.08299
208999_at	Sep 8, 2013	0.0689021	1.38171
209001_s_at	ANAPC13	0.0000986	-2.26765
209005_at	FBXL5	0.0000094	-5.68214
209006_s_at	C1orf63	0.0055931	-2.59101
209016_s_at	KRT7	0.064161	1.50771
209019_s_at	PINK1	0.0005773	-1.84752
209030_s_at	CADM1	0.0050675	-2.13222
209034_at	PNRC1	0.0000062	-4.87361
209035_at	MDK	0.0009495	1.63308
209040_s_at	PSMB8	0.0033552	-3.57932
209043_at	PAPSS1	0.0024151	-3.21882
209046_s_at	GABARAPL2	0.0000103	-3.95764
209047_at	AQP1	0.0001974	-1.95223
209048_s_at	ZMYND8	0.0236518	1.64848
209053_s_at	WHSC1	0.0001591	2.44917
209067_s_at	HNRPDL	0.001487	-2.40053
209069_s_at	H3F3B	0.0042544	-2.10882
209074_s_at	FAM107A	0.0057749	2.55007
209075_s_at	ISCU	0.0001156	-2.39707
209082_s_at	COL18A1	0.0030418	2.5494
209091_s_at	SH3GLB1	0.0005775	-4.24518
209092_s_at	GLOD4	0.0013738	-2.97093
209095_at	DLD	0.0030795	-4.03287
209102_s_at	HBP1	0.0000165	-3.54825
209103_s_at	UFD1L	0.0045584	-1.95039
209111_at	RNF5	0.0749057	1.5198
209112_at	CDKN1B	0.0005906	-4.39669
209122_at	PLIN2	0.0000483	-3.57321
209127_s_at	SART3	0.0414016	-2.35559
209134_s_at	RPS6	0.0005995	-2.90013
209141_at	UBE2G1	0.0026795	-2.63855
209146_at	SC4MOL	0.00413	-2.58005
209147_s_at	PPAP2A	0	-15.2058
209148_at	RXRB	0.0365686	1.35309
209153_s_at	TCF3	0.0755977	1.43472
209161_at	PRPF4	0.0015199	-2.64481
209170_s_at	GPM6B	0.0034399	3.38399
209172_s_at	CENPF	0	18.9076
209175_at	SEC23IP	0.0015078	-3.57797

209180_at	RABGGTB	0.002794	-3.48583
209186_at	ATP2A2	0.0339255	1.81396
209190_s_at	DIAPH1	0.000549	1.77773
209191_at	TUBB6	0.0102802	-2.2475
209194_at	CETN2	0.0000288	-2.71433
209198_s_at	SYT11	0.0000264	3.71569
209199_s_at	MEF2C	0.0000013	-4.36241
209210_s_at	FERMT2	0.0005026	-4.93229
209218_at	SQLE	0.0483693	-2.54383
209219_at	RDBP	0.0014125	-3.14113
209229_s_at	SAPS1	0.0007158	1.57205
209231_s_at	DCTN5	0.0143886	1.37806
209234_at	KIF1B	0.010774	-2.10576
209239_at	NFKB1	0.0011616	-1.66432
209242_at	PEG3	0.0069146	-6.28574
209243_s_at	PEG3///ZIM2	0.0126372	-2.46754
209247_s_at	ABCF2	0.0137425	1.4634
209248_at	GHITM	0.0001412	-2.79031
209254_at	KLHDC10	0.0952657	1.25055
209259_s_at	SMC3	0.0061811	-2.27005
209268_at	VPS45	0.0454768	1.30754
209271_at	BPTF	0.0040501	-2.1958
209273_s_at	ISCA1	0.0522841	1.44139
209277_at	TFPI2	0.0000023	-37.0229
209280_at	MRC2	0.0897347	1.41021
209285_s_at	C3orf63	0.0001466	-3.21539
209288_s_at	CDC42EP3	0.000646	-3.22685
209291_at	ID4	0.0144063	-2.28666
209298_s_at	ITSN1	0.0030124	-1.88187
209300_s_at	NECAP1	0.0041111	-2.44159
209302_at	POLR2H	0.0122666	2.24034
209303_at	NDUFS4	0.000319	-3.25515
209307_at	SWAP70	0.0000739	-3.89859
209308_s_at	BNIP2	0.0005982	-3.02876
209310_s_at	CASP4	0.0002004	-3.40329
209314_s_at	HBS1L	0.0075972	-2.43561
209323_at	PRKRIR	0.0289055	-2.14281
209326_at	SLC35A2	0.0663562	1.53968
209335_at	DCN	0.0000464	-5.1888
209340_at	UAP1	0.0000002	-6.20628

209354_at	TNFRSF14	0.0371383	1.45407
209358_at	TAF11	0.0335614	1.41849
209360_s_at	RUNX1	0.0114138	2.20421
209361_s_at	PCBP4	0.003078	1.58648
209362_at	MED21	0.0000274	-7.39704
209369_at	ANXA3	0.0000035	-10.3174
209375_at	XPC	0.0000034	-1.97071
209379_s_at	FAM190B	0.0000016	-4.13892
209385_s_at	PROSC	0.0001461	-5.61648
209386_at	TM4SF1	0.0067144	-4.1996
209395_at	CHI3L1	0.0146159	2.16912
209397_at	ME2	0.0052027	-3.07176
209398_at	HIST1H1C	0.0955758	1.80426
209408_at	KIF2C	0.0000005	6.06544
209409_at	GRB10	0.0016183	-2.09673
209418_s_at	THOC5	0.052487	1.40005
209425_at	AMACR///C1QT	0.0000058	-5.266
209430_at	BTAF1	0.0005735	-2.32271
209431_s_at	PATZ1	0.0678678	1.35811
209435_s_at	ARHGEF2	0.00114	2.45865
209440_at	PRPS1	0.0000809	-2.89045
209451_at	TANK	0.0137423	-2.82971
209452_s_at	VTI1B	0.000811	-2.13993
209454_s_at	TEAD3	0.0116054	1.51631
209455_at	FBXW11	0.0011928	-2.17217
209470_s_at	GPM6A	0.0002383	-5.43199
209471_s_at	FNTA	0.0129897	-2.00608
209472_at	CCBL2	0.0057496	-2.04598
209479_at	CCDC28A	0.0000008	-6.83407
209481_at	SNRK	0.0006548	-2.53162
209482_at	POP7	0.0447879	1.35456
209485_s_at	OSBPL1A	0.0006976	-5.33732
209486_at	UTP3	0.0043115	-1.84421
209501_at	CDR2	0.0019383	-2.6677
209503_s_at	PSMC5	0.0009155	-2.26878
209505_at	NR2F1	0.0021449	-2.36235
209510_at	RNF139	0.0091988	-2.75552
209512_at	HSDL2	0.0000949	-2.90901
209517_s_at	ASH2L	0.0000144	-3.33024
209518_at	SMARCD1	0.0023366	1.86581

209528_s_at	TELO2	0.0131212	1.94204
209537_at	EXTL2	0.0005588	-2.83697
209538_at	ZNF32	0.0000003	-5.09943
209547_s_at	SF4	0.0808276	1.29188
209549_s_at	DGUOK	0.0307426	1.38349
209550_at	NDN	0.000003	-12.7709
209558_s_at	HIP1R///LOC10	0.0006494	2.21648
209565_at	RNF113A	0.0034352	-1.74098
209568_s_at	RGL1	0.0000215	-4.49069
209570_s_at	D4S234E///FOX	0.0000505	-6.29668
209572_s_at	EED	0.0147244	-2.33427
209575_at	IL10RB	0.000008	-2.62464
209576_at	GNAI1	0.0000717	-3.74944
209580_s_at	MBD4	0.0099241	-3.14718
209581_at	PLA2G16	0.0034756	-1.90314
209583_s_at	CD200	0.0039175	-2.49324
209585_s_at	MINPP1	0.0000581	-3.36979
209589_s_at	EPHB2	0.0003538	1.45655
209598_at	PNMA2	0.0067028	-2.95081
209605_at	TST	0.0046403	-2.0756
209614_at	ADH1B	0.0033768	-2.14006
209620_s_at	ABCB7	0.00018	-2.33783
209625_at	PIGH	0.0000019	-1.84482
209626_s_at	OSBPL3	0.0136219	2.37679
209635_at	AP1S1	0.0018559	2.31675
209639_s_at	RGS12	0.0002647	1.49435
209643_s_at	PLD2	0.0961355	1.3834
209647_s_at	SOCS5	0.000768	-4.28069
209649_at	STAM2	0.0106543	-2.4899
209655_s_at	TMEM47	0.0118185	-2.08417
209657_s_at	HSF2	0.0000402	-2.12129
209659_s_at	CDC16	0.001366	-2.4998
209662_at	CETN3	0.0054395	-2.55718
209666_s_at	CHUK	0.039577	-2.32665
209667_at	CES2	0.0018895	-2.84137
209672_s_at	MIOS	0.0044877	-1.97479
209674_at	CRY1	0.0069566	-1.77007
209682_at	CBLB	0.0640519	1.28118
209684_at	RIN2	0.0127819	1.54691
209688_s_at	CCDC93	0.0116137	-2.31471

209692_at	EYA2	0.0050953	2.16409
209695_at	PTP4A3	0.0096148	2.9283
209696_at	FBP1	0.0014281	-1.79352
209702_at	FTO	0.0000042	-3.29628
209706_at	NKX3-1	0	-20.5675
209710_at	GATA2	0.0272373	1.88532
209714_s_at	CDKN3	0.000403	3.37318
209716_at	CSF1	0.0522244	1.22415
209717_at	EVI5	0.0001505	-2.84929
209721_s_at	IFFO1	0.0002586	-2.03923
209723_at	SERPINB9	0.0013717	-3.2609
209724_s_at	ZFP161	0.0011718	-1.90724
209729_at	GAS2L1	0.0199228	1.2023
209732_at	CLEC2B	0.0058382	-3.1975
209733_at	MID2	0.0000391	-1.70451
209735_at	ABCG2	0.0003565	-2.5498
209736_at	SOX13	0.0006231	1.82072
209737_at	MAGI2	0.0000059	-5.19262
209740_s_at	PNPLA4	0.0000066	-6.53677
209745_at	COQ7	0.0045743	-1.7563
209753_s_at	TMPO	0.0436427	1.56027
209757_s_at	MYCN	0.0860844	2.69159
209763_at	CHRDL1	0	-159.489
209787_s_at	HMGN4	0.0009373	-1.91177
209790_s_at	CASP6	0.0151518	-2.45794
209794_at	SRGAP3	0.0020058	2.12481
209797_at	CNPY2	0.0012787	2.40693
209806_at	HIST1H2BK	0.0003448	3.28077
209814_at	ZNF330	0.0000704	-5.36673
209822_s_at	VLDLR	0.0000051	-12.6421
209824_s_at	ARNTL	0.0006347	-1.75842
209828_s_at	IL16	0.0000008	-3.60558
209829_at	FAM65B	0.0000027	-4.8248
209841_s_at	LRRN3	0.0004289	-5.30804
209849_s_at	RAD51C	0.0000799	-3.57114
209860_s_at	ANXA7	0.0126516	-2.19711
209864_at	FRAT2	0.0188611	1.34111
209865_at	SLC35A3	0.0010535	-2.38624
209869_at	ADRA2A	0.0033595	-3.36761
209871_s_at	APBA2	0.0000484	2.10397

209873_s_at	PKP3	0.0004164	1.52235
209879_at	SELPLG	0.059507	1.57109
209883_at	GLT25D2	0.005548	-1.93355
209884_s_at	SLC4A7	0.0031318	2.82017
209891_at	SPC25	0.0019056	2.98894
209894_at	LEPR	0.0059723	-3.88569
209897_s_at	SLIT2	0.0000032	-2.10854
209899_s_at	PUF60	0.0004532	3.28153
209906_at	C3AR1	0.0069987	-2.41055
209910_at	SLC25A16	0.008585	1.27408
209921_at	SLC7A11	0.0505314	2.15691
209935_at	ATP2C1	0.0087395	-2.54317
209938_at	TADA2A	0.0161563	1.20111
209940_at	PARP3	0.0442998	1.47069
209943_at	FBXL4	0.0050264	-2.82979
209972_s_at	AIMP2	0.0001597	1.70663
209975_at	CYP2E1	0.0024754	1.8443
209988_s_at	ASCL1	0.0061168	-4.02806
210001_s_at	SOCS1	0.0232391	1.49252
210007_s_at	GPD2	0.0483644	1.45197
210026_s_at	CARD10	0.0398825	2.46807
210034_s_at	RPL5	0.0823335	1.19022
210046_s_at	IDH2	0.0017242	1.91813
210052_s_at	TPX2	0.001418	3.04821
210059_s_at	MAPK13	0.0009516	2.16998
210064_s_at	UPK1B	0.0105982	-2.41611
210069_at	CHKB-CPT1B//	0.0174051	2.87833
210072_at	CCL19	0.0193481	1.26708
210078_s_at	KCNAB1	0.0083287	-1.97177
210096_at	CYP4B1	0.0472638	-4.86512
210105_s_at	FYN	0.0030899	-2.01827
210121_at	B3GALT2	0.0000292	-2.04821
210125_s_at	BANF1	0.0501397	1.30611
210135_s_at	SHOX2	0.0031727	-2.06234
210137_s_at	DCTD	0.0014859	-2.16128
210139_s_at	PMP22	0.0000002	-12.274
210145_at	PLA2G4A	0.000001	-5.86787
210150_s_at	LAMA5	0.0000604	2.6691
210152_at	LILRB4	0.0517132	1.66842
210156_s_at	PCMT1	0.0009403	-1.75624

210172_at	SF1	0.0457248	-2.27905
210176_at	TLR1	0.0220252	-2.1628
210187_at	FKBP1A	0.0043163	1.59618
210200_at	WWP2	0.0499298	1.1597
210213_s_at	EIF6	0.0002011	2.44068
210222_s_at	RTN1	0.0054915	-2.55744
210239_at	IRX5	0.000441	-3.00903
210243_s_at	B4GALT3	0.0016622	1.86331
210249_s_at	NCOA1	0.0000436	-2.88155
210251_s_at	RUFY3	0.0151381	-1.87833
210253_at	HTATIP2	0.0565536	2.09253
210260_s_at	TNFAIP8	0.000286	-5.19049
210275_s_at	ZFAND5	0.0164906	-2.20753
210277_at	AP4S1	0.0085761	-2.30669
210296_s_at	PEX2	0.0018394	-6.97473
210299_s_at	FHL1	0.0003318	-2.04869
210300_at	REM1	0.0247557	1.41546
210312_s_at	IFT20	0.0018797	-2.19769
210313_at	LILRA4	0.0275766	2.31438
210316_at	FLT4	0.0092491	1.46635
210338_s_at	HSPA8	0.0002257	-5.40197
210347_s_at	BCL11A	0.0056808	3.32536
210355_at	PTHLH	0.0003012	-3.40502
210372_s_at	TPD52L1	0.0000005	-5.57189
210380_s_at	CACNA1G	0.0323056	1.2177
210386_s_at	MTX1	0.0012401	2.63861
210396_s_at	BOLA2///LOC4	0.0121454	2.02666
210397_at	DEFB1	0.0124119	3.8638
210407_at	PPM1A	0.024014	-1.95718
210428_s_at	HGS	0.0040555	1.36701
210445_at	FABP6	0.0138135	2.78125
210465_s_at	SNAPC3	0.0908475	1.30689
210466_s_at	SERBP1	0.0913568	1.54734
210473_s_at	GPR125	0.0000954	-2.36371
210479_s_at	RORA	0.0018036	-3.96924
210481_s_at	CLEC4M	0.0001265	-7.23823
210484_s_at	MGC31957///TI	0.0064144	1.50579
210499_s_at	PQBP1	0.0079586	1.55579
210503_at	MAGEA11	0.0139812	2.01271
210511_s_at	INHBA	0.0149964	1.58579

210512_s_at	VEGFA	0.0000542	5.53667
210519_s_at	NQO1	0.0043375	-1.98985
210530_s_at	NR2C1	0.0126057	2.21689
210538_s_at	BIRC3	0.0016514	-2.71371
210552_s_at	RALGPS1	0.0041246	1.98148
210554_s_at	CTBP2	0.003282	1.51559
210561_s_at	WSB1	0.0010683	-4.19575
210589_s_at	GBAP1	0.0214222	1.68349
210608_s_at	FUT2	0.0812875	1.17296
210609_s_at	TP53I3	0.0098777	1.88321
210621_s_at	RASA1	0.0012042	-3.32629
210623_at	UBXN1	0.0104549	1.52914
210652_s_at	TTC39A	0.0032412	2.1848
210665_at	TFPI	0.0002599	-2.7766
210670_at	PPY	0.0310328	1.70894
210674_s_at	PCDHA1///PCD	0.0003641	-4.34584
210715_s_at	SPINT2	0.0044852	1.97541
210719_s_at	HMG20B	0.0887735	1.31684
210720_s_at	NECAB3	0.0001753	1.83318
210721_s_at	PAK7	0.0001357	-1.70118
210746_s_at	EPB42	0.0000006	-6.00828
210751_s_at	RGN	0.0004166	-2.90031
210759_s_at	PSMA1	0.0000875	-4.91096
210762_s_at	DLC1	0.0011288	-2.76463
210774_s_at	NCOA4	0.000613	-2.27168
210786_s_at	FLI1	0.0303937	1.12431
210788_s_at	DHRS7	0.0000017	-6.28338
210797_s_at	OASL	0.0937193	1.12358
210811_s_at	DDX49	0.0759699	1.42009
210889_s_at	FCGR2B	0.0397496	-2.23748
210892_s_at	GTF2I	0.0546457	1.15892
210896_s_at	ASPH	0.0043135	-2.17659
210929_s_at	AHSG///LOC10	0.002433	1.6266
210933_s_at	FSCN1	0.0089042	1.90484
210944_s_at	CAPN3	0.0713891	1.52154
210962_s_at	AKAP9	0.0001148	-2.97154
210970_s_at	IBTK	0.002203	-2.60402
210976_s_at	PFKM	0.0000392	-2.6827
210981_s_at	GRK6	0.000543	2.13854
211006_s_at	KCNB1	0.0013609	-3.46106

211009_s_at	ZNF271	0.0018549	-2.74369
211033_s_at	PEX7	0.0276703	-2.02791
211071_s_at	MLLT11	0.0223126	2.02268
211081_s_at	MAP4K5	0.011472	-2.4741
211090_s_at	PRPF4B	0.0357833	-2.0987
211121_s_at	DOK1	0.0870071	1.55911
211123_at	SLC5A5	0.0329577	1.29296
211204_at	ME1	0.0556305	1.29595
211275_s_at	GYG1	0.0002095	-2.18285
211276_at	TCEAL2	0.0000003	-77.0008
211297_s_at	CDK7	0.0003317	-5.86387
211340_s_at	MCAM	0.0002688	3.01765
211341_at	POU4F1	0.060698	1.25297
211343_s_at	COL13A1	0.0001541	8.53815
211349_at	SLC15A1	0.0133693	1.17709
211382_s_at	TACC2	0.0239688	1.50689
211429_s_at	SERPINA1	0.0359702	1.75907
211458_s_at	GABARAPL1///	0.0000299	-2.64054
211470_s_at	SULT1C2	0.0000287	13.4161
211491_at	ADRA1A	0.0063194	1.46897
211494_s_at	SLC4A4	0.0000708	-8.22327
211509_s_at	RTN4	0.0227361	-2.05705
211538_s_at	HSPA2	0.0000506	-6.49867
211551_at	EGFR	0.0235403	1.1805
211558_s_at	DHPS	0.0836512	1.34717
211576_s_at	SLC19A1	0.0017175	2.33892
211596_s_at	LRIG1	0.0279563	2.83576
211600_at	PTPRO	0.0006889	-2.27554
211615_s_at	LRPPRC	0.0258571	-1.96209
211623_s_at	FBL	0.0031306	-2.15483
211651_s_at	LAMB1	0.0015361	-2.81807
211662_s_at	VDAC2	0.0262539	-2.58648
211673_s_at	MOCS1	0.0377614	1.43548
211675_s_at	MDFIC	0.0000003	-8.87337
211676_s_at	IFNGR1	0.001044	-3.04061
211685_s_at	NCALD	0.0109431	2.00652
211698_at	EID1	0.0000898	-2.77684
211701_s_at	TRO	0.0650513	1.62185
211712_s_at	ANXA9	0.0008999	-2.96687
211715_s_at	BDH1	0.0000569	1.90301

211755_s_at	ATP5F1	0.0056072	-3.12991
211773_s_at	ZKSCAN3	0.0040498	1.45307
211778_s_at	OVOL2	0.0000185	2.55878
211783_s_at	MTA1	0.0004447	2.29955
211815_s_at	GGA3	0.0519934	1.19384
211905_s_at	ITGB4	0.0603922	1.3896
211926_s_at	MYH9	0.0718922	1.37504
211932_at	HNRNPA3	0.0009547	-2.01834
211936_at	HSPA5	0.0429856	-2.52849
211937_at	EIF4B	0.0038602	-2.01574
211950_at	UBR4	0.0161397	1.40765
211952_at	IPO5	0.000059	-3.17692
211956_s_at	EIF1	0.0003274	-2.11471
211959_at	IGFBP5	0.0719837	1.83126
211962_s_at	ZFP36L1	0.0072836	-1.92035
211963_s_at	ARPC5	0.0000214	-6.74607
211964_at	COL4A2	0.0000405	4.95959
211967_at	TMEM123	0.0160638	-3.24341
211969_at	HSP90AA1	0.0088182	-2.42836
211979_at	GPR107	0.0731635	1.36296
211980_at	COL4A1	0.0015724	4.56867
211984_at	CALM1	0.0035503	-3.19555
211986_at	AHNAK	0.0000127	-3.64103
212001_at	SFRS14	0.000169	1.58775
212006_at	UBXN4	0.0121221	-2.08852
212013_at	PXDN	0.0012229	3.94912
212018_s_at	RSL1D1	0.0010058	-5.14869
212023_s_at	MKI67	0.0000027	3.27847
212031_at	RBM25	0.0002895	3.56129
212034_s_at	EXOC7	0.0722774	1.41485
212049_at	WIPF2	0.0029468	-2.11474
212052_s_at	TBC1D9B	0.0028623	2.21561
212063_at	CD44	0.0029003	-3.73998
212067_s_at	C1R	0.0003732	-3.76139
212070_at	GPR56	0.0000031	8.33086
212072_s_at	CSNK2A1	0.0598274	1.79001
212073_at	CSNK2A1///CS	0.011244	-3.10288
212077_at	CALD1	0.0001664	-2.37489
212093_s_at	MTUS1	0.0012396	-2.28133
212097_at	CAV1	0.0000008	-11.797

212104_s_at	RBM9	0.0044938	-2.12346
212108_at	FAF2	0.0091442	-2.25343
212111_at	STX12	0.0000287	-3.27244
212115_at	HN1L	0.0019476	1.67007
212116_at	TRIM27	0.030211	1.48707
212117_at	RHOQ	0.0002047	-2.51028
212123_at	TCTN3	0.002108	-1.97083
212126_at	CBX5	0.0045256	1.82452
212131_at	LSM14A	0.0002621	-3.21324
212139_at	GCN1L1	0.0042783	1.8286
212140_at	PDS5A	0.0027739	-1.70361
212150_at	EFR3A	0.0499247	-2.24578
212157_at	SDC2	0.000116	-3.20951
212160_at	XPOT	0.0089736	2.35952
212163_at	KIDINS220	0.0031531	-2.71201
212170_at	RBM12	0.0001607	-3.59819
212184_s_at	TAB2	0.0022745	-1.85692
212194_s_at	TM9SF4	0.0747155	1.41484
212196_at	IL6ST	0.0000031	-4.50721
212199_at	MRFAP1L1	0.0004624	-2.83519
212202_s_at	TMEM87A	0.0000434	-2.95492
212207_at	MED13L	0.0265016	-2.16484
212217_at	PREPL	0.0031681	-2.31409
212219_at	PSME4	0.0163827	1.68969
212224_at	ALDH1A1	0.0000857	-3.66334
212231_at	FBXO21	0.0264106	-1.98096
212232_at	FNBP4	0.0351766	-2.28817
212238_at	ASXL1	0.0074618	1.40221
212239_at	PIK3R1	0.0140158	-2.21985
212241_at	GCOM1///GRIN	0.0000778	-2.31442
212242_at	TUBA4A	0.0000128	2.65834
212245_at	MCFD2	0.0001022	-4.85804
212250_at	MTDH	0.026752	-2.15248
212254_s_at	DST	0.0000018	-4.84498
212256_at	GALNT10	0.0002529	-2.99138
212268_at	SERPINB1	0.0330111	-3.25886
212274_at	LPIN1	0.00026	-2.44553
212285_s_at	AGRN	0.0119849	1.39238
212287_at	SUZ12	0.0001123	-1.80176
212294_at	GNG12	0.0000035	-4.89138

212295_s_at	SLC7A1	0.0003743	3.32702
212299_at	NEK9	0.0000195	-2.52726
212301_at	RTF1	0.0002625	-2.45026
212308_at	CLASP2	0.0004281	-2.72796
212310_at	MIA3	0.0010753	-2.3476
212314_at	SEL1L3	0.0012522	-3.33552
212322_at	SGPL1	0.0000917	1.99259
212327_at	LIMCH1	0.0041686	-2.66851
212331_at	RBL2	0.0008061	-2.3731
212335_at	GNS	0.0042586	-2.15236
212341_at	YIPF6	0.0013428	-3.01439
212349_at	POFUT1	0.0134812	1.71939
212354_at	SULF1	0.0120279	-5.60684
212359_s_at	KIAA0913	0.057698	1.24217
212366_at	ZNF292	0.000465	-3.06094
212367_at	FEM1B	0.002013	-2.15799
212369_at	ZNF384	0.0011509	1.70944
212375_at	EP400	0.0037276	-2.26459
212380_at	FTSJD2	0.0018133	1.63714
212388_at	USP24	0.0013191	-2.85312
212397_at	RDX	0.0005254	-7.63316
212401_s_at	CDK11A	0.0019546	2.01892
212405_s_at	METTL13	0.000562	1.80684
212409_s_at	TOR1AIP1	0.0004472	-2.28643
212410_at	EFHA1	0.0010643	-2.32352
212414_s_at	GLYR1///SEPT6	0.0337423	1.60821
212418_at	ELF1	0.012319	-2.38995
212425_at	SCAMP1	0.0012528	-1.7348
212427_at	KIAA0368	0.0005755	-2.02672
212430_at	RBM38	0.0062762	1.62937
212438_at	SNRNP27	0.0002112	1.89123
212446_s_at	LASS6	0.006886	-1.93964
212447_at	KBTBD2	0.0090166	-1.88618
212451_at	SECISBP2L	0.0346896	-3.02535
212456_at	KIAA0664	0.0564706	1.74993
212458_at	SPRED2	0.0184837	-1.96269
212461_at	AZIN1	0.0398196	-2.43558
212467_at	DNAJC13	0.0209621	-1.93639
212468_at	SPAG9	0.0004731	-3.60722
212473_s_at	MICAL2	0.0011763	-2.94864

212481_s_at	TPM4	0.0491464	1.84488
212482_at	RMND5A	0.0005145	-1.82736
212487_at	GPATCH8	0.008723	-3.02621
212490_at	DNAJC8	0.0000003	-3.27641
212500_at	ADO	0.0000689	-5.28932
212507_at	TMEM131	0.012556	-1.88797
212510_at	GPD1L	0.0046947	-3.44332
212512_s_at	CARM1	0.0297784	1.60512
212513_s_at	USP33	0.0029079	-3.29634
212515_s_at	DDX3X	0.0022368	-1.75877
212522_at	PDE8A	0.0001022	-3.61658
212530_at	NEK7	0.0000002	-5.50767
212542_s_at	PHIP	0.0014731	-5.32307
212544_at	ZNHIT3	0.0001071	-4.66944
212553_at	RPRD2	0.0003176	2.08278
212554_at	CAP2	0.0025106	-2.06274
212556_at	SCRIB	0.0000103	5.68956
212558_at	SPRY1	0.0017461	-3.2703
212560_at	SORL1	0.0029721	4.55945
212571_at	CHD8	0.0607433	1.28046
212573_at	ENDOD1	0.0000375	-7.41346
212580_at	ERAP1	0.0009016	-1.97588
212585_at	OSBPL8	0.0124326	-1.98604
212586_at	CAST	0	-12.0408
212588_at	PTPRC	0.0058618	-5.28058
212589_at	RRAS2	0.0001952	-4.3705
212591_at	RBM34	0.0155378	-2.15889
212600_s_at	UQCRC2	0.0001322	-2.0264
212604_at	MRPS31	0.0011367	-2.38762
212606_at	WDFY3	0.0000201	-3.38956
212609_s_at	AKT3	0.0000392	-4.25856
212613_at	BTN3A2	0.0021957	-1.7241
212614_at	ARID5B	0.0002374	-5.7015
212622_at	TMEM41B	0.010331	-2.3757
212624_s_at	CHN1	0.0084564	-3.12582
212625_at	STX10	0.0855297	1.22566
212628_at	PKN2	0.0055333	-3.48131
212631_at	STX7	0.000545	-1.96937
212634_at	KIAA0776	0.0149643	-2.4699
212637_s_at	WWP1	0.0402134	-2.08778

212648_at	DHX29	0.0000157	-3.22334
212650_at	EHBP1	0.0377094	-2.45141
212655_at	ZCCHC14	0.0003498	-3.9492
212658_at	LHFPL2	0.0003429	-3.40687
212665_at	TIPARP	0.0150542	-2.42662
212666_at	SMURF1	0.005492	1.22679
212667_at	SPARC	0.0154857	-2.6445
212672_at	ATM	0.0001208	-4.76829
212681_at	EPB41L3	0.0029683	-1.9386
212686_at	PPM1H	0.0115494	1.48828
212690_at	DDHD2	0.0000291	-3.58169
212698_s_at	Sep 10, 2013	0.0005785	-3.42337
212716_s_at	EIF3K	0.0002408	-3.0107
212727_at	DLG3	0.0031068	1.67085
212730_at	SYNM	0.0011459	2.01612
212731_at	ANKRD46	0.0078363	-3.23699
212738_at	ARHGAP19	0.0000159	-2.36486
212739_s_at	NME4	0.0483208	1.43266
212740_at	PIK3R4	0.0069974	-2.15606
212744_at	BBS4	0.0069414	-2.14544
212747_at	ANKS1A	0.0020652	1.42529
212751_at	UBE2N	0.0334914	-2.17473
212754_s_at	MON2	0.0002545	-2.59005
212757_s_at	CAMK2G	0.0088305	1.33439
212758_s_at	ZEB1	0.0189689	-2.22812
212760_at	UBR2	0.0000314	-2.08858
212769_at	TLE3	0.0017759	2.32764
212771_at	FAM171A1	0.0000031	7.86246
212772_s_at	ABCA2	0.0037219	1.37256
212774_at	ZNF238	0.0089111	-2.29967
212779_at	KIAA1109	0.0017866	-3.66439
212785_s_at	LARP7	0.0046973	-2.05971
212789_at	NCAPD3	0.0002453	2.11338
212792_at	DPY19L1	0.0000036	-5.76789
212794_s_at	KIAA1033	0.0123784	-2.67346
212798_s_at	ANKMY2	0.0000002	-6.15093
212800_at	STX6	0.0007224	1.91502
212802_s_at	GAPVD1	0.0028902	-2.75555
212805_at	PRUNE2	0.0115215	-2.90907
212807_s_at	SORT1	0.0000005	4.80691

212809_at	NFATC2IP	0.0000016	3.09354
212813_at	JAM3	0.0117967	-2.52297
212820_at	DMXL2	0.0049561	-2.33974
212827_at	IGHM	0.0487328	1.52925
212829_at	PIP4K2A	0.0008317	-1.80477
212830_at	MEGF9	0.0003792	-2.55031
212832_s_at	CKAP5	0.0327469	1.43351
212833_at	SLC25A46	0.0320026	-2.21674
212837_at	FAM175B	0.0000946	-2.89461
212851_at	DCUN1D4	0.0023015	-2.3876
212852_s_at	TROVE2	0.0113135	-2.36643
212864_at	CDS2	0.0026481	-2.09338
212865_s_at	COL14A1	0.0269746	-2.9024
212870_at	SOS2	0.0000032	-3.53757
212877_at	KLC1	0.0328142	1.55355
212880_at	WDR7	0.0006145	-2.5635
212886_at	CCDC69	0.001201	-1.6532
212887_at	SEC23A	0.0113491	-2.94435
212898_at	KIAA0406	0.0030103	1.71022
212905_at	CSTF2T	0.0004851	-3.16607
212907_at	SLC30A1	0.0018956	-5.36404
212908_at	DNAJC16	0.0050042	-3.40561
212909_at	LYPD1	0.0000843	4.52004
212913_at	C6orf26	0.0000076	4.05584
212914_at	CBX7	0.0002896	-2.58127
212923_s_at	C6orf145	0.0953156	1.55822
212930_at	ATP2B1	0.0027286	-2.11926
212936_at	FAM172A	0.0020541	-1.84846
212942_s_at	KIAA1199	0.0007882	2.74249
212945_s_at	MGA	0.0011953	1.38467
212946_at	KIAA0564	0.0010035	-3.18958
212949_at	NCAPH	0.0002195	1.76259
212950_at	GPR116	0.0159816	1.88499
212955_s_at	POLR2I	0.0414252	1.64123
212956_at	TBC1D9	0.0004321	-6.88865
212973_at	RPIA	0.0230789	1.6846
212975_at	DENND3	0.0355218	1.54405
212976_at	LRRC8B	0.0294889	1.42142
212982_at	ZDHHC17	0.0019639	-2.58925
212984_at	ATF2	0.0071592	-1.91169

212989_at	SGMS1	0.0129484	-3.68114
212990_at	SYNJ1	0.0002162	-2.83727
212992_at	AHNAK2	0.0205225	3.18561
212993_at	NACC2	0.000546	-4.14625
213000_at	MORC3	0.001058	-2.65537
213001_at	ANGPTL2	0.000027	-2.62725
213007_at	FANCI	0.0001799	2.35423
213009_s_at	TRIM37	0.0096568	-2.12935
213011_s_at	TPI1	0.0021457	2.68961
213012_at	NEDD4	0.0000802	-1.73029
213017_at	ABHD3	0.0363948	-2.07966
213018_at	GATAD1	0.0009644	-1.67552
213019_at	RANBP6	0.0022035	-2.27012
213021_at	GOSR1	0.0008276	-1.91727
213025_at	THUMPD1	0.0041204	-2.38395
213026_at	ATG12	0.0028711	-3.67644
213033_s_at	NFIB	0.0003035	-2.13641
213034_at	SIK3	0.0000095	-3.74271
213044_at	ROCK1	0.0000234	-4.13773
213049_at	RALGAPA1	0.0022389	-2.46884
213050_at	COBL	0.0000061	-4.66447
213056_at	FRMD4B	0.0000555	-8.10461
213061_s_at	NTAN1	0.0005222	-3.48713
213064_at	ZC3H14	0.0036261	-2.33903
213069_at	HEG1	0.0000054	-5.61358
213075_at	OLFML2A	0.0002428	3.04557
213079_at	TSR2	0.0100057	1.3333
213093_at	PRKCA	0.0000008	-8.4217
213094_at	GPR126	0.0012865	-2.72385
213101_s_at	ACTR3	0.0861493	1.41415
213105_s_at	C16orf42	0.0034295	2.33364
213106_at	ATP8A1	0.0001023	-3.90075
213110_s_at	COL4A5	0.0015476	-3.17047
213111_at	PIKFYVE	0.0052491	-2.84404
213117_at	KLHL9	0.007359	-2.00663
213118_at	UHRF1BP1L	0.0036003	-3.75071
213123_at	MFAP3	0.002949	-2.15277
213125_at	OLFML2B	0.0156068	1.44677
213130_at	ZNF473	0.0161657	1.23157
213132_s_at	MCAT	0.0437258	1.25789

213135_at	TIAM1	0	-16.2959
213139_at	SNAI2	0.0001031	-4.91292
213145_at	FBXL14	0.053625	1.33531
213151_s_at	Sep 7, 2013	0.0004503	-2.36287
213153_at	SETD1B	0.0105844	1.47212
213161_at	TMOD1///TSTD	0.0020279	-2.37505
213183_s_at	CDKN1C	0.0819069	1.62591
213186_at	DZIP3	0.0018239	-1.84436
213191_at	TICAM1	0.0070052	1.59312
213194_at	ROBO1	0.0000023	-6.49838
213197_at	ASTN1	0.0067307	-1.94518
213198_at	ACVR1B	0.0176948	1.40472
213204_at	CUL9	0.0517747	1.93617
213208_at	KIAA0240	0.0016188	-1.77821
213222_at	PLCB1	0.0001523	-4.49606
213224_s_at	NCRNA00081	0.0000058	-2.7568
213226_at	CCNA2	0.0022775	2.86396
213230_at	CDR2L	0.0699589	1.41716
213237_at	C16orf88	0.0003464	1.49608
213238_at	ATP10D	0.0000002	-6.11833
213239_at	PIBF1	0.004869	-1.95417
213241_at	PLXNC1	0.0121878	-3.30454
213246_at	C14orf109	0.0135492	-2.55737
213247_at	SVEP1	0	-7.11218
213249_at	FBXL7	0.0000039	-3.86794
213251_at	SMARCA5	0.0010367	-2.00745
213270_at	MPP2	0.0818353	1.28921
213272_s_at	TMEM159	0.0020584	-2.69953
213278_at	MTMR9	0.0010906	-2.27024
213280_at	RAP1GAP2	0.0018962	2.9928
213283_s_at	SALL2	0.0094546	-1.97868
213287_s_at	KRT10	0.0025692	-2.1454
213293_s_at	TRIM22	0.0010417	-4.34822
213302_at	PFAS	0.0240988	2.03801
213304_at	FAM179B	0.000881	-3.57071
213309_at	PLCL2	0	-13.508
213310_at	EIF2C2	0.0068882	2.4139
213317_at	CLIC5	0.0000028	-3.42142
213318_s_at	BAT3	0.0357171	1.66661
213322_at	C6orf130	0.013931	-1.8864

213326_at	VAMP1	0.0129199	1.9462
213327_s_at	USP12	0.0000004	-6.2
213331_s_at	NEK1	0.0001703	-2.06846
213344_s_at	H2AFX	0.0008503	2.35524
213349_at	TMCC1	0.0171849	1.39637
213353_at	ABCA5	0.0016063	-3.23702
213355_at	ST3GAL6	0.0862624	1.86409
213357_at	GTF2H5	0.0034862	-2.4551
213364_s_at	SNX1	0.0000011	-4.01749
213370_s_at	SFMBT1	0.0000428	2.0053
213372_at	PAQR3	0.0005313	-2.82788
213376_at	ZBTB1	0.0019039	-2.53371
213385_at	CHN2	0.0000598	-6.68685
213386_at	C9orf125	0.0040585	-1.73584
213389_at	ZNF592	0.0002344	1.73595
213405_at	RAB22A	0.0002233	-2.75038
213407_at	PHLPP2	0	-11.1414
213422_s_at	MXRA8	0.0310533	1.74483
213424_at	KIAA0895	0.0000013	-3.42353
213427_at	RPP40	0.0692819	1.86268
213434_at	STX2	0.0010733	-2.52349
213438_at	NFASC	0.0018034	-2.27246
213440_at	RAB1A	0.0123173	-1.91997
213446_s_at	IQGAP1	0.0378892	-2.70069
213455_at	FAM114A1	0.0000745	-4.09911
213456_at	SOSTDC1	0.0000236	-7.64646
213457_at	MFHAS1	0.0303422	2.09701
213463_s_at	FAM149B1	0.0026881	-2.61866
213473_at	BRAP	0.0031063	-2.11655
213478_at	KAZ	0.0297087	1.7053
213479_at	NPTX2	0.0023777	2.48956
213483_at	PPWD1	0.0001142	-2.96931
213485_s_at	ABCC10	0.0426654	2.14293
213499_at	CLCN2	0.0672445	1.42031
213501_at	ACOX1	0.0121377	-2.08523
213507_s_at	KPNB1	0.0035631	-1.70006
213508_at	C14orf147	0.0000594	-3.2493
213511_s_at	MTMR1	0.0603067	1.33738
213518_at	PRKCI	0.013525	1.80742
213519_s_at	LAMA2	0.0044692	-2.03845

213520_at	RECQL4	0.0000798	2.10576
213523_at	CCNE1	0.0000159	10.6148
213524_s_at	G0S2	0.0000454	-5.06051
213528_at	C1orf156	0.0004729	-2.94574
213531_s_at	RAB3GAP1	0.0018528	-1.72045
213546_at	DKFZP586I142(	0.0000004	-8.48391
213547_at	CAND2	0.0086699	-2.25323
213548_s_at	CDV3	0.0041379	-1.80262
213549_at	PDZD8	0.0003351	-2.37785
213550_s_at	TMCO6	0.0598825	1.52728
213555_at	RWDD2A	0.0031196	-2.25446
213557_at	CDK12	0.0165735	2.47523
213558_at	PCLO	0.0000144	-4.03783
213578_at	BMPR1A	0.0003624	-1.93028
213582_at	ATP11A	0.0023648	-1.70351
213587_s_at	ATP6V0E2	0.0029062	1.81551
213595_s_at	CDC42BPA	0.013465	1.39936
213599_at	OIP5	0.0003282	2.3629
213603_s_at	RAC2	0.0001083	-3.27614
213604_at	TCEB3	0.0009243	-1.96304
213622_at	COL9A2	0.0007223	2.09135
213624_at	SMPDL3A	0.000132	-2.43043
213627_at	MAGED2	0.0611424	1.29403
213628_at	CLCC1	0.0056277	-1.76631
213644_at	CCDC46	0.0003516	-3.20749
213647_at	DNA2	0.0214588	1.67156
213654_at	TAF5L	0.0970158	1.24029
213655_at	YWHAE	0.0051149	-4.0689
213664_at	SLC1A1	0.00025	-2.16326
213666_at	Sep 6, 2013	0.0018453	1.5663
213671_s_at	MARS	0.0020032	1.82649
213679_at	TTC30A	0.0282872	1.77782
213682_at	NUP50	0.0038531	-2.94475
213693_s_at	MUC1	0	29.3727
213694_at	RSBN1	0.0004711	-2.52363
213701_at	C12orf29	0.0000029	-6.12146
213720_s_at	SMARCA4	0.0029081	2.33512
213728_at	LAMP1	0.0764954	1.42991
213736_at	COX5B	0.0003029	2.59271
213738_s_at	ATP5A1	0.0000004	-4.07136

213745_at	ATRNL1	0.0289279	-2.12231
213757_at	EIF5A	0.0512043	1.60571
213764_s_at	MFAP5	0.0075738	4.57429
213786_at	TAX1BP1	0.0000224	-2.90859
213798_s_at	CAP1	0.0061818	-1.8405
213800_at	CFH	0.0000003	-12.3037
213816_s_at	MET	0.005007	-2.24489
213817_at	IRAK3	0.0000404	-3.01911
213832_at	KCND3	0.0052063	-1.86784
213838_at	NOL7	0.0011289	-3.72122
213846_at	COX7C	0.0205117	-2.34685
213849_s_at	PPP2R2B	0.0032071	-3.04053
213851_at	TMEM110	0.0106839	1.56392
213857_s_at	CD47	0.0028038	1.99779
213872_at	C6orf62	0.0007595	-5.041
213882_at	TM2D1	0.0347931	-2.07535
213891_s_at	TCF4	0.0019704	-2.07353
213900_at	FAM189A2	0.0544148	1.33114
213902_at	ASAH1	0.0237944	-1.96233
213907_at	EEF1E1	0.0627108	1.13572
213908_at	WHAMML1///W	0.0024364	-2.28976
213909_at	LRRC15	0.0014329	2.70986
213927_at	MAP3K9	0.0001983	1.86351
213933_at	PTGER3	0	-6.7459
213934_s_at	ZNF23	0.0063805	-1.89986
213938_at	ERC2	0.0000225	-2.89413
213960_at	NTRK3	0.0170309	1.50891
213970_at	RABL3	0.0069219	-2.08857
213975_s_at	LYZ	0.0222122	-3.57427
213976_at	CIZ1	0.0206046	1.29068
213982_s_at	RABGAP1L	0.0003378	-3.57149
213988_s_at	SAT1	0.0640051	-2.35633
213992_at	COL4A6	0.0000005	-6.72648
213993_at	SPON1	0	23.519
213998_s_at	DDX17	0.001086	-9.70375
214005_at	GGCX	0.0002204	3.57829
214009_at	MSL3	0.0357067	1.18516
214011_s_at	NOP16	0.0654193	1.59554
214030_at	CRYBG3	0.000033	-4.33273
214036_at	EFNA5	0.000144	-4.45091

214037_s_at	CCDC22	0.0132381	1.6298
214042_s_at	RPL22	0.0027512	-1.76483
214044_at	RYR2	0.0000001	-20.1022
214046_at	FUT9	0.0178339	-2.24909
214058_at	MYCL1	0.0051247	2.00313
214073_at	CTTN	0.0572051	2.40369
214091_s_at	GPX3	0.0023961	1.4973
214096_s_at	SHMT2	0.0000068	3.3137
214102_at	ARAP2	0.0024474	-2.74564
214120_at	RFPL1S	0.000806	-2.45334
214129_at	PDE4DIP	0.0788654	1.49709
214149_s_at	ATP6V0E1	0.0474112	1.66409
214152_at	CCPG1	0.008626	-1.90415
214155_s_at	LARP4	0.0211171	2.02297
214157_at	GNAS	0.0012883	4.29619
214158_s_at	PRDM10	0.0621307	1.3396
214167_s_at	RPLP0///RPLP(	0.0057491	-3.53049
214168_s_at	TJP1	0.0563295	1.3997
214185_at	KHDRBS1	0.0438132	1.18929
214194_at	DIS3	0.0000602	-2.04569
214199_at	SFTPD	0.0008956	-2.29554
214200_s_at	COL6A1	0.0606875	1.6998
214204_at	PACRG	0.0009091	-3.13167
214210_at	SLC25A17	0.0004393	-2.2501
214211_at	FTH1	0.0165838	-1.95783
214234_s_at	CYP3A5	0.000002	-2.96911
214241_at	NDUFB8	0.0043366	1.59938
214254_at	MAGEA4	0.008402	3.19748
214255_at	ATP10A	0.0104788	2.26166
214264_s_at	C14orf143	0.0233033	1.8046
214276_at	KLF12	0.0162518	1.37539
214281_s_at	RCHY1	0.0003346	-3.50874
214285_at	FABP3	0.0400484	-2.09973
214290_s_at	HIST2H2AA3///	0.0162229	1.57932
214297_at	CSPG4	0.0004464	1.68542
214319_at	FRY	0.0000101	-4.89693
214338_at	DNAJB12	0.0363488	1.5688
214339_s_at	MAP4K1	0.0006859	1.92835
214359_s_at	HSP90AB1	0.0155539	-3.76394
214369_s_at	RASGRP2	0.0177714	1.44015

214370_at	S100A8	0.0162406	-2.2924
214417_s_at	FETUB	0.0534576	1.44366
214427_at	NOP2	0.0000186	1.86905
214429_at	MTMR6	0.000035	-2.02319
214430_at	GLA	0.0000008	-3.18127
214431_at	GMPS	0.0018154	1.948
214433_s_at	SELENBP1	0.0052117	-2.15489
214434_at	HSPA12A	0.0000001	-6.08452
214440_at	NAT1	0.0300027	-2.53714
214452_at	BCAT1	0.0192774	1.77528
214455_at	HIST1H2BC	0.048745	1.46402
214551_s_at	CD7	0.0451808	2.02569
214582_at	PDE3B	0.00067	-1.78375
214585_s_at	VPS52	0.0190168	1.51631
214586_at	GPR37	0.0000158	-3.41802
214591_at	KLHL4	0.0036289	-1.82292
214638_s_at	CCNT2	0.0496638	1.19438
214657_s_at	NEAT1	0.0004905	8.16344
214658_at	TMED7	0.0043737	-3.54981
214662_at	WDR43	0.0001083	2.95808
214663_at	DSTYK	0.0037224	-1.84844
214674_at	USP19	0.0010032	1.45405
214683_s_at	CLK1	0.0000238	-11.8082
214696_at	C17orf91	0.0033619	-2.72099
214705_at	INADL	0.0216953	-2.35012
214708_at	SNTB1	0.0012149	2.28944
214709_s_at	KTN1	0.0000037	-3.61294
214714_at	ZNF394	0.0002361	-2.71158
214719_at	SLC46A3	0.0000003	-4.06484
214722_at	NOTCH2NL	0.0088281	-2.46797
214724_at	DIXDC1	0.0000013	-6.36678
214725_at	C8orf84	0.0000005	-5.12085
214736_s_at	ADD1	0.005563	-1.76871
214741_at	ZNF131	0.0018188	-2.26348
214743_at	CUX1	0.0126219	-1.85284
214746_s_at	ZNF467	0.0101043	1.33716
214760_at	ZNF337	0.0733074	1.21024
214761_at	ZNF423	0.0015775	4.43776
214764_at	RRP15	0.0009329	-2.75471
214779_s_at	SGSM3	0.0620945	1.49071

214785_at	VPS13A	0.0065437	-1.85749
214804_at	CENPI	0.0000374	2.65767
214823_at	ZNF204P	0.0010054	-2.70595
214829_at	AASS	0.0090601	-2.42316
214835_s_at	SUCLG2	0.0131848	-3.0318
214838_at	SFT2D2	0.0206204	1.85886
214841_at	CNIH3	0.0000284	-2.91145
214844_s_at	DOK5	0.0126808	-3.20735
214853_s_at	SHC1	0.0003645	2.39948
214861_at	KDM4C	0.001546	-2.37944
214909_s_at	DDAH2	0.0441387	1.39699
214913_at	ADAMTS3	0.0000043	-6.18786
214914_at	FAM13C	0.0345828	1.30264
214920_at	THSD7A	0.0878586	1.41414
214924_s_at	TRAK1	0.0014326	1.92937
214930_at	SLITRK5	0.0155265	-2.94893
214933_at	CACNA1A	0.0002557	5.38966
214945_at	FAM153A///FAM	0	-54.7874
214965_at	SPATA2L	0.0308301	1.58038
214978_s_at	PPFIA4	0.0065596	1.76861
214987_at	GAB1	0.0000027	-2.3749
214988_s_at	SON	0.0037675	-1.97568
214993_at	ASPHD1	0.0000593	-2.1246
214994_at	APOBEC3F	0.09686	1.51721
215000_s_at	FEZ2	0	-7.97134
215001_s_at	GLUL	0.0000882	-1.86838
215009_s_at	SEC31A	0.0329661	-2.11427
215012_at	ZNF451	0.0066824	-2.70189
215039_at	HS2ST1///LOC3	0.0012597	-2.23823
215054_at	EPOR	0.0104038	2.52358
215082_at	ELOVL5	0.011721	1.32813
215088_s_at	SDHC	0.0201623	1.60369
215096_s_at	ESD	0.0000003	-2.99817
215131_at	IQCK	0.004196	1.41423
215133_s_at	FAM153A///FAM	0.0000302	-3.9745
215136_s_at	EXOSC8	0.0009965	-2.27136
215139_at	ARHGEF10	0.0040072	-1.78709
215143_at	DPY19L2P2	0.0000031	-8.64523
215156_at	WDR61	0.000284	-1.88649
215220_s_at	TPR	0.0273159	-2.07058

215243_s_at	GJB3	0.0430144	1.2552
215269_at	TRAPPC10	0.0038508	-1.84488
215271_at	TNN	0.091389	1.35137
215276_at	WFDC8	0.03809	1.45176
215285_s_at	PHTF1	0.0939594	1.18314
215321_at	RUNDC3B	0.0000331	-2.51804
215354_s_at	PELP1	0.0144756	1.86781
215388_s_at	CFH///CFHR1	0.000001	-10.4566
215411_s_at	TRAF3IP2	0.0000981	-1.90449
215440_s_at	BEX4	0.0043259	-3.54986
215478_at	RIMS2	0.0006598	1.41126
215501_s_at	DUSP10	0.0194479	1.35796
215506_s_at	DIRAS3	0.0003216	-4.17714
215513_at	HYMAI	0.0036158	-2.63813
215544_s_at	UBOX5	0.0179408	1.26491
215548_s_at	SCFD1	0.0002216	-4.7434
215567_at	FCF1	0.0030436	-3.05391
215591_at	SATB2	0.0157791	1.56433
215640_at	TBC1D2B	0.0002445	-1.65707
215683_at	C18orf22	0.0054372	1.80194
215704_at	FLG	0.0467867	2.38075
215707_s_at	PRNP	0.0260115	1.19898
215726_s_at	CYB5A	0.0167119	-3.38497
215760_s_at	SBNO2	0.001633	1.93622
215765_at	LRRC41	0.0013114	1.66704
215884_s_at	UBQLN2	0.0009859	-2.17571
215888_at	PDS5B	0.0233948	-2.321
215903_s_at	MAST2	0.0000448	2.789
215917_at	RAD21L1	0.0691562	1.28284
215947_s_at	FAM136A	0.0000168	2.17841
215972_at	PART1	0.0056319	2.22347
215983_s_at	UBXN8	0.0031936	-2.04642
216026_s_at	POLE	0.0185577	1.38101
216085_at	DKFZP434C153	0.0597221	1.26528
216115_at	NF1	0.0022088	-1.7019
216194_s_at	TBCB	0.0728388	1.75326
216199_s_at	MAP3K4	0.0008329	-2.06306
216221_s_at	PUM2	0.0001205	-3.49987
216231_s_at	B2M	0.0000034	-12.0098
216241_s_at	TCEA1	0.0044846	-4.10194

216250_s_at	LPXN	0.008533	-2.43331
216257_at	SERPINB13	0.0101445	1.22807
216268_s_at	JAG1	0.0047276	2.82286
216269_s_at	ELN	0.0717876	1.47695
216274_s_at	SEC11A	0.0027977	-2.28609
216296_at	CLTA	0.0244391	1.24154
216321_s_at	NR3C1	0.0002911	-5.37634
216331_at	ITGA7	0.0000674	3.97231
216341_s_at	GNRHR	0.0065098	1.18462
216347_s_at	PPP1R13B	0.0067426	1.92704
216383_at	RPL18A///RPL1	0.0147908	1.54168
216397_s_at	BOP1///LOC72	0.0058761	1.61573
216452_at	TRPM3	0.0000028	-2.52358
216497_at	HNRNPA1///HN	0.0039918	-2.48081
216520_s_at	TPT1	0.0000009	-3.47157
216540_at	TRD@	0.0061941	1.35671
216563_at	ANKRD12	0.0287069	-2.22936
216678_at	IFT122	0.0319402	1.30823
216804_s_at	PDLIM5	0.0017976	-1.98694
216823_at	RPS3A	0.0002795	-1.63381
216834_at	RGS1	0.0019925	8.74697
216873_s_at	ATP8B2	0.0362326	1.60361
216913_s_at	RRP12	0.0271725	1.27411
216993_s_at	COL11A2	0.0251092	1.47656
217028_at	CXCR4	0.0011472	14.1594
217047_s_at	FAM13A	0.0006376	-2.35359
217097_s_at	PHTF2	0.0605446	1.15856
217118_s_at	C22orf9	0.0515998	1.49724
217122_s_at	SLC35E2	0.0064172	-1.97951
217168_s_at	HERPUD1	0.0000524	-3.38508
217182_at	MUC5AC	0.0529137	2.78771
217196_s_at	CAMSAP1L1	0.0132341	-1.97383
217204_at	MTRF1L	0.0366949	1.15689
217226_s_at	SFXN3	0.0934915	1.3339
217230_at	EZR	0.0002921	-2.48908
217369_at	IGHG1///LOC1(	0.009657	1.26842
217427_s_at	HIRA	0.0725494	1.66724
217428_s_at	COL10A1	0.0961711	2.04924
217484_at	CR1	0.0484108	1.30816
217492_s_at	PTEN///PTENP	0.0026483	-3.01682

217497_at	TYMP	0.0590811	1.35886
217525_at	OLFML1	0	-16.3135
217538_at	SGSM2	0.0615945	1.26477
217550_at	ATF6	0.0098557	-2.15887
217590_s_at	TRPA1	0.0000001	-9.38419
217627_at	ZNF573	0.0000324	-5.24458
217645_at	COX16	0.0020793	-4.13237
217717_s_at	YWHAB	0.0172058	-2.44302
217719_at	EIF3L	0.0000001	-2.41594
217722_s_at	NGRN	0.0102638	-1.88949
217726_at	COPZ1	0.0021053	-2.13531
217731_s_at	ITM2B	0.001214	-1.93978
217733_s_at	TMSB10	0.0005811	3.01792
217738_at	NAMPT	0.0000735	-6.71947
217746_s_at	PDCD6IP	0.0219282	-2.15292
217747_s_at	RPS9	0.0200182	-2.63057
217749_at	COPG	0.0081356	1.41556
217751_at	GSTK1	0.0115743	1.71169
217759_at	TRIM44	0.0026924	-2.20971
217762_s_at	RAB31	0.000037	-6.75116
217767_at	C3	0.0003241	-12.0441
217768_at	C14orf166	0.0000193	-4.61735
217771_at	GOLM1	0.0000001	-16.9044
217773_s_at	NDUFA4	0.0006844	-2.09496
217778_at	SLC39A1	0.0028034	1.60729
217781_s_at	ZFP106	0.0000786	-4.14316
217783_s_at	YPEL5	0.0003959	-1.90434
217788_s_at	GALNT2	0.0101647	1.90007
217790_s_at	SSR3	0.0021875	-1.96532
217795_s_at	TMEM43	0.0000594	-2.63525
217796_s_at	NPLOC4	0.0678603	1.22932
217797_at	UFC1	0.0002619	-1.81893
217800_s_at	NDFIP1	0.0076923	-1.87053
217801_at	ATP5E	0.0318229	1.52607
217804_s_at	ILF3	0.0015052	1.73492
217807_s_at	GLTSCR2	0.0000991	-2.01544
217809_at	BZW2	0.0001031	3.41026
217811_at	SELT	0.0160807	-1.9464
217814_at	CCDC47	0.0353404	-2.1596
217823_s_at	UBE2J1	0.0001102	-3.69102

217834_s_at	SYNCRIP	0.0720323	2.00431
217837_s_at	VPS24	0.0000142	-2.68436
217838_s_at	EVL	0.004891	1.84867
217848_s_at	PPA1	0.0052406	-4.08948
217852_s_at	ARL8B	0.0010709	-2.88414
217863_at	PIAS1	0.0005197	-2.11201
217865_at	RNF130	0.0005427	-4.65191
217869_at	HSD17B12	0.0011973	-2.69332
217873_at	CAB39	0.0000283	-4.53975
217874_at	SUCLG1	0.0013388	-1.69065
217877_s_at	GPBP1L1	0.0009298	1.40722
217883_at	MMADHC	0.0010696	-2.64301
217886_at	EPS15	0.0163445	-2.07369
217888_s_at	ARFGAP1	0.0098076	1.83469
217889_s_at	CYBRD1	0.0000928	-2.30833
217890_s_at	PARVA	0.0003771	-3.60233
217894_at	KCTD3	0.0038332	-3.14819
217898_at	C15orf24	0.000601	-4.34338
217899_at	TMEM214	0.0324113	1.31407
217900_at	IARS2	0.0142025	-2.42222
217901_at	DSG2	0.0118925	-2.62736
217906_at	KLHDC2	0.0001869	-3.49473
217907_at	MRPL18	0.0014263	-3.17414
217908_s_at	DCAF6	0.0024148	-1.779
217911_s_at	BAG3	0.0004696	-2.67533
217915_s_at	RSL24D1	0.0027123	-2.90785
217925_s_at	C6orf106	0.0003194	2.24504
217926_at	C19orf53	0.0865495	1.48738
217927_at	SPCS1	0.0158531	-1.97883
217928_s_at	SAPS3	0.0889566	1.16818
217933_s_at	LAP3	0.0218298	-2.80688
217935_s_at	UQCC	0.0639157	1.38691
217939_s_at	AFTPH	0.0000441	-2.12867
217941_s_at	ERBB2IP	0.0086685	-1.95953
217944_at	POMGNT1	0.0004227	1.67205
217945_at	BTBD1	0.0000504	-2.26376
217950_at	NOSIP	0.0347692	2.03254
217953_at	PHF3	0.0002991	-1.98768
217955_at	BCL2L13	0.0004766	-4.12291
217956_s_at	ENOPH1	0.0041184	-4.23522

217957_at	C16orf80	0.0091974	-1.94793
217959_s_at	TRAPPC4	0.0067298	-3.1524
217964_at	TTC19	0.000006	-4.08772
217966_s_at	FAM129A	0.0159289	-2.61809
217971_at	MAPKSP1	0.0004987	-4.7527
217974_at	TM7SF3	0.0106088	-2.39511
217978_s_at	UBE2Q1	0.0001864	-1.92503
217979_at	TSPAN13	0.0000019	-4.97948
217980_s_at	MRPL16	0.0012655	-2.73217
217982_s_at	MORF4L1	0.0000219	-2.56834
217987_at	ASNSD1	0.0004168	-5.74677
217989_at	HSD17B11	0.0000004	-6.00333
217990_at	GMPR2	0.0207696	-2.58221
217993_s_at	MAT2B	0.0001675	-4.11071
217995_at	SQRDL	0.0000244	-10.94
218009_s_at	PRC1	0.0010945	3.66203
218023_s_at	FAM53C	0.0291567	1.27613
218024_at	BRP44L	0.0054683	-2.01485
218025_s_at	PECI	0.0000084	-6.79275
218031_s_at	FOXN3	0.003833	-2.27975
218035_s_at	RBM47	0.0082476	-2.98613
218039_at	NUSAP1	0.0000003	19.5353
218042_at	COPS4	0.0000091	-5.61143
218048_at	COMMD3	0.0009266	-2.01078
218050_at	UFM1	0.0031196	-2.37593
218061_at	MEA1	0.0213859	1.61922
218065_s_at	TMEM9B	0.0001959	-2.80982
218066_at	SLC12A7	0.0000041	2.38897
218068_s_at	ZNF672	0.0008569	1.63938
218069_at	DCTPP1	0.0111264	1.29758
218073_s_at	TMEM48	0.0299038	2.05519
218079_s_at	GGNBP2	0.0040794	-2.22318
218098_at	ARFGEF2	0.0371251	-2.52051
218104_at	TEX10	0.0070345	-2.07396
218106_s_at	MRPS10	0.017083	-1.86895
218108_at	UBR7	0.0009746	-3.2961
218109_s_at	MFSD1	0.0011917	-3.42949
218111_s_at	CMAS	0.0016888	-3.02932
218113_at	TMEM2	0.0001547	-1.58573
218123_at	C21orf59	0.0097522	-2.10876

218131_s_at	GATAD2A	0.0051174	1.97337
218138_at	MKKS	0.0004551	-2.06257
218142_s_at	CRBN	0.0001585	-2.71442
218146_at	GLT8D1	0.000349	-2.11084
218149_s_at	ZNF395	0.0000981	-4.71339
218150_at	ARL5A	0.0004652	-2.45338
218152_at	HMG20A	0.0008087	-3.49878
218154_at	GSDMD	0.001142	1.75979
218159_at	DDRGK1	0.0313528	1.51824
218163_at	MCTS1	0.0139538	-2.62603
218167_at	AMZ2	0.001918	-6.34773
218170_at	ISOC1	0.0059083	-2.85324
218173_s_at	WHSC1L1	0.0079271	-1.91725
218179_s_at	C4orf41	0.0001794	-3.05381
218180_s_at	EPS8L2	0.0213285	2.13564
218182_s_at	CLDN1	0.0164225	-2.0354
218191_s_at	LMBRD1	0.0000045	-8.57617
218195_at	C6orf211	0.0349113	-3.4033
218198_at	DHX32	0.001848	-1.90364
218203_at	ALG5	0.0001723	-5.19083
218204_s_at	FYCO1	0.000013	-6.97242
218217_at	SCPEP1	0.0186501	-2.05875
218220_at	C12orf10	0.0596601	1.37532
218221_at	ARNT	0.0007033	-1.84179
218224_at	PNMA1	0.0013285	-2.97543
218230_at	ARFIP1	0.010634	-2.13329
218236_s_at	PRKD3	0.0002986	-3.71121
218243_at	RUFY1	0.0001324	-1.85771
218244_at	NOL8	0.0157947	-2.19095
218247_s_at	MEX3C	0.011536	-2.55368
218253_s_at	LGTN	0.0065534	-2.10806
218256_s_at	NUP54	0.0038025	-3.23372
218257_s_at	UGGT1	0.0000822	1.53206
218263_s_at	ZBED5	0.0003518	-4.22811
218265_at	SECISBP2	0.0001092	-2.20858
218268_at	TBC1D15	0.0164697	-2.52248
218276_s_at	SAV1	0.0081583	-2.12836
218285_s_at	BDH2	0.0000008	-5.10662
218290_at	PLEKHJ1	0.0817285	1.42694
218292_s_at	PRKAG2	0.0418704	1.19555

218301_at	RNPEPL1	0.0011889	1.68516
218302_at	PSENEN	0.0868232	1.35509
218305_at	IPO4	0.0000053	2.81339
218306_s_at	HERC1	0.0003458	-2.03201
218309_at	CAMK2N1	0.0027688	-2.79557
218310_at	RABGEF1	0.0001267	-2.10537
218311_at	MAP4K3	0.0052478	-3.87092
218316_at	TIMM9	0.0026637	-2.29419
218319_at	PELI1	0.0528737	1.99969
218320_s_at	NDUFB11	0.0004797	2.29736
218323_at	RHOT1	0.0008856	-2.05652
218329_at	PRDM4	0.0063175	-1.77254
218330_s_at	NAV2	0.0001345	3.99736
218332_at	BEX1	0.0063002	-6.72965
218333_at	DERL2	0.0014514	-1.93596
218336_at	PFDN2	0.0154798	1.48144
218338_at	PHC1	0.0307559	1.26382
218346_s_at	SESN1	0.0000711	-3.69543
218347_at	TYW1	0.0005351	-2.42915
218351_at	COMMD8	0.0097966	-2.80164
218353_at	RGS5	0.0143228	-1.94302
218355_at	KIF4A	0.0000076	5.85705
218357_s_at	TIMM8B	0.0174569	1.68231
218361_at	GOLPH3L	0.0007282	-1.63874
218368_s_at	TNFRSF12A	0.0137591	1.38902
218370_s_at	S100PBP	0.0007545	-3.17026
218371_s_at	PSPC1	0.0043171	-2.95024
218375_at	NUDT9	0.0013918	-3.96059
218379_at	RBM7	0.0029126	-5.69097
218381_s_at	U2AF2	0.000193	2.0016
218385_at	MRPS18A	0.0017067	2.65054
218396_at	VPS13C	0.0074557	-2.13186
218401_s_at	ZNF281	0.0003251	-3.48406
218411_s_at	MBIP	0.001338	-3.39572
218412_s_at	GTF2IRD1	0.0000349	2.07878
218421_at	CERK	0.0000145	-3.35813
218422_s_at	RBM26	0.005157	-2.64698
218426_s_at	RNF216	0.0264071	1.30319
218427_at	SDCCAG3	0.0451992	1.25758
218428_s_at	REV1	0.0006551	-2.33633

218437_s_at	LZTFL1	0.0002979	-3.8635
218438_s_at	MED28	0.0147532	-2.44709
218443_s_at	DAZAP1	0.0006954	1.53459
218445_at	H2AFY2	0.0000459	2.38784
218446_s_at	FAM18B	0.0003529	-3.10837
218447_at	C16orf61	0.0655761	1.31003
218448_at	C20orf11	0.0847792	1.20982
218449_at	UFSP2	0.0000014	-7.16847
218450_at	HEBP1	0.0000012	-4.52872
218451_at	CDCP1	0.012313	-2.24414
218459_at	TOR3A	0.0033388	-1.8672
218462_at	RPF1	0.000162	-3.78277
218467_at	PSMG2	0.0037739	-2.39758
218470_at	YARS2	0.0001561	1.80079
218473_s_at	GLT25D1	0.0488679	1.18635
218474_s_at	KCTD5	0.0000006	1.4616
218480_at	AGBL5	0.0634523	1.31133
218486_at	KLF11	0.0004294	-2.36662
218487_at	ALAD	0.0000084	-4.72508
218491_s_at	THYN1	0.0191247	-3.02533
218492_s_at	THAP7	0.0539483	1.48171
218496_at	RNASEH1	0.0006842	-2.33415
218499_at	MST4	0	-17.6492
218501_at	ARHGEF3	0.0036326	-2.01644
218507_at	C7orf68	0.0656311	1.58937
218511_s_at	PNPO	0.0046622	-2.25052
218513_at	C4orf43	0.0019138	-3.96501
218514_at	C17orf71	0.001873	-2.34769
218515_at	GCFC1	0.0000263	-3.89332
218518_at	FAM13B	0.0000083	-5.75325
218519_at	SLC35A5	0.0001093	-3.88027
218528_s_at	RNF38	0.0010232	-4.04824
218531_at	TMEM134	0.0013574	1.56116
218532_s_at	FAM134B	0	-8.28923
218533_s_at	UCKL1	0.0066682	1.68905
218534_s_at	AGGF1	0.0000078	-3.03954
218535_s_at	RIOK2	0.0021277	-2.15926
218541_s_at	C8orf4	0.0085483	10.2431
218542_at	CEP55	0.0000702	10.0836
218545_at	CCDC91	0.0004192	-3.4248

218557_at	NIT2	0.0032452	-2.37932
218558_s_at	MRPL39	0.0089867	-2.85764
218561_s_at	LYRM4	0.0190336	1.59736
218565_at	C9orf114	0.0002175	1.58828
218569_s_at	KBTBD4	0.0005635	-1.74064
218570_at	KBTBD4///PTPI	0.0000609	-2.62595
218573_at	MAGEH1	0.0023961	-2.23058
218576_s_at	DUSP12	0.0107263	-1.99634
218583_s_at	DCUN1D1	0.0391211	1.52674
218584_at	TCTN1	0.0025373	-2.35329
218585_s_at	DTL	0.0000053	5.11274
218586_at	C20orf20	0.0002677	2.31135
218587_s_at	KTELC1	0.0001529	-2.05758
218588_s_at	FAM114A2	0.0004815	-1.84636
218589_at	LPAR6	0.0007299	-3.33094
218592_s_at	CECR5	0.079058	1.33744
218594_at	HEATR1	0.0092593	-1.9969
218598_at	RINT1	0.0068301	-2.45257
218599_at	REC8	0.0003384	-1.8735
218603_at	HECA	0.0011303	-2.44882
218604_at	LEMD3	0.004376	-2.40677
218606_at	ZDHHC7	0.0012453	-1.70628
218611_at	IER5	0.0008737	-2.1721
218616_at	INTS12	0.0001178	-3.67967
218619_s_at	SUV39H1	0.0005188	1.54879
218622_at	NUP37	0.0010073	-3.28781
218626_at	EIF4ENIF1	0.0016284	-1.9322
218628_at	CCDC53	0	-2.90413
218639_s_at	ZXDC	0.0394454	1.32044
218641_at	C11orf95	0.000904	-2.11142
218645_at	ZNF277	0.0000704	-6.59786
218646_at	C4orf27	0.0000607	-4.34969
218660_at	DYSF	0.0024204	1.61854
218662_s_at	NCAPG	0.0000007	8.94615
218669_at	RAP2C	0.0000656	-3.15428
218674_at	C5orf44	0.0111336	-2.80002
218676_s_at	PCTP	0.0119624	-2.28686
218677_at	S100A14	0.0089607	1.79358
218684_at	LRRC8D	0.0023563	1.888
218689_at	FANCF	0.0102192	-2.12059

218692_at	SYBU	0.0000009	-12.9348
218693_at	TSPAN15	0.0018996	1.53086
218694_at	ARMCX1	0.0000048	-4.9772
218699_at	RAB7L1	0.0000631	-2.31764
218703_at	SEC22A	0.0127504	-2.26313
218705_s_at	SNX24	0.0043917	-1.74276
218709_s_at	IFT52	0.0039566	-1.7844
218710_at	TTC27	0.0000038	-3.8122
218715_at	UTP6	0.0134115	-1.97446
218718_at	PDGFC	0.0001028	-3.55911
218719_s_at	GINS3	0.0142749	1.41948
218723_s_at	C13orf15	0.0674584	2.06336
218726_at	HJURP	0.0000302	2.85621
218729_at	LXN	0.0000013	-33.8159
218730_s_at	OGN	0.0000009	-20.982
218731_s_at	VWA1	0.0027197	2.31807
218736_s_at	PALMD	0.0000301	-3.92896
218738_s_at	RNF138	0.0085204	-2.84292
218749_s_at	SLC24A6	0.0428298	1.58687
218758_s_at	RRP1	0.0130686	1.93407
218761_at	RNF111	0.0030822	-2.09953
218769_s_at	ANKRA2	0.0019965	-2.13099
218773_s_at	MSRB2	0.0018008	-2.25274
218775_s_at	WWC2	0.0001597	-3.57973
218776_s_at	TMEM62	0.0290329	1.35761
218777_at	REEP4	0.0000483	1.91816
218784_s_at	C6orf64	0.0030778	-2.51864
218789_s_at	C11orf71	0.0872158	1.73219
218791_s_at	C15orf29	0.0075801	-2.14895
218792_s_at	BSPRY	0.0002695	2.55862
218802_at	CCDC109B	0.0000342	-3.23907
218803_at	CHFR	0.0207618	1.32977
218808_at	DALRD3	0.0113902	1.74892
218816_at	LRRC1	0.0018672	-2.1737
218823_s_at	KCTD9	0.0001357	-2.01932
218824_at	PNMAL1	0.000003	-7.07153
218827_s_at	CEP192	0.0035788	-2.41219
218829_s_at	CHD7	0.0058444	2.01836
218846_at	MED23	0.0163403	-1.9197
218847_at	IGF2BP2	0.0000226	5.2288

218852_at	PPP2R3C	0.0173458	-2.53263
218853_s_at	MOSPD1	0.0020035	-2.97663
218854_at	DSE	0	-19.7869
218862_at	ASB13	0.0733478	1.3637
218866_s_at	POLR3K	0.0776665	1.4046
218873_at	GON4L	0.0539373	1.21739
218877_s_at	TRMT11	0.0057996	-2.66001
218878_s_at	SIRT1	0.0010764	-2.71212
218880_at	FOSL2	0.0015232	2.23215
218883_s_at	MLF1IP	0.0001433	4.53622
218885_s_at	GALNT12	0.0039381	-3.76547
218886_at	PAK1IP1	0.0067777	1.6584
218887_at	MRPL2	0.0001294	2.13519
218888_s_at	NETO2	0.0333673	1.53998
218889_at	NOC3L	0.0004869	-2.75935
218901_at	PLSCR4	0.0000022	-22.957
218905_at	INTS8	0.0001495	-3.34908
218916_at	ZNF768	0.0120599	2.43645
218919_at	ZFAND1	0.0071927	-3.90134
218923_at	CTBS	0.0023258	-3.37807
218929_at	CDKN2AIP	0.0069176	-1.94552
218935_at	EHD3	0.0549427	1.51153
218937_at	ZNF434	0.0002102	-1.68519
218939_at	LETM1	0.0046009	1.49751
218942_at	PIP4K2C	0.073248	1.31515
218946_at	NFU1	0.0001178	-2.23358
218950_at	ARAP3	0.0000717	3.31526
218956_s_at	PTCD1	0.0034032	1.71063
218957_s_at	PAAF1	0.0281857	-2.38988
218962_s_at	TMEM168	0.0004821	-2.99065
218966_at	MYO5C	0.0374621	-2.18776
218970_s_at	CUTC	0.0000635	-2.75614
218973_at	EFTUD1	0.000078	-1.73273
218974_at	SOBP	0.0000533	-4.5092
218976_at	DNAJC12	0.004187	-3.432
218979_at	RMI1	0.0223902	1.69592
218983_at	C1RL	0.000152	-3.79132
218984_at	PUS7	0.0001861	2.56572
218986_s_at	DDX60	0.0041726	-5.12661
218987_at	ATF7IP	0.0121728	1.41866

218988_at	SLC35E3	0.079522	1.80555
218992_at	C9orf46	0.0164786	-2.03906
219000_s_at	DSCC1	0.0300583	1.93868
219001_s_at	DCAF10	0.0014183	-2.28416
219004_s_at	C21orf45	0.0053177	1.86793
219010_at	C1orf106	0	7.45891
219013_at	GALNT11	0.0009415	-2.55086
219017_at	ETNK1	0.0334769	2.61816
219019_at	LRDD	0.0189568	1.80432
219027_s_at	MYO9A	0.0000012	-1.99882
219033_at	PARP8	0.0007971	-2.2555
219035_s_at	RNF34	0.0048938	-1.8785
219038_at	MORC4	0.0000427	-3.4428
219048_at	PIGN	0.0046321	-1.75045
219049_at	CSGALNACT1	0	-25.4896
219054_at	C5orf23	0.0426699	1.70712
219059_s_at	LYVE1	0.000184	-2.24836
219061_s_at	LAGE3	0.0643988	1.94757
219064_at	ITIH5	0.0010073	5.42942
219065_s_at	DPY30///MEMO	0.0003107	2.32363
219067_s_at	NSMCE4A	0.0001665	-2.35108
219069_at	ANKRD49	0.006069	-3.00472
219070_s_at	MOSPD3	0.0296482	1.39883
219077_s_at	WWOX	0.0183708	-1.95128
219080_s_at	CTPS2	0.0016197	1.78651
219083_at	SHQ1	0.0334591	-2.02594
219093_at	PID1	0.0069188	-1.76331
219098_at	MYBBP1A	0.0149784	1.5774
219100_at	OBFC1	0.0000002	-2.15433
219109_at	SPAG16	0.0026851	-4.42242
219112_at	RAPGEF6	0.0006549	-1.95672
219115_s_at	IL20RA	0.0137689	-2.82361
219117_s_at	FKBP11	0.0000015	-6.40863
219124_at	C8orf41	0.0044818	-1.90245
219126_at	PHF10	0.0098764	-2.26727
219127_at	PRR15L	0.0000543	-3.5355
219128_at	C2orf42	0.0000266	-2.31836
219130_at	CCDC76	0.0144368	-2.98419
219137_s_at	MFF	0.0000806	-3.89379
219147_s_at	C9orf95	0.0000004	-6.91379

219148_at	PBK	0.0062981	3.05908
219151_s_at	RABL2A///RABI	0.0027504	-2.03478
219158_s_at	NAA15	0.017069	-2.15647
219164_s_at	ATG2B	0.0142834	-1.93991
219169_s_at	TFB1M	0.0022994	-1.68115
219172_at	UBTD1	0.0041049	2.00658
219174_at	IFT74	0.0004521	-3.14523
219183_s_at	CYTH4	0.0109093	1.41654
219187_at	FKBPL	0.0375242	1.44232
219192_at	UBAP2	0.0035646	1.7576
219202_at	RHBDF2	0.0008751	1.9402
219213_at	JAM2	0.0010598	-2.50159
219215_s_at	SLC39A4	0.0000039	5.64054
219216_at	ETAA1	0.0009646	-1.92703
219229_at	SLCO3A1	0.0001949	-3.30257
219237_s_at	DNAJB14	0.0087046	-2.5004
219244_s_at	MRPL46	0.0003307	-3.09495
219245_s_at	OGFOD2	0.0142622	1.70691
219250_s_at	FLRT3	0.0000062	-5.76467
219253_at	TMEM185B	0.0396347	1.32186
219263_at	RNF128	0.0000011	-14.7637
219266_at	ZNF350	0.0013379	-2.1878
219269_at	HMBOX1	0.0108297	-2.39132
219271_at	GALNT14	0.0012717	-2.25486
219281_at	MSRA	0.0001476	-2.62405
219283_at	C1GALT1C1	0.0069512	-2.41721
219288_at	C3orf14	0.0001784	-3.56087
219291_at	DTWD1	0.003657	-2.0176
219292_at	THAP1	0.0002318	-3.30948
219295_s_at	PCOLCE2	0	-8.88296
219297_at	WDR44	0.0006927	-2.94474
219303_at	RNF219	0.0057218	-2.65705
219304_s_at	PDGFD	0	-133.654
219306_at	KIF15	0.0078146	2.83058
219310_at	TMEM90B	0.0005208	2.33696
219313_at	GRAMD1C	0.0000156	-7.95454
219315_s_at	TMEM204	0.0603783	1.34788
219327_s_at	GPRC5C	0.0026159	1.90726
219329_s_at	C2orf28	0.0050437	-2.54225
219338_s_at	LRRC49	0.0002583	-4.12103

219342_at	CASD1	0.0000026	-9.07724
219350_s_at	DIABLO	0.0942092	1.20666
219352_at	HERC6	0.0064043	-1.78511
219353_at	NHLRC2	0.0051943	-1.98236
219355_at	CXorf57	0.0000005	-4.74732
219359_at	ATHL1	0.0012788	3.27497
219363_s_at	MTERFD1	0.0081626	-2.03762
219368_at	NAP1L2	0.0000001	-12.0122
219370_at	RPRM	0.0000045	-6.72215
219371_s_at	KLF2	0.000036	-2.39688
219373_at	DPM3	0.0079528	2.40585
219374_s_at	ALG9	0.0000327	-2.40684
219378_at	NAA16	0.0025046	-2.1372
219384_s_at	ADAT1	0.0003879	-1.6517
219389_at	SUSD4	0.0002071	2.12104
219394_at	PGS1	0.000168	1.80527
219401_at	XYLT2	0.0795357	1.30473
219402_s_at	DERL1	0.0424738	1.7901
219403_s_at	HPSE	0.0003231	-5.30819
219405_at	TRIM68	0.000001	-6.41644
219411_at	ELMO3	0.005456	1.22195
219412_at	RAB38	0	-11.5596
219421_at	TTC33	0.0004388	-2.97332
219427_at	FAT4	0.0000383	-4.19286
219440_at	RAI2	0.0327055	1.39266
219443_at	TASP1	0.016852	-2.76036
219444_at	BCORL1	0.024048	1.30163
219449_s_at	TMEM70	0.0022511	-4.61862
219469_at	DYNC2H1	0.0002631	-2.95071
219471_at	C13orf18	0.0045369	-1.98234
219476_at	C1orf116	0.0219051	2.75414
219481_at	TTC13	0.0020103	-2.61197
219482_at	SETD4	0.0493437	1.52333
219484_at	HCFC2	0.0027282	-1.69742
219485_s_at	PSMD10	0.0280861	-3.18615
219490_s_at	DCLRE1B	0.0423485	1.23592
219493_at	SHCBP1	0.0074201	1.63492
219495_s_at	ZNF180	0.0003956	-2.51575
219496_at	ANKRD57	0.0008948	-2.75327
219499_at	SEC61A2	0.0048268	2.02847

219501_at	ENOX1	0.0001494	-1.77184
219502_at	NEIL3	0.00411	1.55081
219505_at	CECR1	0.0088377	2.65966
219510_at	POLQ	0.0001796	2.93348
219511_s_at	SNCAIP	0.0000017	-5.51124
219531_at	CEP72	0.0169482	1.45727
219542_at	NEK11	0.027453	-2.10255
219544_at	C13orf34	0.023178	1.9208
219547_at	COX15	0.000066	-2.34846
219555_s_at	CENPN	0.0020146	2.13862
219564_at	KCNJ16	0.0262908	1.46927
219569_s_at	TMEM22	0.000154	-3.48792
219571_s_at	ZNF12	0.0010087	-2.63613
219572_at	CADPS2	0.0000048	-8.52315
219583_s_at	SPATA7	0.0000465	-4.30538
219602_s_at	FAM38B	0.0028991	-2.1662
219603_s_at	ZNF226	0.011334	-2.48933
219607_s_at	MS4A4A	0.0004422	-5.00604
219610_at	RGNEF	0.0000514	-2.59153
219622_at	RAB20	0.0306813	1.8408
219627_at	ZNF767	0.0270946	1.47749
219632_s_at	TRPV1	0.0005602	1.69868
219641_at	DET1	0.0000197	-2.18147
219643_at	LRP1B	0.000284	-2.60951
219644_at	CCDC41	0.0017402	-3.54072
219648_at	MREG	0.018922	1.78125
219650_at	ERCC6L	0.0002608	2.9947
219654_at	PTPLA	0.0000001	-12.5081
219661_at	RANBP17	0.0002442	1.41032
219667_s_at	BANK1	0.0054749	-1.77727
219670_at	BEND5	0.0023934	-2.09841
219674_s_at	HDLBP	0.0531137	1.64979
219676_at	ZSCAN16	0.0049153	1.93066
219677_at	SPSB1	0.0000401	1.92664
219682_s_at	TBX3	0.0001498	-2.37589
219683_at	FZD3	0.0167562	1.83412
219686_at	STK32B	0.0064648	-2.22434
219690_at	TMEM149	0.0001577	1.84062
219695_at	SMPD3	0.0005537	-1.83615
219703_at	MNS1	0.0321913	1.86881

219711_at	ZNF586	0.001621	-2.63463
219736_at	TRIM36	0.0043148	-1.75474
219738_s_at	PCDH9	0.0000001	-7.1448
219747_at	C4orf31	0	-20.3933
219756_s_at	POF1B	0.0265608	1.36977
219764_at	FZD10	0.0000825	6.25788
219765_at	ZNF329	0.0426955	-2.36651
219768_at	VTCN1	0.0394951	3.1761
219771_at	TBC1D8B	0.0000639	-2.89636
219773_at	NOX4	0.0022855	4.64073
219778_at	ZFPM2	0	-33.3942
219787_s_at	ECT2	0.0000288	8.9801
219789_at	NPR3	0.0012445	1.45689
219791_s_at	NBLA00301	0	-6.87406
219792_at	AGMAT	0.0074634	1.48564
219797_at	MGAT4A	0.0457325	1.48586
219798_s_at	MEPCE	0.0382246	1.33303
219802_at	PYROXD1	0.0048664	-3.55948
219818_s_at	GPATCH1	0.0071124	-1.98313
219821_s_at	GFOD1	0.0042171	2.53702
219833_s_at	EFHC1	0.0001557	-3.13017
219836_at	ZBED2	0.0816187	2.60522
219848_s_at	ZNF432	0.0062941	-2.01075
219855_at	NUDT11	0.0000156	-2.75166
219857_at	C10orf81	0.0647914	1.60286
219863_at	HERC5	0.000071	-5.93788
219867_at	CHODL	0.0211855	1.96502
219869_s_at	SLC39A8	0.0000001	-2.79227
219870_at	ATF7IP2	0.0000002	-4.47064
219871_at	FLJ13197	0.0200602	-1.98647
219874_at	SLC12A8	0.0532333	1.49694
219888_at	SPAG4	0.0057881	1.62248
219895_at	FAM70A	0	-42.7345
219905_at	ERMAP	0.0039001	-2.06427
219906_at	FLJ10213	0.0000121	-3.89362
219913_s_at	CRNKL1	0.0012895	-3.44518
219930_at	KLF8	0.0087971	1.39529
219933_at	GLRX2	0.0427621	1.4175
219936_s_at	GPR87	0.0350915	1.24533
219961_s_at	PLK1S1	0.000032	-4.73324

219972_s_at	C14orf135	0.0000939	-3.31287
219976_at	HOOK1	0.0000417	-3.8899
219990_at	E2F8	0.0039692	4.6266
220011_at	C1orf135	0.0001765	1.60797
220030_at	STYK1	0.032434	1.92942
220036_s_at	LMBR1L	0.033392	1.57785
220051_at	PRSS21	0.0014052	1.58079
220054_at	IL23A	0.0217716	1.26042
220060_s_at	C12orf48	0.0000993	2.24617
220094_s_at	CCDC90A	0.0201874	1.72472
220104_at	ZC3HAV1	0.0328682	1.48518
220111_s_at	ANO2	0.0457864	1.31257
220137_at	VSIG10	0.0114881	1.5956
220149_at	C2orf54	0.0101502	1.41312
220155_s_at	BRD9	0.0001061	1.58233
220161_s_at	EPB41L4B	0.0005126	-3.78498
220162_s_at	CARD9	0.0000366	1.52967
220167_s_at	TP53TG3///TP5	0.0335931	2.60381
220173_at	C14orf45	0.001468	-2.06731
220175_s_at	CBWD1///CBW	0.000886	-2.10217
220180_at	CCDC68	0.0000227	-5.20386
220183_s_at	NUDT6	0.0057821	-2.4625
220184_at	NANOG	0.0022424	-5.08682
220196_at	MUC16	0.0051553	3.53155
220255_at	FANCE	0.000009	2.05317
220260_at	TBC1D19	0.0011575	-2.08441
220261_s_at	ZDHHC4	0.0024512	-1.91182
220287_at	ADAMTS9	0.0987877	1.12373
220288_at	MYO15A	0.0181614	1.37076
220321_s_at	CCDC121	0.0033967	-1.9516
220327_at	VGLL3	0.000025	-3.9233
220330_s_at	SAMSN1	0.069617	-2.69767
220348_at	KLHL29	0.0460087	1.23784
220356_at	CORIN	0.0482535	1.2338
220391_at	ZBTB3	0.008041	-2.37154
220397_at	MDM1	0.0085249	-1.79584
220419_s_at	USP25	0.0003345	-3.151
220424_at	NPHS2	0.0064085	-2.1405
220446_s_at	CHST4	0.0000195	-1.64557
220474_at	SLC25A21	0.0000499	-2.28278

220525_s_at	AUP1	0.0013639	2.01788
220540_at	KCNK15	0.0862984	2.07682
220543_at	C21orf62	0.0000002	-8.59039
220556_at	ATP1B4	0.0955357	1.23102
220595_at	PDZRN4	0.013902	-1.93872
220597_s_at	ARL6IP4	0.0780705	1.30481
220603_s_at	MCTP2	0.0000062	-5.59574
220647_s_at	CHCHD8	0.0370849	1.3334
220661_s_at	ZNF692	0.0001606	1.67305
220664_at	SPRR2C	0.0004078	-1.69905
220668_s_at	DNMT3B	0.0000047	2.06641
220741_s_at	PPA2	0.0008384	-4.08857
220742_s_at	NGLY1	0.0135338	-1.88104
220745_at	IL19	0.0806682	1.40124
220746_s_at	UIMC1	0.0002881	-2.83811
220751_s_at	C5orf4	0.0000006	-2.22963
220761_s_at	TAOK3	0	-5.12363
220770_s_at	C5orf54	0.0001023	-4.74842
220776_at	KCNJ14	0.02627	1.30895
220777_at	KIF13A	0.002342	-3.04774
220917_s_at	WDR19	0.000042	-3.06157
220922_s_at	SPANXA1///SPA	0.0000512	-3.35471
220925_at	NAA35	0.0229446	-2.73213
220933_s_at	ZCCHC6	0.0037061	-2.28784
220947_s_at	TBC1D10B	0.0202074	1.73667
220948_s_at	ATP1A1	0.0029205	-2.26455
220949_s_at	C7orf49	0.0104131	1.46521
220983_s_at	SPRY4	0.0613275	1.27878
220987_s_at	C11orf17///NUA	0.0001445	2.74983
220992_s_at	C1orf25	0.0010485	-2.29625
221004_s_at	ITM2C	0.0001164	3.75765
221007_s_at	FIP1L1	0.0010531	-1.8607
221008_s_at	AGXT2L1	0.0020012	-2.38736
221014_s_at	RAB33B	0.0080686	-2.90651
221019_s_at	COLEC12	0.0000004	-4.66217
221027_s_at	PLA2G12A	0.0010514	-2.13082
221031_s_at	APOLD1	0.0002904	4.42539
221044_s_at	TRIM34///TRIM	0.0000639	-2.17164
221045_s_at	PER3	0.0000072	-3.37128
221082_s_at	NDRG3	0.0153384	1.6523

221135_s_at	ASTE1	0.0011338	-1.94344
221185_s_at	IQCG	0.0098144	-3.25151
221193_s_at	ZCCHC10	0.0232396	-2.0246
221203_s_at	YEATS2	0.0530649	1.52702
221204_s_at	CRTAC1	0.0000382	2.89107
221210_s_at	NPL	0.0410325	-2.26734
221216_s_at	SCMH1	0.0769372	1.30493
221218_s_at	TPK1	0.0031096	-3.14025
221221_s_at	KLHL3	0.0394109	1.37734
221229_s_at	TRMT61B	0.0162848	-1.93856
221234_s_at	BACH2	0.0003334	-2.16527
221255_s_at	TMEM93	0.0290448	1.3213
221265_s_at	C15orf44	0.0027008	-1.84509
221276_s_at	SYNC	0.0004318	-1.731
221277_s_at	PUS3	0.0008773	-2.01245
221306_at	GPR27	0.0576917	1.3219
221319_at	PCDHB8	0.0158711	1.30937
221381_s_at	MORF4///MORI	0.0003041	-3.44707
221449_s_at	ITFG1	0.02043	-2.33781
221452_s_at	TMEM14B	0.0271206	-3.59934
221476_s_at	RPL15	0.0000077	-4.66529
221477_s_at	SOD2	0.0000489	-4.91765
221479_s_at	BNIP3L	0.0068297	-2.70131
221480_at	HNRNPD	0.0001091	-3.36622
221482_s_at	ARPP19	0.0561641	-2.48277
221485_at	B4GALT5	0.0808885	1.87263
221492_s_at	ATG3	0.0003475	-3.85572
221493_at	TSPYL1	0.0000991	-4.22534
221498_at	SNX27	0.0343595	1.44744
221500_s_at	STX16	0.0573771	1.25011
221502_at	KPNA3	0.0011928	-3.7622
221516_s_at	SMCR7L	0.0147936	-2.44654
221521_s_at	GINS2	0.0003187	2.95361
221538_s_at	PLXNA1	0.0023214	2.36453
221542_s_at	ERLIN2	0.0002991	-2.05004
221549_at	GRWD1	0.0309805	1.50496
221559_s_at	MIS12	0.0007288	-2.33832
221568_s_at	LIN7C	0.0112966	-2.14993
221569_at	AHI1	0.0037348	-2.64672
221582_at	HIST3H2A	0.0095696	1.98094

221584_s_at	KCNMA1	0.0082924	2.59924
221591_s_at	FAM64A	0.0035401	1.992
221600_s_at	C11orf67	0.0007389	-2.18202
221601_s_at	FAIM3	0.000016	-2.84801
221605_s_at	PIPOX	0.0314107	2.74417
221606_s_at	HMGN5	0.0088914	-2.13704
221616_s_at	TAF9B	0.0013697	-1.66441
221621_at	C17orf86	0.0651597	1.17879
221622_s_at	TMEM126B	0.0070955	-4.51158
221641_s_at	ACOT9	0.0000007	-4.62671
221654_s_at	USP3	0.0027005	-2.14742
221656_s_at	ARHGEF10L	0.0006288	1.49109
221667_s_at	HSPB8	0.0020223	-1.66603
221675_s_at	CHPT1	0.0213009	-2.09799
221677_s_at	DONSON	0.0148062	1.69971
221681_s_at	DSPP	0.0947854	1.98819
221683_s_at	CEP290	0.0171264	-2.15424
221689_s_at	PIGP	0.0119996	-1.94516
221699_s_at	DDX50	0.0012006	-3.23879
221718_s_at	AKAP13	0.0096332	-2.49065
221727_at	SUB1	0.0001786	-3.61498
221729_at	COL5A2	0.020398	2.11248
221738_at	RALGAPB	0.0747776	1.51911
221748_s_at	TNS1	0.0071275	1.86042
221756_at	PIK3IP1	0	-8.36937
221760_at	MAN1A1	0.0000188	-5.4117
221771_s_at	MPHOSPH8	0.0003825	-2.68604
221776_s_at	BRD7	0.0137152	-2.81033
221787_at	C6orf120	0.0001206	-2.87548
221791_s_at	CCDC72	0.0631531	1.27143
221803_s_at	NRBF2	0.0359702	-2.05362
221804_s_at	FAM45A///FAM4	0.0000191	-2.44905
221808_at	RAB9A	0.0000208	-4.8315
221825_at	ANGEL2	0.0033201	-1.95966
221832_s_at	LUZP1	0.018027	-2.01184
221833_at	LONP2	0.0008194	-2.5069
221836_s_at	TRAPPC9	0.0051702	1.73753
221840_at	PTPRE	0.0009906	-2.4685
221841_s_at	KLF4	0.0002057	-5.16232
221843_s_at	KIAA1609	0.0007316	-1.97915

221858_at	TBC1D12	0.0004096	-2.74985
221880_s_at	FAM174B	0.0000763	2.31867
221898_at	PDPN	0.0000165	-6.32641
221906_at	TXNRD3	0.0021361	-1.82625
221918_at	CDK17	0.016851	-2.05142
221921_s_at	CADM3	0.0840014	1.56704
221922_at	GPSM2	0.0135447	3.0493
221927_s_at	ABHD11	0.0000005	2.77093
221933_at	NLGN4X	0.0032944	-9.98288
221935_s_at	C3orf64	0.0014237	-2.33363
221970_s_at	NOL11	0.0064111	-2.4271
221984_s_at	FAM134A	0.0005328	2.09334
221992_at	NPIPL2	0.0024361	2.26034
222018_at	NACA	0.0020562	3.09834
222028_at	ZNF45	0.0001727	-2.48217
222036_s_at	MCM4	0.000018	3.03541
222039_at	KIF18B	0.0000923	5.95248
222040_at	HNRNPA1	0.0235513	2.2984
222047_s_at	SRRT	0.0007163	1.84461
222062_at	IL27RA	0.0123296	1.61274
222077_s_at	RACGAP1	0.000051	7.25573
222103_at	ATF1	0.0019256	-2.22643
222108_at	AMIGO2	0.0002501	-6.39262
222112_at	EPS15L1	0.0104404	1.33408
222119_s_at	FBXO11	0.000685	-3.01904
222127_s_at	EXOC1	0.0013406	-3.21616
222139_at	KIAA1466	0.0001125	4.68018
222155_s_at	GPR172A	0.0011327	1.35992
222161_at	NAALAD2	0.0025339	-1.79311
222163_s_at	SPATA5L1	0.0077655	-2.27171
222182_s_at	CNOT2	0.0046186	-1.74763
222190_s_at	C16orf58	0.0579871	1.36552
222195_s_at	C9orf156	0.0024658	-1.78587
222201_s_at	CASP8AP2	0.055112	-2.44102
222203_s_at	RDH14	0.0015345	-1.98895
222209_s_at	TMEM135	0.008106	-2.89206
222219_s_at	TLE6	0.0381527	1.28274
222230_s_at	ACTR10	0.0000991	-4.20754
222231_s_at	LRRC59	0.0069093	-2.95166
222235_s_at	CSGALNACT2/	0.0275506	-2.31666

222237_s_at	ZFP112	0.0001442	-4.62597
222244_s_at	TUG1	0.0000003	-5.83861
222258_s_at	SH3BP4	0.0029187	-2.02375
222266_at	C19orf2	0.0484375	-2.72881
222275_at	MRPS30	0.0103086	-1.86731
222360_at	DPH5	0.0004587	-2.98756
222361_at	TUBBP5	0.0088364	2.85098
222369_at	NAA40	0.0012252	1.93533
222381_at	PDCD6	0.0917232	1.3716
222389_s_at	WAC	0.0021505	-2.83292
222391_at	TMEM30A	0.0129092	-1.92135
222396_at	HN1	0.0727991	1.4558
222401_s_at	TMEM50A	0.0001836	-4.13268
222403_at	MTCH2	0.0044585	-2.05082
222406_s_at	PNRC2	0.007066	-1.93689
222424_s_at	NUCKS1	0.0357875	1.52153
222426_at	MAPKAP1	0.0005544	-2.11458
222430_s_at	YTHDF2	0.0001682	-3.54772
222433_at	ENAH	0.0795402	1.594
222438_at	MED4	0.0025192	-2.6921
222440_s_at	THRAP3	0.0633657	1.21412
222444_at	ARMCX3	0.0122239	-1.82417
222445_at	SLC39A9	0.0026287	-1.81605
222446_s_at	BACE2	0.0003911	2.89614
222447_at	METTL9	0.0008763	-2.98482
222451_s_at	ZDHHC9	0.0684399	1.65861
222457_s_at	LIMA1	0.0000263	-5.31126
222464_s_at	C10orf119	0.002126	-2.01777
222468_at	KIAA0319L	0.0438467	1.46212
222475_at	SAP30BP	0.0888337	1.24
222478_at	VPS36	0.0011951	-2.2625
222481_at	FXC1	0.0097168	-1.94274
222484_s_at	CXCL14	0.0023339	3.03727
222486_s_at	ADAMTS1	0.0058608	1.50907
222488_s_at	DCTN4	0.0022278	-2.13242
222495_at	TMEM167B	0.0000136	-4.48424
222503_s_at	WDR41	0.0000151	-2.20376
222513_s_at	SORBS1	0.0001528	2.40695
222514_at	RRAGC	0.0151797	-2.16532
222517_at	AP3M1	0.0085423	-2.14761

222525_s_at	CCDC25	0.001516	-1.82751
222531_s_at	MUDENG	0.0028406	-3.29515
222537_s_at	CDC42SE1	0.0001804	2.0074
222538_s_at	APPL1	0.0090456	-2.04845
222557_at	STMN3	0.0010621	-2.78187
222559_s_at	RPRD1A	0.0004446	-3.41295
222560_at	LANCL2	0.0531022	1.29627
222566_at	SUV420H1	0.0049796	-1.8964
222574_s_at	DHX40	0.0000437	-2.97447
222575_at	SETD5	0.0211061	1.52914
222579_at	UBA5	0.0062664	-3.30523
222584_at	MSTO1	0.0214075	1.40377
222588_s_at	C11orf57	0.0058832	-2.42164
222594_s_at	SPATS2	0.0101858	1.30297
222597_at	SNAP29	0.0004797	-2.18617
222600_s_at	UBA6	0.0612609	-2.41664
222603_at	ERMP1	0.0001672	-3.63513
222606_at	ZWILCH	0.0161063	1.63235
222615_s_at	PRKRIP1	0.032142	1.5192
222616_s_at	USP16	0.0103299	-2.90357
222617_s_at	C10orf84	0.0149629	-1.99212
222621_at	DNAJC1	0.0018607	-2.24527
222630_at	RFX7	0.0097681	-2.58457
222640_at	DNMT3A	0.0000176	2.91331
222647_at	SLC35C1	0.0012106	1.64687
222659_at	IPO11	0.0006803	-1.92621
222667_s_at	ASH1L	0.0084965	-4.41308
222668_at	KCTD15	0.0035319	-2.79648
222669_s_at	SBDS///SBDSP	0.0003881	-3.05147
222683_at	RNF20	0.0058092	-2.49933
222684_s_at	NOL10	0.0004623	2.10773
222691_at	SLC35B3	0.0075665	-4.1921
222692_s_at	FNDC3B	0.0278258	2.37051
222696_at	AXIN2	0.0111887	-1.85288
222701_s_at	CHCHD7	0.0012349	3.7007
222708_s_at	STX17	0.0311259	-2.47394
222717_at	SDPR	0.0000003	-10.4052
222721_at	CNIH4	0.0021417	-2.00217
222730_s_at	ZDHHC2	0.0008449	-2.67797
222740_at	ATAD2	0.0156197	3.28348

222747_s_at	SCML1	0.0032697	-2.59384
222761_at	BIVM	0.0000021	-5.14497
222764_at	ASRGL1	0.0007928	3.09868
222781_s_at	C9orf40	0.0051287	1.61356
222787_s_at	TMEM106B	0.0043304	-3.52813
222798_at	PTER	0.0192526	-1.8897
222799_at	WDR91	0.0009751	1.67544
222805_at	MANEA	0.0023875	-2.89381
222808_at	ALG13	0.0021501	-2.44778
222811_at	FTSJD1	0.0005606	-4.11114
222843_at	FIGNL1	0.0163877	-1.9991
222847_s_at	EGLN3	0.0741076	1.7493
222848_at	CENPK	0.0000824	4.72647
222857_s_at	KCNMB4	0.0078796	-1.8904
222869_s_at	ELAC1	0.0003003	-1.96549
222870_s_at	B3GNT2	0.0299229	-2.69377
222871_at	KLHDC8A	0.0004101	-9.51481
222875_at	DHX33	0.010353	-1.95999
222893_s_at	RPAP2	0.001645	-2.13581
222900_at	NRIP3	0.0861883	1.23196
222906_at	FLVCR1	0.002164	-1.68227
222910_s_at	PEX5L	0.0000003	-10.3017
222912_at	ARRB1	0.0064797	-2.34209
222921_s_at	HEY2	0.0000618	3.2519
222924_at	SLMAP	0.0000155	-2.31281
222925_at	DCDC2	0.0072097	5.11383
222931_s_at	THNSL1	0.0002599	-2.84739
222958_s_at	DEPDC1	0.0015947	5.83128
222959_at	CNGB3	0.0000001	-6.70849
222975_s_at	CSDE1	0.0002723	-2.73892
222981_s_at	RAB10	0.0685522	1.6558
222984_at	PAIP2	0.000028	-5.30197
222986_s_at	SHISA5	0.0115425	1.71423
222988_s_at	TMEM9	0.0646967	1.23091
222989_s_at	UBQLN1	0.0056954	1.67902
222994_at	PRDX5	0.0749031	1.44178
222999_s_at	CCNL2	0.0147968	1.6055
223001_at	OSTC	0.0005907	-6.17565
223003_at	C19orf43	0.0859922	1.885
223008_s_at	C9orf5	0.0000845	-4.2163

223011_s_at	OCIAD1	0.0040738	-2.11233
223015_at	EIF2A	0.0011826	-2.28914
223017_at	TXNDC12	0.0037262	-2.48566
223022_s_at	VTA1	0.0000539	-2.66335
223026_s_at	VPS29	0.0481481	-2.34885
223028_s_at	SNX9	0.0004014	-5.72862
223031_s_at	TRAF7	0.0422031	1.38897
223039_at	C22orf13	0.0021672	-2.79427
223040_at	NAA20	0.0367986	-2.40318
223042_s_at	FUNDC2	0.0023841	-3.41348
223043_at	TMEM85	0.0009405	-2.79797
223044_at	SLC40A1	0.0008338	-3.40052
223046_at	EGLN1	0.0001584	-3.83432
223056_s_at	XPO5	0.0619224	1.27566
223059_s_at	FAM107B	0.0351952	-3.02399
223062_s_at	PSAT1	0	37.4878
223063_at	C1orf198	0.0011438	-1.96569
223068_at	EML4	0.000186	-4.41819
223070_at	SELK	0.0010318	-2.42664
223071_at	IER3IP1	0.0033585	-2.19361
223075_s_at	AIF1L	0.0000001	19.2343
223076_s_at	NSUN2	0.0408382	1.4926
223077_at	TMOD3	0.0088024	-2.12414
223084_s_at	CCNDBP1	0.0000008	-7.95766
223087_at	ECHDC1	0.000055	-7.58371
223089_at	VEZT	0.0332179	-2.46412
223104_at	JAGN1	0.0003822	-1.8986
223107_s_at	ZCCHC17	0.0001341	-2.35227
223110_at	KIAA1429	0.0003114	-2.6777
223114_at	COQ5	0.0115096	-1.98029
223115_at	MED17	0.0000342	-1.78897
223119_s_at	USP47	0.0000118	-2.80084
223122_s_at	SFRP2	0.0000083	-18.3962
223126_s_at	C1orf21	0.0007968	-2.34896
223132_s_at	TRIM8	0.0002805	2.60461
223139_s_at	DHX36	0.0020858	-6.5992
223144_s_at	AKIRIN2	0.0580369	1.34813
223154_at	MRPL1	0.0085213	-2.65154
223155_at	HDHD2	0.000029	-3.86871
223156_at	MRPS23	0.0071217	-2.09788

223157_at	C4orf14	0.0041634	-2.17066
223159_s_at	NEK6	0.0152948	1.57996
223165_s_at	IP6K2	0.0010518	2.55897
223168_at	RHOU	0.0001542	-4.39062
223170_at	TMEM98	0.0000023	-5.59529
223171_at	DYM	0.0037199	-1.77947
223172_s_at	MTP18	0.0213993	1.56596
223174_at	BTBD10	0.0065085	-1.89067
223176_at	KCTD20	0.0100233	-1.82434
223177_at	NT5DC1	0.0072987	-3.57759
223181_at	C18orf55	0.0015673	-2.17941
223187_s_at	ORMDL1	0.0243471	-1.9804
223190_s_at	MLL5	0.0182896	-2.52768
223192_at	SLC25A28	0.0473243	1.24919
223197_s_at	SMARCAD1	0.000591	-2.50659
223202_s_at	TMEM164	0.0126558	1.81039
223203_at	FAM156A///FAM	0.0183616	1.296
223208_at	KCTD10	0.0018635	1.67682
223209_s_at	SELS	0.0036392	-3.1817
223211_at	HACL1	0.0000467	-3.56355
223213_s_at	ZHX1	0.0037965	-2.86304
223218_s_at	NFKBIZ	0.0068477	-2.00207
223220_s_at	PARP9	0.0276431	-2.34354
223223_at	ARV1	0.0000379	-4.30957
223227_at	BBS2	0.000017	-5.49334
223229_at	UBE2T	0.0003264	2.08729
223232_s_at	CGN	0.0010457	-2.37514
223235_s_at	SMOC2	0.0003291	-4.93036
223236_at	CCDC55	0.0009379	-3.26579
223239_at	C14orf129	0.0018279	-2.878
223240_at	FBXO8	0.0000091	-3.17583
223249_at	CLDN12	0.0001576	-2.93567
223250_at	KLHL7	0.0000156	-7.7626
223252_at	HDGFRP2	0.0459698	1.37677
223254_s_at	G2E3	0.0205002	-2.45041
223261_at	POLK	0.0000029	-5.89739
223264_at	MESDC1	0.0023014	1.88745
223265_at	SH3BP5L	0.0050199	1.49868
223266_at	STRADB	0	-3.83529
223267_at	RG9MTD1	0.0195319	-2.00652

223269_at	POLR3GL	0.000001	-4.4846
223275_at	PRMT6	0.0069751	-2.26912
223288_at	USP38	0.0050624	-3.26452
223292_s_at	MRPS15	0.0344992	1.81034
223294_at	CXorf26	0.0036076	-2.27598
223295_s_at	LUC7L	0.0063515	2.14074
223296_at	SLC25A33	0.0148807	2.43014
223298_s_at	NT5C3	0.0245547	-2.73691
223301_s_at	CCDC82	0.0229713	-2.08458
223305_at	TMEM216	0.029064	-2.10572
223306_at	EBPL	0.0003161	-4.11944
223307_at	CDCA3	0.0000001	17.9213
223315_at	NTN4	0.0006594	-10.6254
223319_at	GPHN	0.0001729	-2.33933
223330_s_at	SUGT1	0.0131385	-2.78473
223334_at	TMEM126A	0.0163489	-2.59253
223340_at	ATL1	0	-8.13963
223342_at	RRM2B	0.0024857	-2.04365
223343_at	MS4A7	0.0352175	-2.42977
223351_at	C17orf80	0.0001155	-2.27696
223356_s_at	MTIF3	0.0000678	-1.89344
223361_at	C6orf115	0.0234783	-2.13302
223363_at	PSMG3	0.0055978	1.55147
223365_at	DHX37	0.0178975	1.609
223381_at	NUF2	0.000783	4.10408
223384_s_at	TRIM4	0.0000021	-14.0173
223387_at	ZFYVE1	0.0001121	-1.9199
223391_at	SGPP1	0.0008652	-3.12968
223392_s_at	TSHZ3	0.0002208	-2.25711
223395_at	ABI3BP	0.0000002	-15.4005
223396_at	TMEM60	0.0028463	-2.54539
223401_at	C17orf48	0.0000038	-2.6873
223402_at	DUSP23	0.0466441	1.48797
223412_at	KBTBD7	0.0000074	-4.79621
223414_s_at	LYAR	0.008423	1.73752
223421_at	CYHR1	0.0045046	1.59285
223423_at	GPR160	0.0688862	3.32699
223424_s_at	ZSCAN21	0.0006516	-1.81617
223433_at	C7orf36	0.0017335	-2.2779
223434_at	GBP3	0.0108319	-4.14455

223439_at	NKAP	0.010856	-1.99184
223441_at	SLC17A5	0.0000014	-4.2435
223443_s_at	AMZ2P1	0.000033	-2.52777
223444_at	SENP7	0.0018197	-2.19251
223450_s_at	COG3	0.001545	-2.49447
223454_at	CXCL16	0.0848576	1.50016
223461_at	TBC1D7	0.018858	2.03002
223465_at	COL4A3BP	0.0404749	-2.4719
223467_at	RASD1	0.0568899	1.77028
223468_s_at	RGMA	0.038279	1.32241
223473_at	MPV17L2	0.0274799	1.33894
223475_at	CRISPLD1	0.0353356	-2.04469
223497_at	FAM135A	0.009777	-2.36511
223501_at	TNFSF13B	0	-16.2398
223503_at	TMEM163	0.0002028	-3.22506
223504_at	DNAJC27	0.0049938	-2.26759
223508_at	NOTCH1	0.043719	1.89645
223526_at	C18orf21	0.0035858	-2.36635
223529_at	SYT4	0.0000005	-20.7551
223530_at	TDRKH	0.0135464	1.23412
223535_at	NUDT12	0.0053792	-1.75062
223547_at	JKAMP	0.0000767	-3.07799
223556_at	HELLS	0.0170731	2.20555
223570_at	MCM10	0.0001949	1.85578
223575_at	KIAA1549	0.0775739	1.24036
223576_at	C6orf203	0.0027043	-2.42643
223580_at	SPSB2	0.0036466	2.04595
223590_at	ZNF700	0.0060721	-1.95966
223597_at	ITLN1	0	-625.457
223599_at	TRIM6	0.0000001	-4.18596
223604_at	GARNL3	0.0000023	-2.48799
223620_at	GPR34	0.0021043	-2.45472
223623_at	C2orf40	0.0000177	-22.56
223637_s_at	FAM160A2	0.0912845	1.30136
223638_at	NBPF3	0.0200948	1.37302
223652_at	AS3MT	0.000027	-4.05744
223654_s_at	CELF4	0.0053924	1.49697
223666_at	SNX5	0.0003037	2.0537
223685_s_at	PRPF18	0.0007471	1.6747
223687_s_at	LY6K	0.0523715	2.55231

223691_at	RGS22	0.0106072	-2.12654
223700_at	MND1	0.0042104	2.66549
223704_s_at	DMRT2	0.0000033	-5.66005
223705_s_at	GPBP1	0.0021552	-2.40859
223727_at	KCNIP2	0.0402648	1.18841
223734_at	C4orf49	0	-61.3212
223738_s_at	PGM2	0.0002706	-1.60003
223748_at	SLC4A11	0	38.6874
223753_s_at	CFC1///CFC1B	0.0125832	-3.79714
223775_at	HHIP	0.0015043	-1.79033
223784_at	TMEM27	0.0034167	-2.08611
223798_at	SLC41A2	0.0001712	-3.21591
223800_s_at	LIMS3	0.0001234	5.57786
223802_s_at	RBBP6	0.0000308	-3.05637
223805_at	OSBPL6	0.0048019	-1.73763
223824_at	RNLS	0.000208	-1.79681
223833_at	WDR55	0.0000012	1.95837
223843_at	SCARA3	0.0157027	4.37666
223874_at	ACTR3C	0.0984344	1.31198
223876_at	SPATA16	0.0097841	1.3182
223883_s_at	STK31	0	-32.3302
223886_s_at	RNF146	0.0002384	-4.05059
223892_s_at	TMBIM4	0.0002239	-3.29814
223907_s_at	PINX1	0.0458318	1.36227
223949_at	TMPRSS3	0.0124306	3.08927
223980_s_at	SP110	0.0018692	-3.12997
223982_s_at	PNPLA8	0.0089592	-2.19915
223991_s_at	GALNT2///LOC	0.0000094	2.57889
223995_at	SLC12A9	0.0527972	1.16811
224000_at	C2orf16	0.0085136	1.33658
224002_s_at	FKBP7	0.0062787	-1.94163
224049_at	KCNK17	0.0408171	1.1634
224061_at	INMT	0.0019738	-1.7233
224097_s_at	F11R	0.0095459	1.95086
224145_s_at	SPTBN4	0.0505742	1.45774
224164_at	TPM3	0.0020435	1.31647
224209_s_at	GDA	0.0991356	2.58073
224279_s_at	CABYR	0.0002699	1.59299
224321_at	TMEFF2	0.0000821	4.30085
224325_at	FZD8	0.0001078	-2.71263

224332_s_at	MRPL43	0.0295447	1.37323
224339_s_at	ANGPTL1	0.0011702	-1.8041
224364_s_at	PPIL3	0.0370271	-2.22822
224365_s_at	TIGD7	0.0006856	-2.62904
224366_s_at	REPS1	0.0024779	-2.11964
224367_at	BEX2	0.0104947	-2.52756
224370_s_at	CAPS2	0.0024223	-2.28687
224374_s_at	EMILIN2	0.0000195	-3.07624
224387_at	COMMD5	0.0291014	1.9702
224413_s_at	TM2D2	0.0000266	-3.45963
224428_s_at	CDCA7	0.0031106	4.74728
224430_s_at	MTO1	0.0000863	-3.09149
224431_s_at	SUV420H2	0.042924	1.3896
224444_s_at	C1orf97	0.0016781	2.07498
224445_s_at	ZFYVE21	0.0009254	-2.49632
224446_at	LLPH	0.0163516	-1.89011
224447_s_at	C17orf37	0.0653634	1.62738
224448_s_at	C6orf125	0.0145029	1.52765
224452_s_at	C7orf70	0.0000045	-4.38882
224463_s_at	C11orf70	0.0231322	-2.62486
224465_s_at	WIBG	0.000305	1.94478
224471_s_at	BTRC	0.000109	-2.8391
224477_s_at	NUDT16L1	0.0374894	1.60534
224480_s_at	AGPAT9	0.0005235	-1.78705
224481_s_at	HECTD1	0.0003673	-2.31775
224489_at	KIAA1267///LO(	0.0111086	-1.83502
224512_s_at	LSMD1	0.0027235	3.07793
224513_s_at	UBQLN4	0.0028064	1.82792
224516_s_at	CXXC5	0.0000002	9.80199
224518_s_at	ZNF559	0.0019189	-3.09917
224521_s_at	CCDC77	0.0055143	1.35991
224524_s_at	ASB3///GPR75/	0.0004939	-4.85251
224558_s_at	MALAT1	0.0186733	-2.28739
224560_at	TIMP2	0.0047251	-3.31854
224564_s_at	RTN3	0.0002238	2.44125
224569_s_at	IRF2BP2	0.0607793	1.39924
224576_at	ERGIC1	0.0739653	1.80861
224578_at	RCC2	0.0027445	2.32221
224584_at	C20orf30	0.0032913	-2.16874
224590_at	XIST	0.0259796	-2.63504

224598_at	MGAT4B	0.0025146	2.6614
224600_at	CGGBP1	0.0036744	-2.1582
224604_at	C4orf3	0.0000811	-2.40956
224607_s_at	SRP68	0.0011665	-2.57609
224609_at	SLC44A2	0.00077	4.39777
224612_s_at	DNAJC5	0.0006051	1.75958
224619_at	CASC4	0.0002386	-2.41328
224621_at	MAPK1	0.0031866	-4.93409
224628_at	ERLEC1	0.0061502	-1.94212
224629_at	LMAN1	0.0004809	-3.98664
224636_at	ZFP91	0.0000573	-3.05818
224637_at	OST4	0.0006003	2.2213
224641_at	FYTTD1	0.0272519	-2.42156
224643_at	PRRC1	0.0003736	-7.09152
224652_at	CCNY	0.0014989	2.23187
224653_at	EIF4EBP2	0.002821	-2.24959
224655_at	AK3	0.0010325	-3.5396
224657_at	ERRFI1	0.0445338	-2.55846
224660_at	PIGY	0.0001307	-4.75947
224662_at	KIF5B	0.0003756	-2.25549
224664_at	ANAPC16	0.0003042	-2.67169
224671_at	MRPL10	0.0027289	-2.05092
224688_at	C7orf42	0.0004873	-1.77776
224693_at	C20orf108	0.0059643	-2.64616
224694_at	ANTXR1	0.0076247	-2.89823
224700_at	STT3B	0.0019899	-3.60787
224702_at	TMEM167A	0.0008052	-2.51067
224707_at	C5orf32	0	-5.25933
224709_s_at	CDC42SE2	0.0029225	-2.94209
224710_at	RAB34	0.0116656	-1.95217
224713_at	MKI67IP	0.0141204	-2.17981
224715_at	WDR34	0.0685649	1.66193
224716_at	SLC35B2	0.0044253	1.81561
224717_s_at	C19orf42	0.0140448	-2.75082
224724_at	SULF2	0.0079492	-2.1322
224726_at	MIB1	0.0002401	-6.12427
224728_at	ATPAF1	0.001048	-2.12808
224736_at	CCAR1	0.0182255	-3.00598
224740_at	C5orf43	0.0032395	-4.08062
224747_at	UBE2Q2	0.0060741	-2.3965

224749_at	ITFG3	0.0564602	1.46133
224752_at	PL-5283	0.0005313	3.42099
224753_at	CDCA5	0.0000109	3.70491
224755_at	TM9SF3	0.0000002	-6.2773
224759_s_at	C12orf23	0.019916	-2.07503
224760_at	SP1	0.0438381	1.57978
224764_at	ARHGAP21	0.0001159	-2.43548
224767_at	RPL37	0.0020299	-1.70124
224777_s_at	PAFAH1B2	0.0006938	-3.70077
224780_at	RBM17	0.0006898	-2.76064
224786_at	SCOC	0.0019657	-4.99583
224791_at	ASAP1	0.0120438	1.98607
224797_at	ARRDC3	0.0061236	-3.05428
224800_at	WDFY1	0.0072713	-2.8391
224801_at	NDFIP2	0	-8.59942
224807_at	GRAMD1A	0.0058024	2.21956
224812_at	HIBADH	0.0000937	-3.25491
224813_at	WASL	0.0000774	-3.64972
224815_at	COMMD7	0.0020738	1.62509
224819_at	TCEAL8	0.0087441	-1.95248
224824_at	FAM36A	0.0188644	1.42672
224828_at	CPEB4	0.0163755	-2.44101
224830_at	NUDT21	0.0001225	-5.28871
224836_at	TP53INP2	0.0148729	1.31222
224839_s_at	GPT2	0.0030805	2.0215
224840_at	FKBP5	0.0000849	-6.61179
224843_at	SLAIN2	0.002014	-1.81004
224851_at	CDK6	0.05426	-2.47473
224852_at	TTC17	0.0025884	-2.04619
224860_at	C9orf123	0.0010524	-2.33731
224861_at	GNAQ	0.0117814	-2.5763
224864_at	SRA1	0.0322153	1.99788
224865_at	FAR1	0.0077387	-2.32213
224869_s_at	MRPS25	0.0051709	1.66051
224870_at	KIAA0114	0.0255028	1.9195
224871_at	TPRG1L	0.0000099	-4.85848
224874_at	POLR1D	0.0014445	-1.75271
224889_at	FOXO3	0.003898	-2.03791
224890_s_at	C7orf59	0.0111224	2.05813
224892_at	PLDN	0.0000058	-2.09179

224893_at	ATL3	0.0000017	-7.5816
224895_at	YAP1	0.0981386	1.61093
224896_s_at	TTL	0.0006064	-2.22191
224899_s_at	MAGT1	0.0210533	-2.37055
224900_at	ANKFY1	0.0002398	-2.49558
224901_at	SCD5	0.0000019	-17.2067
224904_at	PDPR	0.0064903	-1.86668
224905_at	WDR26	0.0427175	1.2868
224906_at	ANO6	0.0000018	-4.41803
224911_s_at	DCBLD2	0.000043	-4.10536
224913_s_at	TIMM50	0.0335939	1.4829
224914_s_at	SARNP	0.001377	-3.58062
224917_at	MIR21	0.0036073	3.44129
224921_at	SCAMP2	0.0056299	1.42667
224928_at	SETD7	0.0009964	-3.93653
224929_at	TMEM173	0.0139573	1.63285
224931_at	SLC41A3	0.002089	-1.72708
224932_at	CHCHD10	0.0001044	3.33628
224933_s_at	JMJD1C	0.016659	-2.99392
224937_at	PTGFRN	0.0000029	-2.705
224938_at	NUFIP2	0.0028382	-2.13229
224945_at	BTBD7	0.0031812	-1.97082
224949_at	YIPF5	0.0012306	-2.80046
224954_at	SHMT1	0.0002731	1.91558
224957_at	C18orf32	0.0154348	-2.09918
224964_s_at	GNG2	0.0001146	-4.70354
224967_at	UGCG	0.0137348	-3.81114
224968_at	CCDC104	0.0001784	-7.02086
224969_at	ATXN7L3	0.0323073	1.24546
224972_at	ROMO1	0.0025277	2.90862
224973_at	FAM46A	0.0008355	-2.64626
224977_at	C6orf89	0.0164137	-1.8763
224985_at	NRAS	0.0520517	1.49382
224994_at	CAMK2D	0.0006406	-1.9377
224995_at	SPIRE1	0.0130608	-2.82096
225001_at	RAB3D	0.021177	1.44638
225002_s_at	SUMF2	0.053776	1.27877
225009_at	CMTM4	0.0081074	-3.3881
225014_at	C4orf52	0.0003841	-4.87633
225017_at	CCDC14	0.0047181	1.82698

225021_at	ZNF532	0.0011299	-2.33847
225022_at	GOPC	0.0055096	-1.8908
225037_at	SLC35C2	0.0016361	2.40618
225039_at	RPE	0.0886806	1.39944
225042_s_at	CSRNP2	0.0008029	1.93225
225043_at	SLC15A4	0.0190391	-1.93295
225049_at	BLOC1S2	0.0463368	-2.38878
225052_at	TMEM203	0.0040974	-2.72512
225056_at	SIPA1L2	0.0051081	-2.76556
225060_at	LRP11	0.0000652	-7.27986
225064_at	RABEP1	0.0007293	-1.82227
225075_at	PDRG1	0.065954	1.21241
225081_s_at	CDCA7L	0.000115	-2.62268
225082_at	CPSF3	0.049624	1.38786
225086_at	FAM98B	0.0030772	-2.95857
225093_at	UTRN	0.0007493	-1.98305
225096_at	C17orf79	0.0019977	-1.78702
225101_s_at	SNX14	0.0138745	-2.1986
225102_at	MGLL	0.0565953	2.09718
225110_at	OGFOD1	0.0015037	-2.09686
225112_at	ABI2	0.0020208	-1.99506
225113_at	AGPS	0.00255	-2.22427
225121_at	TBC1D23	0.000125	-2.84018
225126_at	MRRF	0.0029496	1.38723
225127_at	TMEM181	0.0100985	-1.93102
225132_at	FBXL3	0.0000121	-7.73969
225133_at	KLF3	0.0001112	-4.79315
225139_at	NFATC3	0.0005429	-2.1857
225142_at	JHDM1D	0.0293377	2.59827
225149_at	PCID2	0.0170088	-2.56725
225159_s_at	ELK4	0.0024166	-2.10247
225162_at	SH3D19	0.0016457	-2.19782
225166_at	ARHGAP18	0	-31.4015
225168_at	FRMD4A	0.0069093	1.5613
225183_at	C16orf72	0.0065864	-1.83055
225187_at	KIAA1967	0.007944	1.65817
225189_s_at	RAPH1	0.0179609	-2.24536
225191_at	CIRBP	0.0101097	1.27839
225196_s_at	MRPS26	0.0001042	2.08521
225198_at	VAPA	0.0001193	-4.06769

225200_at	DPH3	0.0003232	-4.41854
225205_at	KIF3B	0.0013138	-2.31428
225207_at	PDK4	0.0000044	-6.89083
225218_at	ZFYVE27	0.0054707	1.56166
225220_at	SNHG8	0.0017914	-2.01063
225222_at	HIAT1	0.0021698	-4.69365
225223_at	SMAD5	0.0000484	-2.81634
225230_at	DRAM2	0.000278	-3.80947
225232_at	MTMR12	0.006592	-1.93649
225235_at	TSPAN17	0.0059142	1.38384
225236_at	RBM18	0.0010463	-2.08262
225238_at	MSI2	0.090755	1.15653
225258_at	FBLIM1	0.0027765	1.6843
225259_at	RAB6B	0.0115201	1.76646
225260_s_at	MRPL32	0.00005	-3.06684
225263_at	HS6ST1	0.0000889	2.0943
225264_at	RARS2	0.0089851	-3.01593
225269_s_at	RBMS1	0.0039835	-1.75714
225272_at	SAT2	0.0001918	-3.8987
225274_at	PCYOX1	0.0036068	-1.89413
225276_at	GSPT1	0.0000588	-4.63561
225278_at	PRKAB2	0.0011986	-2.47742
225283_at	ARRDC4	0.0000005	-13.8417
225284_at	DNAJC3	0.0115538	-2.14673
225295_at	SLC39A10	0.0040508	-4.18777
225297_at	HAUS1	0.000073	-4.54223
225298_at	PNKD	0.0160952	1.94796
225299_at	MYO5B	0.0000029	-5.19412
225300_at	C15orf23	0.004903	2.11837
225302_at	TMX3	0.0000042	-2.68143
225306_s_at	SLC25A29	0.0186744	1.72036
225307_at	ZNF511	0.0162316	1.67504
225312_at	COMMD6	0.0000467	-1.93427
225319_s_at	FAM104A	0.0006733	-1.71548
225321_s_at	PILRB	0.000047	4.20602
225325_at	MFSD6	0.0044994	-3.44076
225326_at	RBM27	0.0039859	-2.2527
225327_at	KIAA1370	0.0000184	-6.99573
225330_at	IGF1R	0.0064832	-1.94933
225334_at	C10orf32	0.0000007	-7.56454

225338_at	ZYG11B	0.0003572	-4.29262
225343_at	TMED8	0.0003448	-2.18048
225344_at	NCOA7	0.0054861	-2.95642
225346_at	MTERFD3	0.0000087	-2.15314
225348_at	SFRS13A	0.0130713	-2.35508
225352_at	SEC62	0.0014553	-2.04559
225353_s_at	C1QC	0.0069857	1.93753
225355_at	NEURL1B	0.0002532	3.33584
225363_at	PTEN	0.0028699	-2.01467
225370_at	PYGO2	0.0067541	1.76168
225373_at	C10orf54	0.0007976	-1.7328
225378_at	VPS37A	0.0054118	-2.52235
225383_at	ZNF275	0.048407	-2.50755
225384_at	DOCK7	0.003696	-4.57862
225386_s_at	HNRPLL	0.0336189	-2.04369
225387_at	TSPAN5	0.0000001	-7.31322
225389_at	BTBD6	0.0003508	-2.68759
225395_s_at	FAM120AOS	0.0007806	-2.4799
225401_at	C1orf85	0.0229017	1.39747
225406_at	TWSG1	0.0010937	-2.71798
225407_at	MBP	0.0000258	-2.69014
225410_at	C2orf64	0.0081301	1.6629
225411_at	TMEM87B	0.0042291	-2.73741
225415_at	DTX3L	0.0619934	1.44708
225417_at	EPC1	0.0029483	-1.74169
225422_at	CDC26	0.0002883	-4.35956
225424_at	GPAM	0.0022248	-2.89172
225425_s_at	MRPL41	0.0193012	1.7589
225432_s_at	CSRP2BP	0.0007816	-2.00837
225436_at	FAM108C1	0.0917003	1.61533
225439_at	NUDCD1	0.0073193	-2.85012
225448_at	NAPG	0.0037052	-2.72616
225455_at	TADA1	0.0147391	-1.97859
225462_at	TMEM128	0.0007859	-2.24219
225464_at	FRMD6	0.0001754	-3.51681
225468_at	PATL1	0.0119993	1.65143
225471_s_at	AKT2	0.0864118	1.35574
225474_at	MAGI1	0.0061602	-1.74875
225482_at	KIF1A	0.0005972	1.9708
225485_at	TSGA14	0.0063225	1.82914

225492_at	TMEM33	0.0282268	-2.44739
225497_at	ATE1	0.0081422	-2.3407
225505_s_at	FAM113A	0.0004569	2.14978
225506_at	KIAA1468	0.0044038	-1.87328
225509_at	SAP30L	0.0000008	-3.59635
225512_at	ZBTB38	0.0009759	-2.04378
225514_at	C14orf21	0.0465851	1.84875
225526_at	MKLN1	0.0045482	-1.81927
225527_at	CEBPG	0.0714269	1.456
225528_at	IPO8	0.0001702	-2.45758
225532_at	CABLES1	0.0018223	1.91565
225534_at	C8orf40	0.0014823	-6.22086
225538_at	ZCCHC9	0.0029561	-3.90057
225540_at	MAP2	0.0017178	-3.20603
225541_at	RPL22L1	0.0319482	1.76645
225545_at	EEF2K	0.0393303	1.61245
225551_at	CNST	0.0148558	-2.67464
225554_s_at	ANAPC7	0.0005699	1.72615
225556_at	VMA21	0.0009766	-2.83124
225557_at	CSRNP1	0.0002937	2.15107
225558_at	GIT2	0.0001182	-1.84602
225559_at	C3orf19	0.0000761	-1.75364
225560_at	POMT2	0.0001951	1.565
225568_at	TMEM141	0.0068539	1.70503
225572_at	CREB1	0.0015629	-1.98013
225576_at	C6orf72	0.0011848	-1.98761
225579_at	PQLC3	0.0000336	-7.08473
225580_at	MRPL50	0.0467157	-2.35836
225585_at	RAP2A	0.0106825	-1.83461
225591_at	FBXO25	0.0004443	-2.77449
225599_s_at	C8orf83	0.0147539	-2.35803
225602_at	GLIPR2	0.0026227	2.11551
225607_at	CCDC43	0.0024265	-1.7712
225611_at	MAST4	0.0170369	-2.26653
225616_at	SPRYD4	0.0681588	1.46075
225618_at	ARHGAP27	0.0010174	2.13873
225619_at	SLAIN1	0.035997	-2.15177
225621_at	ALG2	0.0006293	-3.09252
225625_at	ALKBH2	0.037961	1.47441
225626_at	PAG1	0.006914	-4.21733

225627_s_at	CACHD1	0.0082302	-2.90282
225629_s_at	ZBTB4	0.0057762	-1.75171
225631_at	EEPD1	0.0805024	1.439
225632_s_at	RAB43	0.0513887	1.32861
225633_at	DPY19L3	0.0034461	-3.13911
225639_at	SKAP2	0.0328784	-2.15159
225643_at	MAPK1IP1L	0.0133	-2.31863
225645_at	EHF	0.00002	36.1434
225649_s_at	STK35	0.0018976	1.86447
225655_at	UHRF1	0.000007	5.97261
225658_at	SPOPL	0.0000936	-11.5791
225660_at	SEMA6A	0.0020927	-1.75819
225661_at	IFNAR1	0.0013886	-1.89942
225665_at	ZAK	0.0085724	-2.25817
225672_at	GOLGA2	0.0032349	1.29929
225675_at	C14orf101	0.0018104	-2.12042
225677_at	BCAP29	0.0017676	-1.96919
225679_at	NAA30	0.0058469	-1.96746
225681_at	CTHRC1	0.0004672	17.0697
225684_at	SKA2	0.0076319	1.79828
225687_at	FAM83D	0.0003526	7.35345
225692_at	CAMTA1	0.0642292	1.59292
225698_at	NCRNA00219	0.0007306	-3.41568
225699_at	C7orf40	0.05361	1.46225
225703_at	FBRSL1	0.0027552	3.26262
225707_at	ARL6IP6	0.0033862	-3.35906
225710_at	GNB4	0.0000008	-10.9766
225713_at	STK11IP	0.0082655	1.52238
225715_at	RPTOR	0.016584	1.49997
225721_at	SYNPO2	0.0003774	-2.33921
225726_s_at	PLEKHH1	0.000392	7.00576
225728_at	SORBS2	0.0000009	-12.7964
225733_at	B3GALT6	0.0581552	1.42619
225735_at	ANKRD50	0.0025252	-2.76673
225741_at	THUMPD3	0.0208325	-2.20427
225760_at	MYSM1	0.0034198	-2.69451
225764_at	ETV6	0.0023822	1.81096
225765_at	TNPO1	0.0000049	2.64133
225768_at	NR1D2	0.0109211	-2.42445
225769_at	COG6	0.0051076	-3.38543

225770_at	RSPRY1	0.0003862	-2.57726
225771_at	AP1G1	0.0016999	-1.79287
225778_at	RBMS2	0.0191482	1.34419
225780_at	RSC1A1	0.0130507	-2.20069
225781_at	MAPK9	0.0011323	-2.01723
225782_at	MSRB3	0.0000313	-12.0549
225789_at	AGAP3	0.0232909	1.18596
225793_at	LIX1L	0.0000001	-6.01157
225794_s_at	C22orf32	0.0080772	-2.12602
225796_at	PXK	0.0021002	-2.02184
225798_at	JAZF1	0.000085	-2.69894
225802_at	TOP1MT	0.0105294	1.69477
225806_at	JUB	0.0219764	-2.25289
225809_at	PARM1	0.0003817	-4.18223
225812_at	C6orf225	0.0035658	-1.74613
225817_at	CGNL1	0	-9.28544
225820_at	PHF17	0.0035399	-2.23739
225822_at	TMEM125	0.000001	3.37224
225823_at	C19orf70	0.0087844	1.75872
225834_at	FAM72A///FAM	0.0091003	3.11687
225835_at	SLC12A2	0.0013724	-2.60291
225840_at	TEF	0.0004076	-2.13772
225842_at	PHLDA1	0.0231257	2.21002
225846_at	ESRP1	0.0000144	7.00998
225850_at	SFT2D1	0.0490521	-2.21793
225852_at	ANKRD17	0.0207357	-1.90436
225853_at	GNPNAT1	0.0134524	1.51182
225859_at	XIAP	0.0107166	-1.91439
225862_at	SLC25A26	0.0023928	-1.99222
225866_at	RPF2	0.0202102	-2.50909
225875_s_at	NIPAL3	0.0002487	-2.06358
225881_at	SLC35B4	0.0003311	-2.15778
225884_s_at	GZF1	0.0070009	-2.73606
225885_at	EEA1	0.0519398	-2.318
225887_at	C13orf23	0.0062597	-2.01558
225890_at	C20orf72	0.000619	2.07684
225892_at	IREB2	0.0006493	-3.21336
225910_at	HELZ	0.001211	-2.21514
225911_at	NPNT	0.0001126	-4.80334
225912_at	TP53INP1	0.0001803	-2.62638

225913_at	SGK269	0.0000014	-4.31247
225914_s_at	CAB39L	0.0049052	-1.73256
225919_s_at	C9orf72	0.01659	-2.10053
225921_at	NIN	0.0000001	-4.19913
225922_at	FNIP2	0.0141589	-2.44834
225927_at	MAP3K1	0.0013097	-1.91491
225941_at	EIF4E3	0.0005931	-3.17609
225945_at	ZNF655	0.0001669	-3.24913
225946_at	RASSF8	0.0000007	-7.38223
225947_at	MYO19	0.0000505	1.77387
225949_at	NRBP2	0.0019724	3.4185
225955_at	METRNL	0.0212564	1.4838
225956_at	C5orf41	0.0004952	-3.56447
225963_at	KLHDC5	0.0814346	1.31098
225968_at	PRICKLE2	0.0033979	-1.87442
225971_at	DDHD1	0.0004347	-2.62496
225974_at	TMEM64	0.0000533	-2.99674
225975_at	PCDH18	0.0009628	-2.08978
225976_at	BTF3L4	0.0004592	-2.5964
225982_at	UBTF	0.0290949	1.36723
225989_at	HERC4	0.0085106	-2.15964
225992_at	MLLT10	0.0011427	-3.10592
226007_at	ISCA2	0.0045812	-2.19361
226017_at	CMTM7	0.0001134	3.10023
226019_at	OMA1	0.0004115	-3.81022
226020_s_at	DAB1///OMA1	0.0000591	-7.4566
226022_at	SASH1	0.0000454	-6.44007
226030_at	ACADSB	0.0001122	-2.88452
226031_at	CCDC132	0.0026945	-2.57537
226032_at	CASP2	0.0169419	1.93194
226041_at	NAPEPLD	0.0000757	-4.3486
226043_at	GPSM1	0.0034796	2.13047
226045_at	FRS2	0.0266104	-2.13216
226050_at	TMCO3	0.0003421	-4.94749
226052_at	BRD4	0.0089207	1.93757
226066_at	MITF	0.0061429	-1.99173
226068_at	SYK	0.0004925	3.1364
226077_at	RNF145	0.0225107	-2.47747
226079_at	FLYWCH2	0.0436663	1.85269
226086_at	SYT13	0.0000076	2.457

226091_s_at	MRFAP1	0.0015277	-1.84771
226092_at	MPP5	0.0002666	-2.59673
226098_at	IFT80	0.0356049	-2.11625
226106_at	RNF141	0.0108185	-2.14105
226110_at	PTAR1	0.0005989	-3.35786
226112_at	SGCB	0.0007201	-2.41843
226115_at	AHCTF1	0.0274224	-2.03049
226116_at	DFFA	0.0044464	-2.78177
226117_at	TIFA	0.0004086	-4.58571
226118_at	CENPO	0.0005177	2.25415
226120_at	TTC8	0.0000407	-3.99415
226121_at	DHRS13	0.0000539	1.52066
226122_at	PLEKHG1	0.0003106	-2.15523
226126_at	TBCK	0.004108	-1.93439
226127_at	ALKBH3	0.0008442	-2.28045
226129_at	FAM83H	0.0002543	2.38049
226135_at	UHRF1BP1	0.0010265	-3.13457
226137_at	ZFHX3	0.0031233	2.03216
226140_s_at	OTUD1	0.0002543	-3.59372
226143_at	RAI1	0.0636158	1.29104
226144_at	REXO1	0.0192721	1.42746
226145_s_at	FRAS1	0.0000033	-9.34048
226147_s_at	PIGR	0.0393745	3.45608
226148_at	ZBTB44	0.0090958	-1.95463
226149_at	SNHG11	0.0003405	2.00509
226155_at	FAM160B1	0.0000004	-3.15021
226160_at	H6PD	0.016218	-1.86154
226178_at	SOCS4	0.0009186	-3.00827
226180_at	WDR36	0.0003329	-4.83219
226181_at	TUBE1	0.0016771	-2.94834
226185_at	CDS1	0.0019838	-3.38103
226186_at	TMOD2	0.0000003	-5.78023
226213_at	ERBB3	0.09618	1.63991
226218_at	IL7R	0.0644251	2.52815
226221_at	KIAA1432	0.0117367	-1.99274
226225_at	MCC	0.000051	-6.98831
226239_at	TMEM150A	0.006586	1.67071
226241_s_at	MRPL52	0.0000194	3.1636
226242_at	C1orf131	0.0008674	-3.1369
226245_at	KCTD1	0.0000095	3.64674

226247_at	PLEKHA1	0.0033782	-2.20836
226249_at	SNX30	0.0006324	-2.45809
226259_at	EXOC6	0.0015161	-2.12691
226261_at	ZNRF2	0.0069268	-1.97932
226265_at	QSER1	0.0022689	-3.22462
226269_at	GDAP1	0.0000142	-4.53503
226290_at	BDP1	0.0040372	-2.46331
226298_at	RUNDC1	0.0021058	-1.92919
226303_at	PGM5	0.0007351	-2.23248
226309_at	DNAL1	0.0036691	-2.33684
226314_at	CHST14	0.00513	1.40934
226317_at	PPP4R2	0.0000727	-3.13158
226321_at	LYSMD3	0.0000119	-4.44933
226322_at	TMTC1	0.0000091	12.6295
226323_at	ZNF830	0.0062259	-3.38932
226324_s_at	IFT172	0.0470878	1.3904
226325_at	ADSSL1	0.0002727	2.05399
226329_s_at	MITD1	0.0110378	-3.34026
226330_s_at	FAM48A	0.008377	-1.82147
226335_at	RPS6KA3	0.0108009	-2.17149
226338_at	TMEM55A	0.0000821	-5.74618
226344_at	ZMAT1	0.0420714	-3.45797
226346_at	MEX3A	0.0000168	1.89732
226352_at	JMY	0.0005205	-2.41002
226353_at	SPPL2A	0.0012886	-3.34123
226358_at	APH1B	0.0001632	-2.61213
226360_at	ZNRF3	0.0070198	-1.89395
226361_at	TMEM42	0.0017636	-1.875
226363_at	ABCC5	0.008903	2.07145
226366_at	SHPRH	0.0000927	-3.95263
226376_at	UNK	0.0113281	1.27342
226380_at	PTPN21	0.0000056	-3.9
226381_at	PS1TP4	0.0248509	-3.06144
226382_at	CAMK1D///LOC	0.0000246	-3.3154
226383_at	C11orf46	0.000014	-5.02764
226386_at	C7orf30	0.0087622	-2.13787
226388_at	TCEA3	0.0000394	-5.77193
226390_at	STARD4	0.0079993	-1.77397
226393_at	CYP2U1	0.0004174	-1.94687
226398_s_at	C10orf4	0.0015506	-2.14418

226400_at	CDC42	0.0057504	-2.43683
226403_at	TMC4	0.0086412	2.46953
226408_at	TEAD2	0.0016434	1.53019
226412_at	SFRS18	0.0005614	-2.38
226420_at	MECOM	0.0000001	18.5595
226421_at	AMMECR1	0.0079184	-2.73065
226425_at	CLIP4	0	-16.5281
226430_at	RELL1	0.0000353	-4.4294
226434_at	C7orf47	0.0003662	1.68428
226439_s_at	NBEA	0.0000011	-3.12257
226441_at	MAP3K2	0.0000317	-2.66502
226445_s_at	TRIM41	0.008883	1.52351
226448_at	FAM89A	0.0000006	-10.9691
226449_at	CEP120	0.0000591	-2.65483
226455_at	CREB3L4	0.0027711	1.92535
226456_at	C16orf75	0.0006566	2.86275
226461_at	HOXB9	0.0084016	1.39087
226463_at	ATP6V1C1	0.0005778	-4.33881
226466_s_at	FAM58A	0.0538201	1.4964
226468_at	RNF115	0.003074	-1.72377
226473_at	CBX2	0.0000041	2.54835
226479_at	KBTBD6	0.0026576	-2.5475
226484_at	ZBTB47	0.0027833	-2.08474
226488_at	RCCD1	0.0002826	1.93251
226490_at	NHSL1	0.0002059	-4.84878
226492_at	SEMA6D	0.0000011	-8.44769
226497_s_at	FLT1	0.000143	2.7446
226501_at	XPNPEP3	0.0162639	-2.54894
226502_at	ELMOD2	0.000079	-2.58323
226506_at	THSD4	0.0098072	3.20557
226510_at	HEATR5A	0.0000605	-4.74234
226512_at	ZMYM2	0.0005281	-1.95853
226519_s_at	AGXT2L2	0.041555	1.47074
226521_s_at	FAM175A	0.0000341	-4.69669
226524_at	C3orf38	0.0150578	-1.9097
226528_at	MTX3	0.0027545	-2.24014
226533_at	HINT3	0.000153	-1.57953
226534_at	KITLG	0	-26.7485
226538_at	MAN2A1	0.0032265	-2.24249
226541_at	FBXO30	0.0001715	-3.18396

226552_at	IER5L	0.0081294	1.98158
226561_at	AGFG1	0.000102	-7.00755
226563_at	SMAD2	0.0073841	-1.99672
226566_at	TRIM11	0.000148	1.76453
226567_at	USP14	0.0023269	-1.74321
226569_s_at	CHTF18	0.0014277	2.48038
226571_s_at	PTPRS	0.0067022	2.0695
226580_at	BRMS1L	0.0146294	-2.10242
226581_at	ZFYVE20	0.0009634	-2.0945
226592_at	ZNF618	0.0128949	2.05245
226601_at	SLC30A7	0.0040062	-2.39862
226603_at	SAMD9L	0.0477091	-3.03273
226604_at	TMTC3	0.0024344	-3.10316
226609_at	DCBLD1	0.0001969	1.42693
226615_at	XPR1	0.0371419	2.30944
226622_at	MUC20	0.0003599	2.42002
226625_at	TGFBR3	0.0045338	-1.95657
226628_at	THOC2	0.0256737	-1.95299
226633_at	RAB8B	0.0000264	-5.38469
226634_at	METTL10	0.0167026	-1.99828
226636_at	PLD1	0.0002096	-2.53005
226638_at	ARHGAP23	0.0239294	-2.18106
226642_s_at	NUDCD2	0.005201	-4.2748
226648_at	HIF1AN	0.0014571	-1.96406
226649_at	PANK1	0.0000148	5.23856
226651_at	HOMER1	0.0111013	-1.86993
226660_at	RPS6KB1	0.0085813	-2.24039
226661_at	CDCA2	0.0005941	1.6717
226663_at	ANKRD10	0.0089166	-2.97205
226665_at	AHSA2	0.0554803	1.33703
226666_at	DAAM1	0.0000265	-3.24994
226668_at	WDSUB1	0.0269857	-2.01462
226670_s_at	PABPC1L	0.0000011	7.20353
226671_at	LAMP2	0.0000014	-9.07671
226676_at	ZNF521	0.0001804	-3.19423
226680_at	IKZF5	0.0000348	-4.28008
226685_at	SNTB2	0.0020086	-2.68127
226687_at	PRPF40A	0.0003127	-2.95732
226688_at	C3orf23	0.0000281	-3.22676
226689_at	CISD2	0.0005913	-2.82368

226692_at	SERF2	0.0051714	-1.74438
226693_at	SDHAP2	0.0458806	1.37356
226694_at	AKAP2///PALM2	0.0355437	1.51284
226695_at	PRRX1	0.000025	-8.72465
226701_at	GJA5	0.0081436	1.6441
226706_at	FLJ23867///QS	0.0212549	1.47687
226707_at	NAPRT1	0.0058006	1.71695
226710_at	C8orf82	0.007912	1.93312
226711_at	FOXN2	0.0006047	-1.65709
226726_at	MBOAT2	0.0452124	1.48709
226727_at	CISD3	0.0154156	1.51004
226728_at	SLC27A1	0.016052	1.52039
226732_at	RBM33	0.0101105	1.58785
226736_at	CHURC1	0.0242195	-4.70617
226741_at	SLC12A6	0.0064637	-2.06345
226742_at	SAR1B	0.0011809	-2.9311
226743_at	SLFN11	0.0006705	-2.39155
226744_at	METT10D	0.0052504	-2.10462
226747_at	TXNDC16	0.0173753	-1.88893
226748_at	LYSMD2	0.0000352	-8.26432
226749_at	MRPS9	0.0002774	-3.07647
226751_at	CNRIP1	0.0000001	-10.4371
226757_at	IFIT2	0.0193305	-3.29776
226760_at	MBTPS2	0.0091287	-3.31297
226765_at	SPTBN1	0.0002215	-2.37977
226766_at	ROBO2	0.0044263	-1.94111
226775_at	ENY2	0.0237534	-2.4285
226778_at	C8orf42	0.00057	-1.89923
226780_s_at	C7orf55	0.0584008	1.74207
226784_at	TWISTNB	0.0003672	-1.81497
226785_at	ATP11C	0.0000082	-3.79454
226789_at	EMB	0	-27.0142
226800_at	EFCAB7	0.0000195	-5.5449
226801_s_at	AIDA	0.0005682	-1.7753
226803_at	CHMP4C	0.0009239	5.94437
226806_s_at	NFIA	0.0011367	-1.66753
226807_at	ZFP1	0.0000346	-3.71595
226808_at	ZNF862	0.0000371	-2.73739
226810_at	OGFRL1	0.0000165	-6.03862
226811_at	FAM46C	0.0121555	-2.58729

226816_s_at	KIAA1143	0.0067223	-1.8429
226820_at	ZNF362	0.0055324	1.91042
226823_at	PHACTR4	0.0142723	-1.99173
226824_at	CPXM2	0.0095716	-1.8054
226829_at	AFAP1L2	0.0649056	2.00152
226833_at	CYB5D1	0.0056187	-1.87052
226839_at	NR2C2AP	0.0003532	1.9169
226845_s_at	MYEOV2	0.002571	1.54818
226851_at	LYPLAL1	0.0000016	-13.5006
226853_at	BMP2K	0.0968421	1.60493
226859_at	DNAJC25	0.0038196	-2.64198
226862_at	MBD1	0.058127	1.20193
226863_at	FAM110C	0.0001722	-6.75929
226864_at	PKIA	0.0025061	-1.87552
226886_at	GFPT1	0.0117992	-2.07887
226889_at	WDR35	0.0001068	-2.84456
226902_at	USP13	0.000008	-3.04295
226912_at	ZDHHC23	0.0046001	1.51392
226914_at	ARPC5L	0.0081935	2.65187
226917_s_at	ANAPC4	0.0016201	-2.12664
226921_at	UBR1	0.0000027	-2.44093
226922_at	RANBP2	0.005657	-1.79208
226930_at	FNDC1	0.0230601	1.90122
226932_at	SSPN	0.0064273	-2.69801
226935_s_at	CLPTM1L	0.0989323	1.21702
226943_at	C12orf73	0.0018127	2.40985
226944_at	HTRA3	0.0108755	2.1132
226961_at	PRR15	0.0017363	-3.54374
226962_at	ZBTB41	0.0084174	-2.56388
226965_at	FAM116A	0.0001659	-3.27959
226974_at	NEDD4L	0.0002498	-2.51855
226975_at	RNPC3	0.0673763	-2.68848
226976_at	KPNA6	0.0013964	-1.95095
226977_at	C5orf53	0.0000246	-7.3147
226980_at	DEPDC1B	0.0079605	2.17441
226981_at	MLL	0.0002723	-2.53337
226982_at	ELL2	0.0001402	-2.37251
226994_at	DNAJA2	0.0000033	-3.06063
227001_at	NIPAL2	0.001201	-2.95707
227003_at	RAB28	0.0017841	-1.8

227013_at	LATS2	0.0000626	-3.99369
227018_at	DPP8	0.0525251	1.23456
227020_at	YPEL2	0.0000123	-3.69305
227022_at	GNPDA2	0.0000074	-3.93635
227024_s_at	MRPL55	0.0111109	1.52027
227028_s_at	DGCR2	0.000881	1.61668
227029_at	FAM177A1	0.0019274	-2.56865
227031_at	SNX13	0.0000003	-13.7928
227038_at	SGMS2	0.000156	-8.66025
227040_at	NHLRC3	0.0092378	-1.89903
227053_at	PACSIN1	0.0000657	2.09307
227058_at	C13orf33	0.0000001	-12.43
227063_at	C17orf61	0.0044458	2.34252
227068_at	PGK1	0.0253445	2.15636
227070_at	GLT8D2	0.0000334	-6.69497
227075_at	ELP3	0.0175957	-2.81119
227077_at	ZNF286A///ZNF	0.0047797	-2.05605
227080_at	ZNF697	0.0000108	-2.8545
227083_at	B3GALTL	0.0003586	-2.26246
227085_at	H2AFV	0.0869439	1.24336
227088_at	PDE5A	0.0000004	-6.29053
227089_at	COG5	0.0005237	-1.72437
227090_at	PHF21A	0.0287392	1.30587
227094_at	DHTKD1	0.0006914	1.47963
227095_at	LEPROT	0.000199	-3.16187
227099_s_at	AG2	0.0000498	15.6976
227101_at	ZNF800	0.0441719	-2.56437
227116_at	MON1B	0.0001503	-3.36008
227120_at	FOXP4	0.0005451	1.76249
227132_at	ZNF706	0.0076417	-2.44911
227135_at	NAAA	0.0000064	-2.90793
227141_at	TYW3	0.0475979	-2.3528
227145_at	LOXL4	0.0071682	1.75414
227148_at	PLEKHH2	0.0000001	-11.6735
227153_at	IMMP2L	0.0123976	-2.03107
227157_at	CCDC111	0.0000925	-4.3585
227158_at	C14orf126	0.0104007	-1.85357
227162_at	ZBTB26	0.0116029	-3.07564
227163_at	GSTO2	0.0660909	1.79207
227165_at	SKA3	0.0020398	1.65087

227174_at	WDR72	0.0835065	3.54195
227176_at	SLC2A13	0.0003265	-2.88516
227180_at	ELOVL7	0.0000043	-4.01845
227184_at	PTAFR	0.0696466	1.702
227187_at	CBLL1	0.0077488	-1.97673
227188_at	C21orf63	0.0067132	-4.66051
227195_at	ZNF503	0.0008741	2.04381
227197_at	SGEF	0.0000469	-3.46221
227198_at	AFF3	0.0005441	-2.48475
227199_at	DIP2A	0.0053319	-2.32608
227203_at	FBXL17	0.0012489	-2.3361
227205_at	TAF1	0.0016172	1.59637
227209_at	CNTN1	0.0123292	-3.31392
227211_at	PHF19	0.0430884	1.55634
227221_at	ZMAT3	0.0022088	-2.31259
227224_at	RALGPS2	0.0000956	-2.97829
227235_at	GUCY1A3	0.0003462	5.2931
227239_at	FAM126A	0.0008446	-3.44268
227245_at	NAA25	0.0079468	-2.32302
227246_at	PLRG1	0.0002857	-3.70565
227251_at	DCAF5	0.0854599	1.13478
227267_at	POC5	0.0007673	-2.06104
227268_at	RNFT1	0.0000439	-5.47038
227274_at	SYNJ2BP	0.000919	-3.69455
227279_at	TCEAL3	0.0000005	-3.56698
227280_s_at	CCNYL1	0.0021753	-3.08798
227282_at	PCDH19	0.0000005	4.63034
227284_at	ZNF766	0.001626	-1.89117
227286_at	INO80E	0.0061438	1.4579
227288_at	SFRS12IP1	0.0005374	-3.83626
227291_s_at	BOLA3	0.0973529	1.33888
227292_at	C11orf84	0.0040593	1.3848
227297_at	ITGA9	0.0045833	3.65072
227314_at	ITGA2	0.000169	-3.84431
227322_s_at	BCCIP	0.0011246	-4.20408
227340_s_at	RGMB	0.0518249	1.21258
227342_s_at	MYEOV	0.0781965	1.40633
227353_at	TMC8	0.0559885	1.34175
227357_at	TAB3	0.0023158	-3.54803
227370_at	FAM171B	0.0000137	-7.97149

227375_at	ANKRD13C	0.0001754	-2.92435
227388_at	TUSC1	0.0007516	-2.47748
227394_at	NCAM1	0.0007319	4.7038
227409_at	PPP1R3E	0.0042505	1.44187
227425_at	REPS2	0.0001005	-2.83524
227428_at	GABPA	0.0005464	-3.38304
227435_at	KIAA2018	0.000318	-3.21198
227438_at	ALPK1	0.0101007	-1.99018
227444_at	ARMCX4	0.0000056	-4.41378
227446_s_at	C14orf167	0.012204	-1.87126
227447_at	SKIV2L2	0.0000122	-3.22755
227448_at	ARGLU1	0.0026537	-2.11821
227454_at	TAOK1	0.0258193	-2.39281
227458_at	CD274	0.0136453	-3.08601
227461_at	STON2	0	18.8026
227462_at	ERAP2	0.0135643	-3.25581
227466_at	FAM200B	0.0211414	-1.95508
227467_at	RDH10	0.000042	-1.81278
227470_at	ZNF48	0.000785	1.68001
227471_at	HACE1	0.002488	-3.26465
227475_at	FOXQ1	0.0000492	8.28622
227484_at	SRGAP1	0.0357128	-3.10533
227485_at	DDX26B	0.0000001	-3.70618
227486_at	NT5E	0.0001316	-2.72319
227498_at	SOX6	0.0015124	-4.13022
227506_at	SLC16A9	0.0278443	-2.60006
227517_s_at	GAS5	0.0001352	4.33069
227520_at	TXLNG	0.0014037	-3.51821
227521_at	FBXO33	0.0082977	-1.94033
227522_at	CMBL	0.0000089	-6.42203
227526_at	CDON	0.0000001	-3.21879
227528_s_at	MLL2	0.0002189	3.01596
227530_at	AKAP12	0.077442	-2.54823
227534_at	C9orf21	0.0000668	-3.16409
227536_at	ZC3H13	0.0000016	-6.91691
227539_at	GNA13	0.0002577	-2.73168
227540_at	EEFSEC	0.0056905	1.39964
227542_at	SOCS6	0.0001075	-3.21693
227545_at	BARD1	0.0164666	2.12786
227556_at	NME7	0.070205	1.61382

227560_at	SFXN2	0.0000349	1.76785
227561_at	DDR2	0.0000043	-3.64113
227563_at	FAM27E3	0.0352836	1.21853
227566_at	NTM	0.0700661	1.24346
227569_at	LNX2	0.0001046	-2.70387
227572_at	USP30	0.0003235	-2.61387
227577_at	EXOC8	0.003357	-2.57671
227579_at	FER	0.0000514	-3.00963
227580_s_at	TECPR1	0.0286175	1.69648
227582_at	KLHDC9	0.0037675	2.05524
227584_at	NAV1	0.0000004	2.01124
227585_at	ATAD1	0.0000582	-2.1818
227587_at	KRI1	0.0219595	1.41845
227593_at	FLJ37453	0.0208852	-2.02296
227595_at	ZMYM6	0.0086114	-2.31939
227599_at	C3orf59	0.0000824	-2.36219
227607_at	STAMBPL1	0	-5.04728
227608_at	YY1AP1	0.0072087	1.33763
227611_at	TARSL2	0.0022725	-1.75814
227614_at	HKDC1	0.0016381	2.12855
227621_at	WTAP	0.0250456	-2.40659
227624_at	TET2	0.0003554	-3.63919
227627_at	SGK3	0.0000173	-4.87186
227638_at	KIAA1632	0.008268	-2.20853
227639_at	PIGK	0.0001584	-3.73134
227646_at	EBF1	0.0692847	2.06201
227651_at	NACC1	0.0120468	1.98162
227655_at	SNORD123	0.0040726	-2.13787
227656_at	C6orf70	0.0005959	-1.7264
227657_at	RNF150	0.0108108	-2.50263
227668_at	C17orf56	0.0059576	1.40596
227685_at	TMF1	0.0104746	-2.81591
227688_at	LRCH2	0.0001156	-2.90617
227689_at	ZNF227	0.0008383	-1.96202
227693_at	WDR20	0.0091764	-1.99489
227697_at	SOCS3	0.0000254	2.70911
227699_at	C14orf149	0.001219	-1.73999
227701_at	C10orf118	0.0000531	-3.12142
227702_at	CYP4X1	0.0004047	-6.3797
227703_s_at	SYTL4	0.0087724	-2.46141

227705_at	TCEAL7	0.0000005	-19.4381
227708_at	EEF1A1	0.0000011	-17.6449
227713_at	KATNAL1	0.0000251	-2.58697
227719_at	SMAD9	0	-9.91878
227721_at	CPAMD8	0.0598993	1.45102
227725_at	ST6GALNAC1	0.0048113	7.285
227727_at	MRGPRF	0.0000001	-2.0493
227731_at	CNBP	0.0025342	-2.40405
227736_at	C10orf99	0.0173543	2.49171
227748_at	RBMXL1	0.0081325	-2.24745
227753_at	TMEM139	0.0000001	6.27791
227761_at	MYO5A	0.0000049	-4.22957
227766_at	LIG4	0.0363248	-2.43569
227767_at	CSNK1G3	0.001009	-2.47076
227771_at	LIFR	0.0000068	-2.86774
227775_at	CELF6	0.0899363	1.40155
227787_s_at	MED30	0.0125411	-2.50411
227798_at	SMAD1	0.0056396	1.81534
227814_at	WDR53	0.0646927	1.24182
227819_at	LGR6	0.0000055	5.44935
227824_at	PRKCB	0.0215304	1.43173
227828_s_at	FAM176A	0.0011834	1.54122
227829_at	GYLTL1B	0.0003153	1.41153
227830_at	GABRB3	0.0007909	-2.35075
227833_s_at	MBD6	0.0005655	2.67898
227836_at	UTP23	0.0010536	-3.45352
227840_at	C2orf76	0.0028069	-2.58975
227846_at	GPR176	0.0005924	-1.86828
227856_at	C4orf32	0.0000473	-5.76959
227861_at	TMEM161B	0.0026299	-3.08854
227862_at	TRNP1	0.0016871	-2.4031
227865_at	C9orf103	0.0000619	-2.88088
227870_at	IGDCC4	0.0870498	2.03191
227871_at	CHM	0.0211938	-2.83646
227873_at	TXNDC15	0.0000113	-2.26984
227875_at	KLHL13	0.0001013	-2.47783
227876_at	ARHGAP39	0.0005404	1.50018
227889_at	LPCAT2	0.027372	-2.2909
227892_at	PRKAA2	0.0001229	-2.41489
227894_at	WDR90	0.0001108	2.91513

227895_at	FAM120B	0.0855098	1.24097
227909_at	NCRNA00086//	0.0188241	-2.3122
227911_at	ARHGAP28	0	-10.5793
227917_at	FAM85A	0.0210503	-2.14834
227919_at	UCA1	0.0150901	2.01991
227920_at	BEND3	0.0117312	1.67203
227933_at	LINGO1	0.0008889	1.93483
227934_at	KPNA5	0.0000037	-4.40187
227935_s_at	PCGF5	0.0300317	1.47701
227944_at	PTPN3	0.0041667	-1.73863
227945_at	TBC1D1	0.0000124	-2.82364
227946_at	OSBPL7	0.0557438	1.52193
227960_s_at	FAHD1	0.0001801	-2.75648
227961_at	CTSB	0.0152591	-2.18879
227982_at	SEPSECS	0.0035507	-2.6267
227983_at	RILPL2	0.0019411	-1.98956
227993_at	METAP2	0.0037493	-2.05837
227998_at	S100A16	0.0097385	1.6399
228001_at	TMEM50B	0.0629661	1.28946
228005_at	ZXDB	0.0007715	-2.89008
228010_at	PPP2R2C	0.0219912	1.74925
228027_at	GPRASP2	0.0000015	-5.49822
228029_at	ZNF721	0.0073637	-1.91455
228033_at	E2F7	0.0000837	6.17025
228035_at	STK33	0.0017936	-1.78989
228039_at	DDX46	0.0056152	-2.35579
228040_at	MGC21881	0.0002209	-5.44528
228041_at	AASDH	0.0000012	-2.44689
228044_at	SERP2	0.0027701	-2.08245
228063_s_at	NAP1L5	0.0002357	-5.16058
228067_at	C2orf55	0.0055942	-2.00717
228069_at	FAM54A	0.0000062	4.80089
228070_at	PPP2R5E	0.0013747	-2.29662
228073_at	NANP	0.0016135	-2.35863
228077_at	MRI1	0.0221312	-1.95494
228087_at	CCDC126	0.0000159	-4.23976
228090_at	NMNAT3	0.0053871	-2.10085
228093_at	ZNF599	0.0090924	1.53748
228095_at	PHF14	0.0005304	-2.62152
228096_at	C1orf151	0.0004507	-1.62831

228100_at	C1orf88	0.0064105	-1.78776
228121_at	TGFB2	0.0001713	-2.39726
228122_at	CCDC66	0.0013714	-2.51873
228124_at	ABHD12	0.091557	1.45416
228131_at	ERCC1	0.0012279	-1.78247
228141_at	GPX8	0.0015395	-2.18345
228142_at	UQCR10	0.000608	-2.59117
228149_at	C7orf60	0.0000358	-4.36093
228153_at	RNF144B	0.0001025	4.51317
228155_at	C10orf58	0.0000772	-5.24482
228164_at	AP4E1	0.0012743	-2.2177
228168_at	ATP5G3	0.0046218	-1.85434
228174_at	SCAI	0.005348	-2.81889
228176_at	S1PR3	0.0554119	1.6743
228184_at	DISP1	0	-4.03538
228195_at	C2orf88	0.0905376	2.32866
228198_s_at	FAHD2A///LOC	0.0743613	1.37406
228204_at	PSMB4	0.0068292	-1.82883
228220_at	FCHO2	0.0002042	-5.66411
228221_at	SLC44A3	0.0099351	-1.86141
228234_at	TICAM2///TMEI	0.0041903	-2.34076
228242_at	N4BP2	0.0507382	-2.25227
228245_s_at	OVOS///OVOS2	0	8.11427
228248_at	RICTOR	0.0023329	-2.10095
228249_at	C11orf74	0.0001482	-2.66827
228259_s_at	EPB41L4A	0.0020935	-1.88615
228260_at	ELAVL2	0.0000559	-3.07916
228263_at	GRASP	0.0008517	1.44914
228282_at	MFSD8	0.0102073	-2.10659
228286_at	GEN1	0.0001177	2.23304
228298_at	FAM113B	0.0004157	-2.62792
228304_at	RBM43	0.0009022	-3.41061
228323_at	CASC5	0.0006296	2.84679
228325_at	KIAA0146	0.0010486	-1.66817
228330_at	ZUFSP	0.0283885	-1.98205
228336_at	PWWP2A	0.0001186	-1.81151
228345_at	CHIC1	0.0000044	-3.13093
228346_at	ZNF844	0.0129519	-2.1246
228347_at	SIX1	0.0196713	9.26961
228348_at	LINS1	0.0000071	-3.17971

228349_at	KIAA1958	0.0039928	-2.01519
228365_at	CPNE8	0.0000091	-4.21162
228367_at	ALPK2	0.0981477	1.31998
228368_at	ARHGAP20	0.0008507	-2.27067
228369_at	CNPY3	0.0446183	1.65927
228375_at	IGSF11	0.0014445	-2.67148
228377_at	KLHL14	0.000001	43.2151
228385_at	DDX59	0.003573	-1.89661
228391_at	CYP4V2	0.0019311	-4.12996
228393_s_at	ZNF302	0.0052929	-2.33328
228396_at	PRKG1	0.0000003	-4.64362
228400_at	SHROOM3	0.0000722	-4.54183
228404_at	IRX2	0.0825403	1.42299
228411_at	PARD3B	0.0000313	-3.03083
228415_at	AP1S2	0.0002903	4.49329
228416_at	ACVR2A	0.0109949	-2.05601
228423_at	MAP9	0.0103794	-2.72734
228434_at	BTNL9	0.003972	2.56226
228436_at	KCNC4	0.0028177	1.59318
228446_at	KIAA2026	0.0034109	-2.01925
228450_at	PLEKHA7	0.0000096	-2.4127
228454_at	LCOR	0.0043441	-2.64052
228458_at	C6orf226	0.0194506	1.63032
228461_at	SH3RF3	0.0986	1.22875
228468_at	MASTL	0.0920039	1.81178
228469_at	PPID	0.0000103	-2.86985
228479_at	SOAT1	0.0006859	-2.36188
228486_at	SLC44A1	0.0220228	-3.14664
228488_at	TBC1D16	0.069134	1.468
228499_at	PFKFB4	0.0000144	2.03911
228512_at	PTCD3	0.0044296	-1.90513
228513_at	TMEM219	0.0581809	1.14697
228520_s_at	APLP2	0.0192713	1.53138
228535_at	RAD1	0.0003464	-1.64299
228536_at	PRMT10	0.0006355	-1.92988
228544_s_at	CSRP2BP///PE	0.0113916	-2.3378
228546_at	DPP6	0.0010495	-3.46561
228551_at	DENND5B	0.001681	-2.55338
228556_at	YTHDC1	0.0225627	1.5592
228561_at	CDC37L1	0.000181	-2.03082

228568_at	GCOM1	0.0000002	-6.66572
228569_at	PAPOLA	0.0206081	-2.86083
228570_at	BTBD11	0.0034008	1.47568
228573_at	ANTXR2	0.0000974	-2.92323
228585_at	ENTPD1	0.0058845	1.44849
228593_at	MTMR9L	0.0698203	1.32015
228598_at	DPP10	0.0000006	-13.2999
228604_at	FAM76A	0.0009966	-2.23077
228613_at	RAB11FIP3	0.0012766	-3.84576
228621_at	HFE2	0.0057626	1.49554
228630_at	ZNF84	0.0064613	-1.76473
228633_s_at	CNTROB	0.0008494	2.16488
228636_at	BHLHE22	0.0945837	1.23829
228640_at	PCDH7	0.0008215	10.1836
228654_at	SPIN4	0.003432	2.21573
228665_at	CYYR1	0.0069256	4.93772
228698_at	SOX7	0.0382176	1.19429
228707_at	CLDN23	0.001449	-2.6314
228708_at	RAB27B	0	-66.4839
228711_at	ZNF37A	0.0058268	-2.22258
228715_at	ZCCHC12	0.0016526	-2.04606
228718_at	ZNF44	0.0110338	-1.81365
228724_at	TTLL7	0.0000768	-5.1695
228728_at	C7orf58	0.0000049	-4.90424
228729_at	CCNB1	0.0003314	5.72926
228739_at	CYS1	0.0000101	-5.53303
228744_at	HAUS2	0.0005375	-1.65919
228745_at	SGTB	0.0003879	-3.92162
228748_at	CD59	0.000615	-2.7362
228749_at	ZDBF2	0.0019916	-2.40259
228751_at	CLK4	0.0009516	-3.09141
228771_at	ADRBK2	0.0311649	-2.03007
228775_at	TMEM111	0.0005259	-2.18204
228777_at	KBTBD3	0.0005997	-2.09982
228778_at	MCPH1	0.002076	-1.80478
228790_at	FAM110B	0.0004004	-2.50979
228802_at	RBPMS2	0.0001398	-4.77981
228805_at	C5orf25	0.0104612	-2.143
228806_at	RORC	0.0006957	-2.99895
228813_at	HDAC4	0.0009808	-2.51031

228821_at	ST6GAL2	0.0000381	-4.17764
228834_at	TOB1	0.0004217	-2.97598
228844_at	SLC13A5	0.0606809	1.84252
228855_at	NUDT7	0.0001481	-2.98255
228859_at	C4orf21	0.0649122	1.26476
228877_at	RGL3	0.0010796	1.60794
228879_at	SNORD104	0.0570414	1.56065
228897_at	DERL3	0.0382111	1.74936
228904_at	HOXB3	0.014381	3.86553
228905_at	PCM1	0.0000475	-3.15762
228906_at	TET1	0.0272303	1.58354
228915_at	DACH1	0.0003305	8.33155
228916_at	CWF19L2	0.000326	-3.45726
228923_at	S100A6	0.0019409	1.62636
228927_at	ZNF397	0.0006717	-7.86993
228930_at	SCARNA15	0.0001924	1.95681
228931_at	COQ4	0.0927849	1.47546
228933_at	NHS	0.0031758	-2.57872
228937_at	C13orf31	0.0111016	-2.08982
228946_at	INTU	0.000131	-4.64924
228950_s_at	WLS	0.000248	-2.68076
228953_at	WHAMM	0.000503	-2.23244
228959_at	PDK3	0.0000416	-5.79991
228960_at	NARG2	0.0000253	-2.46481
228961_at	MIER3	0.0000566	-3.17658
228973_at	DLG2	0.0000005	-3.74156
228990_at	SNHG12	0.00811	1.74809
228997_at	TRNAU1AP	0.0037005	1.34212
228999_at	CHD2	0.0867293	1.56045
229002_at	FAM69B	0.0027668	3.27105
229004_at	ADAMTS15	0.0254089	1.85544
229014_at	FLJ42709	0.010287	-1.89448
229018_at	C12orf26	0.0000025	-5.74422
229019_at	ZNF385B	0.0000682	-3.72003
229022_at	ZFX	0.0003953	-3.01126
229030_at	CAPN8	0.0406653	1.54231
229033_s_at	MUM1	0.0003254	-1.8583
229043_at	PAPD5	0.0002672	-2.25836
229044_at	NUDT17	0.0001162	2.17876
229053_at	SYT17	0.0084427	2.43367

229054_at	C14orf181	0.0220101	1.29075
229064_s_at	RCAN3	0.0192736	1.26887
229067_at	SRGAP2P1	0.0209395	-2.62186
229084_at	CNTN4	0	-16.8082
229095_s_at	LIMS3///LIMS3-	0.0002376	9.57954
229099_at	C11orf83	0.0200773	1.94587
229105_at	GPR39	0.0000442	4.63046
229112_at	SIRT5	0.0244884	1.26423
229116_at	CNKSR2	0.0000183	-6.6712
229119_s_at	ZSWIM7	0.0071765	-2.3148
229134_at	VANGL1	0.0002222	1.86137
229135_at	FASTKD2	0.0049017	-1.9356
229139_at	JPH1	0.014699	2.15806
229146_at	C7orf31	0.000224	-1.92413
229160_at	MUM1L1	0	-48.2238
229172_at	HSPA12B	0.0091327	1.56156
229176_at	ANKH	0.000829	-1.93164
229210_at	RNASEH2B	0.0006381	-1.66785
229222_at	ACSS3	0.0011714	-3.62547
229227_at	FLJ45244	0.0262574	1.50888
229230_at	OSTalpha	0.0007867	-2.1513
229253_at	THEM4	0.0044859	-2.04007
229254_at	MFSD4	0	-30.86
229282_at	GATA6	0.0167176	1.45058
229285_at	RNASEL	0.0000164	-5.01873
229287_at	PCNX	0.0000013	-6.68774
229290_at	DAPL1	0.0281404	5.76596
229292_at	EPB41L5	0.0003301	4.33239
229302_at	TMEM178	0.0139282	2.72485
229312_s_at	GKAP1	0.001101	-3.70382
229313_at	ANO5	0.0000057	-2.82342
229325_at	ZZZ3	0.0125123	-1.82571
229331_at	SPATA18	0.0084285	-2.38088
229377_at	GRTP1	0.0318184	1.8996
229385_s_at	PLAC2	0.000125	-1.64223
229391_s_at	FAM26F	0.0002854	-3.03895
229393_at	L3MBTL3	0.0000098	-6.55771
229398_at	RAB18	0.0172246	-2.13526
229400_at	HOXD10	0.0018295	-5.04207
229419_at	FBXW7	0.0027575	-2.01848

229422_at	NRD1	0.0012889	-2.93619
229431_at	RFXAP	0.0031083	-2.95425
229432_at	NAGS	0.0432263	1.36764
229454_at	BCLAF1	0.0006952	-1.62952
229460_at	FAM126B	0.0094036	-2.92496
229464_at	MYEF2	0.0000368	3.54317
229466_at	TRIM66	0.0070239	1.31481
229468_at	CDK3	0.0064513	1.51421
229501_s_at	USP8	0.0001137	-1.59937
229511_at	SMARCE1	0.00117	-3.42799
229513_at	STRBP	0.0005945	-2.25564
229515_at	PAWR	0.0001063	-2.67502
229537_at	LMO4	0.0001476	-1.90019
229538_s_at	IQGAP3	0.0006584	4.38535
229539_at	ABCB8	0.012749	1.36372
229561_at	LRRC16B	0.0266923	2.11919
229562_at	RPL10A	0.0012551	1.75367
229574_at	TRA2A	0.0073986	-2.43778
229581_at	ELFN1	0.0000127	-2.07298
229584_at	LRRK2	0.012662	-2.12592
229588_at	DNAJC10	0.0042683	-2.44065
229590_at	RPL13	0.000171	-3.58596
229596_at	AMDHD1	0.0000076	-3.45241
229603_at	BBS12	0.0000064	-7.50635
229604_at	CMAH	0.0002883	-3.01031
229610_at	CKAP2L	0.0000351	2.09037
229618_at	SNX16	0.0004973	-3.32834
229622_at	FAM132B	0.0006736	2.69555
229623_at	TMEM150C	0.0000426	-8.70268
229632_s_at	INTS10	0.000141	-3.55209
229638_at	IRX3	0	-17.4993
229641_at	CCBE1	0.0105786	-1.87673
229647_at	NDUFS1	0.0954898	1.35065
229665_at	CSTF3	0.0005924	2.90427
229667_s_at	HOXB8	0.0092193	3.17763
229694_at	WDR11	0.0492343	-3.29912
229697_at	HIRIP3	0.0272396	1.50827
229700_at	ZNF738	0.002043	3.32761
229723_at	TAGAP	0.0357352	-2.47808
229731_at	FOXS1	0.0896069	1.26657

229742_at	C15orf61	0.0168974	-2.4219
229764_at	TPRG1	0.0003374	-2.75187
229765_at	ZNF207	0.010243	-3.8028
229784_at	MGC16121	0.0127451	2.12464
229785_at	KRIT1	0.0014164	-1.96619
229796_at	SIX4	0.0037053	2.89691
229800_at	DCLK1	0.0060921	-2.19473
229801_at	C10orf47	0.0230279	1.66113
229816_at	WDR78	0.0087271	-1.95295
229828_at	CDC73	0.000697	-2.78754
229829_at	C18orf18	0.0021723	-1.91091
229831_at	CNTN3	0.0034442	-3.99257
229842_at	ELF3	0.0001036	9.44388
229844_at	FOXP1	0.0000091	-2.0545
229848_at	ZNF10	0.0024257	-2.04475
229849_at	WIPF3	0.0005664	-3.12576
229885_at	RSF1	0.0094698	-2.02135
229886_at	C5orf34	0.0071905	2.01043
229897_at	ZNF641	0.0036614	-2.74791
229900_at	CD109	0.0473279	1.55954
229905_at	RAP1GDS1	0.0017873	-2.61889
229910_at	SHE	0.0000004	-3.92943
229912_at	SDK1	0.0018309	2.0056
229914_at	FLJ38717	0.0017986	1.78579
229927_at	LEMD1	0.0030101	3.45349
229940_at	SETD3	0.0807212	1.24189
229941_at	FAM166B	0.0220591	1.48634
229942_at	BNC2	0.0001524	-2.37727
229944_at	OPRK1	0.0065817	1.56114
229947_at	PI15	0.0025969	1.71067
229955_at	FBXO3	0.0001817	-2.52704
229963_at	BEX5	0.0147833	-3.18226
229973_at	C1orf173	0.0034035	-2.44865
229983_at	TIGD2	0.0010158	-2.69269
230015_at	PRCD	0.0000114	-2.86759
230018_at	DPP9	0.0008306	1.56167
230061_at	TM4SF18	0.0055167	1.45569
230067_at	FAM124A	0.0096594	1.67028
230069_at	SFXN1	0.0225764	-2.07754
230075_at	RAB39B	0.0001323	3.0113

230087_at	PRIMA1	0.0000047	6.09382
230110_at	MCOLN2	0.0000125	-6.59171
230113_at	MBNL3	0	-3.88708
230125_at	GUSB	0.0989213	1.70004
230141_at	ARID4A	0.0003412	-4.28577
230143_at	RNF165	0.000017	1.86286
230151_at	C13orf1	0.0003001	-1.7281
230158_at	DPY19L2	0.0028267	-2.74601
230169_at	THAP6	0.0188651	1.5016
230172_at	IFI27L1	0.0111457	1.93161
230177_at	GTF2H2B	0.0011665	-2.1559
230192_at	TRIM13	0.000188	-2.56721
230203_at	FLJ46875	0.0346709	1.49706
230204_at	HAPLN1	0.000566	2.84412
230205_at	ZNF561	0.0007709	-2.98468
230208_at	HCN4	0.0839565	1.44319
230220_at	UNC80	0.0022305	-2.42483
230237_at	ADCYAP1	0.0000254	-6.12186
230238_at	ANKRD43	0.0223849	2.35745
230249_at	KHDRBS3	0.0457663	1.27486
230250_at	PTPRB	0.0774014	1.75956
230251_at	C6orf176	0.0003466	-2.27977
230252_at	LPAR5	0	-5.22168
230253_at	SCUBE3	0.0348335	1.37583
230266_at	RAB7B	0.0001333	-2.6271
230270_at	PRPF38B	0.0158299	-3.81127
230283_at	NEURL2	0.088166	1.30516
230285_at	SVIP	0.0020647	-3.79578
230288_at	FGF14	0.0003229	-1.62661
230298_at	MBLAC2	0.0006222	-2.64359
230320_at	TBRG1	0.0003545	-2.24552
230355_at	SEPT7P2	0.0470164	1.26556
230360_at	GLDN	0.0115931	2.43366
230361_at	HEATR7A	0.000806	3.44752
230372_at	HAS2	0.0189021	1.24789
230381_at	C1orf186	0.0000009	10.4884
230391_at	CD84	0.0133875	-2.5004
230421_at	ZNF879	0.0000012	-3.25279
230425_at	EPHB1	0.0171276	-2.49767
230440_at	ZNF469	0.0003583	2.84647

230441_at	PLEKHG4B	0.0035928	2.277
230448_at	SLC38A10	0.0059858	1.60335
230452_at	FLJ42351///LO(	0.0303876	1.51454
230454_at	ICA1L	0.0002811	-3.21728
230469_at	RTKN2	0.0703454	1.39749
230518_at	MPZL2	0.0000037	18.3185
230550_at	MS4A6A	0.0026092	-2.08852
230551_at	KSR2	0.0001336	1.99725
230561_s_at	C2orf67	0.0029754	-2.48964
230563_at	RASGEF1A	0.0137375	2.74819
230569_at	KIAA1430	0.0025989	-2.22624
230618_s_at	BAT2L2	0.0075337	-3.1486
230620_at	USP27X	0.0006689	-2.22014
230621_at	IAH1	0.0094233	-1.80029
230624_at	SLC25A27	0.0002559	-3.19242
230626_at	TSPAN12	0.000573	6.87307
230644_at	LRFN5	0.0000437	-1.87008
230656_s_at	CIRH1A	0.0001364	-2.24085
230669_at	RASA2	0.0575527	-2.72751
230673_at	PKHD1L1	0.0000202	-10.4831
230699_at	PGLS	0.0256756	1.62518
230715_at	ZNF518B	0.000386	-1.97993
230717_at	LCN12	0.0009511	2.35116
230721_at	C16orf52	0.0000204	-2.45298
230744_at	FSTL1	0.0004473	-2.04478
230758_at	GEMIN8	0.0006604	-3.41664
230766_at	GART	0.0809355	1.46249
230777_s_at	PRDM15	0.0000924	2.00537
230779_at	TNRC6B	0.0245456	-3.00106
230788_at	GCNT2	0.0120583	2.75341
230792_at	FAAH2	0.091378	1.23795
230793_at	LRRC16A	0.0020004	-2.53511
230801_at	RPRD1B	0.0739875	1.35976
230807_at	CCDC151	0.045526	1.56395
230821_at	ZNF148	0.0048484	-1.91999
230828_at	GRAMD2	0.0013335	1.88835
230831_at	FRMD5	0.0372802	-2.42539
230847_at	WRNIP1	0.0113802	1.74656
230860_at	C3orf34	0.0009899	-2.3795
230867_at	COL6A6	0.000108	-3.68403

230869_at	FAM155A	0.0000036	-4.77346
230871_at	DHX30	0.0279255	-2.03881
230883_at	NXPH2	0.0000115	-11.0342
230887_at	CDC14B	0.0111418	1.21622
230904_at	FSD1L	0.0016641	-2.66382
230925_at	APBB1IP	0.0111516	-2.36695
230933_at	DSTN	0.0000142	-2.48931
230943_at	SOX17	0	121.253
230949_at	SLC23A3	0.0149069	1.3327
230954_at	C20orf112	0.0017446	2.91148
230974_at	DDX19B	0.0000527	-2.46473
230981_at	CATSPER3	0.0596687	1.16332
231008_at	UNC5CL	0.00936	1.84749
231022_at	OCLN	0.000266	-2.19111
231031_at	KGFLP2	0.0001906	-2.1288
231065_at	PDE6D	0.0354759	1.24309
231090_s_at	ARID2	0.011864	1.43353
231146_at	FAM24B	0.026546	1.46556
231164_at	ABCA17P	0.0033875	1.86925
231175_at	BEND6	0.0022615	-2.43012
231192_at	LPAR3	0.0066389	4.64272
231202_at	ALDH1L2	0.00207	2.18875
231213_at	PDE1A	0.0013464	-2.92316
231227_at	WNT5A	0.0032972	-2.3131
231270_at	CA13	0.0076341	-2.70077
231323_at	PSMB2	0.0148808	1.30991
231325_at	UNC5D	0.0000092	-5.31103
231341_at	SLC35D3	0.045623	1.20765
231361_at	NLGN1	0.0041522	-2.37277
231382_at	FGF18	0.0000003	11.6175
231391_at	CTXN3	0.0019019	-3.90627
231406_at	ORAI2	0.0248131	1.91763
231407_s_at	FOXH1	0.000514	1.65855
231442_at	ZPBP2	0.0816197	1.16182
231454_at	PLAC4	0.0010393	1.64345
231466_at	FAM71F1	0.048387	1.45741
231509_at	C7orf16	0.0018492	1.62702
231530_s_at	C11orf1	0.0027512	-2.57652
231531_at	C3orf24	0.0040087	1.5714
231572_at	PRSS37	0.0303563	1.3845

231577_s_at	GBP1	0.0821195	-2.92852
231622_at	ASB17	0.0443724	1.12085
231683_at	GLYAT	0.0187737	1.45773
231713_s_at	ELP2	0.0034727	-2.20817
231718_at	SLU7	0.0044114	-2.46141
231725_at	PCDHB2	0.0421148	2.79483
231795_at	STON1	0.0071469	1.2662
231804_at	RXFP1	0.0806538	1.38009
231806_s_at	STK36	0.0795629	1.64022
231807_at	KIAA1217	0	5.84093
231841_s_at	KIAA1462	0.0005782	-1.83825
231855_at	KIAA1524	0.058087	2.10916
231856_at	KIAA1244	0.0027754	-1.89411
231869_at	KIAA1586	0.0032145	-1.98014
231873_at	BMPR2	0.0149585	-2.20445
231892_at	C9orf100	0.0008269	1.5625
231895_at	SASS6	0.0096002	1.53499
231897_at	PTGR1	0.0030077	-4.80308
231899_at	ZC3H12C	0.0003112	-3.59558
231902_at	ZNF827	0.0130906	1.35629
231906_at	HOXD8	0	-42.9531
231917_at	GFM2	0.0293774	1.33846
231932_at	TRAF3IP3	0.0001718	1.48917
231940_at	ZNF529	0.0108082	-2.10393
231944_at	ERO1LB	0.0000086	-3.7346
231945_at	FILIP1	0.0055214	3.41121
231950_at	ZNF658	0.0000059	-4.61442
231960_at	BRWD1	0.0199086	-1.95523
231961_at	RBPMS	0.0004124	-1.65611
231966_at	PPP1R9A	0.0589301	1.23113
231986_at	RIMS1	0.0000058	-1.67833
231990_at	USP15	0.0001657	-1.92763
232004_at	HNRNPR	0.0000134	-5.80194
232008_s_at	BBX	0.0172478	-2.17836
232014_at	ZNF30	0.0001179	-1.72275
232017_at	TJP2	0.000651	-2.47825
232024_at	GIMAP2	0.0001841	-6.56256
232027_at	SYNE1	0.0008257	-1.91514
232035_at	HIST1H4H	0.0019796	2.5601
232037_at	IGDCC3	0.0004296	1.37289

232038_at	C6orf170	0.0135339	-2.17388
232048_at	FAM76B	0.0861481	1.3077
232054_at	PCDH20	0.000043	-3.94336
232057_at	SLC7A6OS	0.0259896	-1.97856
232059_at	DSCAML1	0.0000138	-3.66419
232060_at	ROR1	0.0000925	-6.9745
232071_at	MRPL19	0.000436	-2.86963
232080_at	HECW2	0.0513826	1.32615
232087_at	CXorf23	0.0030383	-2.05
232099_at	PCDHB16	0.0011327	-6.08346
232136_s_at	CTTNBP2	0.0288102	-2.1541
232157_at	SPRY3	0.0057904	1.19488
232164_s_at	EPPK1	0.0552092	1.80239
232180_at	UGP2	0.000205	-3.49933
232184_at	ALS2	0.0092015	-1.93841
232195_at	GPR158	0.0281594	2.60236
232201_at	NKD2	0.000566	1.63586
232207_at	GUSBL2	0.0155833	1.38597
232212_at	PLEKHA8	0.012542	1.23704
232235_at	DSEL	0.0031464	-2.67022
232247_at	ZNF502	0.0002242	-2.79778
232267_at	GPR133	0.0000016	-4.83123
232270_at	C9orf3	0.0025041	-2.05498
232289_at	KCNJ12	0.02075	1.41503
232291_at	MIR17HG	0.0448616	1.76427
232293_at	LCORL	0.0016447	-1.80975
232297_at	KLHL5	0.0024167	-1.87029
232305_at	HMGCLL1	0.0059248	-2.07541
232313_at	TMEM132C	0.0218199	1.48434
232317_at	PLXNA4	0.0041962	-2.03163
232327_at	THSD7B	0.050925	1.72398
232366_at	KIAA0232	0.0111483	-2.43735
232382_s_at	PCMTD1	0.0227513	-2.24222
232388_at	CNTNAP4	0.0000016	-14.3742
232424_at	PRDM16	0.0526381	1.53966
232449_at	BCO2	0	-5.81541
232458_at	COL3A1	0.0000001	-16.6091
232481_s_at	SLITRK6	0.0033869	-1.84146
232523_at	MEGF10	0.0154416	2.38152
232560_at	UROS	0.0243729	1.30052

232568_at	MGC24103	0.000003	-10.6372
232610_at	PARP14	0.0972078	1.27486
232641_at	ZNF596	0.0007517	-1.75448
232695_at	KIF6	0.0654354	1.21387
232740_at	MCM3APAS	0.0756707	1.22292
232770_at	TUSC3	0.0000313	-1.96166
232829_at	OR52K3P	0.0160711	-2.09883
232865_at	AFF4	0.0116401	-2.93594
232899_at	FAM41C///RPL:	0.0060458	1.9269
232922_s_at	SLC17A9	0.0379185	1.93054
233011_at	ANXA1	0.0001613	-2.62937
233019_at	CNOT7	0.0021427	-3.23154
233025_at	PDZD2	0.0093072	-2.01788
233070_at	ZNF197	0.0003654	-1.77263
233089_at	QRSL1	0.0843179	1.26819
233093_s_at	BIRC6	0.000755	1.85118
233111_at	PTCSC	0.0011001	1.40456
233136_at	PABPC5	0.0002077	-3.11684
233141_s_at	ST7L	0.000364	-1.71267
233168_s_at	SELO	0.0441791	1.56388
233191_at	RUFY2	0.0023525	-2.05748
233301_at	OXCT2	0.0282033	1.40297
233329_s_at	KRCC1	0.0006045	-4.11827
233337_s_at	SEZ6L2	0.0414658	1.30624
233496_s_at	CFL2	0.0002298	-2.65622
233520_s_at	CMYA5	0.0000008	-3.39262
233536_at	ASXL3	0.0000401	-4.91898
233647_s_at	CDADC1	0.0007816	-2.08708
233656_s_at	VPS54	0.0004931	-1.95625
233734_s_at	OSBPL5	0.0413527	2.10374
233759_s_at	SMEK2	0.0111692	-2.66449
233841_s_at	SUDS3	0.0060126	1.45299
233849_s_at	ARHGAP5	0.0108526	-2.40932
233914_s_at	SBF2	0.0084284	-1.91091
233919_s_at	HABP4	0.0053721	-1.90232
233952_s_at	ZNF295	0.0113212	-2.51462
234000_s_at	PTPLAD1	0.0002985	-4.76792
234018_s_at	SEL1L2	0.0000594	-9.68078
234103_at	KCNT2	0.0000192	-14.7855
234140_s_at	STIM2	0.0001668	-1.81353

234165_at	PTGDR	0.0000851	-1.93248
234225_at	KIFC1	0.0700766	1.25029
234305_s_at	GSDMC	0.0024828	1.6702
234308_at	TUBGCP6	0.0434112	1.42968
234464_s_at	EME1	0.000002	5.19445
234617_at	OR52D1	0.0954859	1.29746
234661_at	CCDC57	0.0033663	1.28107
234709_at	CAPN13	0.0225063	2.27566
234734_s_at	TNRC6A	0.0036453	-1.7465
234946_at	ENTPD6	0.0014567	1.43446
234950_s_at	RFWD2	0.0009451	1.41991
234974_at	GALM	0.0785827	1.31149
234978_at	SLC36A4	0.0000527	-3.57981
234982_at	UBR3	0.0006058	-3.83901
234988_at	VCPIP1	0.0190424	-2.07661
234994_at	TMEM200A	0.0355418	-3.63742
234995_at	CCDC52	0.0006735	2.46243
235005_at	DIS3L	0.0004824	-2.37365
235007_at	BBS7	0.0002717	-2.81114
235032_at	DNAJC21	0.0078172	1.56831
235034_at	VTI1A	0.0062004	1.37669
235051_at	CCDC50	0.0001631	-3.21254
235065_at	FAM59A	0.0005387	-2.65893
235074_at	SPRED1	0.0003035	-2.2999
235076_at	CALCOCO2	0.0000339	-3.31013
235095_at	CCDC64B	0.0000042	1.62897
235096_at	LEO1	0.0082907	-1.94828
235131_at	RHOJ	0.0186629	1.33034
235142_at	ZBTB8A	0.0036489	-2.87149
235146_at	TMCC3	0.0333398	2.74031
235148_at	KRTCAP3	0.0663799	1.42335
235153_at	RNF183	0.0024092	4.51161
235164_at	ZNF25	0.0000718	-2.35097
235165_at	PARD6B	0.005308	-1.9633
235176_at	ZFP82	0.0004447	-1.70692
235177_at	FAM119A	0.0175627	1.99051
235182_at	ISM1	0.0675498	1.56611
235198_at	OSTM1	0.0002018	-2.74522
235199_at	RNF125	0.0152328	-1.95351
235219_at	C5orf55	0.0800375	1.65688

235233_s_at	GMEB1	0.0005826	-2.61655
235240_at	ATXN3	0.0000555	-2.54651
235241_at	SLC38A9	0.0041638	-2.01423
235245_at	TMEM92	0.03438	1.54669
235252_at	KSR1	0.0273706	1.54111
235263_at	STAG3L1	0.0027334	1.38192
235276_at	EPSTI1	0.0038228	-2.20395
235289_at	EIF5A2	0.0007132	-1.84342
235290_at	ZNF782	0.0026883	-1.9858
235308_at	ZBTB20	0.0220275	-2.4076
235309_at	RPS15A	0.0002183	-4.08575
235314_at	RPL32P3	0.0002766	1.85489
235318_at	FBN1	0.000091	-3.10541
235320_at	ARL6	0.0005747	-1.73507
235332_at	FAM22A///FAM:	0.0199399	1.33808
235333_at	B4GALT6	0.0000973	-2.3383
235334_at	ST6GALNAC3	0.0000036	-3.79874
235338_s_at	SETDB2	0.000725	-2.16145
235342_at	SPOCK3	0.0006024	-6.91353
235346_at	FUNDC1	0.0176741	-2.93563
235348_at	ABHD13	0.0002819	-2.86735
235350_at	C4orf19	0.0000839	-4.71245
235374_at	MDH1	0.0065952	-2.10699
235391_at	FAM92A1	0.0002546	-2.42821
235410_at	NPHP3	0.0098002	-2.4693
235421_at	MAP3K8	0.0031605	-1.80277
235433_at	APOOL	0.0008347	-2.16228
235457_at	MAML2	0.0956979	1.77584
235494_at	LSAMP	0.0000135	-1.77284
235508_at	PML	0.0194599	1.69914
235512_at	CDKL1	0.0005064	-2.83323
235521_at	HOXA3	0.0832156	2.10439
235528_at	GUCA1B	0.0516876	1.23661
235542_at	TET3	0.0032854	1.68566
235552_at	METTL14	0.0098941	-1.99752
235569_at	VPS37D	0.0003203	2.14915
235572_at	SPC24	0.0003523	2.50251
235603_at	HNRNPU	0.0018982	-1.68439
235611_at	SFRS12	0.0017902	-2.01462
235615_at	PGGT1B	0.0237093	-2.2701

235625_at	VPS41	0.0002044	-2.06014
235648_at	ZNF567	0.002013	-3.33564
235675_at	DHFRL1	0.0000802	-3.64943
235688_s_at	TRAF4	0.0059493	1.45758
235698_at	ZFP90	0.0016427	-3.96912
235733_at	GXYLT2	0.0026007	-2.467
235763_at	SLC44A5	0.0039707	-4.23281
235764_at	PRDM5	0.0000036	-7.92852
235798_at	TMEM170B	0.0094946	-1.78816
235799_at	NSL1	0.0378499	1.66466
235809_at	LIN54	0.0091012	1.18917
235810_at	ZNF182	0.0121477	1.22403
235812_at	TMEM188	0.0053248	-3.38959
235833_at	PPAT	0.017668	1.30869
235850_at	FAM162A	0.0002662	-3.06149
235867_at	GSTM3	0	-4.39407
235874_at	PRSS35	0.0000024	-13.8351
235888_at	GUSBP1	0.0077864	-2.37583
235890_at	TBL1XR1	0.0020424	-2.41572
235911_at	MFI2	0.0050423	-2.56525
235913_at	ZNF880	0.0265996	1.27687
235962_at	AZI2	0.00002	-2.24004
235977_at	LONRF2	0.0026944	-4.51578
235978_at	FABP4	0.0072111	-1.80134
235980_at	PIK3CA	0.0025884	-2.05438
236007_at	AKAP10	0.0138864	-2.16669
236019_at	RAB12	0.0021798	-1.6937
236034_at	ANGPT2	0.0011749	1.49262
236044_at	PPAPDC1A	0.0855728	2.20298
236046_at	FLJ44896	0.0005611	-3.20014
236087_at	ABLIM2	0.0591797	1.71227
236088_at	NTNG1	0.0000084	-3.4134
236115_at	HTR7P1	0.0000965	-2.10932
236154_at	QKI	0.0000479	-2.17145
236160_at	TRIP11	0.0211793	-2.00713
236163_at	LIX1	0.0381413	2.72191
236179_at	CDH11	0.0119244	-1.85535
236232_at	STX4	0.02665	1.2904
236241_at	MED31	0.0009059	-2.53028
236247_at	NSUN4	0.0205189	1.2365

236254_at	VPS13B	0.0420595	-2.15327
236265_at	SP4	0.0040382	-1.998
236290_at	DOK6	0.0008902	-2.17792
236302_at	PPM1E	0	-12.4746
236325_at	KIAA1377	0.001797	-2.17177
236328_at	ZNF285	0.0000028	-4.95142
236331_at	CDKL2	0.0000297	-5.44643
236471_at	NFE2L3	0.0608623	1.49644
236492_at	PPP2R2A	0.0060746	-2.23448
236514_at	ACOT8	0.0149096	2.29046
236548_at	GIPC2	0.0000074	-4.09226
236556_s_at	LONRF1	0.0914814	1.89789
236562_at	ZNF439	0.043651	2.05961
236565_s_at	LARP6	0.0000876	5.41106
236600_at	SPG20	0.0000526	-4.19902
236604_at	BAHCC1	0.0379189	1.8915
236634_at	C8orf48	0.0008581	-2.2073
236641_at	KIF14	0.0002496	2.2763
236642_at	FLJ44606	0.0262707	1.24815
236651_at	KALRN	0.0007849	1.7289
236728_at	LNPEP	0.048043	1.41922
236814_at	MDM4	0.0167375	-2.34216
236834_at	SCFD2	0.0010743	-2.13896
236933_at	EVPLL	0.0516868	1.54073
237003_at	BEST3	0.0130189	1.24435
237045_at	FAM91A1	0.0020564	1.32651
237054_at	ENPP5	0.0012831	-3.92283
237107_at	PRKRA///PRKR	0.0474068	-2.47291
237252_at	THBD	0.0002677	-2.94905
237291_at	PRDXDD1P	0.0191899	1.96912
237411_at	ADAMTS6	0.0852729	1.41101
237461_at	NLRP7	0.0024442	1.34017
237465_at	USP53	0.0015248	-3.41575
237515_at	TMEM56	0.0008969	-2.03723
237654_at	C14orf50	0.0000008	-3.14201
237715_at	AKNA	0.0241851	1.38006
237732_at	PRR9	0.0322523	-2.41642
237783_at	PLAC8L1	0.075144	1.33817
237802_at	XKR4	0.0032101	2.66775
237819_at	CREB3L2	0.010198	1.28329

237974_at	ABHD12B	0	-24.4798
238004_at	PGBD2	0.0172142	1.57843
238018_at	FAM150B	0.0293633	-2.23937
238022_at	CRNDE	0.0000002	-6.11372
238026_at	RPL35A	0.0009937	-3.49154
238030_at	ZNF268	0.002203	-2.11855
238035_at	SP3	0.0007179	-3.64514
238041_at	TCF12	0.0003964	-2.12913
238045_at	TMEM65	0.0021858	1.68639
238063_at	TMEM154	0.0264586	-2.4613
238066_at	RBP7	0.0013077	-2.65062
238071_at	LCN10///LCN6	0.0228137	1.19987
238077_at	KCTD6	0.0012391	-4.50605
238081_at	C4orf12	0.0001321	-3.59061
238116_at	DYNLRB2	0.0000405	-4.41748
238124_at	MYOM3	0.0173154	2.00043
238147_at	TRIM46	0.0453984	1.61475
238148_s_at	ZNF818P	0.0018562	-2.46963
238153_at	PDE6B	0.0022971	1.28079
238158_at	MEIG1	0.0178016	1.58622
238197_at	GATA5	0.0020204	-2.25148
238205_at	DCAF12L1	0.0413985	1.8545
238273_at	PL-5283///SLC	0.0000094	3.38435
238295_at	C17orf42	0.0097276	-1.8737
238332_at	ANKRD29	0	-12.9705
238333_s_at	SPRN	0.0046907	1.71417
238353_at	RASL11A	0.0012756	1.27002
238356_at	DOCK11	0.0017493	-2.55693
238419_at	PHLDB2	0.0035301	-1.83724
238432_at	FLJ35776	0.0049426	1.51224
238435_at	CA5B///CA5BP	0.0401556	1.33688
238436_s_at	ZNF805	0.005124	-2.33865
238454_at	ZNF540	0.0000003	-2.89893
238458_at	EFHA2	0.0000198	-5.49351
238472_at	FBXO9	0.0017717	-1.856
238483_at	SSBP2	0.0039394	-2.09647
238497_at	TMEM136	0.0037653	-2.09981
238519_at	DDI2	0.0027654	-1.70651
238520_at	TRERF1	0.0075412	-2.85011
238523_at	KLHL36	0.000003	-1.90188

238584_at	IQCA1	0.0115335	-3.34228
238592_at	PDLIM3	0.0000049	-2.97003
238609_at	FAM200A	0.0030622	-1.79354
238625_at	C1orf168	0	-88.9619
238647_at	C14orf28	0.0251322	-1.9826
238649_at	PITPNC1	0	-10.866
238657_at	UBXN10	0.0002818	-5.31431
238669_at	PTGS1	0.0034434	-2.64676
238681_at	GDPD1	0.0019036	-2.92568
238691_at	SNHG10	0.0005296	1.44402
238694_at	DGKE	0.002706	1.415
238719_at	PPP2CA	0.0009934	-3.18115
238738_at	PSMD7	0.0014408	-1.84154
238750_at	CCL28	0.0144565	2.06277
238756_at	GAS2L3	0.000148	6.79275
238787_at	DENND1B	0.0015857	-2.65918
238803_at	HECTD2	0.0001919	-3.4163
238805_at	C11orf52	0.000173	-2.50156
238819_at	ZNF347	0.0054723	-1.82665
238868_at	UACA	0.000081	-2.86485
238871_at	MLLT4	0.0596413	1.59601
238877_at	EYA4	0.0740207	2.41151
238878_at	ARX	0.0000031	-38.529
238880_at	GTF3A	0.0019597	-3.08819
238886_at	TMED10	0.0069463	-1.88337
238903_at	UBXN2B	0.0049958	-2.32354
238935_at	RPS27L	0.0092755	-2.25082
238937_at	ZNF420	0.0117588	-2.04995
238973_s_at	TSNAX	0.0004659	1.74662
238992_at	POLI	0.0001062	-4.42808
239002_at	ASPM	0.0061603	2.80291
239006_at	SLC26A7	0.0010098	4.66814
239010_at	DUXAP10	0.0216939	3.16907
239013_at	SEC22C	0.006817	1.66676
239018_at	DNAJC30	0.0021053	-1.91765
239028_at	LYPD6	0.0014138	1.78645
239043_at	ZNF404	0.00414	-1.85807
239067_s_at	PANX2	0.00471	1.39369
239114_at	SERGEF	0.064583	1.15179
239130_at	MIR101-1	0.0018617	-2.71752

239148_at	MARVELD3	0.0008668	2.01206
239177_at	IRGQ	0.0250909	1.36771
239178_at	FGF9	0.0013543	-8.07462
239190_at	VRK3	0.0001226	1.42845
239201_at	CDK15	0.0007676	-1.7425
239229_at	PHEX	0.0014552	1.43962
239246_at	FARP1	0.0111494	2.17344
239247_at	NCRNA00103	0.0576829	1.27883
239250_at	ZNF542	0.0208944	-2.93267
239252_at	COX7B	0.0027491	-2.22601
239273_s_at	MMP28	0.0000054	-2.94594
239283_at	TMED5	0.0000195	-2.01579
239349_at	C1QTNF7	0.0013485	-2.34031
239355_at	GMCL1	0.0006018	-2.76
239381_at	KLK7	0.0003099	8.49025
239398_at	KLHL31	0.0000168	-3.60696
239400_at	FLJ45513	0.0614866	1.23692
239422_at	GPC2	0.0016357	2.53512
239433_at	LRRC8E	0.0814692	1.50591
239441_at	ZNF780A	0.0016035	-2.00647
239481_at	FAM133A	0.0002853	-5.26698
239487_at	FAM98A	0.0317379	-2.47797
239537_at	ST8SIA2	0.0027831	1.80692
239542_at	ITPR3	0.002772	1.27666
239552_at	VWDE	0.0182971	1.35351
239629_at	CFLAR	0.0326937	-2.832
239654_at	CHD9	0.0149503	-2.63527
239660_at	RALGAPA2	0.0420386	-2.22217
239699_s_at	PMS2L1///PMS	0.0378637	1.25146
239757_at	ZFAND6	0.0360424	-2.15459
239761_at	GCNT1	0.0000038	-3.18722
239763_at	PRDM11	0.0865531	1.41057
239839_at	ZNF555	0.0000132	-1.58709
239883_s_at	ANO4	0.0086187	1.59557
240061_at	PION	0.0061198	-2.26795
240117_at	FBN3	0.0008976	2.24811
240172_at	ERGIC2	0.0009711	-1.6562
240204_at	SNRPN	0.0434302	1.25536
240214_at	RWDD1	0.0000023	-3.72091
240221_at	CSNK1A1	0.0175225	-2.10814

240363_at	ANK1	0.0119049	1.39152
240383_at	UBE2D3	0.0116239	-2.17708
240420_at	AADACL2	0.0000061	-5.69814
240429_at	ZNF546	0.0000358	-1.81174
240554_at	AKAP8L	0.0158737	1.38615
240650_at	CACNA1E	0.0507288	1.19554
240757_at	CLASP1	0.0313983	1.15517
240801_at	C21orf37	0.0015631	1.26946
241150_at	SPTAN1	0.0264188	1.61292
241348_at	ZNF654	0.0151608	-2.24048
241359_at	TLCD2	0.0003012	-2.28083
241368_at	PLIN5	0.0499866	1.20091
241372_at	ZC3H6	0.0002774	-1.72206
241412_at	BTC	0.0142849	-2.51222
241425_at	NUPL1	0.0149682	-3.64111
241433_at	RCOR3	0.0128489	1.89262
241450_at	RSPO1	0	-10.8075
241672_at	C13orf36	0	-93.4932
241741_at	CRLS1	0.0003876	-2.40703
241746_at	CUL7	0.0316228	1.32399
241751_at	OFD1	0.0000528	-2.8222
241789_at	RBMS3	0.0064964	-2.09001
241805_at	GABRG1	0	-20.4968
241808_at	FAM164A	0.0007913	-2.23124
241820_at	RIF1	0.0017131	1.28145
241827_at	ZNF615	0.0000132	-3.29387
241834_at	IPW	0.0013055	-2.6459
241898_at	LIPH	0.0229162	1.27613
241905_at	PIK3C2A	0.0220388	-2.61982
241978_at	AKR1A1	0.045318	1.18447
241981_at	FAM20A	0.0148378	-3.3025
241992_at	DRAM1	0.0000023	-2.81618
241994_at	XDH	0.069638	1.97954
242002_at	NKAIN2	0.0000477	-3.15864
242028_at	ZNF709	0.0037753	-2.19285
242045_at	ANKRD18A	0.010488	1.39958
242141_at	HDAC2	0.0157719	1.41141
242190_at	SDAD1	0.0188922	1.24634
242201_at	PMS2L2	0.0001986	-1.63447
242214_at	RPS27A	0.0010632	-2.39524

242268_at	CELF2	0.0004607	-4.02493
242271_at	SLC26A9	0.0132731	1.64212
242286_at	GRIN2A	0.0000167	-5.20162
242290_at	TACC1	0.0022464	-1.98259
242308_at	MCOLN3	0.0018133	-1.66961
242443_at	EML5	0.006593	-1.82551
242447_at	C3orf70	0.001291	-2.8032
242470_at	EID2B	0.0021344	-2.26022
242482_at	PRKAR1A	0.0000079	-2.00486
242524_at	CBLN4	0.0000564	-3.50852
242546_at	FLJ39632	0.0247436	2.71453
242560_at	FANCD2	0.0000008	6.98315
242592_at	GPR137C	0.0056138	1.8775
242750_at	MMAA	0.000874	-1.71185
242752_at	PPM1K	0.0101364	-1.9996
242766_at	ERLEC1P1	0.0017568	1.37716
242767_at	LMCD1	0.0306071	1.52986
242794_at	MAML3	0.0004726	-5.02541
242828_at	FIGN	0.0138586	-2.96463
242838_at	MAP6D1	0.0831655	1.47451
242843_at	BCAN	0.0171816	1.6834
242855_at	KCP	0.0558748	1.53971
242905_at	PNO1	0.074464	1.74795
242912_at	P704P	0.0138497	2.04611
242915_at	ZNF682	0.0008046	2.75592
242939_at	TFDP1	0.0022688	2.25511
242953_at	ZNF234	0.0002748	-2.16897
242957_at	VWCE	0.0000707	3.04883
243166_at	SLC30A5	0.0019163	-2.63986
243188_at	ZNF283	0.0040805	-2.25296
243194_at	ZNF551	0.0005713	1.38327
243209_at	KCNQ4	0.0006157	1.64191
243237_at	PCDP1	0.0000062	-4.6902
243264_s_at	C8orf44///SGK3	0.0013988	-1.65288
243309_at	FLJ27352	0.0015618	-1.78512
243357_at	NEGR1	0.0002626	-2.07353
243386_at	CASZ1	0.0078234	1.80509
243437_at	GCC1	0.0060313	1.38345
243438_at	PDE7B	0.0006344	-1.8223
243521_at	ZXDA	0.0045276	-2.12687

243531_at	ORAOV1	0.0172577	2.12687
243582_at	SH3RF2	0.0000015	-5.66279
243606_at	FAM55C	0.0003892	-1.99805
243619_at	FGFR1OP2	0.0007994	-1.63012
243624_at	PIAS2	0.0417891	1.29792
243772_at	SDCCAG8	0.001786	1.56458
243790_at	ZNF585A	0.023851	-2.14521
243843_at	N4BP2L1	0.0000007	-6.93725
243864_at	CCDC80	0.0000001	-4.33698
243_g_at	MAP4	0.0009791	1.71547
244050_at	PTPLAD2	0.0266466	-2.23178
244214_at	PELI3	0.0107794	1.33172
244317_at	KIAA1324L	0.0670865	1.26848
244353_s_at	SLC2A12	0.0163147	1.45406
244377_at	SLC1A4	0.0459088	1.31295
244406_at	ZNF20///ZNF62	0.0007416	1.87299
244407_at	CYP39A1	0.0259614	-2.60795
244509_at	GPR155	0.0134593	1.30772
244533_at	PTPN14	0.0023978	-1.81843
244552_at	ZNF788	0.0021844	-1.71688
244597_at	SPATS2L	0.0019805	-2.00988
244704_at	NFYB	0.0028635	-2.04133
244710_at	LRGUK	0.0079867	1.58088
244738_at	BRWD3	0.0374175	1.44087
244745_at	RERG	0.0000017	-10.9739
244764_at	HIVEP3	0.0000011	-3.17954
244777_at	DCP2	0.007606	-2.40138
244804_at	SQSTM1	0.0204355	1.33606
244825_at	SHROOM4	0.0001806	1.65943
244881_at	LMLN	0.0077561	-3.12397
32088_at	BLZF1	0.0008173	-1.74243
32099_at	SAFB2	0.0006391	1.5914
32137_at	JAG2	0.0001146	1.6377
32209_at	FAM89B	0.01646	1.26572
32837_at	AGPAT2	0.0160866	1.38433
33132_at	CPSF1	0.0020012	1.84228
33307_at	RRP7A	0.0011908	1.52828
33323_r_at	SFN	0.0000027	13.8857
33736_at	STOML1	0.0055265	1.83563
33778_at	TBC1D22A	0.0114591	-1.99319

34221_at	HMGXB3	0.0661072	1.53472
34408_at	RTN2	0.048453	1.31959
34726_at	CACNB3	0.0078012	-2.31465
34858_at	KCTD2	0.0000468	-1.99692
34868_at	SMG5	0.0014057	1.77585
35148_at	TJP3	0.0628824	1.5771
35150_at	CD40	0.0082659	1.12702
35156_at	R3HCC1	0.0053107	-1.87313
35201_at	HNRNPL	0.0120893	1.16945
35254_at	TRAFD1	0.0119264	1.85518
35666_at	SEMA3F	0.001073	5.15238
35685_at	RING1	0.0813773	1.2105
35820_at	GM2A	0.0015028	-1.68672
36019_at	STK19	0.0020209	1.36223
36499_at	CELSR2	0.0044143	1.61327
36552_at	C2CD3	0.0007982	2.05181
36830_at	MIPEP	0.000507	-2.12141
36936_at	TSTA3	0.0005369	1.78991
37004_at	SFTPB	0.0459988	1.2319
37117_at	ARHGAP8///PR	0.0001052	2.08436
37152_at	PPARD	0.0003584	2.95907
37425_g_at	CCHCR1	0.003323	1.76471
37549_g_at	BBS9	0.0000199	-2.67797
37652_at	CABIN1	0.0525161	1.26833
37872_at	JRK	0.0255105	1.63215
37892_at	COL11A1	0.0434893	-3.40248
37943_at	ZFYVE26	0.0000027	-2.0585
38069_at	CLCN7	0.0095575	1.63943
38157_at	DOM3Z	0.0136307	1.42973
38158_at	ESPL1	0.0000026	4.97616
38290_at	RGS14	0.001143	1.39187
38671_at	PLXND1	0.0017045	1.72121
38703_at	DNPEP	0.0304966	1.53378
38710_at	OTUB1	0.0385011	1.49361
38964_r_at	WAS	0.0595317	1.32787
39402_at	IL1B	0.069939	1.24356
39549_at	NPAS2	0.0974874	1.60575
39582_at	CYLD	0.0002318	-2.48069
39650_s_at	PCNXL2	0.0263918	1.42648
39817_s_at	C6orf108	0.007657	1.87587

39891_at	ZNF710	0.0013918	1.77389
39966_at	CSPG5	0.0029274	3.33066
40020_at	CELSR3	0.0000528	2.28816
40093_at	BCAM	0.0357216	1.35564
40225_at	GAK	0.0046212	2.00906
40273_at	SPHK2	0.0052956	1.61215
40560_at	TBX2	0.01011	2.34783
40829_at	WDTC1	0.0697535	1.30531
41047_at	C9orf16	0.0052489	1.83719
41220_at	Sep 9, 2013	0.0168789	1.58783
41387_r_at	KDM6B	0.0416308	1.3947
41660_at	CELSR1	0.0045022	2.08419
41858_at	PGAP2	0.0902415	1.2968
43977_at	TMEM161A	0.0056746	1.66373
44040_at	FBXO41	0.0051807	1.64069
44111_at	VPS33B	0.0874013	1.21456
44146_at	GMEB2	0.0000381	1.85457
44563_at	WRAP53	0.0062957	1.53305
45288_at	ABHD6	0.0001117	-2.16988
45572_s_at	GGA1	0.0059655	1.6197
45653_at	KCTD13	0.076697	1.21514
45687_at	PRR14	0.0086511	1.57402
46665_at	SEMA4C	0.0005812	2.71835
47069_at	PRR5	0.0339254	1.19535
47550_at	LZTS1	0.0006413	2.81713
47560_at	LPHN1	0.0006338	2.09594
47608_at	TJAP1	0.0025456	1.99902
48106_at	SLC48A1	0.0307145	1.47693
48531_at	TNIP2	0.0135021	1.72336
48825_at	ING4	0.0001078	1.98739
49077_at	PPME1	0.002245	1.49743
49878_at	PEX16	0.0261831	1.57435
50314_i_at	C20orf27	0.0614817	1.40802
50374_at	C17orf90	0.0055815	1.54712
50376_at	ZNF444	0.0064161	1.66141
50400_at	PAOX	0.0801972	1.34585
50965_at	RAB26	0.0013544	1.70388
51200_at	C19orf60	0.007255	2.34549
52159_at	HEMK1	0.0463421	1.42999
52164_at	C11orf24	0.0163819	1.5388

52651_at	COL8A2	0.043669	1.23418
52741_at	TRMT61A	0.0343741	1.19248
52837_at	KIAA1644	0.0002608	2.17828
53202_at	C7orf25///PSM/	0.0118777	-1.83772
53968_at	INTS5	0.0008344	1.83929
54970_at	ZMIZ2	0.0041314	2.1216
55065_at	MARK4	0.0997044	1.4352
55081_at	MICALL1	0.0071403	1.77943
55616_at	PGAP3	0.0535811	1.95144
55705_at	C19orf22	0.016751	1.37604
55872_at	ZNF512B	0.0002629	2.84551
56256_at	SIDT2	0.0269065	1.53351
57082_at	LDLRAP1	0.0019195	1.54703
57163_at	ELOVL1	0.0618689	1.50203
57532_at	DVL2	0.0126537	1.78207
57739_at	DND1	0.0145236	1.71502
58994_at	CC2D1A	0.0795388	2.08362
59437_at	C9orf116	0.0142112	1.32746
59625_at	NOL3	0.0233454	1.5009
59697_at	RAB15	0.0000022	1.78338
60471_at	RIN3	0.0608253	1.34382
60474_at	FERMT1	0.0016145	-2.34393
60528_at	JMJD7-PLA2G4	0.0115569	1.16568
61874_at	C9orf7	0.0799207	1.24281
62987_r_at	CACNG4	0.0122718	1.76822
632_at	GSK3A	0.012257	1.2647
635_s_at	PPP2R5B	0.0035014	1.52403
64474_g_at	DGCR8	0.0004234	1.52409
64486_at	CORO1B	0.0208973	1.53548
65635_at	ENGASE	0.002046	1.48209
65718_at	GPR124	0.0518699	1.15251
823_at	CX3CL1	0.0000426	1.93472
91826_at	EPS8L1	0.035378	1.98606
