# Supplementary material for: Transcriptional override: a regulatory network model of indirect responses to modulations in microRNA expression
Source: BMC Syst Biol. 2014 Mar 25;8:36. doi: 10.1186/1752-0509-8-36 (PMC3987680; doi:10.1186/1752-0509-8-36)
Supplement: Additional file 3 — List of genes that are 1) targets of one-or more of the 31 upregulated miRNAs, 2) downregulated in our cancer samples and 3) previously characterized as transcriptional repressors [[14]]. Genes belonging to gene ontology categories GO:0045892, GO:0000122, GO:0010944, GO:0032088 and GO:0008156, relating to the negative-regulation-of-transcription, were downloaded from the European Bioinformatics Institute. Downregulated repressor genes that were also predicted microRNA targets formed the 105 transcriptional repressor genes presented in this list. [file 1752-0509-8-36-S3.pdf]

Additional file 3

| Probeset_ID | Gene-Symbol | p-value | Fold-change |
|---|---|---|---|
| 230141_at | ARID4A | 0.00034 | -4.29 |
| 209988_s_at | ASCL1 | 0.00612 | -4.03 |
| 203232_s_at | ATXN1 | 0.00005 | -2.45 |
| 204194_at | BACH1 | 0.00056 | -3.65 |
| 236796_at | BACH2 | 0.00371 | -3.07 |
| 202391_at | BASP1 | 0.03447 | -2.44 |
| 201084_s_at | BCLAF1 | 0.00049 | -3.24 |
| 205289_at | BMP2 | 0.00000 | -8.73 |
| 207186_s_at | BPTF | 0.00173 | -6.64 |
| 224471_s_at | BTRC | 0.00011 | -2.84 |
| 212914_at | CBX7 | 0.00029 | -2.58 |
| 235196_at | CDC73 | 0.00010 | -3.72 |
| 209112_at | CDKN1B | 0.00059 | -4.40 |
| 204314_s_at | CREB1 | 0.02689 | -2.09 |
| 209674_at | CRY1 | 0.00696 | -1.77 |
| 201278_at | DAB2 | 0.00000 | -22.47 |
| 200033_at | DDX5 | 0.00936 | -3.69 |
| 204602_at | DKK1 | 0.00861 | -2.08 |
| 204273_at | EDNRB | 0.00336 | -5.25 |
| 208669_s_at | EID1 | 0.00000 | -13.74 |
| 212420_at | ELF1 | 0.03501 | -2.80 |
| 203822_s_at | ELF2 | 0.00188 | -3.77 |
| 225159_s_at | ELK4 | 0.00242 | -2.10 |
| 225417_at | EPC1 | 0.00295 | -1.74 |
| 209455_at | FBXW11 | 0.00119 | -2.17 |
| 202949_s_at | FHL2 | 0.00178 | -2.83 |
| 226460_at | FNIP2 | 0.00038 | -3.13 |
| 218031_s_at | FOXN3 | 0.00383 | -2.28 |
| 224891_at | FOXO3 | 0.00195 | -2.23 |
| 235444_at | FOXP1 | 0.00000 | -5.64 |
| 210002_at | GATA6 | 0.00000 | -56.60 |
| 225884_s_at | GZF1 | 0.00700 | -2.74 |
| 228813_at | HDAC4 | 0.00098 | -2.51 |
| 226648_at | HIF1AN | 0.00146 | -1.96 |
| 219269_at | HMBOX1 | 0.01083 | -2.39 |
| 210338_s_at | HSPA8 | 0.00023 | -5.40 |
| 201565_s_at | ID2 | 0.01252 | -2.02 |
| 209292_at | ID4 | 0.00001 | -16.97 |
| 206332_s_at | IFI16 | 0.00000 | -7.22 |
| 225798_at | JAZF1 | 0.00009 | -2.70 |
| 218486_at | KLF11 | 0.00043 | -2.37 |
| 221841_s_at | KLF4 | 0.00021 | -5.16 |
| 222561_at | LANCL2 | 0.00009 | -2.13 |
| 209348_s_at | MAF | 0.00000 | -10.56 |
| 236814_at | MDM4 | 0.01674 | -2.34 |
| 212535_at | MEF2A | 0.00002 | -4.99 |

| | | | |
|---|---|---|---|
| 209200_at | MEF2C | 0.00000 | -5.66 |
| 212251_at | MTDH | 0.00219 | -4.70 |
| 219363_s_at | MTERFD1 | 0.00816 | -2.04 |
| 212993_at | NACC2 | 0.00055 | -4.15 |
| 200854_at | NCOR1 | 0.00233 | -1.77 |
| 213012_at | NEDD4 | 0.00008 | -1.73 |
| 213032_at | NFIB | 0.00147 | -5.00 |
| 203574_at | NFIL3 | 0.00582 | -2.27 |
| 209239_at | NFKB1 | 0.00116 | -1.66 |
| 223439_at | NKAP | 0.01086 | -1.99 |
| 209706_at | NKX3-1 | 0.00000 | -20.57 |
| 206645_s_at | NR0B1 | 0.00000 | -6.22 |
| 212594_at | PDCD4 | 0.00000 | -4.50 |
| 210296_s_at | PEX2 | 0.00184 | -6.97 |
| 228469_at | PPID | 0.00001 | -2.87 |
| 235764_at | PRDM5 | 0.00000 | -7.93 |
| 223275_at | PRMT6 | 0.00698 | -2.27 |
| 219485_s_at | PSMD10 | 0.02809 | -3.19 |
| 207785_s_at | RBPJ | 0.00007 | -3.50 |
| 204633_s_at | RPS6KA5 | 0.00353 | -1.98 |
| 222540_s_at | RSF1 | 0.06553 | -2.57 |
| 203408_s_at | SATB1 | 0.00003 | -6.71 |
| 40189_at | SET | 0.00288 | -3.52 |
| 223122_s_at | SFRP2 | 0.00001 | -18.40 |
| 218878_s_at | SIRT1 | 0.00108 | -2.71 |
| 203076_s_at | SMAD2 | 0.00224 | -2.20 |
| 202527_s_at | SMAD4 | 0.00503 | -3.04 |
| 206542_s_at | SMARCA2 | 0.00005 | -8.79 |
| 211988_at | SMARCE1 | 0.00006 | -3.90 |
| 213139_at | SNAI2 | 0.00010 | -4.91 |
| 202864_s_at | SP100 | 0.00135 | -4.37 |
| 232529_at | SP3 | 0.00021 | -5.91 |
| 201996_s_at | SPEN | 0.01748 | -3.31 |
| 201023_at | TAF7 | 0.00019 | -2.57 |
| 226037_s_at | TAF9B | 0.00000 | -6.58 |
| 235890_at | TBL1XR1 | 0.00204 | -2.42 |
| 225544_at | TBX3 | 0.00178 | -3.21 |
| 227705_at | TCEAL7 | 0.00000 | -19.44 |
| 204931_at | TCF21 | 0.00043 | -3.93 |
| 201730_s_at | TPR | 0.00958 | -4.40 |
| 223393_s_at | TSHZ3 | 0.00000 | -5.95 |
| 204771_s_at | TTF1 | 0.01342 | -1.90 |
| 208760_at | UBE2I | 0.00002 | -5.03 |
| 220746_s_at | UIMC1 | 0.00029 | -2.84 |
| 223118_s_at | USP47 | 0.00015 | -4.99 |
| 213425_at | WNT5A | 0.00000 | -7.21 |
| 206067_s_at | WT1 | 0.00146 | -3.43 |
| 208643_s_at | XRCC5 | 0.00082 | -2.37 |

| | | | |
|---|---|---|---|
| 224718_at | YY1 | 0.00076 | -2.75 |
| 212764_at | ZEB1 | 0.00019 | -4.74 |
| 228333_at | ZEB2 | 0.00020 | -2.90 |
| 219778_at | ZFPM2 | 0.00000 | -33.39 |
| 223213_s_at | ZHX1 | 0.00380 | -2.86 |
| 226015_at | ZNF12 | 0.00036 | -4.27 |
| 228545_at | ZNF148 | 0.00001 | -6.64 |
| 203247_s_at | ZNF24 | 0.00002 | -4.38 |
| 218401_s_at | ZNF281 | 0.00033 | -3.48 |
| 225539_at | ZNF295 | 0.00051 | -4.22 |
| 219266_at | ZNF350 | 0.00134 | -2.19 |
