# Supplementary material for: Transcriptional override: a regulatory network model of indirect responses to modulations in microRNA expression
Source: BMC Syst Biol. 2014 Mar 25;8:36. doi: 10.1186/1752-0509-8-36 (PMC3987680; doi:10.1186/1752-0509-8-36)
Supplement: Additional file 4 — List of TRANSFAC [[15]]-identified regulatory targets of 10 repressor genes (see Table 3) that are also the targets of one-or-more of 31 miRNAs upregulated in CEPI (843 genes). [file 1752-0509-8-36-S4.pdf]

Additional file 4

Probeset_ID	Gene-Symbol	P-value	Fold-change	microRNAs	Repressors
1007_s_at	DDR1	0.0549881	0.297337	3	1
1405_i_at	CCL5	0.0530068	-1.719105	1	1
1552644_a_at	PHC3	0.0598531	0.328783	16	1
1552727_s_at	ADAMTS17	0.0006301	-1.124799	3	1
1553101_a_at	ALKBH5	0.0444306	0.34223	2	1
1553217_s_at	ZNF41	0.06359	0.195504	4	1
1553514_a_at	VNN3	0.0840234	0.317879	13	1
1553677_a_at	TIPRL	0.038681	0.520289	7	1
1554114_s_at	SSH2	0.037096	0.315589	19	2
1554248_at	ZNF638	0.0000758	-1.037898	17	1
1554352_s_at	DENND4A	0.0002966	-0.802854	3	2
1554890_a_at	TIA1	0.0033883	0.71951	19	1
1555612_s_at	G6PC	0.0203328	0.757128	10	2
1556203_a_at	SRGAP2	0.0343167	-1.045245	15	2
1556269_at	MYT1	0.0020707	0.615946	4	1
1557122_s_at	GABRB2	0.0056141	-1.08952	4	1
1557674_s_at	EFCAB2	0.0435443	0.375076	4	1
1559064_at	NUP153	0.0000463	0.611535	6	1
1559419_at	CACNB2	0.0002579	-1.859759	11	3
1561651_s_at	TAL1	0.0006895	0.403658	1	2
1562736_at	LHX9	0	-7.248838	13	2
1563533_at	GADL1	0.0000001	-4.902442	5	1
1569030_s_at	NUB1	0.0055588	0.939148	6	1
1569652_at	MLLT3	0.0189736	-1.016633	7	1
1569990_at	NUDT3	0.0166389	0.539776	4	2
200014_s_at	HNRNPC	0.0064343	-0.882451	11	1
200046_at	DAD1	0.0163689	-1.029707	5	1
200059_s_at	RHOA	0.0075581	-1.756405	7	2
200608_s_at	RAD21	0.0233044	-1.078185	5	1
200626_s_at	MATR3	0.0017625	-1.752712	11	1
200662_s_at	TOMM20	0.0087716	-1.158418	12	1
200710_at	ACADVL	0.0521686	0.572218	1	2
200721_s_at	ACTR1A	0.045596	0.392014	6	1
200732_s_at	PTP4A1	0.0000036	-2.775245	20	1
200743_s_at	TPP1	0.0000001	-1.944242	8	2
200745_s_at	GNB1	0.055637	-1.383516	1	1
200758_s_at	NFE2L1	0.0001762	-1.05	8	1

200770_s_at	LAMC1	0.0009695	1.444136	13	1
200788_s_at	PEA15	0.0006107	1.158641	5	1
200815_s_at	PAFAH1B1	0.0019771	1.01795	19	2
200854_at	NCOR1	0.0023295	-0.825878	5	2
200872_at	S100A10	0.0000005	-2.450352	1	3
200880_at	DNAJA1	0.02334	-1.201484	3	1
200912_s_at	EIF4A2	0.0039001	-0.860695	19	1
200916_at	TAGLN2	0.0000874	1.324729	10	3
200919_at	PHC2	0.0209175	0.517703	2	1
200952_s_at	CCND2	0.046266	0.191074	13	1
201055_s_at	HNRNPA0	0.0154005	0.415633	5	1
201091_s_at	CBX3	0.0502767	0.86077	5	1
201153_s_at	MBNL1	0.0077724	-1.028487	13	2
201166_s_at	PUM1	0.0107867	-0.959101	11	1
201176_s_at	ARCN1	0.0039351	-0.904217	17	1
201184_s_at	CHD4	0.0004561	0.585539	5	2
201207_at	TNFAIP1	0.0027508	-0.762638	9	1
201271_s_at	RALY	0.0422732	0.667731	1	1
201312_s_at	SH3BGRL	0.0000331	-1.725855	4	1
201313_at	ENO2	0.0033328	1.329579	3	3
201368_at	ZFP36L2	0.0006934	-1.581127	7	1
201375_s_at	PPP2CB	0.0007792	-1.99623	1	1
201380_at	CRTAP	0.0007949	-1.107551	3	1
201386_s_at	DHX15	0.0003401	-2.237166	8	1
201407_s_at	PPP1CB	0.0427217	0.810864	7	1
201431_s_at	DPYSL3	0.0007917	-1.663272	1	1
201439_at	GBF1	0.0071887	0.429848	8	2
201466_s_at	JUN	0.0358071	1.215739	7	1
201534_s_at	UBL3	0.0000011	-2.248038	8	1
201556_s_at	VAMP2	0.044219	0.391144	3	1
201565_s_at	ID2	0.0125169	-1.01267	5	2
201574_at	ETF1	0.0030488	-1.574004	17	1
201583_s_at	SEC23B	0.0019266	-1.152699	5	1
201669_s_at	MARCKS	0.0079956	-1.776271	13	1
201693_s_at	EGR1	0.0139932	1.308176	7	1
201733_at	CLCN3	0.0252303	-1.134301	5	1
201739_at	SGK1	0.0148015	-1.416594	7	3
201746_at	TP53	0.0424727	0.656228	4	1
201751_at	JOSD1	0.0000039	-1.543616	12	1
201773_at	ADNP	0.0028255	-1.525688	6	2

201794_s_at	SMG7	0.0019682	1.15492	5	1
201800_s_at	OSBP	0.0013978	-0.870333	6	2
201807_at	VPS26A	0.0026892	-1.496955	10	1
201842_s_at	EFEMP1	0	-6.398945	2	1
201844_s_at	RYBP	0.0218756	-1.381224	14	1
201877_s_at	PPP2R5C	0.0001572	-1.194455	8	1
201879_at	ARIH1	0.0279271	-1.006543	7	2
201886_at	DCAF11	0.0166602	0.532776	3	1
201988_s_at	CREBL2	0.0000779	-0.702	11	1
201995_at	EXT1	0.020278	0.585584	2	1
201997_s_at	SPEN	0.0931099	0.460492	8	1
202081_at	IER2	0.014949	0.71365	2	1
202121_s_at	CHMP2A	0.0025423	-1.391456	1	1
202124_s_at	TRAK2	0.0054626	-1.14341	7	3
202130_at	RIOK3	0.002789	-1.157232	8	1
202172_at	VEZF1	0.0198751	-0.935978	7	3
202197_at	MTMR3	0.0006289	-1.163005	7	1
202241_at	TRIB1	0.0879485	0.792996	3	3
202254_at	SIPA1L1	0.000018	-1.251093	2	1
202258_s_at	N4BP2L2	0.0000179	-1.324317	4	1
202267_at	LAMC2	0.0419863	1.058705	7	2
202290_at	PDAP1	0.0768885	0.264699	5	1
202294_at	STAG1	0.000007	-1.325741	11	1
202370_s_at	CBFB	0.0030802	-0.858356	14	1
202381_at	ADAM9	0.0055029	-1.442786	22	1
202388_at	RGS2	0.0016648	-1.892173	6	2
202429_s_at	PPP3CA	0.0057081	-1.93346	10	1
202451_at	GTF2H1	0.0022332	-1.040456	8	1
202468_s_at	CTNNAL1	0.0000016	-3.523364	1	2
202478_at	TRIB2	0.0022577	0.923593	6	2
202512_s_at	ATG5	0.0000061	-2.620507	13	1
202531_at	IRF1	0.0622341	0.518098	9	1
202555_s_at	MYLK	0.0103137	-2.020528	18	1
202568_s_at	MARK3	0.0001886	-1.768459	9	1
202607_at	NDST1	0.0010263	0.581936	2	1
202609_at	EPS8	0.0000011	-2.985403	12	1
202631_s_at	APPBP2	0.004057	-0.879226	10	1
202688_at	TNFSF10	0.0528153	-1.606724	7	1
202723_s_at	FOXO1	0.0000316	-1.232452	5	1
202729_s_at	LTBP1	0.0109984	1.7466	10	1

202731_at	PDCD4	0.0000709	-1.242051	11	2
202770_s_at	CCNG2	0.0060031	-1.270275	8	1
202790_at	CLDN7	0.0019604	0.852085	2	1
202820_at	AHR	0.0020042	-1.189633	16	1
202825_at	SLC25A4	0.000106	-1.554365	8	2
202840_at	TAF15	0.0007252	-0.918415	10	1
202920_at	ANK2	0.0013474	-1.252653	9	1
202960_s_at	MUT	0.0039413	-0.801194	3	1
202964_s_at	RFX5	0.0097579	0.913162	3	2
202998_s_at	LOXL2	0.0000141	1.039316	2	1
203024_s_at	C5orf15	0.0081674	-1.226383	4	1
203035_s_at	PIAS3	0.0364938	0.711248	8	1
203069_at	SV2A	0.0079764	0.475724	8	1
203083_at	THBS2	0.0188116	1.80251	9	1
203117_s_at	PAN2	0.0174286	0.320039	5	1
203131_at	PDGFRA	0.0088368	-2.25246	20	2
203178_at	GATM	0.0016347	-2.270564	12	1
203194_s_at	NUP98	0.0054003	0.237789	13	1
203249_at	EZH1	0.0005361	-0.75004	7	1
203286_at	RNF44	0.0173856	0.712405	8	2
203297_s_at	JARID2	0.0251135	0.664379	17	3
203301_s_at	DMTF1	0.0092661	-1.119912	14	1
203367_at	DUSP14	0.0543891	0.445671	3	1
203373_at	SOCS2	0.0001313	-1.420337	3	1
203378_at	PCF11	0.0065301	-1.076925	7	2
203384_s_at	GOLGA1	0.0000901	-0.739683	15	1
203408_s_at	SATB1	0.000027	-2.747028	14	1
203413_at	NELL2	0	-6.994244	12	1
203429_s_at	C1orf9	0.0001859	-1.121258	20	1
203438_at	STC2	0.0000862	1.687306	6	2
203491_s_at	CEP57	0.0290776	-1.480416	14	1
203497_at	MED1	0.002568	-1.692014	11	1
203525_s_at	APC	0.0012043	-1.668275	12	1
203542_s_at	KLF9	0.0000016	-2.573766	17	1
203556_at	ZHX2	0.0433269	0.701142	9	2
203593_at	CD2AP	0.0314606	-1.435085	8	1
203603_s_at	ZEB2	0.0062582	-1.425736	25	3
203640_at	MBNL2	0.0011979	-1.290369	9	3
203650_at	PROCR	0	-4.44941	2	1
203700_s_at	DIO2	0.0127142	0.558753	3	1

203710_at	ITPR1	0.0000626	-1.684374	20	1
203725_at	GADD45A	0.0388019	-1.055521	4	1
203734_at	FOXJ2	0.0036956	-0.862733	14	1
203797_at	VSNL1	0.0111679	-0.906471	4	2
203810_at	DNAJB4	0.0001022	-2.39187	7	1
203874_s_at	SMARCA1	0.0129792	-1.101049	2	1
203881_s_at	DMD	0.0000022	-3.427764	8	3
203913_s_at	HPGD	0.0000921	-1.886077	5	1
203915_at	CXCL9	0.0099599	-2.161133	4	1
203992_s_at	KDM6A	0.0046746	-1.014192	10	3
204021_s_at	PURA	0.0001071	-0.743521	14	2
204031_s_at	PCBP2	0.0287964	-1.300085	7	1
204055_s_at	CTAGE5	0.0157426	-1.04089	5	1
204073_s_at	C11orf9	0.0005168	-1.151306	3	2
204082_at	PBX3	0.0000035	-2.548493	16	1
204136_at	COL7A1	0.0000207	1.805357	2	2
204176_at	KLHL20	0.0071964	-1.40699	18	1
204194_at	BACH1	0.0005642	-1.867651	7	1
204206_at	MNT	0.0005586	0.834758	3	4
204254_s_at	VDR	0.0028522	1.618324	2	1
204256_at	ELOVL6	0.0013155	2.121157	9	2
204259_at	MMP7	0.0014337	2.503034	12	1
204334_at	KLF7	0.0011293	-1.10692	3	2
204377_s_at	VPRBP	0.0016844	0.641428	4	1
204388_s_at	MAOA	0.0003939	-2.150482	6	1
204393_s_at	ACPP	0.0086241	-2.116468	3	2
204424_s_at	LMO3	0.0039247	1.356545	15	5
204432_at	SOX12	0.0005868	1.163844	3	2
204440_at	CD83	0.0199012	-1.530383	4	1
204491_at	PDE4D	0.0226521	0.390979	17	1
204499_at	AGTPBP1	0.0056095	-0.808088	11	1
204544_at	HPS5	0.0006628	-1.109518	14	1
204589_at	NUAK1	0.0003767	1.79139	4	2
204602_at	DKK1	0.0086067	-1.059223	3	1
204604_at	CDK14	0.0007342	-0.856783	14	1
204641_at	NEK2	0.0000009	3.240147	3	1
204684_at	NPTX1	0.0000631	-1.01103	8	1
204719_at	ABCA8	0	-7.007857	10	1
204783_at	MLF1	0.0034949	-1.300187	7	1
204798_at	MYB	0.0001793	1.324382	13	2

204823_at	NAV3	0	-3.871375	13	1
204831_at	CDK8	0.006364	-0.972824	5	1
204847_at	ZBTB11	0.0032681	-1.275871	9	2
204866_at	PHF16	0.0250931	0.806731	7	1
204872_at	TLE4	0.0000115	-2.992518	14	1
204886_at	PLK4	0.0776164	0.789601	2	1
204944_at	PTPRG	0.0016838	1.758673	11	1
204984_at	GPC4	0.0000183	-1.100214	2	1
204998_s_at	ATF5	0.0001652	0.820493	3	1
205003_at	DOCK4	0.0000058	-2.744086	21	1
205026_at	STAT5B	0.0959995	0.196403	9	1
205070_at	ING3	0.0051991	-2.409646	21	1
205080_at	RARB	0.003962	-0.883173	12	1
205117_at	FGF1	0.0001005	-1.923914	7	1
205176_s_at	ITGB3BP	0.0358793	-1.081048	9	2
205189_s_at	FANCC	0.0204434	0.51774	6	1
205242_at	CXCL13	0.0293612	1.366128	2	1
205259_at	NR3C2	0.0000001	-3.471671	8	2
205267_at	POU2AF1	0.0893755	1.155985	1	1
205288_at	CDC14A	0.0000038	-2.369064	25	1
205290_s_at	BMP2	0.0000954	-2.494889	8	2
205292_s_at	HNRNPA2B1	0.0110437	-1.21112	3	2
205301_s_at	OGG1	0.0413989	0.32033	12	1
205372_at	PLAG1	0.000074	2.543048	15	2
205428_s_at	CALB2	0	-4.116188	10	1
205453_at	HOXB2	0.0070041	1.01787	4	2
205466_s_at	HS3ST1	0.0001097	-1.617209	8	1
205528_s_at	RUNX1T1	0.0031019	-0.91885	5	1
205532_s_at	CDH6	0.0000019	4.857035	4	1
205632_s_at	PIP5K1B	0.0000021	-2.671878	14	1
205656_at	PCDH17	0.000218	-3.288846	3	2
205710_at	LRP2	0.0000066	-0.989988	9	1
205728_at	ODZ1	0.0513236	-1.212458	13	2
205741_s_at	DTNA	0.0000764	-1.781665	14	1
205795_at	NRXN3	0.0002605	-2.26908	10	2
205816_at	ITGB8	0.036766	1.077668	11	2
205836_s_at	YTHDC2	0.0520748	0.435179	8	1
205862_at	GREB1	0.0078259	-2.82845	5	1
205907_s_at	OMD	0.0000004	-4.071845	2	1
205932_s_at	MSX1	0.033488	2.393504	1	1

206117_at	TPM1	0.0180227	1.382081	7	2
206201_s_at	MEOX2	0.0031485	-2.437754	11	1
206245_s_at	IVNS1ABP	0.0276745	-1.009287	9	1
206336_at	CXCL6	0.0036218	-1.857953	10	1
206363_at	MAF	0	-3.226272	6	1
206373_at	ZIC1	0.0008602	4.561977	7	2
206375_s_at	HSPB3	0.000003	-2.460988	1	1
206415_at	TLL1	0.000048	-1.313726	8	1
206508_at	CD70	0.0719021	0.284148	3	1
206542_s_at	SMARCA2	0.0000466	-3.136173	11	2
206601_s_at	HOXD3	0.0000348	-2.705747	1	1
206698_at	XK	0.0272642	1.393309	8	1
206765_at	KCNJ2	0.0000022	-2.353415	11	1
206854_s_at	MAP3K7	0.0566626	-1.229237	11	1
206858_s_at	HOXC6	0.0000007	-2.516193	3	1
206949_s_at	RUSC1	0.000017	1.725964	3	1
207010_at	GABRB1	0.0002279	-1.103294	9	1
207264_at	KDELR3	0.0055509	0.637615	6	1
207309_at	NOS1	0.0005797	0.467364	1	1
207435_s_at	SRRM2	0.0047064	0.792306	5	1
207480_s_at	MEIS2	0	-4.512211	8	1
207785_s_at	RBPJ	0.0000735	-1.805595	5	1
207788_s_at	SORBS3	0.0792509	0.643164	4	1
207845_s_at	ANAPC10	0.0055946	-1.452617	2	2
207922_s_at	MAEA	0.0007307	-0.740588	1	1
207983_s_at	STAG2	0.0012539	-0.75524	15	1
208021_s_at	RFC1	0.0000738	-1.506256	3	1
208066_s_at	GTF2B	0.0007373	-1.631062	2	3
208264_s_at	EIF3J	0.0002849	-1.32876	1	2
208625_s_at	EIF4G1	0.0359401	0.687202	1	3
208632_at	RNF10	0.0001911	-1.308139	2	1
208636_at	ACTN1	0.000011	1.580712	6	2
208650_s_at	CD24	0.0000054	6.168816	3	1
208656_s_at	CCNI	0.0000009	-3.176909	1	1
208666_s_at	ST13	0.0123473	-1.709144	9	1
208679_s_at	ARPC2	0.000228	-2.995241	1	1
208712_at	CCND1	0.0000322	2.916517	8	1
208732_at	RAB2A	0.0033941	-1.222365	3	1
208760_at	UBE2I	0.0000167	-2.330648	6	1
208776_at	PSMD11	0.000012	-0.906958	10	2

208835_s_at	LUC7L3	0.0047352	-2.128058	9	5
208841_s_at	G3BP2	0.0093038	-1.767009	10	1
208869_s_at	GABARAPL1	0.0000003	-1.778305	9	2
208886_at	H1F0	0.0439952	1.35167	2	1
208925_at	CLDND1	0.001786	-1.035245	16	1
208989_s_at	KDM2A	0.0010964	0.582311	15	2
208991_at	STAT3	0.0032482	-1.803919	10	1
208993_s_at	PPIG	0.0064909	-1.951293	1	1
209030_s_at	CADM1	0.0050675	-1.092357	9	3
209034_at	PNRC1	0.0000062	-2.284992	5	1
209046_s_at	GABARAPL2	0.0000103	-1.984642	4	1
209047_at	AQP1	0.0001974	-0.965124	5	2
209048_s_at	ZMYND8	0.0236518	0.721138	7	3
209069_s_at	H3F3B	0.0042544	-1.076433	11	2
209075_s_at	ISCU	0.0001156	-1.261271	1	2
209102_s_at	HBP1	0.0000165	-1.827106	20	1
209112_at	CDKN1B	0.0005906	-2.136418	7	1
209127_s_at	SART3	0.0414016	-1.236089	3	2
209148_at	RXRB	0.0365686	0.436259	3	2
209170_s_at	GPM6B	0.0034399	1.758726	4	1
209186_at	ATP2A2	0.0339255	0.859145	15	1
209190_s_at	DIAPH1	0.000549	0.83004	1	2
209198_s_at	SYT11	0.0000264	1.89363	3	1
209199_s_at	MEF2C	0.0000013	-2.125127	7	2
209234_at	KIF1B	0.010774	-1.074343	17	1
209242_at	PEG3	0.0069146	-2.652082	5	2
209271_at	BPTF	0.0040501	-1.134744	16	1
209280_at	MRC2	0.0897347	0.495913	6	1
209288_s_at	CDC42EP3	0.000646	-1.690128	3	2
209291_at	ID4	0.0144063	-1.193243	9	1
209300_s_at	NECAP1	0.0041111	-1.28782	10	1
209308_s_at	BNIP2	0.0005982	-1.598726	9	1
209323_at	PRKRIR	0.0289055	-1.099505	4	1
209358_at	TAF11	0.0335614	0.504356	2	1
209360_s_at	RUNX1	0.0114138	1.140259	12	2
209379_s_at	FAM190B	0.0000016	-2.049255	15	2
209430_at	BTAF1	0.0005735	-1.215807	14	1
209431_s_at	PATZ1	0.0678678	0.441604	11	1
209435_s_at	ARHGEF2	0.00114	1.297868	3	1
209451_at	TANK	0.0137423	-1.500653	8	1

209455_at	FBXW11	0.0011928	-1.119136	16	2
209518_at	SMARCD1	0.0023366	0.899803	12	2
209549_s_at	DGUOK	0.0307426	0.468311	2	1
209568_s_at	RGL1	0.0000215	-2.166938	18	1
209576_at	GNAI1	0.0000717	-1.906677	14	1
209589_s_at	EPHB2	0.0003538	0.542553	5	2
209639_s_at	RGS12	0.0002647	0.579519	5	1
209672_s_at	MIOS	0.0044877	-0.981699	10	1
209684_at	RIN2	0.0127819	0.629392	9	1
209797_at	CNPY2	0.0012787	1.267193	2	1
209824_s_at	ARNTL	0.0006347	-0.81428	6	1
209849_s_at	RAD51C	0.0000799	-1.836386	3	1
209894_at	LEPR	0.0059723	-1.95817	9	1
210064_s_at	UPK1B	0.0105982	-1.272685	10	1
210135_s_at	SHOX2	0.0031727	-1.044281	5	2
210200_at	WWP2	0.0499298	0.213752	15	1
210251_s_at	RUFY3	0.0151381	-0.909447	9	1
210275_s_at	ZFAND5	0.0164906	-1.142436	4	3
210277_at	AP4S1	0.0085761	-1.205824	13	1
210312_s_at	IFT20	0.0018797	-1.135987	1	1
210338_s_at	HSPA8	0.0002257	-2.433485	7	1
210347_s_at	BCL11A	0.0056808	1.733511	17	4
210355_at	PTHLH	0.0003012	-1.767662	20	1
210407_at	PPM1A	0.024014	-0.968773	13	1
210466_s_at	SERBP1	0.0913568	0.629791	2	1
210512_s_at	VEGFA	0.0000542	2.469018	11	1
210538_s_at	BIRC3	0.0016514	-1.440266	1	1
210561_s_at	WSB1	0.0010683	-2.068929	12	1
210759_s_at	PSMA1	0.0000875	-2.296006	4	3
210762_s_at	DLC1	0.0011288	-1.467089	19	1
210786_s_at	FLI1	0.0303937	0.169044	12	1
210896_s_at	ASPH	0.0043135	-1.122069	19	1
211033_s_at	PEX7	0.0276703	-1.019993	5	1
211071_s_at	MLLT11	0.0223126	1.016265	5	1
211081_s_at	MAP4K5	0.011472	-1.306904	11	2
211275_s_at	GYG1	0.0002095	-1.126213	3	1
211341_at	POU4F1	0.060698	0.325352	4	1
211343_s_at	COL13A1	0.0001541	3.093923	9	1
211491_at	ADRA1A	0.0063194	0.554808	1	3
211509_s_at	RTN4	0.0227361	-1.040578	8	2

211600_at	PTPRO	0.0006889	-1.186209	7	1
211685_s_at	NCALD	0.0109431	1.004698	5	1
211755_s_at	ATP5F1	0.0056072	-1.646122	6	1
211905_s_at	ITGB4	0.0603922	0.474673	1	3
211956_s_at	EIF1	0.0003274	-1.080457	2	1
211959_at	IGFBP5	0.0719837	0.872833	3	1
211963_s_at	ARPC5	0.0000214	-2.754047	11	1
212077_at	CALD1	0.0001664	-1.247861	22	1
212111_at	STX12	0.0000287	-1.710368	11	1
212115_at	HN1L	0.0019476	0.739908	1	1
212160_at	XPOT	0.0089736	1.238496	2	1
212219_at	PSME4	0.0163827	0.756756	11	2
212238_at	ASXL1	0.0074618	0.487706	6	1
212349_at	POFUT1	0.0134812	0.781901	11	2
212369_at	ZNF384	0.0011509	0.773522	4	1
212425_at	SCAMP1	0.0012528	-0.79477	3	1
212458_at	SPRED2	0.0184837	-0.972829	2	1
212461_at	AZIN1	0.0398196	-1.284265	9	1
212468_at	SPAG9	0.0004731	-1.850888	13	1
212481_s_at	TPM4	0.0491464	0.883529	3	1
212500_at	ADO	0.0000689	-2.403081	4	1
212515_s_at	DDX3X	0.0022368	-0.814566	16	2
212553_at	RPRD2	0.0003176	1.058508	9	1
212586_at	CAST	0	-3.589859	15	1
212609_s_at	AKT3	0.0000392	-2.090366	9	2
212628_at	PKN2	0.0055333	-1.79963	13	1
212650_at	EHBP1	0.0377094	-1.293609	7	1
212667_at	SPARC	0.0154857	-1.402993	13	1
212681_at	EPB41L3	0.0029683	-0.955015	11	1
212727_at	DLG3	0.0031068	0.740581	6	1
212747_at	ANKS1A	0.0020652	0.51126	5	1
212769_at	TLE3	0.0017759	1.21887	1	2
212774_at	ZNF238	0.0089111	-1.201425	21	3
212798_s_at	ANKMY2	0.0000002	-2.620805	5	1
212837_at	FAM175B	0.0000946	-1.53337	6	1
212907_at	SLC30A1	0.0018956	-2.42332	3	1
212973_at	RPIA	0.0230789	0.752403	4	1
212984_at	ATF2	0.0071592	-0.934847	8	1
213012_at	NEDD4	0.0000802	-0.791018	9	3
213033_s_at	NFIB	0.0003035	-1.095186	11	3

213034_at	SIK3	0.0000095	-1.904082	15	1
213183_s_at	CDKN1C	0.0819069	0.701249	4	1
213194_at	ROBO1	0.0000023	-2.70008	17	1
213197_at	ASTN1	0.0067307	-0.959903	12	1
213226_at	CCNA2	0.0022775	1.518012	9	1
213251_at	SMARCA5	0.0010367	-1.005363	8	1
213280_at	RAP1GAP2	0.0018962	1.581495	1	1
213283_s_at	SALL2	0.0094546	-0.98454	4	2
213302_at	PFAS	0.0240988	1.027159	4	1
213364_s_at	SNX1	0.0000011	-2.006294	8	1
213440_at	RAB1A	0.0123173	-0.941083	20	1
213446_s_at	IQGAP1	0.0378892	-1.43333	6	1
213518_at	PRKCI	0.013525	0.853929	6	1
213655_at	YWHAE	0.0051149	-2.024639	5	1
213849_s_at	PPP2R2B	0.0032071	-1.604321	3	1
213872_at	C6orf62	0.0007595	-2.33371	14	1
213891_s_at	TCF4	0.0019704	-1.052089	14	1
213934_s_at	ZNF23	0.0063805	-0.925895	8	2
213960_at	NTRK3	0.0170309	0.593508	11	2
213988_s_at	SAT1	0.0640051	-1.236541	1	1
213998_s_at	DDX17	0.001086	-3.278542	3	3
214102_at	ARAP2	0.0024474	-1.457143	16	3
214155_s_at	LARP4	0.0211171	1.016472	4	1
214157_at	GNAS	0.0012883	2.103057	11	3
214158_s_at	PRDM10	0.0621307	0.421801	10	1
214168_s_at	TJP1	0.0563295	0.485122	7	1
214276_at	KLF12	0.0162518	0.459839	8	2
214319_at	FRY	0.0000101	-2.291877	10	1
214582_at	PDE3B	0.00067	-0.834914	13	2
214709_s_at	KTN1	0.0000037	-1.853172	11	1
214724_at	DIXDC1	0.0000013	-2.670564	11	1
214743_at	CUX1	0.0126219	-0.88974	6	1
214761_at	ZNF423	0.0015775	2.149831	3	2
214930_at	SLITRK5	0.0155265	-1.560192	7	2
215082_at	ELOVL5	0.011721	0.409397	11	1
215220_s_at	TPR	0.0273159	-1.050033	5	1
215269_at	TRAPPC10	0.0038508	-0.88353	9	1
215478_at	RIMS2	0.0006598	0.49698	10	2
215501_s_at	DUSP10	0.0194479	0.441437	10	1
215726_s_at	CYB5A	0.0167119	-1.759142	2	1

215884_s_at	UBQLN2	0.0009859	-1.121483	11	1
215903_s_at	MAST2	0.0000448	1.479748	6	1
215972_at	PART1	0.0056319	1.152814	13	1
216115_at	NF1	0.0022088	-0.767144	13	1
216199_s_at	MAP3K4	0.0008329	-1.044786	11	1
216221_s_at	PUM2	0.0001205	-1.807302	5	1
216268_s_at	JAG1	0.0047276	1.497156	7	1
216321_s_at	NR3C1	0.0002911	-2.426625	13	1
216347_s_at	PPP1R13B	0.0067426	0.946388	2	1
216520_s_at	TPT1	0.0000009	-1.795588	2	1
216804_s_at	PDLIM5	0.0017976	-0.990548	14	1
216993_s_at	COL11A2	0.0251092	0.562244	2	1
217097_s_at	PHTF2	0.0605446	0.21233	23	1
217168_s_at	HERPUD1	0.0000524	-1.759189	3	1
217738_at	NAMPT	0.0000735	-2.748348	13	1
217783_s_at	YPEL5	0.0003959	-0.929293	7	3
217809_at	BZW2	0.0001031	1.769883	15	1
217811_at	SELT	0.0160807	-0.960805	5	1
217834_s_at	SYNCRIP	0.0720323	1.003107	14	1
217873_at	CAB39	0.0000283	-2.182613	8	2
217955_at	BCL2L13	0.0004766	-2.043662	3	1
217966_s_at	FAM129A	0.0159289	-1.388517	12	1
217979_at	TSPAN13	0.0000019	-2.315995	6	1
218048_at	COMMD3	0.0009266	-1.007753	14	1
218079_s_at	GGNBP2	0.0040794	-1.152628	9	1
218111_s_at	CMAS	0.0016888	-1.598996	3	1
218173_s_at	WHSC1L1	0.0079271	-0.939037	9	2
218191_s_at	LMBRD1	0.0000045	-3.100334	9	1
218224_at	PNMA1	0.0013285	-1.573098	7	2
218230_at	ARFIP1	0.010634	-1.093081	7	1
218243_at	RUFY1	0.0001324	-0.893528	2	1
218263_s_at	ZBED5	0.0003518	-2.080013	2	1
218361_at	GOLPH3L	0.0007282	-0.712586	7	1
218368_s_at	TNFRSF12A	0.0137591	0.474066	2	2
218370_s_at	S100PBP	0.0007545	-1.664602	16	1
218401_s_at	ZNF281	0.0003251	-1.800771	9	1
218411_s_at	MBIP	0.001338	-1.763717	8	1
218422_s_at	RBM26	0.005157	-1.404346	14	1
218486_at	KLF11	0.0004294	-1.24283	13	1
218532_s_at	FAM134B	0	-3.051238	4	2

218534_s_at	AGGF1	0.0000078	-1.603855	13	1
218583_s_at	DCUN1D1	0.0391211	0.610459	7	1
218611_at	IER5	0.0008737	-1.11909	3	1
218645_at	ZNF277	0.0000704	-2.721998	5	1
218669_at	RAP2C	0.0000656	-1.657312	18	2
218694_at	ARMCX1	0.0000048	-2.315335	11	1
218730_s_at	OGN	0.0000009	-4.391083	10	1
218761_at	RNF111	0.0030822	-1.070065	9	1
218776_s_at	TMEM62	0.0290329	0.441074	2	1
218808_at	DALRD3	0.0113902	0.806463	1	1
218880_at	FOSL2	0.0015232	1.158436	3	1
218974_at	SOBP	0.0000533	-2.172871	15	4
219027_s_at	MYO9A	0.0000012	-0.999146	17	1
219033_at	PARP8	0.0007971	-1.173448	11	1
219035_s_at	RNF34	0.0048938	-0.909581	1	1
219250_s_at	FLRT3	0.0000062	-2.527238	7	1
219263_at	RNF128	0.0000011	-3.883979	8	1
219281_at	MSRA	0.0001476	-1.391793	2	1
219292_at	THAP1	0.0002318	-1.726603	5	1
219421_at	TTC33	0.0004388	-1.572073	5	1
219502_at	NEIL3	0.00411	0.633018	3	1
219511_s_at	SNCAIP	0.0000017	-2.462377	8	1
219622_at	RAB20	0.0306813	0.880332	1	1
219644_at	CCDC41	0.0017402	-1.824043	7	1
219695_at	SMPD3	0.0005537	-0.876687	3	1
219738_s_at	PCDH9	0.0000001	-2.836894	11	1
219778_at	ZFPM2	0	-5.061526	20	3
219787_s_at	ECT2	0.0000288	3.166731	4	2
219961_s_at	PLK1S1	0.000032	-2.242828	7	2
220054_at	IL23A	0.0217716	0.333901	1	1
220330_s_at	SAMSN1	0.069617	-1.431716	2	1
220446_s_at	CHST4	0.0000195	-0.718584	3	1
220595_at	PDZRN4	0.013902	-0.955102	5	4
220983_s_at	SPRY4	0.0613275	0.354765	2	2
221082_s_at	NDRG3	0.0153384	0.724478	3	1
221479_s_at	BNIP3L	0.0068297	-1.433659	12	1
221502_at	KPNA3	0.0011928	-1.911577	14	1
221569_at	AHI1	0.0037348	-1.404204	7	1
221584_s_at	KCNMA1	0.0082924	1.37809	13	2
221654_s_at	USP3	0.0027005	-1.102603	12	1

221656_s_at	ARHGEF10L	0.0006288	0.576368	3	1
221667_s_at	HSPB8	0.0020223	-0.736417	7	2
221718_s_at	AKAP13	0.0096332	-1.316525	4	1
221756_at	PIK3IP1	0	-3.065119	5	1
221832_s_at	LUZP1	0.018027	-1.008515	1	1
221921_s_at	CADM3	0.0840014	0.648039	1	1
222018_at	NACA	0.0020562	1.631494	2	1
222062_at	IL27RA	0.0123296	0.689516	1	1
222103_at	ATF1	0.0019256	-1.154731	4	1
222119_s_at	FBXO11	0.000685	-1.594091	16	2
222244_s_at	TUG1	0.0000003	-2.545626	2	2
222444_at	ARMCX3	0.0122239	-0.867243	3	2
222445_at	SLC39A9	0.0026287	-0.860803	17	1
222513_s_at	SORBS1	0.0001528	1.267206	4	1
222517_at	AP3M1	0.0085423	-1.10273	5	2
222525_s_at	CCDC25	0.001516	-0.869879	4	1
222537_s_at	CDC42SE1	0.0001804	1.005325	15	1
222538_s_at	APPL1	0.0090456	-1.03453	14	1
222559_s_at	RPRD1A	0.0004446	-1.771021	11	1
222574_s_at	DHX40	0.0000437	-1.572632	9	1
222594_s_at	SPATS2	0.0101858	0.381799	16	1
222600_s_at	UBA6	0.0612609	-1.273002	12	1
222667_s_at	ASH1L	0.0084965	-2.141786	7	2
222684_s_at	NOL10	0.0004623	1.075691	3	2
222701_s_at	CHCHD7	0.0012349	1.887799	6	2
222747_s_at	SCML1	0.0032697	-1.375091	8	1
222811_at	FTSJD1	0.0005606	-2.03954	13	1
222975_s_at	CSDE1	0.0002723	-1.453609	12	1
222981_s_at	RAB10	0.0685522	0.727531	16	2
222989_s_at	UBQLN1	0.0056954	0.747621	11	1
223017_at	TXNDC12	0.0037262	-1.313629	7	1
223059_s_at	FAM107B	0.0351952	-1.596451	8	2
223068_at	EML4	0.000186	-2.143456	7	1
223075_s_at	AIF1L	0.0000001	4.265611	4	1
223089_at	VEZT	0.0332179	-1.301074	15	1
223119_s_at	USP47	0.0000118	-1.485862	16	1
223132_s_at	TRIM8	0.0002805	1.381066	11	3
223139_s_at	DHX36	0.0020858	-2.722292	6	1
223144_s_at	AKIRIN2	0.0580369	0.430957	5	2
223154_at	MRPL1	0.0085213	-1.406831	1	1

223156_at	MRPS23	0.0071217	-1.068931	2	1
223159_s_at	NEK6	0.0152948	0.659887	6	1
223165_s_at	IP6K2	0.0010518	1.355565	3	2
223190_s_at	MLL5	0.0182896	-1.337814	9	1
223197_s_at	SMARCAD1	0.000591	-1.325725	5	2
223232_s_at	CGN	0.0010457	-1.248013	3	1
223261_at	POLK	0.0000029	-2.560077	8	1
223264_at	MESDC1	0.0023014	0.916435	1	1
223266_at	STRADB	0	-1.939335	7	3
223295_s_at	LUC7L	0.0063515	1.098107	3	2
223315_at	NTN4	0.0006594	-3.409443	8	2
223319_at	GPHN	0.0001729	-1.226097	1	1
223392_s_at	TSHZ3	0.0002208	-1.174476	16	1
223465_at	COL4A3BP	0.0404749	-1.305618	7	1
223467_at	RASD1	0.0568899	0.823978	9	1
223503_at	TMEM163	0.0002028	-1.689328	1	1
223504_at	DNAJC27	0.0049938	-1.181158	9	1
223508_at	NOTCH1	0.043719	0.923304	6	1
223654_s_at	CELF4	0.0053924	0.582048	9	2
223705_s_at	GPBP1	0.0021552	-1.268191	19	4
223775_at	HHIP	0.0015043	-0.840223	7	1
223784_at	TMEM27	0.0034167	-1.060812	4	1
223802_s_at	RBBP6	0.0000308	-1.611819	19	1
224164_at	TPM3	0.0020435	0.396675	8	1
224325_at	FZD8	0.0001078	-1.439694	10	1
224413_s_at	TM2D2	0.0000266	-1.790616	15	1
224480_s_at	AGPAT9	0.0005235	-0.837581	3	1
224560_at	TIMP2	0.0047251	-1.730547	11	1
224600_at	CGGBP1	0.0036744	-1.10983	8	1
224636_at	ZFP91	0.0000573	-1.612673	13	1
224657_at	ERRFI1	0.0445338	-1.355273	12	1
224710_at	RAB34	0.0116656	-0.965075	7	2
224755_at	TM9SF3	0.0000002	-2.650144	7	1
224786_at	SCOC	0.0019657	-2.320724	8	1
224797_at	ARRDC3	0.0061236	-1.61083	13	1
224812_at	HIBADH	0.0000937	-1.702617	9	1
224813_at	WASL	0.0000774	-1.867786	15	1
224828_at	CPEB4	0.0163755	-1.287478	9	1
224836_at	TP53INP2	0.0148729	0.392005	4	1
224889_at	FOXO3	0.003898	-1.027091	5	1

224914_s_at	SARNP	0.001377	-1.84021	9	1
224933_s_at	JMJD1C	0.016659	-1.582036	11	1
224938_at	NUFIP2	0.0028382	-1.092406	1	1
224973_at	FAM46A	0.0008355	-1.403957	9	1
224985_at	NRAS	0.0520517	0.579011	6	5
224994_at	CAMK2D	0.0006406	-0.954348	7	1
225037_at	SLC35C2	0.0016361	1.266746	7	1
225126_at	MRRF	0.0029496	0.472207	5	1
225159_s_at	ELK4	0.0024166	-1.072084	1	1
225183_at	C16orf72	0.0065864	-0.872276	14	1
225189_s_at	RAPH1	0.0179609	-1.166945	4	1
225200_at	DPH3	0.0003232	-2.14357	4	1
225207_at	PDK4	0.0000044	-2.784678	7	1
225218_at	ZFYVE27	0.0054707	0.643084	1	1
225223_at	SMAD5	0.0000484	-1.493819	10	3
225236_at	RBM18	0.0010463	-1.058397	4	1
225269_s_at	RBMS1	0.0039835	-0.813226	18	3
225276_at	GSPT1	0.0000588	-2.212759	5	1
225278_at	PRKAB2	0.0011986	-1.308841	8	1
225298_at	PNKD	0.0160952	0.961967	12	1
225312_at	COMMD6	0.0000467	-0.951786	12	1
225378_at	VPS37A	0.0054118	-1.334771	11	2
225417_at	EPC1	0.0029483	-0.800489	1	1
225474_at	MAGI1	0.0061602	-0.806323	10	1
225497_at	ATE1	0.0081422	-1.226941	2	1
225534_at	C8orf40	0.0014823	-2.637115	1	1
225540_at	MAP2	0.0017178	-1.680788	15	1
225557_at	CSRNP1	0.0002937	1.105056	1	2
225572_at	CREB1	0.0015629	-0.985594	5	2
225645_at	EHF	0.00002	5.175659	7	1
225665_at	ZAK	0.0085724	-1.175151	14	1
225681_at	CTHRC1	0.0004672	4.09337	9	1
225710_at	GNB4	0.0000008	-3.456361	12	2
225733_at	B3GALT6	0.0581552	0.512165	3	1
225764_at	ETV6	0.0023822	0.856753	3	2
225771_at	AP1G1	0.0016999	-0.842269	8	1
225820_at	PHF17	0.0035399	-1.161815	16	1
225835_at	SLC12A2	0.0013724	-1.380123	8	1
225840_at	TEF	0.0004076	-1.096072	1	1
225852_at	ANKRD17	0.0207357	-0.929303	4	2

225859_at	XIAP	0.0107166	-0.936881	3	1
225911_at	NPNT	0.0001126	-2.264037	9	1
225956_at	C5orf41	0.0004952	-1.833687	16	1
225968_at	PRICKLE2	0.0033979	-0.906448	6	1
225975_at	PCDH18	0.0009628	-1.063354	6	1
225982_at	UBTF	0.0290949	0.451252	7	1
226030_at	ACADSB	0.0001122	-1.52833	11	1
226066_at	MITF	0.0061429	-0.994025	7	1
226115_at	AHCTF1	0.0274224	-1.021826	14	1
226117_at	TIFA	0.0004086	-2.197144	8	1
226143_at	RAI1	0.0636158	0.368538	4	3
226144_at	REXO1	0.0192721	0.513454	1	1
226178_at	SOCS4	0.0009186	-1.588932	4	1
226185_at	CDS1	0.0019838	-1.757464	9	1
226259_at	EXOC6	0.0015161	-1.08876	11	2
226298_at	RUNDC1	0.0021058	-0.947992	8	1
226335_at	RPS6KA3	0.0108009	-1.118684	7	1
226388_at	TCEA3	0.0000394	-2.529053	1	1
226400_at	CDC42	0.0057504	-1.285004	9	1
226420_at	MECOM	0.0000001	4.214084	7	1
226421_at	AMMECR1	0.0079184	-1.449243	10	1
226439_s_at	NBEA	0.0000011	-1.642732	12	1
226449_at	CEP120	0.0000591	-1.408621	15	1
226461_at	HOXB9	0.0084016	0.475986	2	1
226473_at	CBX2	0.0000041	1.349561	8	1
226497_s_at	FLT1	0.000143	1.456598	18	1
226501_at	XPNPEP3	0.0162639	-1.349896	7	1
226512_at	ZMYM2	0.0005281	-0.96977	9	1
226534_at	KITLG	0	-4.741384	9	1
226552_at	IER5L	0.0081294	0.986651	7	1
226567_at	USP14	0.0023269	-0.80175	8	2
226615_at	XPR1	0.0371419	1.207542	11	1
226633_at	RAB8B	0.0000264	-2.428862	18	1
226665_at	AHSA2	0.0554803	0.419031	3	1
226676_at	ZNF521	0.0001804	-1.675469	6	2
226680_at	IKZF5	0.0000348	-2.097639	5	1
226695_at	PRRX1	0.000025	-3.125098	11	2
226748_at	LYSMD2	0.0000352	-3.046896	2	1
226780_s_at	C7orf55	0.0584008	0.800806	1	1
226816_s_at	KIAA1143	0.0067223	-0.881978	2	1

226820_at	ZNF362	0.0055324	0.933893	12	4
226853_at	BMP2K	0.0968421	0.682508	8	1
226864_at	PKIA	0.0025061	-0.907288	12	1
226932_at	SSPN	0.0064273	-1.431896	3	1
226965_at	FAM116A	0.0001659	-1.713515	12	1
226982_at	ELL2	0.0001402	-1.246412	13	1
227028_s_at	DGCR2	0.000881	0.693036	3	1
227031_at	SNX13	0.0000003	-3.785845	20	1
227083_at	B3GALTL	0.0003586	-1.177895	7	1
227090_at	PHF21A	0.0287392	0.385014	2	1
227095_at	LEPROT	0.000199	-1.660776	9	1
227101_at	ZNF800	0.0441719	-1.358607	21	1
227187_at	CBLL1	0.0077488	-0.983117	4	1
227198_at	AFF3	0.0005441	-1.3131	5	1
227209_at	CNTN1	0.0123292	-1.728537	4	1
227357_at	TAB3	0.0023158	-1.827019	10	1
227425_at	REPS2	0.0001005	-1.503472	8	1
227461_at	STON2	0	4.23286	3	2
227484_at	SRGAP1	0.0357128	-1.634747	10	1
227526_at	CDON	0.0000001	-1.686518	6	1
227530_at	AKAP12	0.077442	-1.349498	2	1
227539_at	GNA13	0.0002577	-1.449789	5	1
227561_at	DDR2	0.0000043	-1.864387	6	1
227621_at	WTAP	0.0250456	-1.26699	11	1
227624_at	TET2	0.0003554	-1.863616	21	1
227646_at	EBF1	0.0692847	1.044049	6	1
227688_at	LRCH2	0.0001156	-1.539117	9	2
227701_at	C10orf118	0.0000531	-1.642204	11	1
227708_at	EEF1A1	0.0000011	-4.141182	6	3
227798_at	SMAD1	0.0056396	0.860237	9	2
227830_at	GABRB3	0.0007909	-1.233123	9	1
227856_at	C4orf32	0.0000473	-2.52847	7	1
227871_at	CHM	0.0211938	-1.504089	8	3
228063_s_at	NAP1L5	0.0002357	-2.367533	5	1
228067_at	C2orf55	0.0055942	-1.005164	3	2
228070_at	PPP2R5E	0.0013747	-1.199514	23	1
228153_at	RNF144B	0.0001025	2.17414	3	2
228260_at	ELAVL2	0.0000559	-1.622538	20	2
228286_at	GEN1	0.0001177	1.15901	8	1
228323_at	CASC5	0.0006296	1.509335	4	1

228347_at	SIX1	0.0196713	3.212509	9	2
228367_at	ALPK2	0.0981477	0.40052	3	1
228415_at	AP1S2	0.0002903	2.167771	8	1
228416_at	ACVR2A	0.0109949	-1.03985	11	2
228454_at	LCOR	0.0043441	-1.400823	7	1
228486_at	SLC44A1	0.0220228	-1.653811	8	1
228570_at	BTBD11	0.0034008	0.561377	9	1
228621_at	HFE2	0.0057626	0.580662	1	2
228636_at	BHLHE22	0.0945837	0.30835	6	1
228745_at	SGTB	0.0003879	-1.971449	10	1
228806_at	RORC	0.0006957	-1.584458	8	1
228834_at	TOB1	0.0004217	-1.573365	8	1
228859_at	C4orf21	0.0649122	0.338858	1	1
228904_at	HOXB3	0.014381	1.950668	5	4
228960_at	NARG2	0.0000253	-1.301474	14	1
228973_at	DLG2	0.0000005	-1.903638	12	3
228999_at	CHD2	0.0867293	0.641966	23	3
229019_at	ZNF385B	0.0000682	-1.895313	6	1
229022_at	ZFX	0.0003953	-1.590367	5	1
229176_at	ANKH	0.000829	-0.949829	4	1
229282_at	GATA6	0.0167176	0.536627	13	1
229325_at	ZZZ3	0.0125123	-0.86846	13	1
229400_at	HOXD10	0.0018295	-2.334015	3	1
229419_at	FBXW7	0.0027575	-1.013267	15	1
229537_at	LMO4	0.0001476	-0.926143	5	2
229665_at	CSTF3	0.0005924	1.538174	5	1
229667_s_at	HOXB8	0.0092193	1.66795	1	2
229765_at	ZNF207	0.010243	-1.927062	8	1
229796_at	SIX4	0.0037053	1.534514	12	1
229800_at	DCLK1	0.0060921	-1.134043	6	2
229844_at	FOXP1	0.0000091	-1.038788	14	3
229885_at	RSF1	0.0094698	-1.015317	3	2
229942_at	BNC2	0.0001524	-1.249304	2	3
230087_at	PRIMA1	0.0000047	2.607346	1	1
230141_at	ARID4A	0.0003412	-2.099554	17	2
230204_at	HAPLN1	0.000566	1.50798	4	1
230237_at	ADCYAP1	0.0000254	-2.613969	6	1
230253_at	SCUBE3	0.0348335	0.460301	5	2
230270_at	PRPF38B	0.0158299	-1.930273	2	1
230288_at	FGF14	0.0003229	-0.701867	4	1

230372_at	HAS2	0.0189021	0.319486	18	3
230425_at	EPHB1	0.0171276	-1.32058	14	1
230561_s_at	C2orf67	0.0029754	-1.315937	15	1
230626_at	TSPAN12	0.000573	2.780954	7	1
230644_at	LRFN5	0.0000437	-0.9031	12	1
230871_at	DHX30	0.0279255	-1.02773	1	1
230887_at	CDC14B	0.0111418	0.282405	8	1
230933_at	DSTN	0.0000142	-1.315745	1	1
230954_at	C20orf112	0.0017446	1.541751	6	2
231065_at	PDE6D	0.0354759	0.313927	3	1
231407_s_at	FOXH1	0.000514	0.729923	2	1
231442_at	ZPBP2	0.0816197	0.216388	2	1
231902_at	ZNF827	0.0130906	0.439663	1	2
231945_at	FILIP1	0.0055214	1.770284	6	1
231961_at	RBPMS	0.0004124	-0.727796	13	1
231986_at	RIMS1	0.0000058	-0.747029	14	1
232037_at	IGDCC3	0.0004296	0.45722	3	1
232087_at	CXorf23	0.0030383	-1.035626	10	1
232184_at	ALS2	0.0092015	-0.954875	13	1
232195_at	GPR158	0.0281594	1.379823	18	1
232270_at	C9orf3	0.0025041	-1.039121	14	1
232291_at	MIR17HG	0.0448616	0.819071	4	1
232382_s_at	PCMTD1	0.0227513	-1.164931	15	1
232388_at	CNTNAP4	0.0000016	-3.845413	3	1
232481_s_at	SLITRK6	0.0033869	-0.880853	1	1
232865_at	AFF4	0.0116401	-1.553822	10	1
233019_at	CNOT7	0.0021427	-1.692222	17	2
233025_at	PDZD2	0.0093072	-1.012843	22	1
233337_s_at	SEZ6L2	0.0414658	0.385418	2	2
233496_s_at	CFL2	0.0002298	-1.409374	21	2
233849_s_at	ARHGAP5	0.0108526	-1.268625	7	1
233952_s_at	ZNF295	0.0113212	-1.330338	14	1
234103_at	KCNT2	0.0000192	-3.886109	15	3
234734_s_at	TNRC6A	0.0036453	-0.804465	20	4
234982_at	UBR3	0.0006058	-1.940733	13	1
235131_at	RHOJ	0.0186629	0.411799	2	1
235308_at	ZBTB20	0.0220275	-1.267598	1	1
235421_at	MAP3K8	0.0031605	-0.850218	13	1
235521_at	HOXA3	0.0832156	1.073402	8	2
235688_s_at	TRAF4	0.0059493	0.543576	8	2

236034_at	ANGPT2	0.0011749	0.577847	10	1
236232_at	STX4	0.02665	0.367813	1	1
236302_at	PPM1E	0	-3.640917	9	1
236492_at	PPP2R2A	0.0060746	-1.159937	12	3
237003_at	BEST3	0.0130189	0.315394	11	2
237045_at	FAM91A1	0.0020564	0.407637	15	1
238041_at	TCF12	0.0003964	-1.090261	10	1
238472_at	FBXO9	0.0017717	-0.892198	6	2
238592_at	PDLIM3	0.0000049	-1.570477	2	1
238657_at	UBXN10	0.0002818	-2.409883	2	2
238719_at	PPP2CA	0.0009934	-1.66955	6	3
238738_at	PSMD7	0.0014408	-0.880911	4	1
238787_at	DENND1B	0.0015857	-1.410984	8	4
238868_at	UACA	0.000081	-1.518461	6	1
238877_at	EYA4	0.0740207	1.269938	14	1
238878_at	ARX	0.0000031	-5.267873	3	1
239178_at	FGF9	0.0013543	-3.013395	8	2
239349_at	C1QTNF7	0.0013485	-1.226701	3	1
239433_at	LRRC8E	0.0814692	0.590633	1	2
240221_at	CSNK1A1	0.0175225	-1.075974	6	1
240383_at	UBE2D3	0.0116239	-1.122395	12	2
240554_at	AKAP8L	0.0158737	0.47108	1	1
240650_at	CACNA1E	0.0507288	0.257667	2	1
241348_at	ZNF654	0.0151608	-1.16381	9	1
241372_at	ZC3H6	0.0002774	-0.784133	9	1
241789_at	RBMS3	0.0064964	-1.063511	13	1
241820_at	RIF1	0.0017131	0.357774	12	1
242271_at	SLC26A9	0.0132731	0.715562	3	3
242290_at	TACC1	0.0022464	-0.987385	6	1
242560_at	FANCD2	0.0000008	2.803878	13	1
242767_at	LMCD1	0.0306071	0.613395	4	2
243166_at	SLC30A5	0.0019163	-1.400463	10	1
243386_at	CASZ1	0.0078234	0.852074	5	2
244509_at	GPR155	0.0134593	0.387051	2	1
244738_at	BRWD3	0.0374175	0.526945	4	1
244804_at	SQSTM1	0.0204355	0.417988	7	2
33323_r_at	SFN	0.0000027	3.795532	1	1
35150_at	CD40	0.0082659	0.172515	3	1
39582_at	CYLD	0.0002318	-1.31074	15	2
39891_at	ZNF710	0.0013918	0.82692	2	1

40225_at	GAK	0.0046212	1.006518	4	1
41047_at	C9orf16	0.0052489	0.877502	2	1
44563_at	WRAP53	0.0062957	0.616404	2	1
46665_at	SEMA4C	0.0005812	1.442729	10	1
47608_at	TJAP1	0.0025456	0.999296	3	1
91826_at	EPS8L1	0.035378	0.989912	2	1
